# Supplementary material for: Identification and Validation of STC1 Act as a Biomarker for High-Altitude Diseases and Its Pan-Cancer Analysis
Source: Int J Mol Sci. 2024 Aug 21;25(16):9085. doi: 10.3390/ijms25169085 (PMC11354978; doi:10.3390/ijms25169085)
Supplement: Supplementary file 1 [file ijms-25-09085-s001.zip › Supplementary Figures S1 and S2.pdf]

Supplementary Materials

Supplementary Figure S1. Correlation between STC1 gene expression and disease-specific survival.

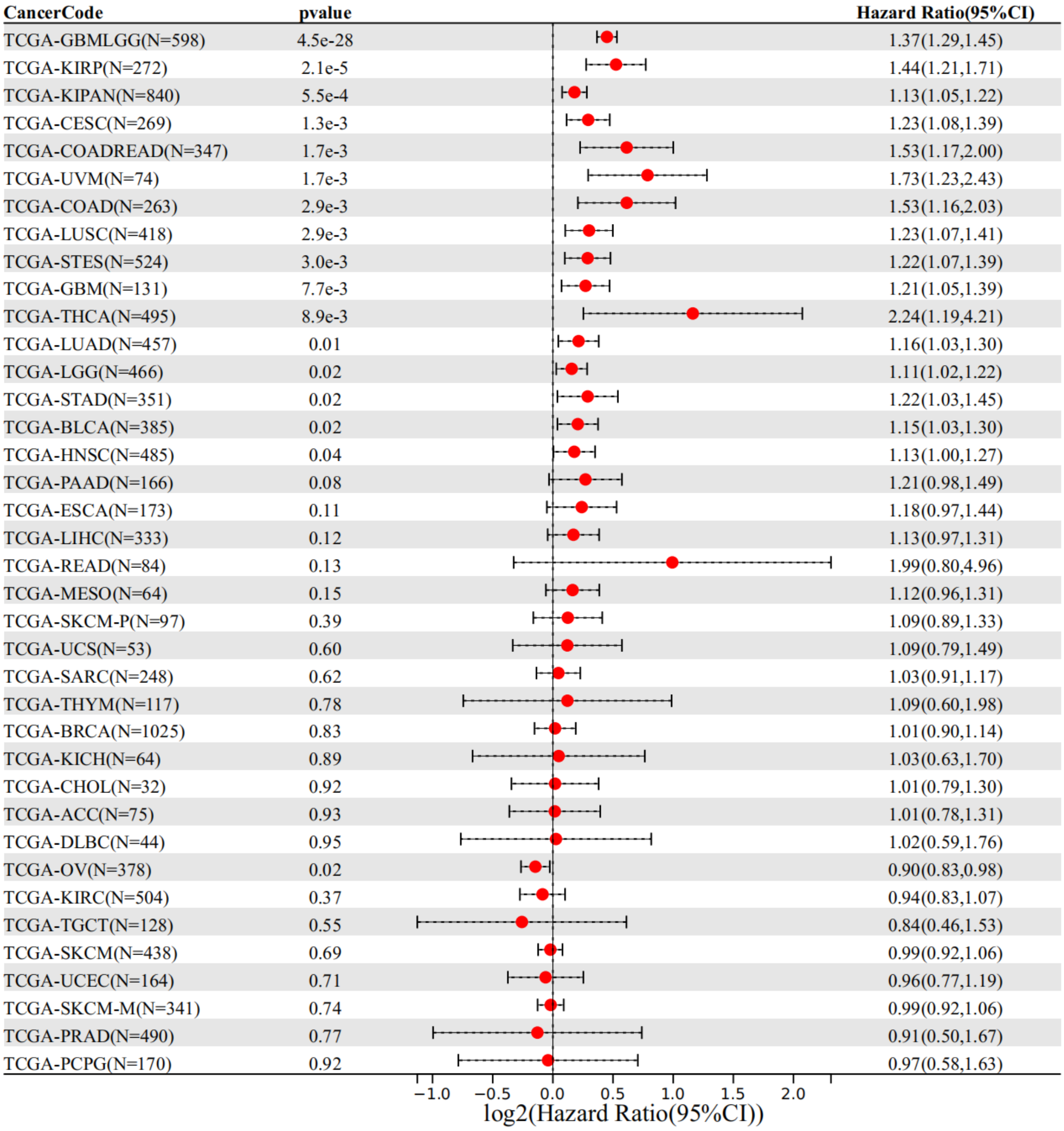

Supplementary Figure S2. Correlation between STC1 gene expression and progression-free interval.

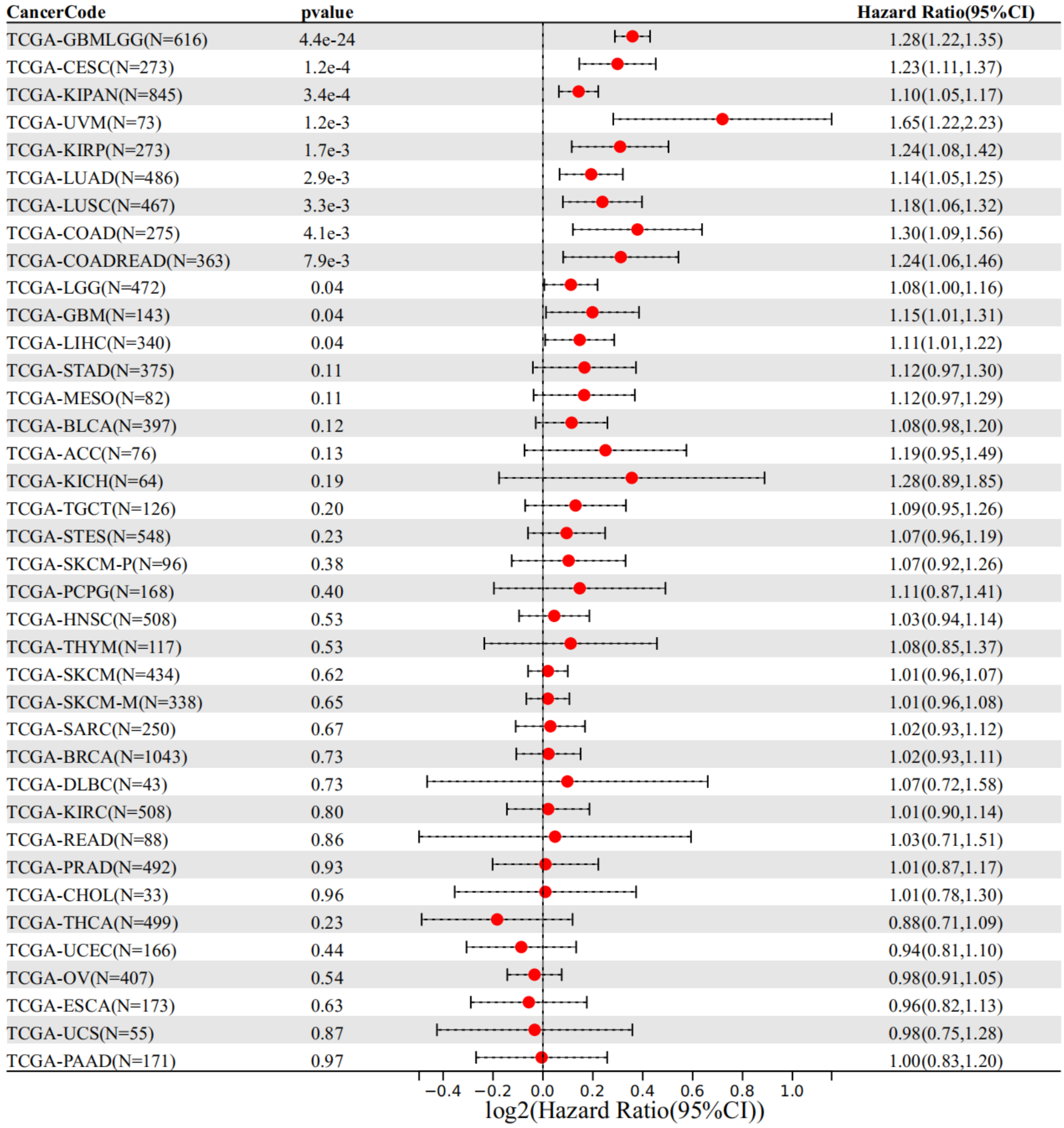

CancerCode
pvalue
Hazard Ratio(95%CI)
TCGA-GBMLGG(N=616) 4.4e-24 1.28(1.22,1.35)
TCGA-CESC(N=273) 1.2e-4 1.23(1.11,1.37)
TCGA-KIPAN(N=845) 3.4e-4 1.10(1.05,1.17)
TCGA-UVM(N=73) 1.2e-3 1.65(1.22,2.23)
TCGA-KIRP(N=273) 1.7e-3 1.24(1.08,1.42)
TCGA-LUAD(N=486) 2.9e-3 1.14(1.05,1.25)
TCGA-LUSC(N=467) 3.3e-3 1.18(1.06,1.32)
TCGA-COAD(N=275) 4.1e-3 1.30(1.09,1.56)
TCGA-COADREAD(N=363) 7.9e-3 1.24(1.06,1.46)
TCGA-LGG(N=472) 0.04 1.08(1.00,1.16)
TCGA-GBM(N=143) 0.04 1.15(1.01,1.31)
TCGA-LIHC(N=340) 0.04 1.11(1.01,1.22)
TCGA-STAD(N=375) 0.11 1.12(0.97,1.30)
TCGA-MESO(N=82) 0.11 1.12(0.97,1.29)
TCGA-BLCA(N=397) 0.12 1.08(0.98,1.20)
TCGA-ACC(N=76) 0.13 1.19(0.95,1.49)
TCGA-KICH(N=64) 0.19 1.28(0.89,1.85)
TCGA-TGCT(N=126) 0.20 1.09(0.95,1.26)
TCGA-STES(N=548) 0.23 1.07(0.96,1.19)
TCGA-SKCM-P(N=96) 0.38 1.07(0.92,1.26)
TCGA-PCPG(N=168) 0.40 1.11(0.87,1.41)
TCGA-HNSC(N=508) 0.53 1.03(0.94,1.14)
TCGA-THYM(N=117) 0.53 1.08(0.85,1.37)
TCGA-SKCM(N=434) 0.62 1.01(0.96,1.07)
TCGA-SKCM-M(N=338) 0.65 1.01(0.96,1.08)
TCGA-SARC(N=250) 0.67 1.02(0.93,1.12)
TCGA-BRCA(N=1043) 0.73 1.02(0.93,1.11)
TCGA-DLBC(N=43) 0.73 1.07(0.72,1.58)
TCGA-KIRC(N=508) 0.80 1.01(0.90,1.14)
TCGA-READ(N=88) 0.86 1.03(0.71,1.51)
TCGA-PRAD(N=492) 0.93 1.01(0.87,1.17)
TCGA-CHOL(N=33) 0.96 1.01(0.78,1.30)
TCGA-THCA(N=499) 0.23 0.88(0.71,1.09)
TCGA-UCEC(N=166) 0.44 0.94(0.81,1.10)
TCGA-OV(N=407) 0.54 0.98(0.91,1.05)
TCGA-ESCA(N=173) 0.63 0.96(0.82,1.13)
TCGA-UCS(N=55) 0.87 0.98(0.75,1.28)
TCGA-PAAD(N=171) 0.97 1.00(0.83,1.20)
−0.4 −0.2 0.0 0.2 0.4 0.6 0.8 1.0
log2(Hazard Ratio(95%CI))
