# Supplementary material for: Identification and Validation of STC1 Act as a Biomarker for High-Altitude Diseases and Its Pan-Cancer Analysis
Source: Int J Mol Sci. 2024 Aug 21;25(16):9085. doi: 10.3390/ijms25169085 (PMC11354978; doi:10.3390/ijms25169085)
Supplement: Supplementary file 1 [file ijms-25-09085-s001.zip › Supplementary Table S1.pdf]

Supplementary Table S1. The primers

**RAW 264.7 cell**	
GAPDH-F	5’-GCATCTTCTTGTGCAGTGCC-3’
GAPDH-R	5’-ACTGTGCCGTTGAATTTGCC-3’
STC1-F	5’-AACAAAATGATTCTGTGAGCCC-3’
STC1-R	5’-TTGACAAATGCTTTTCCCTGAG -3’
**blood from HAPE patients and control volunteers**	
GAPDH-F	5’-TGGCCAAGGTCATCCATGACAAC-3’
GAPDH-R	5’-TCCAGAGGGGCCATCCACAGTCTTCTG-3’
STC1-F	5’-AGCGCTGCTAAATTTGACACT-3’
STC1-R	5’-CTTTGGAAAGTGGAGCACCTCCG-3’
