# Supplementary material for: Identification and Validation of STC1 Act as a Biomarker for High-Altitude Diseases and Its Pan-Cancer Analysis
Source: Int J Mol Sci. 2024 Aug 21;25(16):9085. doi: 10.3390/ijms25169085 (PMC11354978; doi:10.3390/ijms25169085)
Supplement: Supplementary file 1 [file ijms-25-09085-s001.zip › Supplementary Table S2.pdf]

Supplementary Table S2.DE-miRNA of GSE90500

| miRNA_ID | logFC | t | adj.P.Val | P.Value | B |
|---|---|---|---|---|---|
| hsa-miR-15b-5p | -2.59 | -2.9 | 0.17 | 0.01 | -2.59 |
| hsa-miR-155-5p | -2.21 | -3.1 | 0.14 | 0.01 | -2.16 |
| hsa-miR-20a-5p | -1.93 | -2.39 | 0.27 | 0.03 | -3.6 |
| hsa-miR-877-3p | -1.55 | -2.49 | 0.25 | 0.02 | -3.41 |
| hsa-miR-1301 | -1.51 | -3.32 | 0.11 | 0 | -1.69 |
| hsa-miRPlus-A1025 | -1.5 | -3.19 | 0.14 | 0 | -1.96 |
| hsa-miR-1273a | -1.36 | -2.93 | 0.16 | 0.01 | -2.52 |
| hsa-miR-106b-3p | -1.3 | -2.75 | 0.19 | 0.01 | -2.89 |
| hsa-miR-423-5p | -1.27 | -2.37 | 0.27 | 0.03 | -3.63 |
| hsa-miR-3591-3p | -1.24 | -2.19 | 0.33 | 0.04 | -3.96 |
| hsa-miR-4787-5p | -1.13 | -2.42 | 0.27 | 0.02 | -3.55 |
| hsa-miR-4762-5p | -1.08 | -3.41 | 0.09 | 0 | -1.48 |
| hsa-miR-4646-3p | -1.07 | -2.14 | 0.35 | 0.04 | -4.06 |
| hsa-miR-3199 | -1.02 | -2.44 | 0.26 | 0.02 | -3.51 |
| hsa-miR-144-5p | -1.02 | -2.08 | 0.37 | 0.05 | -4.15 |
| hsa-miR-218-5p | 1.1 | 2.95 | 0.16 | 0.01 | -2.48 |
| hsa-miR-1908 | 1.11 | 3.64 | 0.06 | 0 | -0.98 |
| hsa-miRPlus-A1015 | 1.11 | 2.6 | 0.23 | 0.02 | -3.2 |
| hsa-miR-4712-3p | 1.15 | 2.85 | 0.17 | 0.01 | -2.69 |
| hsa-miR-4726-5p | 1.16 | 4.05 | 0.04 | 0 | -0.04 |
| hsa-miR-3173-3p | 1.28 | 2.28 | 0.3 | 0.03 | -3.81 |
