# Supplementary material for: Identification and Validation of STC1 Act as a Biomarker for High-Altitude Diseases and Its Pan-Cancer Analysis
Source: Int J Mol Sci. 2024 Aug 21;25(16):9085. doi: 10.3390/ijms25169085 (PMC11354978; doi:10.3390/ijms25169085)
Supplement: Supplementary file 1 [file ijms-25-09085-s001.zip › Supplementary Table S3.pdf]

Supplementary Table S3.DE-mRNAs of GSE52209

Tag	logFC	AveExpr	t	P.Value	adj.P.Val	B
FBXW11	1.69	9.98	6.36	0	0	6.76
NAXE	1.52	9.35	6.34	0	0	6.73
EGLN3	1.25	7.84	6.26	0	0	6.5
CA9	1.36	8.56	6.19	0	0	6.31
SLC28A2	2.1	9.6	6.13	0	0	6.13
TTLL4	1.85	9.91	5.83	0	0	5.31
PEX10	1.46	9.05	5.82	0	0	5.27
MOXD1	1.63	8.06	5.67	0	0	4.86
HSP90AB3P	-1.85	11.19	-5.61	0	0	4.7
CPT2	2.29	9.6	5.6	0	0	4.66
RAB39B	-1.18	7.84	-5.57	0	0	4.59
PDCL	1.17	7.6	5.55	0	0	4.54
PGBD5	1.1	7.35	5.52	0	0	4.44
KIRREL3	1.21	7.71	5.48	0	0	4.33
CAMK1G	1.34	8.46	5.48	0	0	4.33
DXO	1.76	9.8	5.41	0	0	4.13
SEC61A1	1.08	8.1	5.38	0	0	4.05
TACC1	1.13	9.05	5.36	0	0	4.01
HSD17B14	2.35	10.55	5.36	0	0	4.01
LY6G5C	1.98	9.74	5.35	0	0	3.97
IMPACT	1.99	8.47	5.34	0	0	3.96
PCYOX1L	1.17	8.33	5.33	0	0	3.91
EFCAB3	1.46	8.55	5.28	0	0	3.78
GZMM	1.11	8.46	5.18	0	0.01	3.51
IL11RA	1.6	9.23	5.17	0	0.01	3.48
ECE1	1.55	9.5	5.15	0	0.01	3.42
BSND	1.64	9.45	5.12	0	0.01	3.33
TM9SF1	1.32	7.6	5.07	0	0.01	3.21
DPCD	1.28	8.57	5.04	0	0.01	3.13
LIMS2	1.59	9.84	5.03	0	0.01	3.1
KIFAP3	1.41	7.64	5.02	0	0.01	3.08
PDIA6	1.33	8.76	4.95	0	0.01	2.86
DNAJA4	1.93	9.95	4.91	0	0.01	2.77
GUCD1	1.23	8.92	4.88	0	0.01	2.66
STX18	1.58	9.38	4.87	0	0.01	2.64
MTCL1	1.25	8.76	4.85	0	0.01	2.6
AP5S1	1.65	9.34	4.85	0	0.01	2.59
CECR7	1.16	7.55	4.83	0	0.01	2.53
CLCN1	1.03	8.76	4.82	0	0.01	2.52
TMEM161A	1.51	10.63	4.82	0	0.01	2.51
NRBF2	1.63	10.81	4.81	0	0.01	2.48
KCNN2	1.88	11.62	4.79	0	0.01	2.42

GIMAP7	-1.9	9.95	-4.77	0	0.01	2.37
OR1A1	1.03	8.15	4.76	0	0.01	2.36
SLC3A2	1.56	10.83	4.76	0	0.01	2.35
BLCAP	1.11	8.53	4.75	0	0.01	2.31
CRYGC	1.69	9.64	4.72	0	0.01	2.25
IMPDH1	1.29	9.01	4.71	0	0.01	2.21
ARRDC1	1.19	7.77	4.7	0	0.01	2.2
DEFB118	1.06	7.58	4.67	0	0.01	2.11
CHST6	1.23	7.78	4.66	0	0.01	2.07
ANKH	1.59	10.64	4.66	0	0.01	2.07
PTCD1	1.27	8.21	4.64	0	0.01	2.02
HSPB2	1.1	8.34	4.61	0	0.01	1.95
PDK2	1.28	10.21	4.57	0	0.01	1.83
SLC39A5	1.15	7.53	4.56	0	0.01	1.8
MBL2	1.52	9.81	4.53	0	0.02	1.71
HSH2D	1.21	8.42	4.5	0	0.02	1.63
PAX2	1.03	8.18	4.5	0	0.02	1.63
SSTR5	1.6	9.32	4.49	0	0.02	1.62
SPNS1	1.48	9.21	4.49	0	0.02	1.6
NCMAP	-1.16	10.41	-4.48	0	0.02	1.58
KIAA2013	1.49	8.87	4.47	0	0.02	1.57
DDX11	1.32	8.78	4.46	0	0.02	1.53
RPLP0	-2.08	9.2	-4.45	0	0.02	1.52
MICOS10	-1.61	9.14	-4.45	0	0.02	1.51
PAGR1	1.17	7.67	4.43	0	0.02	1.46
PIP4P1	1.44	8.51	4.42	0	0.02	1.43
HOXD4	1.17	8.31	4.42	0	0.02	1.43
HERC6	1.08	9.76	4.38	0	0.02	1.32
NOTCH4	1.17	8.95	4.37	0	0.02	1.29
HAX1	1.12	8.46	4.36	0	0.02	1.25
CCDC62	1.16	8.85	4.35	0	0.02	1.24
COX7A2P2	-1.17	7.93	-4.35	0	0.02	1.23
SLC9A3R1	1.75	10.12	4.35	0	0.02	1.22
SATB2	1.14	8.51	4.34	0	0.02	1.19
TROAP	1.09	8.35	4.33	0	0.02	1.19
MTHFR	1.05	8.97	4.33	0	0.02	1.19
DUX4	1.06	9.01	4.33	0	0.02	1.19
OPA3	1.21	8.82	4.32	0	0.02	1.14
RASSF7	1.89	10.12	4.31	0	0.02	1.13
FAM171A2	-1.1	8.97	-4.31	0	0.02	1.13
SNHG12	-1.07	7.6	-4.31	0	0.02	1.12
YIF1A	-1.1	9.8	-4.31	0	0.02	1.12
LOC388242	1.27	8.76	4.26	0	0.02	0.98
TTC7A	1.44	9.09	4.24	0	0.02	0.93

KDM5A	1.38	9.09	4.23	0	0.02	0.92
PDCD1LG2	1.18	9.3	4.22	0	0.02	0.88
KIAA1211	1.22	9.81	4.22	0	0.02	0.88
EEF1G	-1.03	7.47	-4.22	0	0.02	0.87
GATAD2B	1.57	9.86	4.21	0	0.02	0.85
LRP5	1.55	11	4.2	0	0.02	0.82
SCAMP3	1.71	9.43	4.19	0	0.02	0.79
NKAIN1	1.08	7.87	4.17	0	0.02	0.75
DESI1	1.9	10.92	4.16	0	0.02	0.74
SOS1	1.1	8.71	4.16	0	0.02	0.73
ARRB2	1.39	11.11	4.16	0	0.02	0.72
GHSR	1.13	10.23	4.15	0	0.02	0.71
TMEM175	1.24	8.88	4.15	0	0.02	0.7
WWC2	1.11	8.68	4.15	0	0.02	0.69
ADAMTS7	1.18	8.39	4.13	0	0.02	0.66
MFNG	1.18	8.54	4.13	0	0.02	0.66
UBOX5	1.42	8.58	4.13	0	0.02	0.65
JAK2	1.18	9.22	4.13	0	0.02	0.65
VPS28	1.09	8.54	4.13	0	0.02	0.64
FBXL3	-1.07	6.35	-4.12	0	0.03	0.62
SOX7	1.69	10.6	4.12	0	0.03	0.6
OR3A4P	-1.21	10.71	-4.1	0	0.03	0.57
HECTD2	-1.03	8.15	-4.1	0	0.03	0.56
SIGLEC17P	-1.02	9.31	-4.1	0	0.03	0.56
SOSTDC1	-1.05	7.62	-4.09	0	0.03	0.54
MAP2K2	1.26	9.12	4.08	0	0.03	0.5
C20orf85	-1.11	9.88	-4.07	0	0.03	0.48
DRD1	1.07	7.81	4.07	0	0.03	0.48
STK10	1.35	9.19	4.06	0	0.03	0.46
ARMC9	1.5	8.89	4.06	0	0.03	0.45
IL22RA1	1.34	9.85	4.05	0	0.03	0.44
IGKC	-1.09	8.85	-4.05	0	0.03	0.44
NKD1	1.39	9.11	4.02	0	0.03	0.37
MDC1	1.49	9.35	4.01	0	0.03	0.33
LINC01006	1.35	9.48	4.01	0	0.03	0.32
ZNF519	1.68	11.02	3.99	0	0.03	0.27
C7orf50	1.05	8.07	3.98	0	0.03	0.26
KCNV1	1.24	9.96	3.98	0	0.03	0.25
ATXN2L	1.29	9.38	3.97	0	0.03	0.23
SALL2	1.09	8.64	3.95	0	0.03	0.18
C19orf24	1.72	10.86	3.94	0	0.03	0.16
SLURP1	1.04	8.69	3.94	0	0.03	0.14
ACKR1	1.4	9.43	3.93	0	0.03	0.13
LY6E	1.16	8.92	3.93	0	0.03	0.11

APOL5	1.4	9.77	3.92	0	0.03	0.1
CD5	1.23	8.44	3.92	0	0.03	0.09
CLIP3	1.23	9.27	3.91	0	0.03	0.07
EIPR1	1.24	9.35	3.91	0	0.03	0.06
STC1	1.52	11.56	3.91	0	0.03	0.06
FOXB1	1.09	8.6	3.91	0	0.03	0.06
RPP25	1.17	8.44	3.9	0	0.03	0.05
BARHL2	-1.58	10.01	-3.89	0	0.03	0
UNC50	1.12	8.72	3.88	0	0.03	-0.01
LONP1	1.52	9.37	3.87	0	0.03	-0.03
ASCL3	1.04	8.56	3.85	0	0.03	-0.08
ARHGEF11	1.31	9.84	3.85	0	0.03	-0.09
GRIN2C	1.13	8.55	3.85	0	0.03	-0.1
POM121L8P	1.51	10.08	3.84	0	0.03	-0.12
KCNK9	1.15	8.28	3.83	0	0.03	-0.14
SYVN1	-1.16	9.86	-3.83	0	0.03	-0.15
GNRH1	1.03	10.96	3.8	0	0.04	-0.23
PTPN18	1.69	9.61	3.79	0	0.04	-0.23
KRT35	1.77	10.12	3.77	0	0.04	-0.29
ZC3H12A	1.29	8.52	3.77	0	0.04	-0.3
SLC29A2	1.46	10.57	3.77	0	0.04	-0.3
DBNDD1	1.21	9.44	3.74	0	0.04	-0.37
KRT86	1.1	8.27	3.73	0	0.04	-0.39
CNTNAP1	1.13	9.11	3.73	0	0.04	-0.39
C3orf52	1.28	8.62	3.73	0	0.04	-0.4
NIPA1	-1.05	7.98	-3.73	0	0.04	-0.4
E2F2	1.16	8.32	3.72	0	0.04	-0.42
PPM1F	1.53	9	3.72	0	0.04	-0.42
YJEFN3	1.42	10.12	3.72	0	0.04	-0.43
ADCYAP1R1	1.08	8.63	3.71	0	0.04	-0.46
TTYH2	1.07	9.44	3.7	0	0.04	-0.47
CASKIN1	1.16	9.22	3.69	0	0.04	-0.5
EVA1B	1.46	9.36	3.68	0	0.04	-0.52
FKSG49	-1.39	10.08	-3.68	0	0.04	-0.52
CXXC4	1.51	9.33	3.68	0	0.04	-0.52
ZNF703	1.08	10.28	3.68	0	0.04	-0.52
WBSCR23	1.44	9.22	3.68	0	0.04	-0.53
UBA52P3	1.25	9.57	3.68	0	0.04	-0.54
GCKR	1.02	8.8	3.67	0	0.04	-0.54
MVK	1.15	10.13	3.66	0	0.04	-0.57
RHPN1-AS1	1.12	9.45	3.66	0	0.04	-0.58
C10orf88	1.23	9.36	3.65	0	0.04	-0.6
ESPL1	1.02	7.73	3.65	0	0.04	-0.6
NMRK1	1.28	8.72	3.65	0	0.04	-0.61

NKX2-8	1.33	9.46	3.63	0	0.04	-0.66
TRPV5	1.01	8.48	3.62	0	0.04	-0.68
VKORC1L1	-1	6.21	-3.62	0	0.04	-0.68
GPR61	1.23	8.2	3.62	0	0.04	-0.68
NPW	-1.03	9.44	-3.61	0	0.04	-0.71
ZNF496	1.11	8.55	3.6	0	0.04	-0.72
GSN-AS1	1.16	9.86	3.6	0	0.04	-0.73
EFNA5	1.1	8.64	3.6	0	0.04	-0.74
GORASP2	1.19	9.32	3.6	0	0.04	-0.74
LYPLA2	1.75	10.08	3.59	0	0.04	-0.76
RPSA	-1.41	9.17	-3.57	0	0.04	-0.8
EMG1	1.07	8.63	3.57	0	0.04	-0.82
HTR1D	1.36	9.89	3.56	0	0.04	-0.83
STK19	1.13	10.05	3.55	0	0.05	-0.86
IGLL1	-1.17	9.08	-3.55	0	0.05	-0.86
GNG13	1.19	9.04	3.54	0	0.05	-0.88
RAPGEF4	1.04	8.4	3.54	0	0.05	-0.89
ODF3L1	1.13	8.82	3.53	0	0.05	-0.9
MAP6D1	1.1	8.68	3.53	0	0.05	-0.92
RAD54L	1.64	10.35	3.53	0	0.05	-0.92
PROK1	1.01	8.39	3.52	0	0.05	-0.94
SRM	1.23	9.35	3.51	0	0.05	-0.95
PROCR	1.41	9.78	3.48	0	0.05	-1.02
GPR88	1.31	9.99	3.48	0	0.05	-1.03
HSPA8	-1.06	9.58	-3.48	0	0.05	-1.03
CAP1	1.31	12.78	3.47	0	0.05	-1.04
YPEL2	2.51	8.82	3.45	0	0.05	-1.1
MADCAM1	1.21	9.86	3.44	0	0.05	-1.13
GPR3	1.37	10.1	3.43	0	0.05	-1.15
COX6B1	-1.03	9.41	-3.43	0	0.05	-1.15
ZNF576	1.57	10.65	3.43	0	0.05	-1.16
PEA15	1.1	9.4	3.42	0	0.05	-1.18
ROGDI	1.01	9.74	3.42	0	0.05	-1.19
TNNI1	1.06	7.96	3.41	0	0.05	-1.2
DDX4	1.21	9.34	3.39	0	0.05	-1.24
LMF1	1.04	8.35	3.39	0	0.05	-1.24
NRIP2	1.19	9.11	3.39	0	0.05	-1.25
HBG2	1.77	10.55	3.37	0	0.06	-1.3
FUT3	1.69	10.81	3.37	0	0.06	-1.3
HCG4	1.13	9.5	3.36	0	0.06	-1.32
MSLN	1.03	8.42	3.36	0	0.06	-1.33
CYREN	1.12	8.68	3.35	0	0.06	-1.35
NPHS1	1.12	8.7	3.34	0	0.06	-1.36
LOC391813	1.17	8.76	3.34	0	0.06	-1.38

ICAM1	1.47	11.36	3.33	0	0.06	-1.41
DUOX1	1.22	9.46	3.32	0	0.06	-1.41
GSC2	1.03	8.26	3.31	0	0.06	-1.45
CALCOCO2	1.45	8.66	3.3	0	0.06	-1.47
PSENEN	-1.08	8.84	-3.29	0	0.06	-1.5
GFRA2	1.25	8.97	3.29	0	0.06	-1.5
EPHA1	1.13	8.17	3.27	0	0.06	-1.55
STOML2	1.21	8.19	3.26	0	0.06	-1.56
RAB9B	1.1	7.57	3.23	0	0.07	-1.64
CEP68	1.31	8.94	3.21	0	0.07	-1.69
PLAGL1	1.09	10.14	3.19	0	0.07	-1.74
ZNF428	-1.02	7.26	-3.17	0	0.07	-1.79
RNF167	1.32	9.46	3.17	0	0.07	-1.8
BPIFB2	1.25	8.71	3.16	0	0.07	-1.82
CCDC26	-1.05	8	-3.15	0	0.07	-1.84
MT1F	1.02	8.46	3.14	0	0.07	-1.85
OTOS	-1.08	9.43	-3.14	0	0.07	-1.86
IGFLR1	1.47	10.09	3.13	0	0.07	-1.87
LGALS9	1.49	9.33	3.13	0	0.07	-1.88
NEUROG2	1.2	9.14	3.11	0	0.07	-1.92
TAS2R1	1.01	9.78	3.09	0	0.08	-1.96
CYP2U1	-1.44	8.71	-3.09	0	0.08	-1.97
SCAND2P	1.44	11.77	3.09	0	0.08	-1.98
GAPDHS	1.08	9.98	3.09	0	0.08	-1.98
DYRK1B	1.02	9.3	3.08	0	0.08	-2
TSPAN10	1.15	10.39	3.06	0	0.08	-2.05
CNPPD1	1.19	9.57	3.03	0	0.08	-2.1
NBPF10	-1.21	7.59	-3.02	0	0.08	-2.14
ZNF580	1.32	10.8	3.01	0	0.08	-2.15
NCKAP1L	1.39	9.56	3	0	0.08	-2.19
NKX6-1	1.1	8.51	2.99	0	0.08	-2.2
GPR165P	1.27	10.31	2.99	0.01	0.08	-2.2
ATXN7	1.05	8.46	2.98	0.01	0.09	-2.23
BCL3	1.02	10.02	2.97	0.01	0.09	-2.25
SIX5	-1.04	8.99	-2.96	0.01	0.09	-2.26
IGLV2-8	1.14	10.83	2.96	0.01	0.09	-2.26
KCNK12	1.12	9.13	2.96	0.01	0.09	-2.28
TALDO1	1.04	9.21	2.95	0.01	0.09	-2.3
SHH	1.01	9.35	2.95	0.01	0.09	-2.3
BOK	1.06	8.97	2.93	0.01	0.09	-2.34
TMEM196	1.39	9.78	2.92	0.01	0.09	-2.36
IFI27	1.54	8.94	2.92	0.01	0.09	-2.36
RNF112	1.09	8.8	2.92	0.01	0.09	-2.37
SLC4A10	1.17	9.43	2.9	0.01	0.09	-2.4

OAZ3	1.25	11.47	2.89	0.01	0.09	-2.43
C20orf144	-1.02	8.58	-2.87	0.01	0.1	-2.48
PRKAR1B	-1.01	9.11	-2.86	0.01	0.1	-2.49
NRN1	1.01	8.38	2.86	0.01	0.1	-2.5
SCGB3A1	1.05	7.66	2.83	0.01	0.1	-2.56
TFEB	1.01	9.81	2.78	0.01	0.11	-2.67
RNF39	1.05	7.87	2.78	0.01	0.11	-2.68
SSRP1	-1.16	9.3	-2.78	0.01	0.11	-2.68
PNMT	1.01	12.12	2.74	0.01	0.11	-2.76
CD177	1.2	10.68	2.74	0.01	0.11	-2.76
SLC22A12	-1.09	10.13	-2.73	0.01	0.11	-2.78
RNF10	1.38	11.54	2.73	0.01	0.11	-2.8
ARRDC2	1.13	9.8	2.72	0.01	0.11	-2.8
ROR2	1.07	8.14	2.72	0.01	0.12	-2.82
EFNA2	1.3	10.13	2.71	0.01	0.12	-2.84
RDH12	1.04	8.91	2.69	0.01	0.12	-2.88
TIPIN	1.31	8.22	2.65	0.01	0.13	-2.96
BHLHE41	1.22	9.8	2.65	0.01	0.13	-2.96
CLDN6	1.09	8.84	2.64	0.01	0.13	-2.97
ZBED2	1.14	9.29	2.64	0.01	0.13	-2.99
MRPS18A	1.07	8.82	2.62	0.01	0.13	-3.02
HEXA	1.21	9.61	2.58	0.01	0.14	-3.11
SLC8A2	1.21	9.48	2.5	0.02	0.15	-3.28
GBX1	1.05	8.43	2.49	0.02	0.15	-3.3
ASMTL	1.21	9.75	2.48	0.02	0.15	-3.31
ARPC3P1	-1.07	9.62	-2.47	0.02	0.15	-3.33
AMHR2	1.16	9.58	2.47	0.02	0.15	-3.33
FEM1B	1.14	9.43	2.47	0.02	0.15	-3.34
IFNWP2	-1.01	7.73	-2.47	0.02	0.15	-3.34
LY6G6E	1.23	11.03	2.46	0.02	0.15	-3.35
B2M	-1.11	8.21	-2.46	0.02	0.15	-3.35
MRPL45	1.12	9.47	2.46	0.02	0.16	-3.36
SLC38A3	1.2	9.14	2.43	0.02	0.16	-3.42
TRPV1	1.01	9.53	2.38	0.02	0.17	-3.52
CDC42EP3	1.02	11.53	2.31	0.03	0.18	-3.66
ZNF385D	1.18	10.55	2.3	0.03	0.18	-3.67
OR10C1	1.11	9.41	2.26	0.03	0.19	-3.75
ZNHIT1	1.18	10.56	2.26	0.03	0.19	-3.76
LMLN	1	9.88	2.22	0.03	0.2	-3.82
PROCA1	-1.07	11	-2.19	0.03	0.2	-3.87
IFI27L2	1.07	8.57	2.18	0.04	0.21	-3.91
GDPD3	1.12	9.32	2.12	0.04	0.22	-4.01
TNNI3K	1.06	7.6	2.08	0.04	0.22	-4.08
