# Supplementary material for: Identification and Validation of STC1 Act as a Biomarker for High-Altitude Diseases and Its Pan-Cancer Analysis
Source: Int J Mol Sci. 2024 Aug 21;25(16):9085. doi: 10.3390/ijms25169085 (PMC11354978; doi:10.3390/ijms25169085)
Supplement: Supplementary file 1 [file ijms-25-09085-s001.zip › Supplementary Table S4.pdf]

Supplementary Table S4. Target mRNA of DE-miRNAs

Total DE-miRNAs	miRNA	Gene	miRDB	miRTarBase	TargetScan	Sum
	hsa-mir-155-5p	CCNT2	1	1	1	3
	hsa-mir-20a-5p	KATNAL1	1	1	1	3
	hsa-mir-3173-3p	MRRF	1	1	1	3
	hsa-mir-423-5p	PPARGC1B	1	1	1	3
	hsa-mir-155-5p	RREB1	1	1	1	3
	hsa-mir-155-5p	CDC73	1	1	1	3
	hsa-mir-423-5p	NFIX	1	1	1	3
	hsa-mir-15b-5p	STXBP3	1	1	1	3
	hsa-mir-20a-5p	SALL3	1	1	1	3
	hsa-mir-20a-5p	AGO1	1	1	1	3
	hsa-mir-20a-5p	LIMA1	1	1	1	3
	hsa-mir-3199	KDM6B	1	1	1	3
	hsa-mir-15b-5p	PRDM4	1	1	1	3
	hsa-mir-218-5p	COL4A1	1	1	1	3
	hsa-mir-155-5p	MITF	1	1	1	3
	hsa-mir-20a-5p	NABP1	1	1	1	3
	hsa-mir-155-5p	FOS	1	1	1	3
	hsa-mir-15b-5p	BAG4	1	1	1	3
	hsa-mir-15b-5p	KIF3B	1	1	1	3
	hsa-mir-423-5p	ADAMTS14	1	1	1	3
	hsa-mir-20a-5p	TMBIM6	1	1	1	3
	hsa-mir-15b-5p	C2orf42	1	1	1	3
	hsa-mir-15b-5p	C11orf24	1	1	1	3
	hsa-mir-20a-5p	EIF5A2	1	1	1	3
	hsa-mir-155-5p	LRIF1	1	1	1	3
	hsa-mir-20a-5p	TBC1D15	1	1	1	3
	hsa-mir-15b-5p	UBR3	1	1	1	3
	hsa-mir-15b-5p	HMBOX1	1	1	1	3
	hsa-mir-15b-5p	SEPT2	1	1	1	3
	hsa-mir-20a-5p	RB1	1	1	1	3
	hsa-mir-20a-5p	ARID4B	1	1	1	3
	hsa-mir-15b-5p	GABPA	1	1	1	3
	hsa-mir-20a-5p	TOPORS	1	1	1	3
	hsa-mir-155-5p	TAB2	1	1	1	3
	hsa-mir-20a-5p	POLR3G	1	1	1	3
	hsa-mir-877-3p	ZNF629	1	1	1	3
	hsa-mir-15b-5p	CDC27	1	1	1	3
	hsa-mir-155-5p	RAC1	1	1	1	3
	hsa-mir-20a-5p	ZNF280B	1	1	1	3
	hsa-mir-155-5p	PCDH9	1	1	1	3
	hsa-mir-15b-5p	UBN2	1	1	1	3

hsa-mir-155-5p	S1PR1	1	1	1	3
hsa-mir-155-5p	ZNF431	1	1	1	3
hsa-mir-15b-5p	RNF138	1	1	1	3
hsa-mir-423-5p	CBX6	1	1	1	3
hsa-mir-423-5p	NCS1	1	1	1	3
hsa-mir-15b-5p	SALL1	1	1	1	3
hsa-mir-15b-5p	KMT2D	1	1	1	3
hsa-mir-155-5p	ARID2	1	1	1	3
hsa-mir-20a-5p	OXR1	1	1	1	3
hsa-mir-218-5p	ITM2C	1	1	1	3
hsa-mir-155-5p	STRN3	1	1	1	3
hsa-mir-20a-5p	DNAJC27	1	1	1	3
hsa-mir-20a-5p	ZNF532	1	1	1	3
hsa-mir-15b-5p	GABARAPL1	1	1	1	3
hsa-mir-15b-5p	B4GALT1	1	1	1	3
hsa-mir-20a-5p	NHLRC3	1	1	1	3
hsa-mir-20a-5p	RAB11FIP1	1	1	1	3
hsa-mir-15b-5p	ACVR2A	1	1	1	3
hsa-mir-155-5p	PKIA	1	1	1	3
hsa-mir-155-5p	SMARCA4	1	1	1	3
hsa-mir-20a-5p	EPHA4	1	1	1	3
hsa-mir-20a-5p	CRIM1	1	1	1	3
hsa-mir-20a-5p	NRIP3	1	1	1	3
hsa-mir-218-5p	RNF219	1	1	1	3
hsa-mir-15b-5p	CPEB3	1	1	1	3
hsa-mir-20a-5p	SEMA4B	1	1	1	3
hsa-mir-3173-3p	SCD	1	1	1	3
hsa-mir-15b-5p	TMC7	1	1	1	3
hsa-mir-20a-5p	SLC16A9	1	1	1	3
hsa-mir-218-5p	KIRREL3	1	1	1	3
hsa-mir-20a-5p	GIGYF1	1	1	1	3
hsa-mir-15b-5p	SYPL1	1	1	1	3
hsa-mir-20a-5p	FAM117B	1	1	1	3
hsa-mir-20a-5p	PXK	1	1	1	3
hsa-mir-423-5p	ZBTB46	1	1	1	3
hsa-mir-20a-5p	SLAIN2	1	1	1	3
hsa-mir-20a-5p	TGFBR2	1	1	1	3
hsa-mir-218-5p	WIPF2	1	1	1	3
hsa-mir-15b-5p	ACTR2	1	1	1	3
hsa-mir-15b-5p	SMAD3	1	1	1	3
hsa-mir-20a-5p	SEMA7A	1	1	1	3
hsa-mir-218-5p	LYPD6B	1	1	1	3

hsa-mir-20a-5p	KPNA2	1	1	1	3
hsa-mir-15b-5p	NUCKS1	1	1	1	3
hsa-mir-155-5p	RORA	1	1	1	3
hsa-mir-20a-5p	ACSL4	1	1	1	3
hsa-mir-20a-5p	SACS	1	1	1	3
hsa-mir-15b-5p	AXIN2	1	1	1	3
hsa-mir-155-5p	RGL1	1	1	1	3
hsa-mir-155-5p	ZSWIM6	1	1	1	3
hsa-mir-15b-5p	TMEM100	1	1	1	3
hsa-mir-15b-5p	ACOX1	1	1	1	3
hsa-mir-877-3p	DLC1	1	1	1	3
hsa-mir-20a-5p	MORF4L1	1	1	1	3
hsa-mir-218-5p	SEPHS1	1	1	1	3
hsa-mir-15b-5p	PEX13	1	1	1	3
hsa-mir-20a-5p	CMPK1	1	1	1	3
hsa-mir-15b-5p	PPP6C	1	1	1	3
hsa-mir-3173-3p	PPP1R1C	1	1	1	3
hsa-mir-15b-5p	SHOC2	1	1	1	3
hsa-mir-218-5p	PNPLA8	1	1	1	3
hsa-mir-20a-5p	PTGFRN	1	1	1	3
hsa-mir-3173-3p	BSDC1	1	1	1	3
hsa-mir-218-5p	PHC3	1	1	1	3
hsa-mir-20a-5p	RBL2	1	1	1	3
hsa-mir-15b-5p	RFWD2	1	1	1	3
hsa-mir-877-3p	RGS6	1	1	1	3
hsa-mir-15b-5p	ELK4	1	1	1	3
hsa-mir-15b-5p	PURA	1	1	1	3
hsa-mir-15b-5p	CCDC88C	1	1	1	3
hsa-mir-20a-5p	NETO2	1	1	1	3
hsa-mir-15b-5p	ATP13A3	1	1	1	3
hsa-mir-155-5p	SDCBP	1	1	1	3
hsa-mir-155-5p	VEZF1	1	1	1	3
hsa-mir-155-5p	TRAM1	1	1	1	3
hsa-mir-1273a	RNF115	1	1	1	3
hsa-mir-20a-5p	SIK1	1	1	1	3
hsa-mir-15b-5p	KDR	1	1	1	3
hsa-mir-15b-5p	HSPA4L	1	1	1	3
hsa-mir-20a-5p	CAPN15	1	1	1	3
hsa-mir-20a-5p	E2F5	1	1	1	3
hsa-mir-877-3p	SCML2	1	1	1	3
hsa-mir-877-3p	GRAMD1B	1	1	1	3
hsa-mir-218-5p	TOB1	1	1	1	3
hsa-mir-155-5p	ANKFY1	1	1	1	3
hsa-mir-15b-5p	ARHGDIA	1	1	1	3

hsa-mir-218-5p	SHOC2	1	1	1	3
hsa-mir-20a-5p	LIMK1	1	1	1	3
hsa-mir-4762-5p	POT1	1	1	1	3
hsa-mir-15b-5p	PHC3	1	1	1	3
hsa-mir-423-5p	CALM3	1	1	1	3
hsa-mir-3199	KCNK3	1	1	1	3
hsa-mir-218-5p	LGR4	1	1	1	3
hsa-mir-423-5p	MESDC1	1	1	1	3
hsa-mir-15b-5p	BTRC	1	1	1	3
hsa-mir-20a-5p	TANC1	1	1	1	3
hsa-mir-20a-5p	UNK	1	1	1	3
hsa-mir-20a-5p	SPRED1	1	1	1	3
hsa-mir-15b-5p	KPNA1	1	1	1	3
hsa-mir-15b-5p	BTAF1	1	1	1	3
hsa-mir-15b-5p	CHIC1	1	1	1	3
hsa-mir-15b-5p	CPEB2	1	1	1	3
hsa-mir-218-5p	SPAG9	1	1	1	3
hsa-mir-20a-5p	ELAVL2	1	1	1	3
hsa-mir-155-5p	CARHSP1	1	1	1	3
hsa-mir-20a-5p	BAMBI	1	1	1	3
hsa-mir-155-5p	TYRP1	1	1	1	3
hsa-mir-155-5p	ADD3	1	1	1	3
hsa-mir-155-5p	HNRNPA3	1	1	1	3
hsa-mir-20a-5p	ITCH	1	1	1	3
hsa-mir-15b-5p	WIPI2	1	1	1	3
hsa-mir-1273a	LYZ	1	1	1	3
hsa-mir-3173-3p	CHD4	1	1	1	3
hsa-mir-20a-5p	SUCO	1	1	1	3
hsa-mir-15b-5p	YTHDC1	1	1	1	3
hsa-mir-155-5p	MIDN	1	1	1	3
hsa-mir-20a-5p	YOD1	1	1	1	3
hsa-mir-20a-5p	KLHL20	1	1	1	3
hsa-mir-423-5p	MFGE8	1	1	1	3
hsa-mir-3591-3p	CELF1	1	1	1	3
hsa-mir-155-5p	CAB39	1	1	1	3
hsa-mir-218-5p	KIF21B	1	1	1	3
hsa-mir-423-5p	MDM4	1	1	1	3
hsa-mir-218-5p	DCBLD2	1	1	1	3
hsa-mir-15b-5p	PPP1R11	1	1	1	3
hsa-mir-3591-3p	C5orf51	1	1	1	3
hsa-mir-155-5p	SP1	1	1	1	3
hsa-mir-20a-5p	CEP170	1	1	1	3
hsa-mir-20a-5p	LDLR	1	1	1	3
hsa-mir-15b-5p	CBFA2T3	1	1	1	3

hsa-mir-218-5p	RIT1	1	1	1	3
hsa-mir-20a-5p	NIN	1	1	1	3
hsa-mir-155-5p	MAP3K10	1	1	1	3
hsa-mir-155-5p	SPI1	1	1	1	3
hsa-mir-20a-5p	PANK3	1	1	1	3
hsa-mir-20a-5p	PIP4K2C	1	1	1	3
hsa-mir-20a-5p	SNTB2	1	1	1	3
hsa-mir-20a-5p	CNOT7	1	1	1	3
hsa-mir-20a-5p	ORMDL3	1	1	1	3
hsa-mir-155-5p	FAM135A	1	1	1	3
hsa-mir-20a-5p	ETV1	1	1	1	3
hsa-mir-20a-5p	RHOC	1	1	1	3
hsa-mir-20a-5p	FBXL5	1	1	1	3
hsa-mir-218-5p	NFATC3	1	1	1	3
hsa-mir-218-5p	OTUD7B	1	1	1	3
hsa-mir-218-5p	CDC42BPA	1	1	1	3
hsa-mir-155-5p	UBXN2B	1	1	1	3
hsa-mir-218-5p	IKBKB	1	1	1	3
hsa-mir-20a-5p	KIAA0513	1	1	1	3
hsa-mir-20a-5p	SAMD12	1	1	1	3
hsa-mir-20a-5p	RABEP1	1	1	1	3
hsa-mir-15b-5p	ATXN7L3B	1	1	1	3
hsa-mir-15b-5p	CREBRF	1	1	1	3
hsa-mir-20a-5p	FNBP1L	1	1	1	3
hsa-mir-20a-5p	BNIP2	1	1	1	3
hsa-mir-155-5p	FMNL2	1	1	1	3
hsa-mir-15b-5p	PDCD4	1	1	1	3
hsa-mir-15b-5p	AKAP11	1	1	1	3
hsa-mir-155-5p	VCPIP1	1	1	1	3
hsa-mir-218-5p	FAM13B	1	1	1	3
hsa-mir-218-5p	GLCE	1	1	1	3
hsa-mir-15b-5p	CDK17	1	1	1	3
hsa-mir-15b-5p	PLEKHA1	1	1	1	3
hsa-mir-218-5p	SLC25A36	1	1	1	3
hsa-mir-877-3p	SRCIN1	1	1	1	3
hsa-mir-15b-5p	CLSPN	1	1	1	3
hsa-mir-1273a	FAM129A	1	1	1	3
hsa-mir-20a-5p	MTF1	1	1	1	3
hsa-mir-15b-5p	AGO4	1	1	1	3
hsa-mir-15b-5p	PHF19	1	1	1	3
hsa-mir-15b-5p	CARD10	1	1	1	3
hsa-mir-20a-5p	TMEM167A	1	1	1	3
hsa-mir-15b-5p	ZFHX4	1	1	1	3
hsa-mir-218-5p	ETS2	1	1	1	3

hsa-mir-15b-5p	CASK	1	1	1	3
hsa-mir-4712-3p	SLC5A6	1	1	1	3
hsa-mir-20a-5p	PGM2L1	1	1	1	3
hsa-mir-15b-5p	HOXA10	1	1	1	3
hsa-mir-155-5p	TRIM32	1	1	1	3
hsa-mir-20a-5p	ZNFX1	1	1	1	3
hsa-mir-3173-3p	TOB2	1	1	1	3
hsa-mir-155-5p	TRIP13	1	1	1	3
hsa-mir-20a-5p	ITGB8	1	1	1	3
hsa-mir-20a-5p	SCAMP5	1	1	1	3
hsa-mir-218-5p	ZFYVE26	1	1	1	3
hsa-mir-218-5p	IPO8	1	1	1	3
hsa-mir-218-5p	RARA	1	1	1	3
hsa-mir-155-5p	WBP1L	1	1	1	3
hsa-mir-15b-5p	ZNF622	1	1	1	3
hsa-mir-20a-5p	FOXJ2	1	1	1	3
hsa-mir-155-5p	DPY19L1	1	1	1	3
hsa-mir-20a-5p	TMEM127	1	1	1	3
hsa-mir-20a-5p	CLOCK	1	1	1	3
hsa-mir-155-5p	CEBPB	1	1	1	3
hsa-mir-15b-5p	PAFAH1B1	1	1	1	3
hsa-mir-218-5p	BIRC5	1	1	1	3
hsa-mir-15b-5p	CD2AP	1	1	1	3
hsa-mir-155-5p	TAF5L	1	1	1	3
hsa-mir-15b-5p	BCL7A	1	1	1	3
hsa-mir-423-5p	PLCB1	1	1	1	3
hsa-mir-20a-5p	GNS	1	1	1	3
hsa-mir-3173-3p	IGF2	1	1	1	3
hsa-mir-15b-5p	CDC42SE2	1	1	1	3
hsa-mir-218-5p	KIAA1549	1	1	1	3
hsa-mir-155-5p	CBL	1	1	1	3
hsa-mir-155-5p	TRPS1	1	1	1	3
hsa-mir-15b-5p	ZMAT3	1	1	1	3
hsa-mir-155-5p	MORC3	1	1	1	3
hsa-mir-20a-5p	BBX	1	1	1	3
hsa-mir-155-5p	ZNF652	1	1	1	3
hsa-mir-3173-3p	SET	1	1	1	3
hsa-mir-15b-5p	PLAG1	1	1	1	3
hsa-mir-15b-5p	AVL9	1	1	1	3
hsa-mir-20a-5p	CEP97	1	1	1	3
hsa-mir-15b-5p	MLLT6	1	1	1	3
hsa-mir-20a-5p	CEP57	1	1	1	3
hsa-mir-423-5p	MYBL2	1	1	1	3
hsa-mir-20a-5p	USP3	1	1	1	3

hsa-mir-15b-5p	RUNX1T1	1	1	1	3
hsa-mir-4646-3p	NUFIP2	1	1	1	3
hsa-mir-218-5p	GNAS	1	1	1	3
hsa-mir-15b-5p	ZNF275	1	1	1	3
hsa-mir-423-5p	LMNB2	1	1	1	3
hsa-mir-218-5p	MFAP3	1	1	1	3
hsa-mir-423-5p	SPATA2	1	1	1	3
hsa-mir-218-5p	LRIG1	1	1	1	3
hsa-mir-218-5p	MBNL2	1	1	1	3
hsa-mir-20a-5p	LYSMD3	1	1	1	3
hsa-mir-15b-5p	MTMR4	1	1	1	3
hsa-mir-20a-5p	ZNF800	1	1	1	3
hsa-mir-20a-5p	RAB22A	1	1	1	3
hsa-mir-20a-5p	DDX5	1	1	1	3
hsa-mir-15b-5p	SIRT4	1	1	1	3
hsa-mir-15b-5p	PRKAR2A	1	1	1	3
hsa-mir-20a-5p	PLXNA1	1	1	1	3
hsa-mir-20a-5p	SESN3	1	1	1	3
hsa-mir-15b-5p	ABL2	1	1	1	3
hsa-mir-423-5p	PA2G4	1	1	1	3
hsa-mir-15b-5p	PTPRD	1	1	1	3
hsa-mir-20a-5p	NPAT	1	1	1	3
hsa-mir-155-5p	TSHZ3	1	1	1	3
hsa-mir-218-5p	NACC1	1	1	1	3
hsa-mir-3591-3p	TRIM44	1	1	1	3
hsa-mir-218-5p	PPP1CC	1	1	1	3
hsa-mir-15b-5p	TGFBR3	1	1	1	3
hsa-mir-423-5p	PAX2	1	1	1	3
hsa-mir-15b-5p	RARB	1	1	1	3
hsa-mir-20a-5p	ANKRD52	1	1	1	3
hsa-mir-20a-5p	BMPR2	1	1	1	3
hsa-mir-20a-5p	KLF3	1	1	1	3
hsa-mir-423-5p	C20orf27	1	1	1	3
hsa-mir-15b-5p	RASEF	1	1	1	3
hsa-mir-15b-5p	ZNF449	1	1	1	3
hsa-mir-155-5p	C3orf18	1	1	1	3
hsa-mir-15b-5p	LRRFIP2	1	1	1	3
hsa-mir-218-5p	UBE2H	1	1	1	3
hsa-mir-20a-5p	ARHGAP12	1	1	1	3
hsa-mir-20a-5p	LYPD6	1	1	1	3
hsa-mir-15b-5p	AKT3	1	1	1	3
hsa-mir-155-5p	INTS6	1	1	1	3
hsa-mir-155-5p	NARS	1	1	1	3
hsa-mir-155-5p	RAPGEF2	1	1	1	3

hsa-mir-15b-5p	PIM1	1	1	1	3
hsa-mir-155-5p	CSNK1G2	1	1	1	3
hsa-mir-423-5p	SPRY4	1	1	1	3
hsa-mir-155-5p	ZBTB38	1	1	1	3
hsa-mir-155-5p	LRRC59	1	1	1	3
hsa-mir-20a-5p	DUSP2	1	1	1	3
hsa-mir-3199	TRPC4AP	1	1	1	3
hsa-mir-20a-5p	NAGK	1	1	1	3
hsa-mir-20a-5p	CNOT6L	1	1	1	3
hsa-mir-218-5p	RUNX2	1	1	1	3
hsa-mir-155-5p	RAB11FIP2	1	1	1	3
hsa-mir-218-5p	SHMT1	1	1	1	3
hsa-mir-20a-5p	RBL1	1	1	1	3
hsa-mir-4712-3p	RNF111	1	1	1	3
hsa-mir-3173-3p	HMGXB3	1	1	1	3
hsa-mir-218-5p	USP34	1	1	1	3
hsa-mir-15b-5p	TBPL1	1	1	1	3
hsa-mir-20a-5p	ARHGAP1	1	1	1	3
hsa-mir-15b-5p	JARID2	1	1	1	3
hsa-mir-218-5p	RASEF	1	1	1	3
hsa-mir-20a-5p	USP32	1	1	1	3
hsa-mir-20a-5p	TNFAIP1	1	1	1	3
hsa-mir-218-5p	TPD52	1	1	1	3
hsa-mir-20a-5p	ANKH	1	1	1	3
hsa-mir-20a-5p	RORA	1	1	1	3
hsa-mir-15b-5p	LAMC1	1	1	1	3
hsa-mir-218-5p	COPS3	1	1	1	3
hsa-mir-15b-5p	GRAMD3	1	1	1	3
hsa-mir-20a-5p	PRR14L	1	1	1	3
hsa-mir-20a-5p	CFL2	1	1	1	3
hsa-mir-20a-5p	SGTB	1	1	1	3
hsa-mir-3591-3p	LCLAT1	1	1	1	3
hsa-mir-155-5p	WEE1	1	1	1	3
hsa-mir-15b-5p	VPS4A	1	1	1	3
hsa-mir-20a-5p	SMOC1	1	1	1	3
hsa-mir-218-5p	PHIP	1	1	1	3
hsa-mir-423-5p	KCTD15	1	1	1	3
hsa-mir-218-5p	SNX4	1	1	1	3
hsa-mir-20a-5p	CDKN1A	1	1	1	3
hsa-mir-15b-5p	CCND1	1	1	1	3
hsa-mir-20a-5p	FAM126B	1	1	1	3
hsa-mir-15b-5p	RECK	1	1	1	3
hsa-mir-15b-5p	SYNRG	1	1	1	3

hsa-mir-15b-5p	MOB4	1	1	1	3
hsa-mir-20a-5p	MTMR3	1	1	1	3
hsa-mir-3173-3p	NUFIP2	1	1	1	3
hsa-mir-20a-5p	SMAD5	1	1	1	3
hsa-mir-15b-5p	FGF2	1	1	1	3
hsa-mir-423-5p	SYNGR2	1	1	1	3
hsa-mir-15b-5p	PLRG1	1	1	1	3
hsa-mir-20a-5p	FRMD6	1	1	1	3
hsa-mir-20a-5p	CCDC71L	1	1	1	3
hsa-mir-20a-5p	FEM1C	1	1	1	3
hsa-mir-155-5p	IRF2BP2	1	1	1	3
hsa-mir-15b-5p	GALNT1	1	1	1	3
hsa-mir-20a-5p	OSTM1	1	1	1	3
hsa-mir-20a-5p	MAP3K2	1	1	1	3
hsa-mir-218-5p	LASP1	1	1	1	3
hsa-mir-20a-5p	NACC2	1	1	1	3
hsa-mir-15b-5p	TLE4	1	1	1	3
hsa-mir-423-5p	HNRNPUL1	1	1	1	3
hsa-mir-20a-5p	TMEM123	1	1	1	3
hsa-mir-20a-5p	CAPRIN2	1	1	1	3
hsa-mir-155-5p	ARL6IP5	1	1	1	3
hsa-mir-20a-5p	RLIM	1	1	1	3
hsa-mir-20a-5p	ZFYVE9	1	1	1	3
hsa-mir-15b-5p	USP31	1	1	1	3
hsa-mir-15b-5p	EZH1	1	1	1	3
hsa-mir-218-5p	MARCKS	1	1	1	3
hsa-mir-20a-5p	NR2C2	1	1	1	3
hsa-mir-4646-3p	VLDLR	1	1	1	3
hsa-mir-155-5p	EGFR	1	1	1	3
hsa-mir-155-5p	MEIS1	1	1	1	3
hsa-mir-15b-5p	CBX4	1	1	1	3
hsa-mir-20a-5p	MINK1	1	1	1	3
hsa-mir-423-5p	SOX12	1	1	1	3
hsa-mir-423-5p	STRIP2	1	1	1	3
hsa-mir-15b-5p	HOXA3	1	1	1	3
hsa-mir-15b-5p	SREK1	1	1	1	3
hsa-mir-218-5p	LARP4B	1	1	1	3
hsa-mir-20a-5p	TNFRSF21	1	1	1	3
hsa-mir-20a-5p	ATG14	1	1	1	3
hsa-mir-15b-5p	E2F7	1	1	1	3
hsa-mir-15b-5p	TMEM161B	1	1	1	3
hsa-mir-20a-5p	FAM210A	1	1	1	3
hsa-mir-218-5p	FBN2	1	1	1	3
hsa-mir-15b-5p	SPTLC1	1	1	1	3

hsa-mir-3173-3p	ARID1A	1	1	1	3
hsa-mir-20a-5p	UBE2Q2	1	1	1	3
hsa-mir-15b-5p	RAP2C	1	1	1	3
hsa-mir-423-5p	ASPH	1	1	1	3
hsa-mir-3173-3p	KLHDC3	1	1	1	3
hsa-mir-218-5p	YWHAB	1	1	1	3
hsa-mir-20a-5p	LPGAT1	1	1	1	3
hsa-mir-4762-5p	BTBD3	1	1	1	3
hsa-mir-15b-5p	AMOTL1	1	1	1	3
hsa-mir-20a-5p	HMGB3	1	1	1	3
hsa-mir-20a-5p	MIDN	1	1	1	3
hsa-mir-20a-5p	NUP35	1	1	1	3
hsa-mir-20a-5p	PFKP	1	1	1	3
hsa-mir-3173-3p	SSPN	1	1	1	3
hsa-mir-218-5p	SETBP1	1	1	1	3
hsa-mir-20a-5p	FJX1	1	1	1	3
hsa-mir-20a-5p	FOXJ3	1	1	1	3
hsa-mir-20a-5p	CENPQ	1	1	1	3
hsa-mir-423-5p	NSD1	1	1	1	3
hsa-mir-155-5p	LCORL	1	1	1	3
hsa-mir-423-5p	PDPK1	1	1	1	3
hsa-mir-15b-5p	KIF23	1	1	1	3
hsa-mir-20a-5p	PKD1	1	1	1	3
hsa-mir-20a-5p	U2SURP	1	1	1	3
hsa-mir-15b-5p	GPATCH8	1	1	1	3
hsa-mir-15b-5p	SMURF1	1	1	1	3
hsa-mir-20a-5p	HAS2	1	1	1	3
hsa-mir-15b-5p	CDCA4	1	1	1	3
hsa-mir-15b-5p	PPM1D	1	1	1	3
hsa-mir-20a-5p	NCOA3	1	1	1	3
hsa-mir-20a-5p	CADM2	1	1	1	3
hsa-mir-155-5p	SWSAP1	1	1	1	3
hsa-mir-20a-5p	ZBTB7A	1	1	1	3
hsa-mir-15b-5p	PHKA1	1	1	1	3
hsa-mir-15b-5p	PTPRJ	1	1	1	3
hsa-mir-20a-5p	BRMS1L	1	1	1	3
hsa-mir-155-5p	FAM91A1	1	1	1	3
hsa-mir-20a-5p	WAC	1	1	1	3
hsa-mir-877-3p	EIF4A3	1	1	1	3
hsa-mir-218-5p	ZNF609	1	1	1	3
hsa-mir-20a-5p	CLIP4	1	1	1	3
hsa-mir-20a-5p	VPS13C	1	1	1	3
hsa-mir-20a-5p	REST	1	1	1	3
hsa-mir-155-5p	RCN2	1	1	1	3

hsa-mir-15b-5p	ENTPD7	1	1	1	3
hsa-mir-20a-5p	BICD2	1	1	1	3
hsa-mir-20a-5p	PPP1R3B	1	1	1	3
hsa-mir-20a-5p	PHF6	1	1	1	3
hsa-mir-15b-5p	NUP50	1	1	1	3
hsa-mir-20a-5p	KIAA0922	1	1	1	3
hsa-mir-20a-5p	UBXN2A	1	1	1	3
hsa-mir-20a-5p	PLEKHO2	1	1	1	3
hsa-mir-15b-5p	RAD23B	1	1	1	3
hsa-mir-20a-5p	FBXO31	1	1	1	3
hsa-mir-20a-5p	MAP3K5	1	1	1	3
hsa-mir-15b-5p	SIDT2	1	1	1	3
hsa-mir-20a-5p	RBBP7	1	1	1	3
hsa-mir-20a-5p	ANKRD33B	1	1	1	3
hsa-mir-4762-5p	KLF10	1	1	1	3
hsa-mir-15b-5p	SLC39A9	1	1	1	3
hsa-mir-20a-5p	MCC	1	1	1	3
hsa-mir-20a-5p	CHIC1	1	1	1	3
hsa-mir-15b-5p	CRKL	1	1	1	3
hsa-mir-15b-5p	VEGFA	1	1	1	3
hsa-mir-218-5p	OLIG2	1	1	1	3
hsa-mir-20a-5p	MAP3K3	1	1	1	3
hsa-mir-20a-5p	RRAGD	1	1	1	3
hsa-mir-20a-5p	TADA2B	1	1	1	3
hsa-mir-20a-5p	SPOPL	1	1	1	3
hsa-mir-15b-5p	SPRED1	1	1	1	3
hsa-mir-20a-5p	EIF4H	1	1	1	3
hsa-mir-20a-5p	RUFY2	1	1	1	3
hsa-mir-20a-5p	STX6	1	1	1	3
hsa-mir-20a-5p	PPP6R3	1	1	1	3
hsa-mir-20a-5p	ARAP2	1	1	1	3
hsa-mir-20a-5p	ABHD2	1	1	1	3
hsa-mir-15b-5p	SOWAHC	1	1	1	3
hsa-mir-15b-5p	CHEK1	1	1	1	3
hsa-mir-15b-5p	ZCCHC3	1	1	1	3
hsa-mir-155-5p	TLE4	1	1	1	3
hsa-mir-15b-5p	FRYL	1	1	1	3
hsa-mir-4646-3p	NR3C1	1	1	1	3
hsa-mir-20a-5p	PTPN4	1	1	1	3
hsa-mir-15b-5p	PRRC2C	1	1	1	3
hsa-mir-3173-3p	MECP2	1	1	1	3
hsa-mir-15b-5p	MKX	1	1	1	3
hsa-mir-20a-5p	ARHGAP35	1	1	1	3
hsa-mir-20a-5p	SHOC2	1	1	1	3

hsa-mir-155-5p	RAB5C	1	1	1	3
hsa-mir-20a-5p	ELK4	1	1	1	3
hsa-mir-20a-5p	PURA	1	1	1	3
hsa-mir-15b-5p	TAOK1	1	1	1	3
hsa-mir-155-5p	PALD1	1	1	1	3
hsa-mir-15b-5p	CAPZA2	1	1	1	3
hsa-mir-155-5p	TP53INP1	1	1	1	3
hsa-mir-155-5p	MYBL1	1	1	1	3
hsa-mir-20a-5p	TNKS2	1	1	1	3
hsa-mir-155-5p	SEPT11	1	1	1	3
hsa-mir-155-5p	RAPH1	1	1	1	3
hsa-mir-15b-5p	ZBTB10	1	1	1	3
hsa-mir-15b-5p	SIK1	1	1	1	3
hsa-mir-15b-5p	SMAD7	1	1	1	3
hsa-mir-20a-5p	ATAD2	1	1	1	3
hsa-mir-155-5p	ZNF98	1	1	1	3
hsa-mir-20a-5p	SOX4	1	1	1	3
hsa-mir-15b-5p	WEE1	1	1	1	3
hsa-mir-3173-3p	SDK1	1	1	1	3
hsa-mir-20a-5p	CCSER2	1	1	1	3
hsa-mir-4762-5p	MYC	1	1	1	3
hsa-mir-15b-5p	TBL1XR1	1	1	1	3
hsa-mir-20a-5p	ZBTB9	1	1	1	3
hsa-mir-423-5p	WFIKKN2	1	1	1	3
hsa-mir-15b-5p	FKBP1A	1	1	1	3
hsa-mir-15b-5p	PAFAH1B2	1	1	1	3
hsa-mir-218-5p	RNF38	1	1	1	3
hsa-mir-15b-5p	YWHAH	1	1	1	3
hsa-mir-20a-5p	ZBTB4	1	1	1	3
hsa-mir-15b-5p	ATG9A	1	1	1	3
hsa-mir-218-5p	ZFX	1	1	1	3
hsa-mir-20a-5p	TWF1	1	1	1	3
hsa-mir-155-5p	MYO10	1	1	1	3
hsa-mir-218-5p	HECTD2	1	1	1	3
hsa-mir-20a-5p	PTPDC1	1	1	1	3
hsa-mir-15b-5p	LITAF	1	1	1	3
hsa-mir-15b-5p	ARIH1	1	1	1	3
hsa-mir-423-5p	SRM	1	1	1	3
hsa-mir-155-5p	RNF123	1	1	1	3
hsa-mir-877-3p	TP53INP2	1	1	1	3
hsa-mir-423-5p	NAV1	1	1	1	3
hsa-mir-20a-5p	ATG16L1	1	1	1	3
hsa-mir-218-5p	GPM6A	1	1	1	3
hsa-mir-15b-5p	E2F3	1	1	1	3

hsa-mir-423-5p	ARHGDIA	1	1	1	3
hsa-mir-155-5p	ARL5B	1	1	1	3
hsa-mir-20a-5p	C7orf43	1	1	1	3
hsa-mir-20a-5p	RAB10	1	1	1	3
hsa-mir-20a-5p	MCL1	1	1	1	3
hsa-mir-423-5p	MUL1	1	1	1	3
hsa-mir-20a-5p	USP28	1	1	1	3
hsa-mir-15b-5p	UBE2Q1	1	1	1	3
hsa-mir-20a-5p	CEP120	1	1	1	3
hsa-mir-423-5p	CLCN7	1	1	1	3
hsa-mir-155-5p	WWC1	1	1	1	3
hsa-mir-15b-5p	CAMSAP1	1	1	1	3
hsa-mir-155-5p	TAPT1	1	1	1	3
hsa-mir-155-5p	KBTBD2	1	1	1	3
hsa-mir-423-5p	URM1	1	1	1	3
hsa-mir-15b-5p	PDIK1L	1	1	1	3
hsa-mir-155-5p	HBP1	1	1	1	3
hsa-mir-218-5p	MTPAP	1	1	1	3
hsa-mir-4762-5p	PLCB2	1	1	1	3
hsa-mir-423-5p	ATP6V1E1	1	1	1	3
hsa-mir-423-5p	SLC6A6	1	1	1	3
hsa-mir-1273a	SLC35A3	1	1	1	3
hsa-mir-20a-5p	POLQ	1	1	1	3
hsa-mir-15b-5p	SUPT16H	1	1	1	3
hsa-mir-423-5p	ABL1	1	1	1	3
hsa-mir-20a-5p	HMBOX1	1	1	1	3
hsa-mir-20a-5p	SEPT2	1	1	1	3
hsa-mir-155-5p	BTBD1	1	1	1	3
hsa-mir-877-3p	RBMS3	1	1	1	3
hsa-mir-15b-5p	IPPK	1	1	1	3
hsa-mir-218-5p	FAM172A	1	1	1	3
hsa-mir-20a-5p	RUNDC1	1	1	1	3
hsa-mir-218-5p	STAM2	1	1	1	3
hsa-mir-877-3p	PTGFRN	1	1	1	3
hsa-mir-20a-5p	DDHD1	1	1	1	3
hsa-mir-877-3p	NEK9	1	1	1	3
hsa-mir-15b-5p	SKI	1	1	1	3
hsa-mir-15b-5p	RBPJ	1	1	1	3
hsa-mir-15b-5p	AMMECR1L	1	1	1	3
hsa-mir-20a-5p	SLK	1	1	1	3
hsa-mir-20a-5p	ZC3H12C	1	1	1	3
hsa-mir-15b-5p	CEP55	1	1	1	3
hsa-mir-15b-5p	LURAP1L	1	1	1	3
hsa-mir-15b-5p	YWHAQ	1	1	1	3

hsa-mir-20a-5p	FCHO2	1	1	1	3
hsa-mir-218-5p	CCDC6	1	1	1	3
hsa-mir-15b-5p	PNPLA6	1	1	1	3
hsa-mir-155-5p	DNAJB1	1	1	1	3
hsa-mir-15b-5p	LSM11	1	1	1	3
hsa-mir-15b-5p	N4BP1	1	1	1	3
hsa-mir-423-5p	RNF165	1	1	1	3
hsa-mir-218-5p	MAFG	1	1	1	3
hsa-mir-155-5p	PKN2	1	1	1	3
hsa-mir-20a-5p	GID4	1	1	1	3
hsa-mir-20a-5p	MKNK2	1	1	1	3
hsa-mir-20a-5p	MAPK1	1	1	1	3
hsa-mir-155-5p	GNAS	1	1	1	3
hsa-mir-218-5p	GFPT1	1	1	1	3
hsa-mir-20a-5p	ABCA1	1	1	1	3
hsa-mir-218-5p	PDGFRA	1	1	1	3
hsa-mir-15b-5p	OGT	1	1	1	3
hsa-mir-15b-5p	KATNAL1	1	1	1	3
hsa-mir-15b-5p	CDC25A	1	1	1	3
hsa-mir-20a-5p	ULK1	1	1	1	3
hsa-mir-20a-5p	FOXQ1	1	1	1	3
hsa-mir-3173-3p	BTG2	1	1	1	3
hsa-mir-218-5p	VAT1	1	1	1	3
hsa-mir-20a-5p	CREB1	1	1	1	3
hsa-mir-218-5p	AHI1	1	1	1	3
hsa-mir-20a-5p	CTSA	1	1	1	3
hsa-mir-20a-5p	E2F2	1	1	1	3
hsa-mir-15b-5p	ITGA2	1	1	1	3
hsa-mir-218-5p	GHITM	1	1	1	3
hsa-mir-15b-5p	FOXK1	1	1	1	3
hsa-mir-15b-5p	PSAT1	1	1	1	3
hsa-mir-15b-5p	DDX3X	1	1	1	3
hsa-mir-20a-5p	GOLGA1	1	1	1	3
hsa-mir-20a-5p	UXS1	1	1	1	3
hsa-mir-218-5p	TTC33	1	1	1	3
hsa-mir-20a-5p	PLS1	1	1	1	3
hsa-mir-15b-5p	STX17	1	1	1	3
hsa-mir-20a-5p	SCAMP2	1	1	1	3
hsa-mir-20a-5p	KLHL28	1	1	1	3
hsa-mir-15b-5p	STXBP1	1	1	1	3
hsa-mir-155-5p	MASTL	1	1	1	3
hsa-mir-218-5p	PPP2R2A	1	1	1	3
hsa-mir-15b-5p	SYNJ1	1	1	1	3
hsa-mir-155-5p	CSF1R	1	1	1	3

hsa-mir-20a-5p	CHAF1A	1	1	1	3
hsa-mir-155-5p	BACH1	1	1	1	3
hsa-mir-20a-5p	HAUS8	1	1	1	3
hsa-mir-877-3p	UBXN7	1	1	1	3
hsa-mir-155-5p	ZNF260	1	1	1	3
hsa-mir-155-5p	JARID2	1	1	1	3
hsa-mir-423-5p	MKNK2	1	1	1	3
hsa-mir-15b-5p	NUP210	1	1	1	3
hsa-mir-3173-3p	EIF4EBP1	1	1	1	3
hsa-mir-155-5p	CD36	1	1	1	3
hsa-mir-15b-5p	RAB11FIP2	1	1	1	3
hsa-mir-15b-5p	SBNO1	1	1	1	3
hsa-mir-15b-5p	CDC37L1	1	1	1	3
hsa-mir-4712-3p	BASP1	1	1	1	3
hsa-mir-15b-5p	STK38	1	1	1	3
hsa-mir-155-5p	KRAS	1	1	1	3
hsa-mir-20a-5p	ATL3	1	1	1	3
hsa-mir-218-5p	DDX58	1	1	1	3
hsa-mir-4762-5p	DEK	1	1	1	3
hsa-mir-20a-5p	EEA1	1	1	1	3
hsa-mir-218-5p	SFRP2	1	1	1	3
hsa-mir-20a-5p	DENND5B	1	1	1	3
hsa-mir-20a-5p	PPP6C	1	1	1	3
hsa-mir-20a-5p	ZBTB18	1	1	1	3
hsa-mir-20a-5p	PIP4K2A	1	1	1	3
hsa-mir-423-5p	MICALL1	1	1	1	3
hsa-mir-15b-5p	CRIM1	1	1	1	3
hsa-mir-218-5p	SACS	1	1	1	3
hsa-mir-15b-5p	DDX3Y	1	1	1	3
hsa-mir-20a-5p	DPYSL2	1	1	1	3
hsa-mir-423-5p	RPS6KA4	1	1	1	3
hsa-mir-218-5p	FBXO41	1	1	1	3
hsa-mir-20a-5p	CRY2	1	1	1	3
hsa-mir-423-5p	CTDNEP1	1	1	1	3
hsa-mir-20a-5p	EFCAB14	1	1	1	3
hsa-mir-15b-5p	RPS6KA3	1	1	1	3
hsa-mir-155-5p	HIVEP2	1	1	1	3
hsa-mir-218-5p	ROBO1	1	1	1	3
hsa-mir-20a-5p	HIF1A	1	1	1	3
hsa-mir-15b-5p	FASN	1	1	1	3
hsa-mir-155-5p	GPM6B	1	1	1	3
hsa-mir-3591-3p	ST13	1	1	1	3
hsa-mir-15b-5p	NUFIP2	1	1	1	3

hsa-mir-20a-5p	FBXO21	1	1	1	3
hsa-mir-15b-5p	NAPG	1	1	1	3
hsa-mir-20a-5p	TRIM37	1	1	1	3
hsa-mir-15b-5p	CCNE1	1	1	1	3
hsa-mir-20a-5p	TMEM245	1	1	1	3
hsa-mir-15b-5p	SEC24A	1	1	1	3
hsa-mir-15b-5p	LRIG2	1	1	1	3
hsa-mir-20a-5p	C14orf28	1	1	1	3
hsa-mir-3173-3p	CDC42SE1	1	1	1	3
hsa-mir-20a-5p	ANKRD50	1	1	1	3
hsa-mir-155-5p	SLC11A2	1	1	1	3
hsa-mir-15b-5p	PPM1A	1	1	1	3
hsa-mir-423-5p	SQSTM1	1	1	1	3
hsa-mir-20a-5p	EZH1	1	1	1	3
hsa-mir-20a-5p	FAM46C	1	1	1	3
hsa-mir-155-5p	TADA2B	1	1	1	3
hsa-mir-20a-5p	TXLNA	1	1	1	3
hsa-mir-20a-5p	RUNX3	1	1	1	3
hsa-mir-20a-5p	BTG3	1	1	1	3
hsa-mir-20a-5p	FAM129A	1	1	1	3
hsa-mir-15b-5p	TMEM135	1	1	1	3
hsa-mir-20a-5p	PHTF2	1	1	1	3
hsa-mir-15b-5p	ETNK1	1	1	1	3
hsa-mir-15b-5p	NR2C2	1	1	1	3
hsa-mir-15b-5p	MYO5A	1	1	1	3
hsa-mir-20a-5p	F3	1	1	1	3
hsa-mir-218-5p	DOCK9	1	1	1	3
hsa-mir-155-5p	SMAD2	1	1	1	3
hsa-mir-15b-5p	ARHGAP32	1	1	1	3
hsa-mir-20a-5p	CIT	1	1	1	3
hsa-mir-20a-5p	LASP1	1	1	1	3
hsa-mir-20a-5p	TET3	1	1	1	3
hsa-mir-20a-5p	DYNC1LI2	1	1	1	3
hsa-mir-218-5p	KIAA1161	1	1	1	3
hsa-mir-20a-5p	ENPP5	1	1	1	3
hsa-mir-20a-5p	CCND1	1	1	1	3
hsa-mir-3173-3p	B4GALT1	1	1	1	3
hsa-mir-218-5p	CDK6	1	1	1	3
hsa-mir-218-5p	FAM217B	1	1	1	3
hsa-mir-20a-5p	DNAJB9	1	1	1	3
hsa-mir-155-5p	ZNF492	1	1	1	3
hsa-mir-218-5p	MBNL1	1	1	1	3
hsa-mir-15b-5p	TRAM1	1	1	1	3
hsa-mir-20a-5p	CERCAM	1	1	1	3

hsa-mir-155-5p	PDLIM5	1	1	1	3
hsa-mir-20a-5p	VPS26A	1	1	1	3
hsa-mir-15b-5p	ZBTB34	1	1	1	3
hsa-mir-155-5p	PAXBP1	1	1	1	3
hsa-mir-15b-5p	CACUL1	1	1	1	3
hsa-mir-15b-5p	MTMR3	1	1	1	3
hsa-mir-20a-5p	TP53INP1	1	1	1	3
hsa-mir-877-3p	EIF2B2	1	1	1	3
hsa-mir-20a-5p	LAPTM4A	1	1	1	3
hsa-mir-155-5p	FAR1	1	1	1	3
hsa-mir-15b-5p	AMER1	1	1	1	3
hsa-mir-218-5p	BIRC6	1	1	1	3
hsa-mir-3173-3p	FAM168A	1	1	1	3
hsa-mir-218-5p	SLC45A3	1	1	1	3
hsa-mir-15b-5p	RPRD2	1	1	1	3
hsa-mir-15b-5p	NOTCH2	1	1	1	3
hsa-mir-20a-5p	REEP3	1	1	1	3
hsa-mir-423-5p	LASP1	1	1	1	3
hsa-mir-20a-5p	KMT2B	1	1	1	3
hsa-mir-218-5p	RET	1	1	1	3
hsa-mir-218-5p	PTPRK	1	1	1	3
hsa-mir-155-5p	ERMP1	1	1	1	3
hsa-mir-20a-5p	E2F1	1	1	1	3
hsa-mir-20a-5p	KIF23	1	1	1	3
hsa-mir-3173-3p	DDX11	1	1	1	3
hsa-mir-155-5p	CREBRF	1	1	1	3
hsa-mir-155-5p	MPP5	1	1	1	3
hsa-mir-155-5p	IKBIP	1	1	1	3
hsa-mir-15b-5p	LUZP1	1	1	1	3
hsa-mir-155-5p	TWF1	1	1	1	3
hsa-mir-155-5p	TOMM20	1	1	1	3
hsa-mir-20a-5p	NKIRAS1	1	1	1	3
hsa-mir-144-5p	SYNPO2	1	1	1	3
hsa-mir-15b-5p	DMTF1	1	1	1	3
hsa-mir-20a-5p	KIAA1147	1	1	1	3
hsa-mir-15b-5p	PLSCR4	1	1	1	3
hsa-mir-15b-5p	PISD	1	1	1	3
hsa-mir-20a-5p	BTBD7	1	1	1	3
hsa-mir-20a-5p	TRIP10	1	1	1	3
hsa-mir-20a-5p	PITPNA	1	1	1	3
hsa-mir-20a-5p	RPA2	1	1	1	3
hsa-mir-20a-5p	ANKIB1	1	1	1	3
hsa-mir-218-5p	PTP4A1	1	1	1	3
hsa-mir-20a-5p	JAK1	1	1	1	3

hsa-mir-20a-5p	NRBP1	1	1	1	3
hsa-mir-15b-5p	ATG14	1	1	1	3
hsa-mir-20a-5p	TSG101	1	1	1	3
hsa-mir-20a-5p	RAPGEF4	1	1	1	3
hsa-mir-20a-5p	SQSTM1	1	1	1	3
hsa-mir-20a-5p	OCRL	1	1	1	3
hsa-mir-15b-5p	STRADB	1	1	1	3
hsa-mir-20a-5p	RAP2C	1	1	1	3
hsa-mir-15b-5p	PHLPP2	1	1	1	3
hsa-mir-20a-5p	HBP1	1	1	1	3
hsa-mir-20a-5p	STAT3	1	1	1	3
hsa-mir-15b-5p	ZNRF2	1	1	1	3
hsa-mir-155-5p	ETS1	1	1	1	3
hsa-mir-15b-5p	CYP26B1	1	1	1	3
hsa-mir-20a-5p	NFAT5	1	1	1	3
hsa-mir-20a-5p	PARD6B	1	1	1	3
hsa-mir-15b-5p	PNISR	1	1	1	3
hsa-mir-3199	PCSK2	1	1	1	3
hsa-mir-877-3p	PIP4K2B	1	1	1	3
hsa-mir-20a-5p	STC1	1	1	1	3
hsa-mir-15b-5p	HSPE1-MOB4	1	1	1	3
hsa-mir-20a-5p	PKNOX1	1	1	1	3
hsa-mir-20a-5p	PAFAH1B1	1	1	1	3
hsa-mir-15b-5p	SLC9A6	1	1	1	3
hsa-mir-20a-5p	UEVLD	1	1	1	3
hsa-mir-15b-5p	RS1	1	1	1	3
hsa-mir-877-3p	WDR43	1	1	1	3
hsa-mir-15b-5p	MTFR1L	1	1	1	3
hsa-mir-15b-5p	ZNRF3	1	1	1	3
hsa-mir-155-5p	ITK	1	1	1	3
hsa-mir-20a-5p	CYBRD1	1	1	1	3
hsa-mir-15b-5p	PDIA6	1	1	1	3
hsa-mir-3199	SET	1	1	1	3
hsa-mir-218-5p	PRKG1	1	1	1	3
hsa-mir-15b-5p	FAM122B	1	1	1	3
hsa-mir-15b-5p	AFF4	1	1	1	3
hsa-mir-15b-5p	ASH1L	1	1	1	3
hsa-mir-20a-5p	CHD9	1	1	1	3
hsa-mir-15b-5p	YIPF6	1	1	1	3
hsa-mir-218-5p	ARPP19	1	1	1	3
hsa-mir-15b-5p	USP3	1	1	1	3
hsa-mir-20a-5p	TXNIP	1	1	1	3
hsa-mir-218-5p	VOPP1	1	1	1	3

hsa-mir-155-5p	DHX40	1	1	1	3
hsa-mir-20a-5p	MASTL	1	1	1	3
hsa-mir-20a-5p	MAP3K12	1	1	1	3
hsa-mir-20a-5p	RGMB	1	1	1	3
hsa-mir-20a-5p	ZFYVE26	1	1	1	3
hsa-mir-155-5p	NKX3-1	1	1	1	3
hsa-mir-15b-5p	PCMT1	1	1	1	3
hsa-mir-20a-5p	SLC22A23	1	1	1	3
hsa-mir-3173-3p	YWHAE	1	1	1	3
hsa-mir-20a-5p	FRS2	1	1	1	3
hsa-mir-155-5p	IL17RB	1	1	1	3
hsa-mir-218-5p	SUMF2	1	1	1	3
hsa-mir-20a-5p	FYCO1	1	1	1	3
hsa-mir-423-5p	UBE2L3	1	1	1	3
hsa-mir-20a-5p	RRM2	1	1	1	3
hsa-mir-15b-5p	RBBP6	1	1	1	3
hsa-mir-20a-5p	RAB5B	1	1	1	3
hsa-mir-3173-3p	EN2	1	1	1	3
hsa-mir-218-5p	KLF9	1	1	1	3
hsa-mir-877-3p	TRIM39	1	1	1	3
hsa-mir-15b-5p	TBC1D20	1	1	1	3
hsa-mir-20a-5p	PPP1R15B	1	1	1	3
hsa-mir-15b-5p	PSKH1	1	1	1	3
hsa-mir-218-5p	PKP4	1	1	1	3
hsa-mir-15b-5p	UBE4A	1	1	1	3
hsa-mir-15b-5p	CALU	1	1	1	3
hsa-mir-20a-5p	STK17B	1	1	1	3
hsa-mir-4762-5p	ZBTB34	1	1	1	3
hsa-mir-15b-5p	CSDE1	1	1	1	3
hsa-mir-155-5p	UBQLN1	1	1	1	3
hsa-mir-15b-5p	CCNT2	1	1	1	3
hsa-mir-15b-5p	USP42	1	1	1	3
hsa-mir-155-5p	TERF1	1	1	1	3
hsa-mir-218-5p	FAM198B	1	1	1	3
hsa-mir-20a-5p	EGLN3	1	1	1	3
hsa-mir-4762-5p	STAT5B	1	1	1	3
hsa-mir-877-3p	IL2RA	1	1	1	3
hsa-mir-15b-5p	SERBP1	1	1	1	3
hsa-mir-15b-5p	USP15	1	1	1	3
hsa-mir-20a-5p	WDR37	1	1	1	3
hsa-mir-20a-5p	GNB5	1	1	1	3
hsa-mir-423-5p	SLC48A1	1	1	1	3
hsa-mir-3591-3p	ELMO1	1	1	1	3
hsa-mir-155-5p	ZNF236	1	1	1	3

hsa-mir-3199	HOXB8	1	1	1	3
hsa-mir-155-5p	AIMP1	1	1	1	3
hsa-mir-15b-5p	CUL2	1	1	1	3
hsa-mir-20a-5p	ZNF202	1	1	1	3
hsa-mir-15b-5p	FBXL20	1	1	1	3
hsa-mir-20a-5p	CRK	1	1	1	3
hsa-mir-20a-5p	TMEM64	1	1	1	3
hsa-mir-155-5p	NOVA1	1	1	1	3
hsa-mir-218-5p	HP1BP3	1	1	1	3
hsa-mir-155-5p	CHAF1A	1	1	1	3
hsa-mir-218-5p	BTG2	1	1	1	3
hsa-mir-218-5p	PRLR	1	1	1	3
hsa-mir-423-5p	ABCC5	1	1	1	3
hsa-mir-20a-5p	GBF1	1	1	1	3
hsa-mir-15b-5p	ZNF704	1	1	1	3
hsa-mir-15b-5p	BZW1	1	1	1	3
hsa-mir-155-5p	SYPL1	1	1	1	3
hsa-mir-218-5p	SEMA5A	1	1	1	3
hsa-mir-20a-5p	ANKRD13C	1	1	1	3
hsa-mir-15b-5p	UBE2V1	1	1	1	3
hsa-mir-4712-3p	TRIM5	1	1	1	3
hsa-mir-155-5p	VPS36	1	1	1	3
hsa-mir-20a-5p	EGR2	1	1	1	3
hsa-mir-20a-5p	FBXO48	1	1	1	3
hsa-mir-15b-5p	CBX2	1	1	1	3
hsa-mir-20a-5p	ANKRD12	1	1	1	3
hsa-mir-423-5p	DDX54	1	1	1	3
hsa-mir-20a-5p	FAM102A	1	1	1	3
hsa-mir-20a-5p	KLF10	1	1	1	3
hsa-mir-20a-5p	ABL2	1	1	1	3
hsa-mir-20a-5p	RPS6KA5	1	1	1	3
hsa-mir-15b-5p	TNRC6B	1	1	1	3
hsa-mir-155-5p	NAMPT	1	1	1	3
hsa-mir-15b-5p	SNX16	1	1	1	3
hsa-mir-3173-3p	PLA2G2F	1	1	1	3
hsa-mir-15b-5p	TLK1	1	1	1	3
hsa-mir-155-5p	ZIC3	1	1	1	3
hsa-mir-15b-5p	PIK3R1	1	1	1	3
hsa-mir-155-5p	GABARAPL1	1	1	1	3
hsa-mir-15b-5p	BTG2	1	1	1	3
hsa-mir-20a-5p	MAPRE3	1	1	1	3
hsa-mir-20a-5p	GAB1	1	1	1	3
hsa-mir-218-5p	TTYH3	1	1	1	3

hsa-mir-20a-5p	PPP3R1	1	1	1	3
hsa-mir-20a-5p	AKTIP	1	1	1	3
hsa-mir-15b-5p	TRAK1	1	1	1	3
hsa-mir-15b-5p	ZBTB33	1	1	1	3
hsa-mir-20a-5p	SSX2IP	1	1	1	3
