# Supplementary material for: Identification and Validation of STC1 Act as a Biomarker for High-Altitude Diseases and Its Pan-Cancer Analysis
Source: Int J Mol Sci. 2024 Aug 21;25(16):9085. doi: 10.3390/ijms25169085 (PMC11354978; doi:10.3390/ijms25169085)
Supplement: Supplementary file 1 [file ijms-25-09085-s001.zip › Supplementary Table S5.pdf]

Supplementary Table S5. Immune gene list

Symbol	ID	Name	Synonyms	Chromosome	Category
AZGP1	563	alpha-2-glycoprotein 1, zinc-binding	ZA2G|ZAG	7	Antigen_Processing_and_Presentation
B2M	567	beta-2-microglobulin	IMD43	15	Antigen_Processing_and_Presentation
CALR	811	calreticulin	CRT|HEL-S-99n|RO|SSA|cC1qR	19	Antigen_Processing_and_Presentation
CANX	821	calnexin	CNX|IP90|P90	5	Antigen_Processing_and_Presentation
CD1A	909	CD1a molecule	CD1|FCB6|HTA1|R4|T6	1	Antigen_Processing_and_Presentation
CD1B	910	CD1b molecule	CD1|CD1A|R1	1	Antigen_Processing_and_Presentation
CD1C	911	CD1c molecule	BDCA1|CD1|CD1A|R7	1	Antigen_Processing_and_Presentation
CD1D	912	CD1d molecule	CD1A|R3|R3G1	1	Antigen_Processing_and_Presentation
CD1E	913	CD1e molecule	CD1A|R2	1	Antigen_Processing_and_Presentation
CD4	920	CD4 molecule	CD4mut	12	Antigen_Processing_and_Presentation
CD8A	925	CD8a molecule	CD8|Leu2|p32	2	Antigen_Processing_and_Presentation
CD8B	926	CD8b molecule	CD8B1|LEU2|LY3|LYT3|P37	2	Antigen_Processing_and_Presentation
CD74	972	CD74 molecule	DHLAG|HLADG|II|Ia-GAMMA|p33	5	Antigen_Processing_and_Presentation
CREB1	1385	cAMP responsive element binding protein 1	CREB|CREB-1	2	Antigen_Processing_and_Presentation
CTSB	1508	cathepsin B	APPS|CPSB|RECEUP	8	Antigen_Processing_and_Presentation
CTSE	1510	cathepsin E	CATE	1	Antigen_Processing_and_Presentation
CTSL	1514	cathepsin L	CATL|CTSL1|MEP	9	Antigen_Processing_and_Presentation
CTSS	1520	cathepsin S	-	1	Antigen_Processing_and_Presentation
FCER1G	2207	Fc fragment of IgE receptor Ig	FCRG	1	Antigen_Processing_and_Presentation
FCGRT	2217	Fc fragment of IgG receptor and transporter	FCRN|alpha-chain	19	Antigen_Processing_and_Presentation
PDIA3	2923	protein disulfide isomerase family A member 3	ER60|ERp57|ERp60|ERp61|GRP57|GRP58|HEL-S-269|HEL-S-93n|HsT17083|P58|PI-P	15	Antigen_Processing_and_Presentation

			LC		
HFE	3077	homeostatic iron regulator	HFE1|HH|HLA-H|MVCD7|TFQTL2	6	Antigen_Processing_and_Presentation
HLA-A	3105	major histocompatibility complex, class I, A	HLAA	6	Antigen_Processing_and_Presentation
HLA-B	3106	major histocompatibility complex, class I, B	AS|B-4901|HLAB	6	Antigen_Processing_and_Presentation
HLA-C	3107	major histocompatibility complex, class I, C	D6S204|HLA-JY3|HLAC|HLC-C|MHC|PSORS1	6	Antigen_Processing_and_Presentation
HLA-DMA	3108	major histocompatibility complex, class II, DM alpha	D6S222E|DMA|HLADM|RING6	6	Antigen_Processing_and_Presentation
HLA-DMB	3109	major histocompatibility complex, class II, DM beta	D6S221E|RING7	6	Antigen_Processing_and_Presentation
HLA-DOA	3111	major histocompatibility complex, class II, DO alpha	HLA-DNA|HLA-DZA|HLADZ	6	Antigen_Processing_and_Presentation
HLA-DOB	3112	major histocompatibility complex, class II, DO beta	DOB|HLA_DOB	6	Antigen_Processing_and_Presentation
HLA-DPA1	3113	major histocompatibility complex, class II, DP alpha 1	DP(W3)|DP(W4)|DPA1|HLA-DP1A|HLA-DPB1|HLADP|HLASB|PLT1	6	Antigen_Processing_and_Presentation
HLA-DPB1	3115	major histocompatibility complex, class II, DP beta 1	DPB1|HLA-DP|HLA-DP1B|HLA-DPB	6	Antigen_Processing_and_Presentation
HLA-DQA1	3117	major histocompatibility complex, class II, DQ alpha 1	CELIAC1|DQ-A1|DQA1|HLA-DQA	6	Antigen_Processing_and_Presentation
HLA-DQA2	3118	major histocompatibility complex, class II, DQ alpha 2	DC-alpha|DX-ALPHA|HLA-DCA|HLA-DXA|HLADQA2	6	Antigen_Processing_and_Presentation
HLA-DQB1	3119	major histocompatibility complex, class II, DQ beta 1	CELIAC1|HLA-DQB|IDDM1	6	Antigen_Processing_and_Presentation
HLA-DRA	3122	major histocompatibility complex, class II, DR alpha	HLA-DRA1	6	Antigen_Processing_and_Presentation
HLA-D	3123	major histocompatibility complex, class II, DR	DRB1|HLA-DR1B|HLA-DRB|SS1	6	Antigen_Processing_and_Presentation

RB1		beta 1			
HLA-DRB3	3125	major histocompatibility complex, class II, DR beta 3	DRB3|HLA-DPB1|HLA-DR1B|HLA-DR3B	6	Antigen_Processing_and_Presentation
HLA-DRB4	3126	major histocompatibility complex, class II, DR beta 4	DR4|DRB4|HLA-DR4B|HLA-DRB4*	6	Antigen_Processing_and_Presentation
HLA-DRB5	3127	major histocompatibility complex, class II, DR beta 5	-	6	Antigen_Processing_and_Presentation
HLA-E	3133	major histocompatibility complex, class I, E	HLA-6.2|QA1	6	Antigen_Processing_and_Presentation
HLA-F	3134	major histocompatibility complex, class I, F	CDA12|HLA-5.4|HLA-CDA12|HLAF	6	Antigen_Processing_and_Presentation
HLA-G	3135	major histocompatibility complex, class I, G	MHC-G	6	Antigen_Processing_and_Presentation
HLA-H	3136	major histocompatibility complex, class I, H (pseudogene)	HLAHP	6	Antigen_Processing_and_Presentation
MR1	3140	major histocompatibility complex, class I-related	HLALS	1	Antigen_Processing_and_Presentation
HSPA1A	3303	heat shock protein family A (Hsp70) member 1A	HEL-S-103|HSP70-1|HSP70-1A|HSP70-2|HSP70.1|HSP70.2|HSP70I|HSP72|HSPA1	6	Antigen_Processing_and_Presentation
HSPA1B	3304	heat shock protein family A (Hsp70) member 1B	HSP70-1|HSP70-1B|HSP70-2|HSP70.1|HSP70.2|HSP72|HSPA1|HSX70	6	Antigen_Processing_and_Presentation
HSPA1L	3305	heat shock protein family A (Hsp70) member 1 like	HSP70-1L|HSP70-HOM|HSP70T|hum70t	6	Antigen_Processing_and_Presentation
HSPA2	3306	heat shock protein family A (Hsp70) member 2	HSP70-2|HSP70-3	14	Antigen_Processing_and_Presentation
HSPA4	3308	heat shock protein family A (Hsp70) member 4	APG-2|HEL-S-5a|HS24/P52|HSPH2|RY|hsp70|hsp70RY	5	Antigen_Processing_and_Presentation
HSPA5	3309	heat shock protein family A (Hsp70) member 5	BIP|GRP78|HEL-S-89n|MIF2	9	Antigen_Processing_and_Presentation
HSPA6	3310	heat shock protein family A (Hsp70) member 6	HSP70B'	1	Antigen_Processing_and_Presentation
HSPA8	3312	heat shock protein family A (Hsp70) member 8	HEL-33|HEL-S-72p|HSC54|HSC70|HSC71|H	11	Antigen_Processing_and_Presentation

			SP71|HSP73|HSPA10|LAP-1|LAP1|NIP71		
HSP90AA1	3320	heat shock protein 90 alpha family class A member 1	EL52|HEL-S-65p|HSP86|HSP89A|HSP90A|HSP90N|HSPC1|HSPCA|HSPCAL1|HSPCAL4|HSPN|Hsp103|Hsp89|Hsp90|LAP-2|LAP2	14	Antigen_Processing_and_Presentation
HSP90AB1	3326	heat shock protein 90 alpha family class B member 1	D6S182|HSP84|HSP90B|HSPC2|HSPCB	6	Antigen_Processing_and_Presentation
ICAM1	3383	intercellular adhesion molecule 1	BB2|CD54|P3.58	19	Antigen_Processing_and_Presentation
IFNA1	3439	interferon alpha 1	IFL|IFN|IFN-ALPHA|IFN-alphaD|IFNA13|IFNA@|leIF D	9	Antigen_Processing_and_Presentation
IFNA2	3440	interferon alpha 2	IFN-alpha-2|IFN-alphaA|IFNA|IFNA2B|leIF A	9	Antigen_Processing_and_Presentation
IFNA4	3441	interferon alpha 4	IFN-alpha4a|INFA4	9	Antigen_Processing_and_Presentation
IFNA5	3442	interferon alpha 5	IFN-alpha-5|IFN-alphaG|INA5|INFA5|leIF G	9	Antigen_Processing_and_Presentation
IFNA6	3443	interferon alpha 6	IFN-alphaK	9	Antigen_Processing_and_Presentation
IFNA7	3444	interferon alpha 7	IFN-alphaJ|IFNA-J	9	Antigen_Processing_and_Presentation
IFNA8	3445	interferon alpha 8	IFN-alphaB	9	Antigen_Processing_and_Presentation
IFNA10	3446	interferon alpha 10	IFN-alphaC	9	Antigen_Processing_and_Presentation
IFNA13	3447	interferon alpha 13	-	9	Antigen_Processing_and_Presentation
IFNA14	3448	interferon alpha 14	IFN-alphaH|LEIF2H	9	Antigen_Processing_and_Presentation
IFNA16	3449	interferon alpha 16	IFN-alpha-16|IFN-alphaO	9	Antigen_Processing_and_Presentation
IFNA1	3451	interferon alpha 17	IFN-alphaI|IFNA|INFA|LEIF2C1	9	Antigen_Processing_and_Presentation

7					
IFNA21	3452	interferon alpha 21	IFN-alphaI|LeIF F|leIF-F	9	Antigen_Processing_and_Presentation
IFNG	3458	interferon gamma	IFG|IFI	12	Antigen_Processing_and_Presentation
KIR2DL1	3802	killer cell immunoglobulin like receptor, two Ig domains and long cytoplasmic tail 1	CD158A|KIR-K64|KIR221|KIR2DL3|NKAT|NKAT-1|NKAT1|p58.1	19	Antigen_Processing_and_Presentation
KIR2DL2	3803	killer cell immunoglobulin like receptor, two Ig domains and long cytoplasmic tail 2	CD158B1|CD158b|NKAT-6|NKAT6|p58.2	19	Antigen_Processing_and_Presentation
KIR2DL3	3804	killer cell immunoglobulin like receptor, two Ig domains and long cytoplasmic tail 3	CD158B2|CD158b|GL183|KIR-023GB|KIR-K7b|KIR-K7c|KIR2DL|KIR2DS5|KIRCL23|NKAT|NKAT2|NKAT2A|NKAT2B|p58	19	Antigen_Processing_and_Presentation
KIR2DL4	3805	killer cell immunoglobulin like receptor, two Ig domains and long cytoplasmic tail 4	CD158D|G9P|KIR-103AS|KIR-2DL4|KIR103|KIR103AS	19	Antigen_Processing_and_Presentation
KIR2DS1	3806	killer cell immunoglobulin like receptor, two Ig domains and short cytoplasmic tail 1	CD158H|CD158a|p50.1	19	Antigen_Processing_and_Presentation
KIR2DS3	3808	killer cell immunoglobulin like receptor, two Ig domains and short cytoplasmic tail 3	NKAT7	19	Antigen_Processing_and_Presentation
KIR2DS4	3809	killer cell immunoglobulin like receptor, two Ig domains and short cytoplasmic tail 4	CD158I|KIR-2DS4|KIR1D|KIR412|KKA3|NKAT-8|NKAT8	19	Antigen_Processing_and_Presentation
KIR2DS5	3810	killer cell immunoglobulin like receptor, two Ig domains and short cytoplasmic tail 5	CD158G|NKAT9	19	Antigen_Processing_and_Presentation
KIR3DL1	3811	killer cell immunoglobulin like receptor, three Ig domains and long cytoplasmic tail 1	CD158E1|KIR|KIR3DL1/S1|NKAT-3|NKAT3|NKB1|NKB1B	19	Antigen_Processing_and_Presentation
KIR3DL2	3812	killer cell immunoglobulin like receptor, three Ig domains and long cytoplasmic tail 2	3DL2|CD158K|KIR-3DL2|NKAT-4|NKAT4|NKAT4B|p140	19	Antigen_Processing_and_Presentation
KLRC1	3821	killer cell lectin like receptor C1	CD159A|NKG2|NKG2A	12	Antigen_Processing_and_Presentation

KLRC2	3822	killer cell lectin like receptor C2	CD159c|NKG2-C|NKG2C	12	Antigen_Processing_and_Presentation
KLRC3	3823	killer cell lectin like receptor C3	NKG2-E|NKG2E	12	Antigen_Processing_and_Presentation
KLRD1	3824	killer cell lectin like receptor D1	CD94	12	Antigen_Processing_and_Presentation
LTA	4049	lymphotoxin alpha	LT|TNFB|TNFSF1|TNLG1E	6	Antigen_Processing_and_Presentation
CIITA	4261	class II major histocompatibility complex transactivator	C2TA|CIITAIV|MHC2TA|NLRA	16	Antigen_Processing_and_Presentation
MICA	100507436	MHC class I polypeptide-related sequence A	MIC-A|PERB11.1	6	Antigen_Processing_and_Presentation
MICB	4277	MHC class I polypeptide-related sequence B	PERB11.2	6	Antigen_Processing_and_Presentation
NFYA	4800	nuclear transcription factor Y subunit alpha	CBF-A|CBF-B|HAP2|NF-YA	6	Antigen_Processing_and_Presentation
NFYB	4801	nuclear transcription factor Y subunit beta	CBF-A|CBF-B|HAP3|NF-YB	12	Antigen_Processing_and_Presentation
NFYC	4802	nuclear transcription factor Y subunit gamma	CBF-C|CBFC|H1TF2A|HAP5|HSM|NF-YC	1	Antigen_Processing_and_Presentation
LGMN	5641	legumain	AEP|LGMN1|PRSC1	14	Antigen_Processing_and_Presentation
PSMB8	5696	proteasome 20S subunit beta 8	ALDD|D6S216|D6S216E|JMP|LMP7|NKJO|PRAAS1|PSMB5i|RING10	6	Antigen_Processing_and_Presentation
PSMC1	5700	proteasome 26S subunit, ATPase 1	P26S4|S4|p56	14	Antigen_Processing_and_Presentation
PSMC2	5701	proteasome 26S subunit, ATPase 2	MSS1|Nbla10058|S7	7	Antigen_Processing_and_Presentation
PSMC3	5702	proteasome 26S subunit, ATPase 3	TBP1	11	Antigen_Processing_and_Presentation
PSMC4	5704	proteasome 26S subunit, ATPase 4	MIP224|RPT3|S6|TBP-7|TBP7	19	Antigen_Processing_and_Presentation
PSMC5	5705	proteasome 26S subunit, ATPase 5	S8|SUG-1|SUG1|TBP10|TRIP1|p45|p45/SUG	17	Antigen_Processing_and_Presentation
PSMC6	5706	proteasome 26S subunit, ATPase 6	SUG2|p42	14	Antigen_Processing_and_Presentation
PSMD1	5707	proteasome 26S subunit, non-ATPase 1	P112|Rpn2|S1	2	Antigen_Processing_and_Presentation
PSMD2	5708	proteasome 26S subunit, non-ATPase 2	P97|RPN1|S2|TRAP2	3	Antigen_Processing_and_Presentation
PSMD3	5709	proteasome 26S subunit, non-ATPase 3	P58|RPN3|S3|TSTA2	17	Antigen_Processing_and_Presentation

PSMD4	5710	proteasome 26S subunit, non-ATPase 4	AF|AF-1|ASF|MCB1|Rpn10|S5A|pUB-R5		1	Antigen_Processing_and_Presentation
PSMD5	5711	proteasome 26S subunit, non-ATPase 5	S5B		9	Antigen_Processing_and_Presentation
PSMD7	5713	proteasome 26S subunit, non-ATPase 7	MOV34|P40|Rpn8|S12		16	Antigen_Processing_and_Presentation
PSMD8	5714	proteasome 26S subunit, non-ATPase 8	HEL-S-91n|HIP6|HYPF|Nin1p|Rpn12|S14|p31		19	Antigen_Processing_and_Presentation
PSMD10	5716	proteasome 26S subunit, non-ATPase 10	dJ889N15.2|p28|p28(GANK)	X		Antigen_Processing_and_Presentation
PSMD11	5717	proteasome 26S subunit, non-ATPase 11	Rpn6|S9|p44.5		17	Antigen_Processing_and_Presentation
PSMD13	5719	proteasome 26S subunit, non-ATPase 13	HSPC027|Rpn9|S11|p40.5		11	Antigen_Processing_and_Presentation
PSME1	5720	proteasome activator subunit 1	HEL-S-129m|IFI5111|PA28A|PA28alpha|REGalpha		14	Antigen_Processing_and_Presentation
PSME1	5720	proteasome activator subunit 1	HEL-S-129m|IFI5111|PA28A|PA28alpha|REGalpha		14	Antigen_Processing_and_Presentation
PSME2	5721	proteasome activator subunit 2	PA28B|PA28beta|REGbeta		14	Antigen_Processing_and_Presentation
PSME2	5721	proteasome activator subunit 2	PA28B|PA28beta|REGbeta		14	Antigen_Processing_and_Presentation
RELB	5971	RELB proto-oncogene, NF-kB subunit	I-REL|IMD53|IREL|REL-B		19	Antigen_Processing_and_Presentation
RFX5	5993	regulatory factor X5	-		1	Antigen_Processing_and_Presentation
RFXAP	5994	regulatory factor X associated protein	-		13	Antigen_Processing_and_Presentation
SLC10A2	6555	solute carrier family 10 member 2	ASBT|IBAT|ISBT|NTCP2|PBAM		13	Antigen_Processing_and_Presentation
TAP1	6890	transporter 1, ATP binding cassette subfamily B member	ABC17|ABCB2|APT1|D6S114E|PSF-1|PSF1|RING4|TAP1*0102N|TAP1N		6	Antigen_Processing_and_Presentation
TAP2	6891	transporter 2, ATP binding cassette subfamily B member	ABC18|ABCB3|APT2|D6S217E|PSF-2|PSF2|RING11		6	Antigen_Processing_and_Presentation

TAPBP	6892	TAP binding protein	NGS17|TAPA|TPN|TPSN	6	Antigen_Processing_and_Presentation
THBS1	7057	thrombospondin 1	THBS|THBS-1|TSP|TSP-1|TSP1	15	Antigen_Processing_and_Presentation
SEM1	7979	SEM1 26S proteasome complex subunit	C7orf76|DSS1|ECD|SHFD1|SHFM1|SHSF1|Shfdg1	7	Antigen_Processing_and_Presentation
KLRC4	8302	killer cell lectin like receptor C4	NKG2-F|NKG2F	12	Antigen_Processing_and_Presentation
AP3B1	8546	adaptor related protein complex 3 subunit beta 1	ADTB3|ADTB3A|HPS|HPS2|PE	5	Antigen_Processing_and_Presentation
RFXANK	8625	regulatory factor X associated ankyrin containing protein	ANKRA1|BLS|F14150_1|RFX-B	19	Antigen_Processing_and_Presentation
PSMD6	9861	proteasome 26S subunit, non-ATPase 6	Rpn7|S10|SGA-113M|p42A|p44S10	3	Antigen_Processing_and_Presentation
PSME3	10197	proteasome activator subunit 3	HEL-S-283|Ki|PA28-gamma|PA28G|PA28gamma|REG-GAMMA	17	Antigen_Processing_and_Presentation
PSMD14	10213	proteasome 26S subunit, non-ATPase 14	PAD1|POH1|RPN11	2	Antigen_Processing_and_Presentation
CLEC4M	10332	C-type lectin domain family 4 member M	CD209L|CD299|DC-SIGN2|DC-SIGNR|DCSIGNR|HP10347|L-SIGN|LSIGN	19	Antigen_Processing_and_Presentation
IFI30	10437	IFI30 lysosomal thiol reductase	GILT|IFI-30|IP-30|IP30	19	Antigen_Processing_and_Presentation
PROCR	10544	protein C receptor	CCCA|CCD41|EPCR	20	Antigen_Processing_and_Presentation
ADRM1	11047	adhesion regulating molecule 1	ARM-1|ARM1|GP110	20	Antigen_Processing_and_Presentation
ECPAS	23392	Ecm29 proteasome adaptor and scaffold	ECM29|KIAA0368	9	Antigen_Processing_and_Presentation
TRPC4AP	26133	transient receptor potential cation channel subfamily C member 4 associated protein	C20orf188|PPP1R158|TRRP4AP|TRUSS	20	Antigen_Processing_and_Presentation

CD209	30835	CD209 molecule	CDSIGN|CLEC4L|DC-SIGN|DC-SIGN1	19	Antigen_Processing_and_Presentation
UBXN1	51035	UBX domain protein 1	2B28|SAKS1|UBXD10	11	Antigen_Processing_and_Presentation
ERAP1	51752	endoplasmic reticulum aminopeptidase 1	A-LAP|ALAP|APPILS|ARTS-1|ARTS1|ERAAP|ERAAP1|PILS-AP|PILSAP	5	Antigen_Processing_and_Presentation
TAPBPL	55080	TAP binding protein like	TAPBP-R|TAPBPR	12	Antigen_Processing_and_Presentation
KIR2DL5A	57292	killer cell immunoglobulin like receptor, two Ig domains and long cytoplasmic tail 5A	CD158F|KIR2DL5|KIR2DL5.1|KIR2DL5.3	19	Antigen_Processing_and_Presentation
ERAP2	64167	endoplasmic reticulum aminopeptidase 2	L-RAP|LRAP	5	Antigen_Processing_and_Presentation
ULBP3	79465	UL16 binding protein 3	N2DL-3|NKG2DL3|RAET1N	6	Antigen_Processing_and_Presentation
ULBP2	80328	UL16 binding protein 2	ALCAN-alpha|N2DL2|NKG2DL2|RAET1H|RAET1L	6	Antigen_Processing_and_Presentation
ULBP1	80329	UL16 binding protein 1	N2DL-1|NKG2DL1|RAET1I	6	Antigen_Processing_and_Presentation
KIR3DL3	115653	killer cell immunoglobulin like receptor, three Ig domains and long cytoplasmic tail 3	CD158Z|KIR3DL7|KIR44|KIRC1	19	Antigen_Processing_and_Presentation
RAET1E	135250	retinoic acid early transcript 1E	LETAL|N2DL-4|NKG2DL4|RAET1E2|RL-4|ULBP4|bA350J20.7	6	Antigen_Processing_and_Presentation
RAET1L	154064	retinoic acid early transcript 1L	ULBP6	6	Antigen_Processing_and_Presentation
UBR1	197131	ubiquitin protein ligase E3 component n-recognin 1	JBS	15	Antigen_Processing_and_Presentation

RAET1G	353091	retinoic acid early transcript 1G	ULBP5	6	Antigen_Processing_and_Presentation
PDIA2	64714	protein disulfide isomerase family A member 2	PDA2|PDI|PDIP|PDIR	16	Antigen_Processing_and_Presentation
HAMP	57817	hepcidin antimicrobial peptide	HEPC|HFE2B|LEAP1|PLTR	19	Antimicrobials
PI3	5266	peptidase inhibitor 3	ESI|SKALP|WAP3|WFDC14|cementoin	20	Antimicrobials
CAMP	820	cathelicidin antimicrobial peptide	CAP-18|CAP18|CRAMP|FALL-39|FALL39|HSD26|LL37	3	Antimicrobials
DEFB4A	1673	defensin beta 4A	BD-2|DEFB-2|DEFB102|DEFB2|DEFB4|HBD-2|SAP1	8	Antimicrobials
PPBP	5473	pro-platelet basic protein	B-TG1|Beta-TG|CTAP-III|CTAP3|CTAPIII|CXCL7|LA-PF4|LDGF|MDGF|NAP-2|PBP|SCYB7|TC1|TC2|TGB|TGB1|THBGB|THBGB1	4	Antimicrobials
REG3G	130120	regenerating family member 3 gamma	LPPM429|PAP IB|PAP-1B|PAP1B|PAPIB|REG III|REG-III|UNQ429	2	Antimicrobials
CXCL14	9547	C-X-C motif chemokine ligand 14	BMAC|BRAK|KEC|KS1|MIP-2g|MIP2G|NJAC|SCYB14	5	Antimicrobials
CXCL16	58191	C-X-C motif chemokine ligand 16	CXCLG16|SR-PSOX|SRPSOX	17	Antimicrobials
SLPI	6590	secretory leukocyte peptidase inhibitor	ALK1|ALP|BLPI|HUSI|HUSI-I|MPI|WAP4|WFDC4	20	Antimicrobials
CXCL8	3576	C-X-C motif chemokine ligand 8	GCP-1|GCP1|IL8|LECT|LUCT|LYNAP|MDNCF|MONAP|NAF|NAP-1|NAP1|SCYB8	4	Antimicrobials
CXCL1	3627	C-X-C motif chemokine ligand 10	C7|IFI10|INP10|IP-10|SCYB10|crg-2|gI	4	Antimicrobials

0			P-10|mob-1		
CXCL9	4283	C-X-C motif chemokine ligand 9	CMK|Humig|MIG|SCYB9|crg-10	4	Antimicrobials
CXCL5	6374	C-X-C motif chemokine ligand 5	ENA-78|SCYB5	4	Antimicrobials
CXCL11	6373	C-X-C motif chemokine ligand 11	H174|I-TAC|IP-9|IP9|SCYB11|SCYB9B|b-R1	4	Antimicrobials
CXCL6	6372	C-X-C motif chemokine ligand 6	CKA-3|GCP-2|GCP2|SCYB6	4	Antimicrobials
CXCL1	2919	C-X-C motif chemokine ligand 1	FSP|GRO1|GROa|MGSA|MGSA-a|NAP-3|SCYB1	4	Antimicrobials
CXCL12	6387	C-X-C motif chemokine ligand 12	IRH|PBSF|SCYB12|SDF1|TLSF|TPAR1	10	Antimicrobials
CXCL13	10563	C-X-C motif chemokine ligand 13	ANGIE|ANGIE2|BCA-1|BCA1|BLC|BLR1L|SCYB13	4	Antimicrobials
CXCL2	2920	C-X-C motif chemokine ligand 2	CINC-2a|GRO2|GROb|MGSA-b|MIP-2a|MIP2|MIP2A|SCYB2	4	Antimicrobials
PF4	5196	platelet factor 4	CXCL4|PF-4|SCYB4	4	Antimicrobials
XCL1	6375	X-C motif chemokine ligand 1	ATAC|LPTN|LTN|SCM-1|SCM-1a|SCM1|SCM1A|SCYC1	1	Antimicrobials
CXCL3	2921	C-X-C motif chemokine ligand 3	CINC-2b|GRO3|GROg|MIP-2b|MIP2B|SCYB3	4	Antimicrobials
DEFB103B	55894	defensin beta 103B	BD-3|DEFB-3|DEFB103|DEFB3|HBD-3|HBD3|HBP-3|HBP3	8	Antimicrobials
CCL13	6357	C-C motif chemokine ligand 13	CKb10|MCP-4|NCC-1|NCC1|SCYA13|SCYL1	17	Antimicrobials
CCL1	6346	C-C motif chemokine ligand 1	I-309|P500|SCYA1|SISe|TCA3	17	Antimicrobials
DEFB1	1672	defensin beta 1	BD1|DEFB-1|DEFB101|HBD1	8	Antimicrobials
CCL8	6355	C-C motif chemokine ligand 8	HC14|MCP-2|MCP2|SCYA10|SCYA8	17	Antimicrobials
ELANE	1991	elastase, neutrophil expressed	ELA2|GE|HLE|HNE|NE|PMN-E|SCN1	19	Antimicrobials
DEFB1	4143	defensin beta 103A	BD-3|DEFB-3|DEFB103|DEFB3|HBD3|HBP-3	8	Antimicrobials

03A	25		|HBP3		
DEFA3	1668	defensin alpha 3	DEF3|HNP-3|HNP3|HP-3|HP3	8	Antimicrobials
DEFA1	1667	defensin alpha 1	DEF1|DEFA2|HNP-1|HP-1|HP1|MRS	8	Antimicrobials
TMSB10	9168	thymosin beta 10	MIG12|TB10	2	Antimicrobials
DEFA6	1671	defensin alpha 6	DEF6|HD-6	8	Antimicrobials
DEFA5	1670	defensin alpha 5	DEF5|HD-5	8	Antimicrobials
DEFA4	1669	defensin alpha 4	DEF4|HNP-4|HP-4|HP4	8	Antimicrobials
LCN2	3934	lipocalin 2	24p3|MSFI|NGAL|p25	9	Antimicrobials
LCN1	3933	lipocalin 1	PMFA|TLC|TP|VEGP	9	Antimicrobials
COLEC10	10584	collectin subfamily member 10	3MC3|CL-34|CLL1	8	Antimicrobials
BPI	671	bactericidal permeability increasing protein	BPIFD1|rBPI	20	Antimicrobials
S100A9	6280	S100 calcium binding protein A9	60B8AG|CAGB|CFAG|CGLB|L1AG|LIAG|MAC387|MIF|MRP14|NIF|P14	1	Antimicrobials
S100A8	6279	S100 calcium binding protein A8	60B8AG|CAGA|CFAG|CGLA|CP-10|L1Ag|MA387|MIF|MRP8|NIF|P8	1	Antimicrobials
DCD	117159	dermcidin	AIDD|DCD-1|DSEP|HCAP|PIF	12	Antimicrobials
LCN6	158062	lipocalin 6	LCN5|UNQ643|hLcn5	9	Antimicrobials
S100A12	6283	S100 calcium binding protein A12	CAAF1|CAGC|CGRP|ENRAGE|MRP-6|MRP6|p6	1	Antimicrobials
HTN3	3347	histatin 3	HIS2|HTN2|HTN5|PB	4	Antimicrobials
LCN8	138307	lipocalin 8	EP17|LCN5	9	Antimicrobials

DEFA1B	728358	defensin alpha 1B	HNP-1|HP-1|HP1		8	Antimicrobials
CCR10	2826	C-C motif chemokine receptor 10	GPR2		17	Antimicrobials
CELA1	1990	chymotrypsin like elastase 1	ELA1		12	Antimicrobials
DEFB106A	245909	defensin beta 106A	BD-6|DEFB-6|DEFB106		8	Antimicrobials
PENK	5179	proenkephalin	PE|PENK-A		8	Antimicrobials
BPIFC	254240	BPI fold containing family C	BPIL2		22	Antimicrobials
MMP12	4321	matrix metallopeptidase 12	HME|ME|MME|MMP-12		11	Antimicrobials
BPIFB6	128859	BPI fold containing family B member 6	BPIL3|LPLUNC6		20	Antimicrobials
LEAP2	116842	liver enriched antimicrobial peptide 2	LEAP-2		5	Antimicrobials
SFTPD	6441	surfactant protein D	COLEC7|PSP-D|SFTP4|SP-D		10	Antimicrobials
LCN9	392399	lipocalin 9	HEL129		9	Antimicrobials
BPIFB2	80341	BPI fold containing family B member 2	BPIL1|C20orf184|LPLUNC2|RYSR|dJ726C3.2		20	Antimicrobials
PTGDS	5730	prostaglandin D2 synthase	L-PGDS|LPGDS|PDS|PGD2|PGDS|PGDS2		9	Antimicrobials
TMSB4X	7114	thymosin beta 4 X-linked	FX|PTMB4|TB4X|TMSB4	X		Antimicrobials
PGLYRP1	8993	peptidoglycan recognition protein 1	PGLYRP|PGRP|PGRP-S|PGRPS|TAG7|TNFSF3L		19	Antimicrobials
ZC3HAV1	56829	zinc finger CCCH-type containing, antiviral 1	ARTD13|FLB6421|PARP13|ZAP|ZC3H2|ZC3HDC2		7	Antimicrobials

TMSB15A	11013	thymosin beta 15a	TMSB15|TMSB15B|TMSL8|TMSNB|Tb15|TbNB	X	Antimicrobials
S100B	6285	S100 calcium binding protein B	NEF|S100|S100-B|S100beta	21	Antimicrobials
S100A13	6284	S100 calcium binding protein A13	-	1	Antimicrobials
S100A6	6277	S100 calcium binding protein A6	2A9|5B10|CABP|CACY|PRA|S10A6	1	Antimicrobials
DEFB119	245932	defensin beta 119	DEFB-19|DEFB-20|DEFB120|DEFB20|ESC42-RELA|ESC42-RELB	20	Antimicrobials
DEFB107A	245910	defensin beta 107A	BD-7|DEFB-7|DEFB107	8	Antimicrobials
DEFB105A	245908	defensin beta 105A	BD-5|DEFB-5|DEFB105	8	Antimicrobials
SERPIND1	3053	serpin family D member 1	D22S673|HC2|HCF2|HCII|HLS2|LS2|THPH10	22	Antimicrobials
DEFB129	140881	defensin beta 129	C20orf87|DEFB-29|DEFB29|bA530N10.3|hBD-29	20	Antimicrobials
DEFB127	140850	defensin beta 127	C20orf73|DEF-27|DEFB-27|DEFB27|bA530N10.2|hBD-27	20	Antimicrobials
S100P	6286	S100 calcium binding protein P	MIG9	4	Antimicrobials
S100A7	6278	S100 calcium binding protein A7	PSOR1|S100A7c	1	Antimicrobials
DEFB104A	140596	defensin beta 104A	BD-4|DEFB-4|DEFB104|DEFB4|hBD-4	8	Antimicrobials
DEFB126	81623	defensin beta 126	C20orf8|DEFB-26|DEFB26|HBD26|bA530N10.1|hBD-26	20	Antimicrobials

DEFB106B	503841	defensin beta 106B	BD-6|DEFB-6	8	Antimicrobials
DEFB104B	503618	defensin beta 104B	BD-4|DEFB-4|hBD-4	8	Antimicrobials
DEFB107B	503614	defensin beta 107B	HsT21816	8	Antimicrobials
PGLYRP3	114771	peptidoglycan recognition protein 3	PGLYRPIalpha|PGRP-Ialpha|PGRPIA	1	Antimicrobials
PGLYRP2	114770	peptidoglycan recognition protein 2	HMFT0141|PGLYRPL|PGRP-L|PGRPL|TAGL-like|tagL|tagL-alpha|tagl-beta	19	Antimicrobials
S100A10	6281	S100 calcium binding protein A10	42C|ANX2L|ANX2LG|CAL1L|CLP11|Ca[1]|GP11|P11|p10	1	Antimicrobials
S100A2	6273	S100 calcium binding protein A2	CAN19|S100L	1	Antimicrobials
DEFB125	245938	defensin beta 125	DEFB-25	20	Antimicrobials
DEFB123	245936	defensin beta 123	DEFB-23|DEFB23|ESC42-RELD	20	Antimicrobials
DEFB105B	504180	defensin beta 105B	BD-5|DEFB-5	8	Antimicrobials
DEFB132	400830	defensin beta 132	BD-32|DEFB-32|DEFB32|HEL-75|KFLL827|UNQ827	20	Antimicrobials
BPIFB3	359710	BPI fold containing family B member 3	C20orf185|LPLUNC3|RYA3	20	Antimicrobials
LCN12	286256	lipocalin 12	-	9	Antimicrobials

PGLYRP4	57115	peptidoglycan recognition protein 4	PGLYRPIbeta|PGRP-Ibeta|PGRPIB|SBBI67		1	Antimicrobials
S100A11	6282	S100 calcium binding protein A11	HEL-S-43|MLN70|S100C		1	Antimicrobials
S100A5	6276	S100 calcium binding protein A5	S100D		1	Antimicrobials
S100A3	6274	S100 calcium binding protein A3	S100E		1	Antimicrobials
S100A1	6271	S100 calcium binding protein A1	S100|S100-alpha|S100A		1	Antimicrobials
DEFB128	245939	defensin beta 128	DEFB-28|DEFB28|hBD-28		20	Antimicrobials
DEFB108B	245911	defensin beta 108B	DEFB-8|hBD-8		11	Antimicrobials
HTN1	3346	histatin 1	HIS1		4	Antimicrobials
LMBR1L	55716	limb development membrane protein 1 like	LIMR		12	Antimicrobials
S100A7A	338324	S100 calcium binding protein A7A	NICE-2|NICE2|S100A15|S100A7L1|S100A7f		1	Antimicrobials
DEFB118	117285	defensin beta 118	C20orf63|DEFB-18|ESC42|ESP13.6		20	Antimicrobials
COLEC12	81035	collectin subfamily member 12	CLP1|NSR2|SCARA4|SRCL		18	Antimicrobials
TMSB4Y	9087	thymosin beta 4 Y-linked	TB4Y	Y		Antimicrobials
DEFB1	6444	defensin beta 131A	DEFB-31|DEFB131		4	Antimicrobials

31A	14					
DEFB134	613211	defensin beta 134	-		8	Antimicrobials
DEFB130A	245940	defensin beta 130A	DEFB-30|DEFB130|DEFB130L|DEFB30		8	Antimicrobials
DEFB124	245937	defensin beta 124	DEFB-24		20	Antimicrobials
DEFB121	245934	defensin beta 121	DEFB21|ESC42RELC		20	Antimicrobials
DEFB116	245930	defensin beta 116	DEFB-16		20	Antimicrobials
DEFB115	245929	defensin beta 115	DEFB-15		20	Antimicrobials
DEFB114	245928	defensin beta 114	DEFB-14|DEFB14		6	Antimicrobials
DEFB113	245927	defensin beta 113	DEFB-13		6	Antimicrobials
DEFB112	245915	defensin beta 112	DEFB-12		6	Antimicrobials
DEFB110	245913	defensin beta 110	DEFB-10|DEFB-11|DEFB111		6	Antimicrobials
TMSB15B	286527	thymosin beta 15B	TMSB15A|TMSL8|TMSNB|Tbeta15b	X		Antimicrobials
DEFB133	403339	defensin beta 133	-		6	Antimicrobials
S100Z	1705	S100 calcium binding protein Z	Gm625|S100-zeta		5	Antimicrobials

	91				
MAVS	57506	mitochondrial antiviral signaling protein	CARDIF|IPS-1|IPS1|VISA	20	Antimicrobials
TMSB4XP8	7117	TMSB4X pseudogene 8	TMSL3	4	Antimicrobials
S100A14	57402	S100 calcium binding protein A14	BCMP84|S100A15	1	Antimicrobials
LCN10	414332	lipocalin 10	-	9	Antimicrobials
S100A16	140576	S100 calcium binding protein A16	AAG13|DT1P1A7|S100F	1	Antimicrobials
DEFB136	613210	defensin beta 136	DEFB137	8	Antimicrobials
DEFB135	613209	defensin beta 135	DEFB136	8	Antimicrobials
DEFB117	245931	defensin beta 117 (pseudogene)	DEFB-17	20	Antimicrobials
DEFB110	245913	defensin beta 110	DEFB-10|DEFB-11|DEFB111	6	Antimicrobials
ZC3HAV1L	92092	zinc finger CCCH-type containing, antiviral 1 like	C7orf39	7	Antimicrobials
S100A7L2	645922	S100 calcium binding protein A7 like 2	S100a7b	1	Antimicrobials
MBL3P	50639	mannose-binding lectin family member 3, pseudogene	COLEC2|MBL	10	Antimicrobials
DEFB4	1002	defensin beta 4B	DEFB4P	8	Antimicrobials

B	89462					
BPIFB4	149954	BPI fold containing family B member 4	C20orf186|LPLUNC4|RY2G5|dJ726C3.5		20	Antimicrobials
IFNAR1	3454	interferon alpha and beta receptor subunit 1	AVP|IFN-alpha-REC|IFNAR|IFNBR|IFRC		21	Antimicrobials
AZU1	566	azurocidin 1	AZAMP|AZU|CAP37|HBP|HUMAZUR|NAZC|hHBP		19	Antimicrobials
DEFB131B	100129216	defensin beta 131B	-		11	Antimicrobials
DEFA1A3	613253	defensin alpha 1 and alpha 3, variable copy number locus	DEFA1|DEFA3|DEFT1P		8	Antimicrobials
LCN1P1	286310	lipocalin 1 pseudogene 1	LCN1L1|bA430N14.2		9	Antimicrobials
S100G	795	S100 calcium binding protein G	CABP|CABP1|CABP9K|CALB3	X		Antimicrobials
DEFA7P	724067	defensin alpha 7, pseudogene	DEFA7		8	Antimicrobials
DEFB130B	100133267	defensin beta 130B	-		8	Antimicrobials
DEFB108F	100133128	defensin beta 108F (pseudogene)	DEFB108P5		4	Antimicrobials
DEFB131C	10012817	defensin beta 131C (pseudogene)	-		8	Antimicrobials

<table>
<tr><td></td><td>4</td><td></td><td></td><td></td><td></td></tr>
<tr><td>TCHHL1</td><td>126637</td><td>trichohyalin like 1</td><td>S100A17|THHL1</td><td>1</td><td>Antimicrobials</td></tr>
<tr><td>TINAGL1</td><td>64129</td><td>tubulointerstitial nephritis antigen like 1</td><td>ARG1|LCN7|LIECG3|TINAGRP</td><td>1</td><td>Antimicrobials</td></tr>
<tr><td>IFNGR1</td><td>3459</td><td>interferon gamma receptor 1</td><td>CD119|IFNGR|IMD27A|IMD27B</td><td>6</td><td>Antimicrobials</td></tr>
<tr><td>SLC22A17</td><td>51310</td><td>solute carrier family 22 member 17</td><td>24p3R|BOCT|BOIT|NGALR|NGALR2|NGALR3|hBOIT</td><td>14</td><td>Antimicrobials</td></tr>
<tr><td>WFIKKN1</td><td>117166</td><td>WAP, follistatin/kazal, immunoglobulin, kunitz and netrin domain containing 1</td><td>C16orf12|RJD2|WFDC20A|WFIKKN</td><td>16</td><td>Antimicrobials</td></tr>
<tr><td>WFDC2</td><td>10406</td><td>WAP four-disulfide core domain 2</td><td>EDDM4|HE4|WAP5|dJ461P17.6</td><td>20</td><td>Antimicrobials</td></tr>
<tr><td>IL6</td><td>3569</td><td>interleukin 6</td><td>BSF-2|BSF2|CDF|HGF|HSF|IFN-beta-2|IFNB2|IL-6</td><td>7</td><td>Antimicrobials</td></tr>
<tr><td>UMODL1</td><td>89766</td><td>uromodulin like 1</td><td>-</td><td>21</td><td>Antimicrobials</td></tr>
<tr><td>TGFB1</td><td>7040</td><td>transforming growth factor beta 1</td><td>CED|DPD1|IBDIMDE|LAP|TGF-beta1|TGFB|TGFbeta</td><td>19</td><td>Antimicrobials</td></tr>
<tr><td>PF4V1</td><td>5197</td><td>platelet factor 4 variant 1</td><td>CXCL4L1|CXCL4V1|PF4-ALT|PF4A|SCYB4V1</td><td>4</td><td>Antimicrobials</td></tr>
<tr><td>MMP9</td><td>4318</td><td>matrix metallopeptidase 9</td><td>CLG4B|GELB|MANDP2|MMP-9</td><td>20</td><td>Antimicrobials</td></tr>
<tr><td>ANOS1</td><td>3730</td><td>anosmin 1</td><td>ADMLX|HH1|HHA|KAL|KAL1|KALIG-1|KMS|WFDC19</td><td>X</td><td>Antimicrobials</td></tr>
<tr><td>TLR4</td><td>7099</td><td>toll like receptor 4</td><td>ARMD10|CD284|TLR-4|TOLL</td><td>9</td><td>Antimicrobials</td></tr>
<tr><td>IFNG</td><td>3458</td><td>interferon gamma</td><td>IFG|IFI</td><td>12</td><td>Antimicrobials</td></tr>
<tr><td>SPAG1</td><td>1040</td><td>sperm associated antigen 11B</td><td>EDDM2B|EP2|EP2C|EP2D|HE2|HE2C|SPAG11</td><td>8</td><td>Antimicrobials</td></tr>
</table>

1B	7		|SPAG11A		
A2M	2	alpha-2-macroglobulin	A2MD|CPAMD5|FWP007|S863-7	12	Antimicrobials
CTSL	1514	cathepsin L	CATL|CTSL1|MEP	9	Antimicrobials
NFKB1	4790	nuclear factor kappa B subunit 1	CVID12|EBP-1|KBF1|NF-kB|NF-kB1|NF-kappa-B1|NF-kappaB|NF-kappabeta|NFKB-p105|NFKB-p50|NFkappaB	4	Antimicrobials
APOBEC3G	60489	apolipoprotein B mRNA editing enzyme catalytic subunit 3G	A3G|ARCD|ARP-9|ARP9|CEM-15|CEM15|MDS019|bK150C2.7|dJ494G10.1	22	Antimicrobials
FABP6	2172	fatty acid binding protein 6	I-15P|I-BABP|I-BALB|I-BAP|ILBP|ILBP3|ILLBP	5	Antimicrobials
NOD2	64127	nucleotide binding oligomerization domain containing 2	ACUG|BLAU|BLAUS|CARD15|CD|CLR16.3|IBD1|NLRC2|NOD2B|PSORAS1|YAOS	16	Antimicrobials
MBL2	4153	mannose binding lectin 2	COLEC1|HSMBPC|MBL|MBL2D|MBP|MBP-C|MBP1|MBPD	10	Antimicrobials
SFTPA1	653509	surfactant protein A1	COLEC4|PSAP|PSP-A|PSPA|SFTP1|SFTPA1B|SP-A|SP-A1|SP-A1 beta|SP-A1 delta|SP-A1 epsilon|SP-A1 gamma|SPA|SPA1	10	Antimicrobials
RBP1	5947	retinol binding protein 1	CRABP-I|CRBP|CRBP1|CRBPI|RBPC	3	Antimicrobials
TLR2	7097	toll like receptor 2	CD282|TIL4	4	Antimicrobials
SLC40A1	30061	solute carrier family 40 member 1	FPN1|HFE4|IREG1|MST079|MSTP079|MTP1|SLC11A3	2	Antimicrobials
PLAU	5328	plasminogen activator, urokinase	ATF|BDPLT5|QPD|UPA|URK|u-PA	10	Antimicrobials
IL1B	3553	interleukin 1 beta	IL-1|IL1-BETA|IL1F2|IL1beta	2	Antimicrobials
PAEP	5047	progestagen associated endometrial protein	GD|GdA|GdF|GdS|PAEG|PEP|PP14|ZIF-1	9	Antimicrobials
HJV	1487	hemojuvelin BMP co-receptor	HFE2|HFE2A|JH|RGMC	1	Antimicrobials

	38				
MUC5AC	4586	mucin 5AC, oligomeric mucus/gel-forming	MUC5|TBM|leB|mucin	11	Antimicrobials
CTSS	1520	cathepsin S	-	1	Antimicrobials
OBP2A	29991	odorant binding protein 2A	LCN13|OBP|OBP2C|OBPIIa|hOBPIIa	9	Antimicrobials
PLTP	5360	phospholipid transfer protein	BPIFE|HDLCQ9	20	Antimicrobials
MX1	4599	MX dynamin like GTPase 1	IFI-78K|IFI78|MX|MxA|lncMX1-215	21	Antimicrobials
DDX58	23586	DExD/H-box helicase 58	RIG-I|RIG1|RIGI|RLR-1|SGMRT2	9	Antimicrobials
IFNL1	282618	interferon lambda 1	IL-29|IL29	19	Antimicrobials
IRF3	3661	interferon regulatory factor 3	IIAE7	19	Antimicrobials
SFTPA2	729238	surfactant protein A2	COLEC5|PSAP|PSP-A|PSPA|SFTP1|SFTPA2B|SP-2A|SP-A|SPA2|SPAII	10	Antimicrobials
LPA	4018	lipoprotein(a)	AK38|APOA|LP	6	Antimicrobials
LBP	3929	lipopolysaccharide binding protein	BPIFD2	20	Antimicrobials
RBP4	5950	retinol binding protein 4	MCOPCB10|RDCCAS	10	Antimicrobials
SFTPA1	653509	surfactant protein A1	COLEC4|PSAP|PSP-A|PSPA|SFTP1|SFTPA1B|SP-A|SP-A1|SP-A1 beta|SP-A1 delta|SP-A1 epsilon|SP-A1 gamma|SPA|SPA1	10	Antimicrobials
NOX4	50507	NADPH oxidase 4	KOX|KOX-1|RENOX	11	Antimicrobials
LTF	4057	lactotransferrin	GIG12|HEL110|HLF2|LF	3	Antimicrobials
IFNB1	3456	interferon beta 1	IFB|IFF|IFN-beta|IFNB	9	Antimicrobials

RBP5	83758	retinol binding protein 5	CRBP-III|CRBP3|CRBPIII|HRBPiso	12	Antimicrobials
FABP7	2173	fatty acid binding protein 7	B-FABP|BLBP|FABPB|MRG	6	Antimicrobials
FABP5	2171	fatty acid binding protein 5	E-FABP|EFABP|KFABP|PA-FABP|PAFABP	8	Antimicrobials
FABP3	2170	fatty acid binding protein 3	FABP11|H-FABP|M-FABP|MDGI|O-FABP	1	Antimicrobials
FABP2	2169	fatty acid binding protein 2	FABPI|I-FABP	4	Antimicrobials
FABP4	2167	fatty acid binding protein 4	A-FABP|AFABP|ALBP|HEL-S-104|aP2	8	Antimicrobials
R3HDML	140902	R3H domain containing like	dJ881L22.3	20	Antimicrobials
BPIFA3	128861	BPI fold containing family A member 3	C20orf71|SPLUNC3	20	Antimicrobials
BPIFB1	92747	BPI fold containing family B member 1	C20orf114|LPLUNC1	20	Antimicrobials
OASL	8638	2'-5'-oligoadenylate synthetase like	OASL1|OASLd|TRIP-14|TRIP14|p59 OASL|p59-OASL|p59OASL	12	Antimicrobials
CRABP2	1382	cellular retinoic acid binding protein 2	CRABP-II|RBP6	1	Antimicrobials
CRABP1	1381	cellular retinoic acid binding protein 1	CRABP|CRABP-I|CRABPI|RBP5	15	Antimicrobials
RBP7	116362	retinol binding protein 7	CRABP4|CRBP4|CRBPIV	1	Antimicrobials
DUOX1	53905	dual oxidase 1	LNOX1|NOXEF1|THOX1	15	Antimicrobials
OBP2B	29989	odorant binding protein 2B	LCN14|OBPIIb	9	Antimicrobials
RBP2	5948	retinol binding protein 2	CRABP-II|CRBP2|CRBPII|RBPC2	3	Antimicrobials

LCN15	389812	lipocalin 15	PRO6093|UNQ2541		9	Antimicrobials
CETP	1071	cholesteryl ester transfer protein	BPIFF|HDLCQ10		16	Antimicrobials
FABP12	646486	fatty acid binding protein 12	-		8	Antimicrobials
FABP9	646480	fatty acid binding protein 9	PERF|PERF15|T-FABP|TLBP		8	Antimicrobials
BPIFA1	51297	BPI fold containing family A member 1	LUNX|NASG|PLUNC|SPLUNC1|SPURT|bA49G10.5		20	Antimicrobials
LCNL1	401562	lipocalin like 1	-		9	Antimicrobials
C8G	733	complement C8 gamma chain	C8C		9	Antimicrobials
SPAG11A	653423	sperm associated antigen 11A	EDDM2A|HE2		8	Antimicrobials
PI15	51050	peptidase inhibitor 15	CRISP8|P24TI|P25TI		8	Antimicrobials
NOX1	27035	NADPH oxidase 1	GP91-2|MOX1|NOH-1|NOH1	X		Antimicrobials
PMP2	5375	peripheral myelin protein 2	CMT1G|FABP8|M-FABP|MP2|P2		8	Antimicrobials
APOD	347	apolipoprotein D	-		3	Antimicrobials
ORM2	5005	orosomucoid 2	AGP-B|AGP-B'|AGP2		9	Antimicrobials
ORM1	5004	orosomucoid 1	AGP-A|AGP1|HEL-S-153w|ORM		9	Antimicrobials
TNF	7124	tumor necrosis factor	DIF|TNF-alpha|TNFA|TNFSF2|TNLG1F		6	Antimicrobials
CTSG	1511	cathepsin G	CATG|CG		14	Antimicrobials
PRTN3	5657	proteinase 3	ACPA|AGP7|C-ANCA|CANCA|MBN|MBT|NP-4|NP4|P29|PR-3|PR3		19	Antimicrobials

MAPK1	5594	mitogen-activated protein kinase 1	ERK|ERK-2|ERK2|ERT1|MAPK2|P42MAPK|PRKM1|PRKM2|p38|p40|p41|p41mapk|p42-MAPK		22	Antimicrobials
PML	5371	PML nuclear body scaffold	MYL|PP8675|RNF71|TRIM19		15	Antimicrobials
AEN	64782	apoptosis enhancing nuclease	ISG20L1|pp12744		15	Antimicrobials
CYBB	1536	cytochrome b-245 beta chain	AMCBX2|CGD|GP91-1|GP91-PHOX|GP91PHOX|IMD34|NOX2|p91-PHOX	X		Antimicrobials
BPIFA2	140683	BPI fold containing family A member 2	C20orf70|PSP|SPLUNC2|bA49G10.1		20	Antimicrobials
ISG20	3669	interferon stimulated exonuclease gene 20	CD25|HEM45		15	Antimicrobials
BCL3	602	BCL3 transcription coactivator	BCL4|D19S37		19	Antimicrobials
ISG20L2	81875	interferon stimulated exonuclease gene 20 like 2	HSD38		1	Antimicrobials
NOX5	79400	NADPH oxidase 5	-		15	Antimicrobials
NOX3	50508	NADPH oxidase 3	GP91-3|MOX-2		6	Antimicrobials
DUOX2	50506	dual oxidase 2	LNOX2|NOXEF2|P138-TOX|TDH6|THOX2		15	Antimicrobials
TLR3	7098	toll like receptor 3	CD283|IIAE2		4	Antimicrobials
TFRC	7037	transferrin receptor	CD71|IMD46|T9|TFR|TFR1|TR|TRFR|p90		3	Antimicrobials
IFIH1	64135	interferon induced with helicase C domain 1	AGS7|Hlcd|IDDM19|MDA-5|MDA5|RLR-2|SGMRT1		2	Antimicrobials
LRP1	4035	LDL receptor related protein 1	A2MR|APOER|APR|CD91|IGFBP-3R|IGFBP3R|IGFBP3R1|KPA|LRP|LRP1A|TGFBR5		12	Antimicrobials

TRIM5	85363	tripartite motif containing 5	RNF88|TRIM5alpha	11	Antimicrobials
IDO1	3620	indoleamine 2,3-dioxygenase 1	IDO|IDO-1|INDO	8	Antimicrobials
GDF15	9518	growth differentiation factor 15	GDF-15|MIC-1|MIC1|NAG-1|PDF|PLAB|PTGFB	19	Antimicrobials
NEDD4	4734	NEDD4 E3 ubiquitin protein ligase	NEDD4-1|RPF1	15	Antimicrobials
ADIPOQ	9370	adiponectin, C1Q and collagen domain containing	ACDC|ACRP30|ADIPQTL1|ADPN|APM-1|APM1|GBP28	3	Antimicrobials
STAT3	6774	signal transducer and activator of transcription 3	ADMIO|ADMIO1|APRF|HIES	17	Antimicrobials
STAT1	6772	signal transducer and activator of transcription 1	CANDF7|IMD31A|IMD31B|IMD31C|ISGF-3|STAT91	2	Antimicrobials
IFNL2	282616	interferon lambda 2	IL-28A|IL28A	19	Antimicrobials
SOCS3	9021	suppressor of cytokine signaling 3	ATOD4|CIS3|Cish3|SOCS-3|SSI-3|SSI3	17	Antimicrobials
SEMG1	6406	semenogelin 1	CT103|SEMG|SGI|dJ172H20.2	20	Antimicrobials
TNFSF10	8743	TNF superfamily member 10	APO2L|Apo-2L|CD253|TL2|TNLG6A|TRAIL	3	Antimicrobials
CCL20	6364	C-C motif chemokine ligand 20	CKb4|Exodus|LARC|MIP-3-alpha|MIP-3a|MIP3A|SCYA20|ST38	2	Antimicrobials
SOCS1	8651	suppressor of cytokine signaling 1	CIS1|CISH1|JAB|SOCS-1|SSI-1|SSI1|TIP-3|TIP3	16	Antimicrobials
RNASEL	6041	ribonuclease L	PRCA1|RNS4	1	Antimicrobials
IRF1	3659	interferon regulatory factor 1	IRF-1|MAR	5	Antimicrobials
IL15	3600	interleukin 15	IL-15	4	Antimicrobials

APOBEC3F	200316	apolipoprotein B mRNA editing enzyme catalytic subunit 3F	A3F|ARP8|BK150C2.4.MRNA|KA6		22	Antimicrobials
PLAAT4	5920	phospholipase A and acyltransferase 4	HRASLS4|HRSL4|PLA1/2-3|PLAAT-4|RARRES3|RIG1|TIG3		11	Antimicrobials
CHIT1	1118	chitinase 1	CHI3|CHIT|CHITD		1	Antimicrobials
IFNA1	3439	interferon alpha 1	IFL|IFN|IFN-ALPHA|IFN-alphaD|IFNA13|IFNA@|leIF D		9	Antimicrobials
CD40	958	CD40 molecule	Bp50|CDW40|TNFRSF5|p50		20	Antimicrobials
TLR7	51284	toll like receptor 7	TLR7-like	X		Antimicrobials
PPIA	5478	peptidylprolyl isomerase A	CYPA|CYPH|HEL-S-69p		7	Antimicrobials
HFE	3077	homeostatic iron regulator	HFE1|HH|HLA-H|MVCD7|TFQTL2		6	Antimicrobials
ZYX	7791	zyxin	ESP-2|HED-2		7	Antimicrobials
NLRX1	79671	NLR family member X1	CLR11.3|DLNB26|NOD26|NOD5|NOD9		11	Antimicrobials
PGC	5225	progastricsin	PEPC|PGII		6	Antimicrobials
VEGFA	7422	vascular endothelial growth factor A	MVCD1|VEGF|VPF		6	Antimicrobials
IKBKE	9641	inhibitor of nuclear factor kappa B kinase subunit epsilon	IKK-E|IKK-i|IKKE|IKKI		1	Antimicrobials
ISG15	9636	ISG15 ubiquitin like modifier	G1P2|IFI15|IMD38|IP17|UCRP|hUCRP		1	Antimicrobials
DHX58	79132	DExH-box helicase 58	D11LGP2|D11lgp2e|LGP2|RLR-3		17	Antimicrobials
TNFAIP3	7128	TNF alpha induced protein 3	A20|AISBL|OTUD7C|TNFA1P2		6	Antimicrobials
TFR2	7036	transferrin receptor 2	HFE3|TFRC2		7	Antimicrobials
FCN2	2220	ficolin 2	EBP-37|FCNL|P35|ficolin-2		9	Antimicrobials

MUC4	4585	mucin 4, cell surface associated	ASGP|HSA276359|MUC-4	3	Antimicrobials
F2R	2149	coagulation factor II thrombin receptor	CF2R|HTR|PAR-1|PAR1|TR	5	Antimicrobials
ELN	2006	elastin	ADCL1|SVAS|WBS|WS	7	Antimicrobials
IL27	246778	interleukin 27	IL-27|IL-27A|IL27A|IL27p28|IL30|p28	16	Antimicrobials
MAPT	4137	microtubule associated protein tau	DDPAC|FTDP-17|MAPTL|MSTD|MTBT1|MTBT2|PPND|PPP1R103|TAU	17	Antimicrobials
LYZ	4069	lysozyme	LYZF1|LZM	12	Antimicrobials
CCL5	6352	C-C motif chemokine ligand 5	D17S136E|RANTES|SCYA5|SIS-delta|SISd|TCP228|eoCP	17	Antimicrobials
LEP	3952	leptin	LEPD|OB|OBS	7	Antimicrobials
CYLD	1540	CYLD lysine 63 deubiquitinase	BRSS|CDMT|CYLD1|CYLDI|EAC|MFT|MFT1|SBS|TEM|USPL2	16	Antimicrobials
KLKB1	3818	kallikrein B1	KLK3|PKK|PKKD|PPK	4	Antimicrobials
CST4	1472	cystatin S	-	20	Antimicrobials
CSRP1	1465	cysteine and glycine rich protein 1	CRP|CRP1|CSRP|CYRP|D1S181E|HEL-141|HEL-S-286	1	Antimicrobials
MAPK14	1432	mitogen-activated protein kinase 14	CSBP|CSBP1|CSBP2|CSPB1|EXIP|Mxi2|PRKM14|PRKM15|RK|SAPK2A|p38|p38ALPHA	6	Antimicrobials
JUN	3725	Jun proto-oncogene, AP-1 transcription factor subunit	AP-1|AP1|c-Jun|cJUN|p39	1	Antimicrobials
ITGAV	3685	integrin subunit alpha V	CD51|MSK8|VNRA|VTNR	2	Antimicrobials
IRF5	3663	interferon regulatory factor 5	SLEB10	7	Antimicrobials
CCR6	1235	C-C motif chemokine receptor 6	BN-1|C-C CKR-6|CC-CKR-6|CCR-6|CD196|CKR-L3|CKRL3|CMKBR6|DCR2|DRY6|GPR29|GPRCY4|ST	6	Antimicrobials

			RL22		
IL12B	3593	interleukin 12B	CLMF|CLMF2|IL-12B|IMD28|IMD29|NKSF|NKSF2	5	Antimicrobials
TLR8	51311	toll like receptor 8	CD288	X	Antimicrobials
GNLY	10578	granulysin	D2S69E|LAG-2|LAG2|NKG5|TLA519	2	Antimicrobials
CD81	975	CD81 molecule	CVID6|S5.7|TAPA1|TSPAN28	11	Antimicrobials
EIF2AK2	5610	eukaryotic translation initiation factor 2 alpha kinase 2	EIF2AK1|LEUDEN|PKR|PPP1R83|PRKR	2	Antimicrobials
APOM	55937	apolipoprotein M	G3a|HSPC336|NG20|apo-M	6	Antimicrobials
CACYBP	27101	calcyclin binding protein	GIG5|PNAS-107|S100A6BP|SIP	1	Antimicrobials
NOD1	10392	nucleotide binding oligomerization domain containing 1	CARD4|CLR7.1|NLRC1	7	Antimicrobials
MAPK8	5599	mitogen-activated protein kinase 8	JNK|JNK-46|JNK1|JNK1A2|JNK21B1/2|PRKM8|SAPK1|SAPK1c	10	Antimicrobials
MAPK3	5595	mitogen-activated protein kinase 3	ERK-1|ERK1|ERT2|HS44KDAP|HUMKER1A|P44ERK1|P44MAPK|PRKM3|p44-ERK1|p44-MAPK	16	Antimicrobials
BST2	684	bone marrow stromal cell antigen 2	CD317|TETHERIN	19	Antimicrobials
BPHL	670	biphenyl hydrolase like	BPH-RP|MCNAA|VACVASE	6	Antimicrobials
PLA2G2A	5320	phospholipase A2 group IIA	MOM1|PLA2|PLA2B|PLA2L|PLA2S|PLAS1|sPLA2	1	Antimicrobials
GRN	2896	granulin precursor	CLN11|GEP|GP88|PCDGF|PEPI|PGRN	17	Antimicrobials

NEWENTRY	192343	-	-	-	Antimicrobials
PDGFRA	5156	platelet derived growth factor receptor alpha	CD140A|PDGFR-2|PDGFR2	4	Antimicrobials
GNAI1	2770	G protein subunit alpha i1	Gi	7	Antimicrobials
WNT5A	7474	Wnt family member 5A	hWNT5A	3	Antimicrobials
FURIN	5045	furin, paired basic amino acid cleaving enzyme	FUR|PACE|PCSK3|SPC1	15	Antimicrobials
ADAR	103	adenosine deaminase RNA specific	ADAR1|AGS6|DRADA|DSH|DSRAD|G1P1|IFI-4|IFI4|K88DSRBP|P136	1	Antimicrobials
TYK2	7297	tyrosine kinase 2	IMD35|JTK1	19	Antimicrobials
NOS2	4843	nitric oxide synthase 2	HEP-NOS|INOS|NOS|NOS2A	17	Antimicrobials
TRAF3	7187	TNF receptor associated factor 3	CAP-1|CAP1|CD40bp|CRAF1|IIAE5|LAP1|RNF118	14	Antimicrobials
TPT1	7178	tumor protein, translationally-controlled 1	HRF|TCTP|p02|p23	13	Antimicrobials
TPM2	7169	tropomyosin 2	AMCD1|DA1|DA2B|DA2B4|HEL-S-273|NEM4|TMSB	9	Antimicrobials
NEO1	4756	neogenin 1	IGDCC2|NGN|NTN1R2	15	Antimicrobials
AHNAK	79026	AHNAK nucleoprotein	AHNAKRS|PM227	11	Antimicrobials
TLR1	7096	toll like receptor 1	CD281|TIL|TIL. LPRS5|rsc786	4	Antimicrobials
TK2	7084	thymidine kinase 2	MTDPS2|MTTK|PEOB3|SCA31	16	Antimicrobials
PRDX2	7001	peroxiredoxin 2	HEL-S-2a|NKEF-B|NKEFB|PRP|PRX2|PRXII|PTX1|TDPX1|TPX1|TSA	19	Antimicrobials
MX2	4600	MX dynamin like GTPase 2	MXB	21	Antimicrobials
FGF2	2247	fibroblast growth factor 2	BFGF|FGF-2|FGFB|HBGF-2	4	Antimicrobials
FGA	2243	fibrinogen alpha chain	Fib2	4	Antimicrobials

TCF7L2	6934	transcription factor 7 like 2	TCF-4|TCF4	10	Antimicrobials
F2RL1	2150	F2R like trypsin receptor 1	GPR11|PAR2	5	Antimicrobials
TKFC	26007	triokinase and FMN cyclase	DAK|NET45|TKFCD	11	Antimicrobials
MSR1	4481	macrophage scavenger receptor 1	CD204|SCARA1|SR-A|SR-AI|SR-AII|SR-AIII|SRA|phSR1|phSR2	8	Antimicrobials
NFKBIZ	64332	NFKB inhibitor zeta	IKBZ|INAP|MAIL	3	Antimicrobials
LMBR1	64327	limb development membrane protein 1	ACHP|C7orf2|DIF14|LSS|PPD2|THYP|TPT|ZRS	7	Antimicrobials
EPPIN	57119	epididymal peptidase inhibitor	CT71|CT72|SPINLW1|WAP7|WFDC7|dJ461P17.2	20	Antimicrobials
SRC	6714	SRC proto-oncogene, non-receptor tyrosine kinase	ASV|SRC1|THC6|c-SRC|p60-Src	20	Antimicrobials
MPO	4353	myeloperoxidase	-	17	Antimicrobials
ELAVL1	1994	ELAV like RNA binding protein 1	ELAV1|HUR|Hua|MelG	19	Antimicrobials
ROBO3	64221	roundabout guidance receptor 3	HGPPS|HGPPS1|HGPS|RBIG1|RIG1	11	Antimicrobials
SP1	6667	Sp1 transcription factor	-	12	Antimicrobials
SOD1	6647	superoxide dismutase 1	ALS|ALS1|HEL-S-44|IPOA|SOD|STAHP|hSod1|homodimer	21	Antimicrobials
PDF	64146	peptide deformylase, mitochondrial	-	16	Antimicrobials
DLL4	54567	delta like canonical Notch ligand 4	AOS6|delta4|hdelta2	15	Antimicrobials

	7				
ECD	11319	ecdysoneless cell cycle regulator	GCR2|HSGT1|SGT1	10	Antimicrobials
SLC11A1	6556	solute carrier family 11 member 1	LSH|NRAMP|NRAMP1	2	Antimicrobials
DMBT1	1755	deleted in malignant brain tumors 1	GP340|SAG|SALSA|muclin	10	Antimicrobials
STING1	340061	stimulator of interferon response cGAMP interactor 1	ERIS|MITA|MPYS|NET23|SAVI|STING|STING-beta|TMEM173|hMITA|hSTING	5	Antimicrobials
SKIV2L	6499	Ski2 like RNA helicase	170A|DDX13|HLP|SKI2|SKI2W|SKIV2|SKIV2L1|THES2	6	Antimicrobials
SEMG2	6407	semenogelin 2	SGII	20	Antimicrobials
LTA	4049	lymphotoxin alpha	LT|TNFB|TNFSF1|TNLG1E	6	Antimicrobials
DES	1674	desmin	CDCD3|CSM1|CSM2|LGMD1D|LGMD1E|LGMD2R	2	Antimicrobials
DCK	1633	deoxycytidine kinase	-	4	Antimicrobials
DAXX	1616	death domain associated protein	BING2|DAP6|EAP1|SMIM40	6	Antimicrobials
TNFRSF10A	8797	TNF receptor superfamily member 10a	APO2|CD261|DR4|TRAILR-1|TRAILR1	8	Antimicrobials
TNFRSF10B	8795	TNF receptor superfamily member 10b	CD262|DR5|KILLER|KILLER/DR5|TRAIL-R2|TRAILR2|TRICK2|TRICK2A|TRICK2B|TRICKB|ZTNFR9	8	Antimicrobials
EED	8726	embryonic ectoderm development	COGIS|HEED|WAIT1	11	Antimicrobials
CCL4	6351	C-C motif chemokine ligand 4	ACT2|AT744.1|G-26|HC21|LAG-1|LAG1|MIP-1-beta|MIP1B|MIP1B1|SCYA2|SCYA4	17	Antimicrobials
LIMS1	3987	LIM zinc finger domain containing 1	PINCH|PINCH-1|PINCH1	2	Antimicrobials
LALBA	3906	lactalbumin alpha	LYZG	12	Antimicrobials
APOBE	1646	apolipoprotein B mRNA editing enzyme catalytic	A3H|ARP-10|ARP10	22	Antimicrobials

C3H	68	subunit 3H			
TMPRSS6	164656	transmembrane serine protease 6	IRIDA|MT2	22	Antimicrobials
SPINK5	11005	serine peptidase inhibitor Kazal type 5	LEKTI|LETKI|NETS|NS|VAKTI	5	Antimicrobials
MARCO	8685	macrophage receptor with collagenous structure	SCARA2|SR-A6	2	Antimicrobials
BECN1	8678	beclin 1	ATG6|VPS30|beclin1	17	Antimicrobials
TNFSF11	8600	TNF superfamily member 11	CD254|ODF|OPGL|OPTB2|RANKL|TNLG6B|TRANCE|hRANKL2|sOdf	13	Antimicrobials
KNG1	3827	kininogen 1	BDK|BK|HMWK|KNG	3	Antimicrobials
CSK	1445	C-terminal Src kinase	-	15	Antimicrobials
KLRK1	22914	killer cell lectin like receptor K1	CD314|D12S2489E|KLR|NKG2-D|NKG2D	12	Antimicrobials
KCNH2	3757	potassium voltage-gated channel subfamily H member 2	ERG-1|ERG1|H-ERG|HERG|HERG1|Kv11.1|LQT2|SQT1	7	Antimicrobials
JUND	3727	JunD proto-oncogene, AP-1 transcription factor subunit	AP-1	19	Antimicrobials
JAK1	3716	Janus kinase 1	JAK1A|JAK1B|JTK3	1	Antimicrobials
CREB1	1385	cAMP responsive element binding protein 1	CREB|CREB-1	2	Antimicrobials
CLDN4	1364	claudin 4	CPE-R|CPER|CPETR|CPETR1|WBSCR8|hCPE-R	7	Antimicrobials
CCL28	56477	C-C motif chemokine ligand 28	CCK1|MEC|SCYA28	5	Antimicrobials
RNASE3	6037	ribonuclease A family member 3	ECP|RAF1|RNS3	14	Antimicrobials
RN7SL	6029	RNA component of signal recognition particle	7L1a|7SL|RN7SL|RNSRP1	14	Antimicrobials

1		7SL1			
IRF7	3665	interferon regulatory factor 7	IMD39|IRF-7|IRF-7H|IRF7A|IRF7B|IRF7C |IRF7H	11	Antimicrobials
IREB2	3658	iron responsive element binding protein 2	ACO3|IRE-BP 2|IRE-BP2|IRP2|IRP2AD|NDCAMA	15	Antimicrobials
ILK	3611	integrin linked kinase	HEL-S-28|ILK-1|ILK-2|P59|p59ILK	11	Antimicrobials
IL18	3606	interleukin 18	IGIF|IL-18|IL-1g|IL1F4	11	Antimicrobials
IL17A	3605	interleukin 17A	CTLA-8|CTLA8|IL-17|IL-17A|IL17	6	Antimicrobials
LTB4R	1241	leukotriene B4 receptor	BLT1|BLTR|CMKRL1|GPR16|LTB4R1|LTBR1| P2RY7|P2Y7	14	Antimicrobials
APOBE C3A	2003 15	apolipoprotein B mRNA editing enzyme catalytic subunit 3A	A3A|ARP3|PHRBN|bK150C2.1	22	Antimicrobials
MASP2	1074 7	mannan binding lectin serine peptidase 2	MAP19|MASP-2|MASP1P1|sMAP	1	Antimicrobials
TRIM2 7	5987	tripartite motif containing 27	RFP|RNF76	6	Antimicrobials
RELA	5970	RELA proto-oncogene, NF-kB subunit	CMCU|NFKB3|p65	11	Antimicrobials
IL7R	3575	interleukin 7 receptor	CD127|CDW127|IL-7R-alpha|IL7RA|ILRA	5	Antimicrobials
IL1A	3552	interleukin 1 alpha	IL-1 alpha|IL-1A|IL1|IL1-ALPHA|IL1F1	2	Antimicrobials
PTX3	5806	pentraxin 3	TNFAIP5|TSG-14	3	Antimicrobials
IFNAR 2	3455	interferon alpha and beta receptor subunit 2	IFN-R|IFN-alpha-REC|IFNABR|IFNARB|IM D45	21	Antimicrobials
IFN1@	3438	-	IFNA	9	Antimicrobials
SYTL1	8495 8	synaptotagmin like 1	JFC1|SLP1	1	Antimicrobials
APOBE	2735	apolipoprotein B mRNA editing enzyme catalytic	A3C|APOBEC1L|ARDC2|ARDC4|ARP5|PBI|bK	22	Antimicrobials

C3C	0	subunit 3C	150C2.3			
DDX17	1052<br>1	DEAD-box helicase 17	P72|RH70		22	Antimicrobials
PTGS2	5743	prostaglandin-endoperoxide synthase 2	COX-2|COX2|GRIPGHS|PGG/HS|PGHS-2|PHS<br>-2|hCox-2		1	Antimicrobials
HTR1A	3350	5-hydroxytryptamine receptor 1A	5-HT-1A|5-HT1A|5HT1a|ADRB2RL1|ADRBRL<br>1|G-21|PFMCD		5	Antimicrobials
SEPTI<br>N7	989	septin 7	CDC10|CDC3|NBLA02942|SEPT7|SEPT7A		7	Antimicrobials
CD40L<br>G	959	CD40 ligand	CD154|CD40L|HIGM1|IGM|IMD3|T-BAM|TNF<br>SF5|TRAP|gp39|hCD40L	X		Antimicrobials
CD14	929	CD14 molecule	-		5	Antimicrobials
CD8A	925	CD8a molecule	CD8|Leu2|p32		2	Antimicrobials
CD4	920	CD4 molecule	CD4mut		12	Antimicrobials
MASP1	5648	mannan binding lectin serine peptidase 1	3MC1|CRARF|CRARF1|MAP1|MASP|MASP3|MA<br>p44|PRSS5|RaRF		3	Antimicrobials
PROC	5624	protein C, inactivator of coagulation factors<br>Va and VIIIa	APC|PC|PROC1|THPH3|THPH4		2	Antimicrobials
MAP2K<br>2	5605	mitogen-activated protein kinase kinase 2	CFC4|MAPKK2|MEK2|MKK2|PRKMK2		19	Antimicrobials
MAP2K<br>1	5604	mitogen-activated protein kinase kinase 1	CFC3|MAPKK1|MEK1|MKK1|PRKMK1		15	Antimicrobials
HRG	3273	histidine rich glycoprotein	HPRG|HRGP|THPH11		3	Antimicrobials
NDRG1	1039<br>7	N-myc downstream regulated 1	CAP43|CMT4D|DRG-1|DRG1|GC4|HMSNL|NDR<br>1|NMSL|PROXY1|RIT42|RTP|TARG1|TDD5		8	Antimicrobials
IRF9	1037	interferon regulatory factor 9	IRF-9|ISGF3|ISGF3G|p48		14	Antimicrobials

	9				
TRIM22	10346	tripartite motif containing 22	GPSTAF50|RNF94|STAF50	11	Antimicrobials
LANCL1	10314	LanC like 1	GPR69A|p40	2	Antimicrobials
PPP4C	5531	protein phosphatase 4 catalytic subunit	PP-X|PP4|PP4C|PPH3|PPP4|PPX	16	Antimicrobials
HMOX1	3162	heme oxygenase 1	HMOX1D|HO-1|HSP32|bK286B10	22	Antimicrobials
HMGB1	3146	high mobility group box 1	HMG-1|HMG1|HMG3|SBP-1	13	Antimicrobials
HLA-B	3106	major histocompatibility complex, class I, B	AS|B-4901|HLAB	6	Antimicrobials
RNASE7	84659	ribonuclease A family member 7	RAE1	14	Antimicrobials
ABCC4	10257	ATP binding cassette subfamily C member 4	MOAT-B|MOATB|MRP4	13	Antimicrobials
HGF	3082	hepatocyte growth factor	DFNB39|F-TCF|HGFB|HPTA|SF	7	Antimicrobials
HDAC1	3065	histone deacetylase 1	GON-10|HD1|KDAC1|RPD3|RPD3L1	1	Antimicrobials
IFNLR1	163702	interferon lambda receptor 1	CRF2/12|IFNLR|IL-28R1|IL28RA|LICR2	1	Antimicrobials
PLSCR1	5359	phospholipid scramblase 1	MMTRA1B	3	Antimicrobials
B2M	567	beta-2-microglobulin	IMD43	15	Antimicrobials
BACH2	60468	BTB domain and CNC homolog 2	BTBD25|IMD60	6	Antimicrobials
TANK	10010	TRAF family member associated NFKB activator	I-TRAF|ITRAF|TRAF2	2	Antimicrobials
PIK3CG	5294	phosphatidylinositol-4,5-bisphosphate 3-kinase catalytic subunit gamma	PI3CG|PI3K|PI3Kgamma|PIK3|p110gamma|p120-PI3K	7	Antimicrobials

<table>
<tr><td>ARRB1</td><td>408</td><td>arrestin beta 1</td><td>ARB1|ARR1</td><td>11</td><td>Antimicrobials</td></tr>
<tr><td>RSAD2</td><td>91543</td><td>radical S-adenosyl methionine domain containing 2</td><td>2510004L01Rik|cig33|cig5|vig1</td><td>2</td><td>Antimicrobials</td></tr>
<tr><td>STAB2</td><td>55576</td><td>stabilin 2</td><td>FEEL2|FELE-2|FELL2|FEX2|HARE|SCARH1</td><td>12</td><td>Antimicrobials</td></tr>
<tr><td>TBK1</td><td>29110</td><td>TANK binding kinase 1</td><td>FTDALS4|IIAE8|NAK|T2K</td><td>12</td><td>Antimicrobials</td></tr>
<tr><td>PDYN</td><td>5173</td><td>prodynorphin</td><td>ADCA|PENKB|SCA23</td><td>20</td><td>Antimicrobials</td></tr>
<tr><td>PDGFRB</td><td>5159</td><td>platelet derived growth factor receptor beta</td><td>CD140B|IBGC4|IMF1|JTK12|KOGS|PDGFR|PDGFR-1|PDGFR1|PENTT</td><td>5</td><td>Antimicrobials</td></tr>
<tr><td>PDCD1</td><td>5133</td><td>programmed cell death 1</td><td>CD279|PD-1|PD1|SLEB2|hPD-1|hPD-l|hSLE1</td><td>2</td><td>Antimicrobials</td></tr>
<tr><td>PCSK2</td><td>5126</td><td>proprotein convertase subtilisin/kexin type 2</td><td>NEC 2|NEC-2|NEC2|PC2|SPC2</td><td>20</td><td>Antimicrobials</td></tr>
<tr><td>PCSK1</td><td>5122</td><td>proprotein convertase subtilisin/kexin type 1</td><td>BMIQ12|NEC1|PC1|PC3|SPC3</td><td>5</td><td>Antimicrobials</td></tr>
<tr><td>ARG2</td><td>384</td><td>arginase 2</td><td>-</td><td>14</td><td>Antimicrobials</td></tr>
<tr><td>AQP9</td><td>366</td><td>aquaporin 9</td><td>AQP-9|HsT17287|SSC1|T17287</td><td>15</td><td>Antimicrobials</td></tr>
<tr><td>FASLG</td><td>356</td><td>Fas ligand</td><td>ALPS1B|APT1LG1|APTL|CD178|CD95-L|CD95L|FASL|TNFSF6|TNLG1A</td><td>1</td><td>Antimicrobials</td></tr>
<tr><td>APOH</td><td>350</td><td>apolipoprotein H</td><td>B2G1|B2GP1|BG</td><td>17</td><td>Antimicrobials</td></tr>
<tr><td>BIRC5</td><td>332</td><td>baculoviral IAP repeat containing 5</td><td>API4|EPR-1</td><td>17</td><td>Antimicrobials</td></tr>
<tr><td>ANXA6</td><td>309</td><td>annexin A6</td><td>ANX6|CBP68|CPB-II|p68|p70</td><td>5</td><td>Antimicrobials</td></tr>
<tr><td>IL22</td><td>50616</td><td>interleukin 22</td><td>IL-21|IL-22|IL-D110|IL-TIF|ILTIF|TIFIL-23|TIFa|zcyto18</td><td>12</td><td>Antimicrobials</td></tr>
<tr><td>VTN</td><td>7448</td><td>vitronectin</td><td>V75|VN|VNT</td><td>17</td><td>Antimicrobials</td></tr>
<tr><td>VIM</td><td>7431</td><td>vimentin</td><td>-</td><td>10</td><td>Antimicrobials</td></tr>
<tr><td>VCAM1</td><td>7412</td><td>vascular cell adhesion molecule 1</td><td>CD106|INCAM-100</td><td>1</td><td>Antimicrobials</td></tr>
</table>

PRDX1	5052	peroxiredoxin 1	MSP23|NKEF-A|NKEFA|PAG|PAGA|PAGB|PRX1|PRXI|TDPX2	1	Antimicrobials
GFAP	2670	glial fibrillary acidic protein	ALXDRD	17	Antimicrobials
GBP2	2634	guanylate binding protein 2	-	1	Antimicrobials
ALB	213	albumin	HSA|PRO0883|PRO0903|PRO1341	4	Antimicrobials
SLC29A3	55315	solute carrier family 29 member 3	ENT3|HCLAP|HJCD|PHID	10	Antimicrobials
OAS1	4938	2'-5'-oligoadenylate synthetase 1	E18/E16|IFI-4|OIAS|OIASI	12	Antimicrobials
AGER	177	advanced glycosylation end-product specific receptor	RAGE|SCARJ1	6	Antimicrobials
UNC93B1	81622	unc-93 homolog B1, TLR signaling regulator	IIAE1|UNC93|UNC93B|Unc-93B1	11	Antimicrobials
TNFSF4	7292	TNF superfamily member 4	CD134L|CD252|GP34|OX-40L|OX4OL|TNLG2B|TXGP1	1	Antimicrobials
NOS1	4842	nitric oxide synthase 1	IHPS1|N-NOS|NC-NOS|NOS|bNOS|nNOS	12	Antimicrobials
ACTG1	71	actin gamma 1	ACT|ACTG|DFNA20|DFNA26|HEL-176	17	Antimicrobials
ACTA1	58	actin alpha 1, skeletal muscle	ACTA|ASMA|CFTD|CFTD1|CFTDM|MPFD|NEM1|NEM2|NEM3|SHPM	1	Antimicrobials
ACO1	48	aconitase 1	ACONS|HEL60|IREB1|IREBP|IREBP1|IRP1	9	Antimicrobials
SERPINA3	12	serpin family A member 3	AACT|ACT|GIG24|GIG25	14	Antimicrobials
CXCR1	3577	C-X-C motif chemokine receptor 1	C-C|C-C-CKR-1|CD128|CD181|CDw128a|CKR-1|CMKAR1|IL8R1|IL8RA|IL8RBA	2	Antimicrobials
CCL15	6359	C-C motif chemokine ligand 15	HCC-2|HMRP-2B|LKN-1|LKN1|MIP-1 delta|MIP-1D|MIP-5|MRP-2B|NCC-3|NCC3|SCYA15|SCYL3|SY15	17	Antimicrobials

CCL14	6358	C-C motif chemokine ligand 14	CC-1|CC-3|CKB1|HCC-1|HCC-1(1-74)|HCC-1/HCC-3|HCC-3|MCIF|NCC-2|NCC2|SCYA14|SCYL2|SY14	17	Antimicrobials
CCL4	6351	C-C motif chemokine ligand 4	ACT2|AT744.1|G-26|HC21|LAG-1|LAG1|MIP-1-beta|MIP1B|MIP1B1|SCYA2|SCYA4	17	Antimicrobials
CCL16	6360	C-C motif chemokine ligand 16	CKb12|HCC-4|ILINCK|LCC-1|LEC|LMC|Mtn-1|NCC-4|NCC4|SCYA16|SCYL4	17	Antimicrobials
CCL19	6363	C-C motif chemokine ligand 19	CKb11|ELC|MIP-3b|MIP3B|SCYA19	9	Antimicrobials
CCL13	6357	C-C motif chemokine ligand 13	CKb10|MCP-4|NCC-1|NCC1|SCYA13|SCYL1	17	Antimicrobials
CCL18	6362	C-C motif chemokine ligand 18	AMAC-1|AMAC1|CKb7|DC-CK1|DCCK1|MIP-4|PARC|SCYA18	17	Antimicrobials
CCL17	6361	C-C motif chemokine ligand 17	A-152E5.3|ABCD-2|SCYA17|TARC	16	Antimicrobials
CCL26	10344	C-C motif chemokine ligand 26	IMAC|MIP-4a|MIP-4alpha|SCYA26|TSC-1	7	Antimicrobials
CCL22	6367	C-C motif chemokine ligand 22	A-152E5.1|ABCD-1|DC/B-CK|MDC|SCYA22|STCP-1	16	Antimicrobials
CCR3	1232	C-C motif chemokine receptor 3	C C CKR3|CC-CKR-3|CD193|CKR 3|CKR3|CMKBR3	3	Antimicrobials
CCL28	56477	C-C motif chemokine ligand 28	CCK1|MEC|SCYA28	5	Antimicrobials
CCL4L1	388372	C-C motif chemokine ligand 4 like 1	AT744.2|CCL4L|LAG-1|LAG1|MIP-1-beta|SCYA4L|SCYA4L1|SCYA4L2	17	Antimicrobials
ACKR2	1238	atypical chemokine receptor 2	CCBP2|CCR10|CCR9|CMKBR9|D6|hD6	3	Antimicrobials
CCR7	1236	C-C motif chemokine receptor 7	BLR2|CC-CKR-7|CCR-7|CD197|CDw197|CMKBR7|EBI1	17	Antimicrobials
CCL27	1085	C-C motif chemokine ligand 27	ALP|CTACK|CTAK|ESKINE|ILC|PESKY|SCYA	9	Antimicrobials

	0		27		
CCR8	1237	C-C motif chemokine receptor 8	CC-CKR-8|CCR-8|CDw198|CKRL1|CMKBR8|CMKBRL2|CY6|GPRCY6|TER1	3	Antimicrobials
ACKR4	51554	atypical chemokine receptor 4	CC-CKR-11|CCBP2|CCR-11|CCR10|CCR11|CCRL1|CCX CKR|CCX-CKR|CKR-11|PPR1|VSHK1	3	Antimicrobials
CCR10	2826	C-C motif chemokine receptor 10	GPR2	17	Antimicrobials
CCL2	6347	C-C motif chemokine ligand 2	GDCF-2|HC11|HSMCR30|MCAF|MCP-1|MCP1|SCYA2|SMC-CF	17	Antimicrobials
CCL21	6366	C-C motif chemokine ligand 21	6Ckine|CKb9|ECL|SCYA21|SLC|TCA4	9	Antimicrobials
CCL7	6354	C-C motif chemokine ligand 7	FIC|MARC|MCP-3|MCP3|NC28|SCYA6|SCYA7	17	Antimicrobials
CCL5	6352	C-C motif chemokine ligand 5	D17S136E|RANTES|SCYA5|SIS-delta|SISd|TCP228|eoCP	17	Antimicrobials
CCL3	6348	C-C motif chemokine ligand 3	G0S19-1|LD78ALPHA|MIP-1-alpha|MIP1A|SCYA3	17	Antimicrobials
CCL20	6364	C-C motif chemokine ligand 20	CKb4|Exodus|LARC|MIP-3-alpha|MIP-3a|MIP3A|SCYA20|ST38	2	Antimicrobials
CCL11	6356	C-C motif chemokine ligand 11	SCYA11	17	Antimicrobials
CCR5	1234	C-C motif chemokine receptor 5	CC-CKR-5|CCCKR5|CCR-5|CD195|CKR-5|CKR5|CMKBR5|IDDM22	3	Antimicrobials
CCL23	6368	C-C motif chemokine ligand 23	CK-BETA-8|CKb8|Ckb-8|Ckb-8-1|MIP-3|MIP3|MPIF-1|SCYA23|hmrp-2a	17	Antimicrobials
CCL25	6370	C-C motif chemokine ligand 25	Ckb15|SCYA25|TECK	19	Antimicrobials
CCL1	6346	C-C motif chemokine ligand 1	I-309|P500|SCYA1|SISe|TCA3	17	Antimicrobials
CCL3L3	414062	C-C motif chemokine ligand 3 like 3	464.2|D17S1718|G0S19-2|LD78|LD78BETA|SCYA3L|SCYA3L1	17	Antimicrobials

CCL4L2	9560	C-C motif chemokine ligand 4 like 2	AT744.2|CCL4L|SCYA4L|SCYQ4L2	17	Antimicrobials
CXCL12	6387	C-X-C motif chemokine ligand 12	IRH|PBSF|SCYB12|SDF1|TLSF|TPAR1	10	Antimicrobials
XCL1	6375	X-C motif chemokine ligand 1	ATAC|LPTN|LTN|SCM-1|SCM-1a|SCM1|SCM1A|SCYC1	1	Antimicrobials
CCL8	6355	C-C motif chemokine ligand 8	HC14|MCP-2|MCP2|SCYA10|SCYA8	17	Antimicrobials
CCL3L1	6349	C-C motif chemokine ligand 3 like 1	464.2|D17S1718|G0S19-2|LD78|LD78-beta(1-70)|LD78BETA|MIP1AP|SCYA3L|SCYA3L1	17	Antimicrobials
CCR1	1230	C-C motif chemokine receptor 1	CD191|CKR-1|CKR1|CMKBR1|HM145|MIP1aR|SCYAR1	3	Antimicrobials
CCL24	6369	C-C motif chemokine ligand 24	Ckb-6|MPIF-2|MPIF2|SCYA24	7	Antimicrobials
XCL2	6846	X-C motif chemokine ligand 2	SCM-1b|SCM1B|SCYC2	1	Antimicrobials
CXCL1	2919	C-X-C motif chemokine ligand 1	FSP|GRO1|GROa|MGSA|MGSA-a|NAP-3|SCYB1	4	Antimicrobials
CXCL10	3627	C-X-C motif chemokine ligand 10	C7|IFI10|INP10|IP-10|SCYB10|crg-2|gIP-10|mob-1	4	Antimicrobials
CXCR4	7852	C-X-C motif chemokine receptor 4	CD184|D2S201E|FB22|HM89|HSY3RR|LAP-3|LAP3|LCR1|LESTR|NPY3R|NPYR|NPYRL|NPYY3R|WHIM|WHIMS	2	Antimicrobials
CXCL2	2920	C-X-C motif chemokine ligand 2	CINC-2a|GRO2|GROb|MGSA-b|MIP-2a|MIP2|MIP2A|SCYB2	4	Antimicrobials
CXCR6	10663	C-X-C motif chemokine receptor 6	BONZO|CD186|STRL33|TYMSTR	3	Antimicrobials
CCR4	1233	C-C motif chemokine receptor 4	CC-CKR-4|CD194|CKR4|CMKBR4|ChemR13|H	3	Antimicrobials

CXCL11	6373	C-X-C motif chemokine ligand 11	GCN:14099|K5-5 H174|I-TAC|IP-9|IP9|SCYB11|SCYB9B|b-R1	4	Antimicrobials
TAFA5	25817	TAFA chemokine like family member 5	FAM19A5|QLLK5208|TAFA-5|UNQ5208	22	Antimicrobials
TAFA3	284467	TAFA chemokine like family member 3	FAM19A3|TAFA-3	1	Antimicrobials
TAFA4	151647	TAFA chemokine like family member 4	FAM19A4|TAFA-4	3	Antimicrobials
TAFA1	407738	TAFA chemokine like family member 1	FAM19A1|TAFA-1	3	Antimicrobials
TAFA2	338811	TAFA chemokine like family member 2	FAM19A2|TAFA-2	12	Antimicrobials
CCL15-CCL14	348249	CCL15-CCL14 readthrough (NMD candidate)	CCL15|HCC-2|LKN-1|MIP-5|MIP5|Mrp-2b|NCC-3|NCC3|SCYA15	17	Antimicrobials
IL6	3569	interleukin 6	BSF-2|BSF2|CDF|HGF|HSF|IFN-beta-2|IFNB2|IL-6	7	Antimicrobials
TNF	7124	tumor necrosis factor	DIF|TNF-alpha|TNFA|TNFSF2|TNLG1F	6	Antimicrobials
IL1B	3553	interleukin 1 beta	IL-1|IL1-BETA|IL1F2|IL1beta	2	Antimicrobials
IL18	3606	interleukin 18	IGIF|IL-18|IL-1g|IL1F4	11	Antimicrobials
PTK2B	2185	protein tyrosine kinase 2 beta	CADTK|CAKB|FADK2|FAK2|PKB|PTK|PYK2|RAFTK	8	Antimicrobials
VEGFA	7422	vascular endothelial growth factor A	MVCD1|VEGF|VPF	6	Antimicrobials
IL4	3565	interleukin 4	BCGF-1|BCGF1|BSF-1|BSF1|IL-4	5	Antimicrobials
CDH1	999	cadherin 1	Arc-1|BCDS1|CD324|CDHE|ECAD|LCAM|UVO	16	Antimicrobials

CD40	958	CD40 molecule	Bp50|CDW40|TNFRSF5|p50	20	Antimicrobials
DEFB103B	55894	defensin beta 103B	BD-3|DEFB-3|DEFB103|DEFB3|HBD-3|HBD3|HBP-3|HBP3	8	Antimicrobials
F2RL1	2150	F2R like trypsin receptor 1	GPR11|PAR2	5	Antimicrobials
MMP9	4318	matrix metallopeptidase 9	CLG4B|GELB|MANDP2|MMP-9	20	Antimicrobials
LTBP1	4052	latent transforming growth factor beta binding protein 1	-	2	Antimicrobials
DEFB4A	1673	defensin beta 4A	BD-2|DEFB-2|DEFB102|DEFB2|DEFB4|HBD-2|SAP1	8	Antimicrobials
TNFSF10	8743	TNF superfamily member 10	APO2L|Apo-2L|CD253|TL2|TNLG6A|TRAIL	3	Antimicrobials
IL13	3596	interleukin 13	IL-13|P600	5	Antimicrobials
IL10	3586	interleukin 10	CSIF|GVHDS|IL-10|IL10A|TGIF	1	Antimicrobials
IL2	3558	interleukin 2	IL-2|TCGF|lymphokine	4	Antimicrobials
PPARG	5468	peroxisome proliferator activated receptor gamma	CIMT1|GLM1|NR1C3|PPARG1|PPARG2|PPARG5|PPARgamma	3	Antimicrobials
FGR	2268	FGR proto-oncogene, Src family tyrosine kinase	SRC2|c-fgr|c-src2|p55-Fgr|p55c-fgr|p58-Fgr|p58c-fgr	1	Antimicrobials
MIF	4282	macrophage migration inhibitory factor	GIF|GLIF|MMIF	22	Antimicrobials
CRP	1401	C-reactive protein	PTX1	1	Antimicrobials
JAK2	3717	Janus kinase 2	JTK10|THCYT3	9	Antimicrobials
IL1A	3552	interleukin 1 alpha	IL-1 alpha|IL-1A|IL1|IL1-ALPHA|IL1F1	2	Antimicrobials
PTK2	5747	protein tyrosine kinase 2	FADK|FAK|FAK1|FRNK|PPP1R71|p125FAK|pp125FAK	8	Antimicrobials
PTGDR	5729	prostaglandin D2 receptor	AS1|ASRT1|DP|DP1|PTGDR1	14	Antimicrobials
CD86	942	CD86 molecule	B7-2|B7.2|B70|CD28LG2|LAB72	3	Antimicrobials

HCK	3055	HCK proto-oncogene, Src family tyrosine kinase	JTK9|p59Hck|p61Hck	20	Antimicrobials
ARRB1	408	arrestin beta 1	ARB1|ARR1	11	Antimicrobials
GNAI1	2770	G protein subunit alpha i1	Gi	7	Antimicrobials
VDR	7421	vitamin D receptor	NR1I1|PPP1R163	12	Antimicrobials
OLR1	4973	oxidized low density lipoprotein receptor 1	CLEC8A|LOX1|LOXIN|SCARE1|SLOX1	12	Antimicrobials
GRK2	156	G protein-coupled receptor kinase 2	ADRBK1|BARK1|BETA-ARK1	11	Antimicrobials
TXK	7294	TXK tyrosine kinase	BTKL|PSCTK5|PTK4|RLK|TKL	4	Antimicrobials
RNASE2	6036	ribonuclease A family member 2	EDN|RAF3|RNS2	14	Antimicrobials
CD79A	973	CD79a molecule	IGA|MB-1	19	BCRSignalingPathway
CD79B	974	CD79b molecule	AGM6|B29|IGB	17	BCRSignalingPathway
LYN	4067	LYN proto-oncogene, Src family tyrosine kinase	JTK8|p53Lyn|p56Lyn	8	BCRSignalingPathway
SYK	6850	spleen associated tyrosine kinase	p72-Syk	9	BCRSignalingPathway
BTK	695	Bruton tyrosine kinase	AGMX1|AT|ATK|BPK|IGHD3|IMD1|PSCTK1|XLA	X	BCRSignalingPathway
BLNK	29760	B cell linker	AGM4|BASH|BLNK-S|LY57|SLP-65|SLP65|bca	10	BCRSignalingPathway
VAV3	10451	vav guanine nucleotide exchange factor 3	-	1	BCRSignalingPathway
VAV1	7409	vav guanine nucleotide exchange factor 1	VAV	19	BCRSignalingPathway
VAV2	7410	vav guanine nucleotide exchange factor 2	VAV-2	9	BCRSignalingPathway
RAC1	5879	Rac family small GTPase 1	MIG5|MRD48|Rac-1|TC-25|p21-Rac1	7	BCRSignalingPathway
RAC2	5880	Rac family small GTPase 2	EN-7|Gx|HSPC022|p21-Rac2	22	BCRSignalingPathway
RAC3	5881	Rac family small GTPase 3	-	17	BCRSignalingPathway
PPP3CA	5530	protein phosphatase 3 catalytic subunit alpha	ACCIID|CALN|CALNA|CALNA1|CCN1|CNA1|IECEE|IECEE1|PPP2B	4	BCRSignalingPathway

PPP3CB	5532	protein phosphatase 3 catalytic subunit beta	CALNA2|CALNB|CNA2|PP2Bbeta	10	BCRSignalingPathway
PPP3CC	5533	protein phosphatase 3 catalytic subunit gamma	CALNA3|CNA3|PP2Bgamma	8	BCRSignalingPathway
CHP1	11261	calcineurin like EF-hand protein 1	CHP|SLC9A1BP|SPAX9|Sid470p|p22|p24	15	BCRSignalingPathway
PPP3R1	5534	protein phosphatase 3 regulatory subunit B, alpha	CALNB1|CNB|CNB1	2	BCRSignalingPathway
PPP3R2	5535	protein phosphatase 3 regulatory subunit B, beta	PPP3RL	9	BCRSignalingPathway
CHP2	63928	calcineurin like EF-hand protein 2	-	16	BCRSignalingPathway
NFAT5	10725	nuclear factor of activated T cells 5	NF-AT5|NFATL1|NFATZ|OREBP|TONEBP	16	BCRSignalingPathway
NFATC1	4772	nuclear factor of activated T cells 1	NF-ATC|NF-ATc1.2|NFAT2|NFATc	18	BCRSignalingPathway
NFATC2	4773	nuclear factor of activated T cells 2	NFAT1|NFATP	20	BCRSignalingPathway
NFATC3	4775	nuclear factor of activated T cells 3	NF-AT4c|NFAT4|NFATX	16	BCRSignalingPathway
NFATC4	4776	nuclear factor of activated T cells 4	NF-AT3|NF-ATC4|NFAT3	14	BCRSignalingPathway
HRAS	3265	HRas proto-oncogene, GTPase	C-BAS/HAS|C-H-RAS|C-HA-RAS1|CTLO|H-RASIDX|HAMSV|HRAS1|RASH1|p21ras	11	BCRSignalingPathway
KRAS	3845	KRAS proto-oncogene, GTPase	'C-K-RAS|C-K-RAS|CFC2|K-RAS2A|K-RAS2B|K-RAS4A|K-RAS4B|K-Ras|K-Ras	12	BCRSignalingPathway

			2|KI-RAS|KRAS1|KRAS2|NS|NS3|OES|RALD|RASK2|c-Ki-ras|c-Ki-ras2		
NRAS	4893	NRAS proto-oncogene, GTPase	ALPS4|CMNS|N-ras|NCMS|NRAS1|NS6	1	BCRSignalingPathway
FOS	2353	Fos proto-oncogene, AP-1 transcription factor subunit	AP-1|C-FOS|p55	14	BCRSignalingPathway
JUN	3725	Jun proto-oncogene, AP-1 transcription factor subunit	AP-1|AP1|c-Jun|cJUN|p39	1	BCRSignalingPathway
CARD11	84433	caspase recruitment domain family member 11	BENTA|BIMP3|CARMA1|IMD11|IMD11A|PPBL	7	BCRSignalingPathway
BCL10	8915	BCL10 immune signaling adaptor	CARMEN|CIPER|CLAP|IMD37|c-E10|mE10	1	BCRSignalingPathway
MALT1	10892	MALT1 paracaspase	IMD12|MLT|MLT1|PCASP1	18	BCRSignalingPathway
CHUK	1147	component of inhibitor of nuclear factor kappa B kinase complex	IKBKA|IKK-alpha|IKK1|IKKA|NFKBIKA|TCF16	10	BCRSignalingPathway
IKBKB	3551	inhibitor of nuclear factor kappa B kinase subunit beta	IKK-beta|IKK2|IKKB|IMD15|IMD15A|IMD15B|NFKBIKB	8	BCRSignalingPathway
IKBKG	8517	inhibitor of nuclear factor kappa B kinase regulatory subunit gamma	AMCBX1|EDAID1|FIP-3|FIP3|Fip3p|IKK-gamma|IKKAP1|IKKG|IMD33|IP|IP1|IP2|IPD2|NEMO|ZC2HC9	X	BCRSignalingPathway
NFKB1	4790	nuclear factor kappa B subunit 1	CVID12|EBP-1|KBF1|NF-kB|NF-kB1|NF-kappa-B1|NF-kappaB|NF-kappabeta|NFKB-p105|NFKB-p50|NFkappaB	4	BCRSignalingPathway
RELA	5970	RELA proto-oncogene, NF-kB subunit	CMCU|NFKB3|p65	11	BCRSignalingPathway
NFKBIA	4792	NFKB inhibitor alpha	EDAID2|IKBA|MAD-3|NFKBI	14	BCRSignalingPathway
NFKBI	4793	NFKB inhibitor beta	IKBB|TRIP9	19	BCRSignalingPathway

B					
NFKBIE	4794	NFKB inhibitor epsilon	IKBE	6	BCRSignalingPathway
CD81	975	CD81 molecule	CVID6|S5.7|TAPA1|TSPAN28	11	BCRSignalingPathway
CD19	930	CD19 molecule	B4|CVID3	16	BCRSignalingPathway
CR2	1380	complement C3d receptor 2	C3DR|CD21|CR|CVID7|SLEB9	1	BCRSignalingPathway
PIK3R5	23533	phosphoinositide-3-kinase regulatory subunit 5	F730038I15Rik|FOAP-2|P101-PI3K|p101	17	BCRSignalingPathway
PIK3R1	5295	phosphoinositide-3-kinase regulatory subunit 1	AGM7|GRB1|IMD36|p85|p85-ALPHA	5	BCRSignalingPathway
PIK3R2	5296	phosphoinositide-3-kinase regulatory subunit 2	MPPH|MPPH1|P85B|p85|p85-BETA	19	BCRSignalingPathway
PIK3R3	8503	phosphoinositide-3-kinase regulatory subunit 3	p55|p55-GAMMA|p55PIK	1	BCRSignalingPathway
PIK3CA	5290	phosphatidylinositol-4,5-bisphosphate 3-kinase catalytic subunit alpha	CLAPO|CLOVE|CWS5|MCAP|MCM|MCMTC|PI3K|PI3K-alpha|p110-alpha	3	BCRSignalingPathway
PIK3CB	5291	phosphatidylinositol-4,5-bisphosphate 3-kinase catalytic subunit beta	P110BETA|PI3K|PI3KBETA|PIK3C1	3	BCRSignalingPathway
PIK3CD	5293	phosphatidylinositol-4,5-bisphosphate 3-kinase catalytic subunit delta	APDS|IMD14|P110DELTA|PI3K|p110D	1	BCRSignalingPathway
PIK3CG	5294	phosphatidylinositol-4,5-bisphosphate 3-kinase catalytic subunit gamma	PI3CG|PI3K|PI3Kgamma|PIK3|p110gamma|p120-PI3K	7	BCRSignalingPathway
AKT3	10000	AKT serine/threonine kinase 3	MPPH|MPPH2|PKB-GAMMA|PKBG|PRKBG|RAC-PK-gamma|RAC-gamma|STK-2	1	BCRSignalingPathway
AKT1	207	AKT serine/threonine kinase 1	AKT|CWS6|PKB|PKB-ALPHA|PRKBA|RAC|RAC-ALPHA	14	BCRSignalingPathway

AKT2	208	AKT serine/threonine kinase 2	HIHGHH|PKBB|PKBBETA|PRKBB|RAC-BETA	19	BCRSignalingPathway
GSK3B	2932	glycogen synthase kinase 3 beta	-	3	BCRSignalingPathway
INPP5D	3635	inositol polyphosphate-5-phosphatase D	SHIP|SHIP-1|SHIP1|SIP-145|hp51CN|p150Ship	2	BCRSignalingPathway
CD22	933	CD22 molecule	SIGLEC-2|SIGLEC2	19	BCRSignalingPathway
CD72	971	CD72 molecule	CD72b|LYB2	9	BCRSignalingPathway
PTPN6	5777	protein tyrosine phosphatase non-receptor type 6	HCP|HCPH|HPTP1C|PTP-1C|SH-PTP1|SHP-1|SHP-1L|SHP1	12	BCRSignalingPathway
LILRB3	11025	leukocyte immunoglobulin like receptor B3	CD85A|HL9|ILT-5|ILT5|LILRA6|LIR-3|LIR3|PIR-B|PIRB	19	BCRSignalingPathway
FCGR2B	2213	Fc fragment of IgG receptor IIb	CD32|CD32B|FCG2|FCGR2|FCGR2C|FcRII-c|IGFR2	1	BCRSignalingPathway
RASGRP3	25780	RAS guanyl releasing protein 3	GRP3	2	BCRSignalingPathway
PLCG2	5336	phospholipase C gamma 2	APLAID|FCAS3|PLC-IV|PLC-gamma-2	16	BCRSignalingPathway
PRKCB	5579	protein kinase C beta	PKC-beta|PKCB|PKCI(2)|PKCbeta|PRKCB1|PRKCB2	16	BCRSignalingPathway
IFITM1	8519	interferon induced transmembrane protein 1	9-27|CD225|DSPA2a|IFI17|LEU13	11	BCRSignalingPathway
IGH	3492	immunoglobulin heavy locus	IGD1|IGH.1@|IGH@|IGHD@|IGHDY1|IGHJ|IGHJ@|IGHV|IGHV@	14	BCRSignalingPathway
IGHA1	3493	immunoglobulin heavy constant alpha 1	IgA1	14	BCRSignalingPathway
IGHA2	3494	immunoglobulin heavy constant alpha 2 (A2m marker)	-	14	BCRSignalingPathway
IGHD	3495	immunoglobulin heavy constant delta	-	14	BCRSignalingPathway
IGHD1	2851	immunoglobulin heavy diversity 1-1	IGHD11	14	BCRSignalingPathway

-1	0				
IGHD1-14	28508	immunoglobulin heavy diversity 1-14 (non-functional)	DM2|IGHD114	14	BCRSignalingPathway
IGHD1-20	28507	immunoglobulin heavy diversity 1-20	IGHD120	14	BCRSignalingPathway
IGHD1-26	28506	immunoglobulin heavy diversity 1-26	IGHD126	14	BCRSignalingPathway
IGHD1-7	28509	immunoglobulin heavy diversity 1-7	DM1|IGHD17	14	BCRSignalingPathway
IGHD2-15	28503	immunoglobulin heavy diversity 2-15	D2|IGHD215	14	BCRSignalingPathway
IGHD2-2	28505	immunoglobulin heavy diversity 2-2	IGHD22	14	BCRSignalingPathway
IGHD2-21	28502	immunoglobulin heavy diversity 2-21	IGHD221	14	BCRSignalingPathway
IGHD2-8	28504	immunoglobulin heavy diversity 2-8	DLR1|IGHD28	14	BCRSignalingPathway
IGHD3-10	28499	immunoglobulin heavy diversity 3-10	DXP'1|IGHD310	14	BCRSignalingPathway
IGHD3-16	28498	immunoglobulin heavy diversity 3-16	IGHD316	14	BCRSignalingPathway
IGHD3-22	28497	immunoglobulin heavy diversity 3-22	IGHD322	14	BCRSignalingPathway
IGHD3-3	28501	immunoglobulin heavy diversity 3-3	DXP4|IGHD33	14	BCRSignalingPathway
IGHD3	2850	immunoglobulin heavy diversity 3-9	DXP1|IGHD39	14	BCRSignalingPathway

-9	0				
IGHD4-11	28495	immunoglobulin heavy diversity 4-11 (non-functional)	DA1|IGHD411	14	BCRSignalingPathway
IGHD4-17	28494	immunoglobulin heavy diversity 4-17	IGHD417	14	BCRSignalingPathway
IGHD4-23	28493	immunoglobulin heavy diversity 4-23 (non-functional)	IGHD423	14	BCRSignalingPathway
IGHD4-4	28496	immunoglobulin heavy diversity 4-4	DA4|IGHD44	14	BCRSignalingPathway
IGHD5-12	28491	immunoglobulin heavy diversity 5-12	DK1|IGHD512	14	BCRSignalingPathway
IGHD5-18	28490	immunoglobulin heavy diversity 5-18	IGHD518	14	BCRSignalingPathway
IGHD5-24	28489	immunoglobulin heavy diversity 5-24 (non-functional)	IGHD524	14	BCRSignalingPathway
IGHD5-5	28492	immunoglobulin heavy diversity 5-5	DK4|IGHD55	14	BCRSignalingPathway
IGHD6-13	28487	immunoglobulin heavy diversity 6-13	DN1|IGHD613	14	BCRSignalingPathway
IGHD6-19	28486	immunoglobulin heavy diversity 6-19	IGHD619	14	BCRSignalingPathway
IGHD6-25	28485	immunoglobulin heavy diversity 6-25	IGHD625	14	BCRSignalingPathway
IGHD6-6	28488	immunoglobulin heavy diversity 6-6	D(N4)|IGHD66	14	BCRSignalingPathway
IGHD7	2848	immunoglobulin heavy diversity 7-27	DHQ52|IGHD727	14	BCRSignalingPathway

-27	4				
IGHE	3497	immunoglobulin heavy constant epsilon	IgE	14	BCRSignalingPathway
IGHG1	3500	immunoglobulin heavy constant gamma 1 (G1m marker)	-	14	BCRSignalingPathway
IGHG2	3501	immunoglobulin heavy constant gamma 2 (G2m marker)	-	14	BCRSignalingPathway
IGHG3	3502	immunoglobulin heavy constant gamma 3 (G3m marker)	IgG3	14	BCRSignalingPathway
IGHG4	3503	immunoglobulin heavy constant gamma 4 (G4m marker)	-	14	BCRSignalingPathway
IGHJ1	28483	immunoglobulin heavy joining 1	JH1	14	BCRSignalingPathway
IGHJ2	28481	immunoglobulin heavy joining 2	JH2	14	BCRSignalingPathway
IGHJ3	28479	immunoglobulin heavy joining 3	JH3b	14	BCRSignalingPathway
IGHJ4	28477	immunoglobulin heavy joining 4	JH4b	14	BCRSignalingPathway
IGHJ5	28476	immunoglobulin heavy joining 5	JH5b	14	BCRSignalingPathway
IGHJ6	28475	immunoglobulin heavy joining 6	JH6b	14	BCRSignalingPathway
IGHM	3507	immunoglobulin heavy constant mu	AGM1|MU|VH	14	BCRSignalingPathway
IGH	3492	immunoglobulin heavy locus	IGD1|IGH.1@|IGH@|IGHD@|IGHDY1|IGHJ|IGHJ@|IGHV|IGHV@	14	BCRSignalingPathway
IGHV1	2846	immunoglobulin heavy variable 1-18	IGHV118	14	BCRSignalingPathway

-18	8				
IGHV1-2	28474	immunoglobulin heavy variable 1-2	IGHV12|V35	14	BCRSignalingPathway
IGHV1-24	28467	immunoglobulin heavy variable 1-24	IGHV124|VH	14	BCRSignalingPathway
IGHV1-3	28473	immunoglobulin heavy variable 1-3	IGHV13|VI-3B	14	BCRSignalingPathway
IGHV1-45	28466	immunoglobulin heavy variable 1-45	IGHV145|VH	14	BCRSignalingPathway
IGHV1-46	28465	immunoglobulin heavy variable 1-46	IGHV146	14	BCRSignalingPathway
IGHV1-58	28464	immunoglobulin heavy variable 1-58	IGHV158|VH	14	BCRSignalingPathway
IGHV1-69	28461	immunoglobulin heavy variable 1-69	IGHV1-E|IGHV169|IGHV1E	14	BCRSignalingPathway
IGHV1-8	28472	immunoglobulin heavy variable 1-8	IGHV18	14	BCRSignalingPathway
IGHV1-38-4	28460	immunoglobulin heavy variable 1-38-4 (non-functional)	IGHV1-C|IGHV1C	14	BCRSignalingPathway
IGHV1-69-2	28458	immunoglobulin heavy variable 1-69-2	IGHV1-F|IGHV1F	14	BCRSignalingPathway
IGHV2-26	28455	immunoglobulin heavy variable 2-26	IGHV226|VH	14	BCRSignalingPathway
IGHV2-5	28457	immunoglobulin heavy variable 2-5	IGHV25|VH	14	BCRSignalingPathway
IGHV2	2845	immunoglobulin heavy variable 2-70	IGHV270|VH	14	BCRSignalingPathway

-70	4				
IGHV3-11	28450	immunoglobulin heavy variable 3-11	IGHV311|VH	14	BCRSignalingPathway
IGHV3-13	28449	immunoglobulin heavy variable 3-13	IGHV313	14	BCRSignalingPathway
IGHV3-15	28448	immunoglobulin heavy variable 3-15	IGHV315|VH	14	BCRSignalingPathway
IGHV3-16	28447	immunoglobulin heavy variable 3-16 (non-functional)	IGHV316|VH	14	BCRSignalingPathway
IGHV3-20	28445	immunoglobulin heavy variable 3-20	IGHV320|VH	14	BCRSignalingPathway
IGHV3-21	28444	immunoglobulin heavy variable 3-21	IGHV321|VH	14	BCRSignalingPathway
IGHV3-23	28442	immunoglobulin heavy variable 3-23	DP47|IGHV323|V3-23|VH26	14	BCRSignalingPathway
IGHV3-30	28439	immunoglobulin heavy variable 3-30	IGHV330|VH	14	BCRSignalingPathway
IGHV3-30-3	57290	immunoglobulin heavy variable 3-30-3	IGHV3-3|IGHV3303	14	BCRSignalingPathway
IGHV3-30-5	89770	immunoglobulin heavy variable 3-30-5	IGHV3-3|IGHV3305	14	BCRSignalingPathway
IGHV3-33	28434	immunoglobulin heavy variable 3-33	IGHV333|VH	14	BCRSignalingPathway
IGHV3-35	28432	immunoglobulin heavy variable 3-35 (non-functional)	IGHV335|VH	14	BCRSignalingPathway
IGHV3	2842	immunoglobulin heavy variable 3-38	IGHV338|VH	14	BCRSignalingPathway

-38	9	(non-functional)			
IGHV3-43	28426	immunoglobulin heavy variable 3-43	IGHV343|VH	14	BCRSignalingPathway
IGHV3-48	28424	immunoglobulin heavy variable 3-48	IGHV348|VH	14	BCRSignalingPathway
IGHV3-49	28423	immunoglobulin heavy variable 3-49	IGHV349|VH	14	BCRSignalingPathway
IGHV3-53	28420	immunoglobulin heavy variable 3-53	IGHV353|VH	14	BCRSignalingPathway
IGHV3-64	28414	immunoglobulin heavy variable 3-64	IGHV364|VH	14	BCRSignalingPathway
IGHV3-66	28412	immunoglobulin heavy variable 3-66	IGHV366|VH	14	BCRSignalingPathway
IGHV3-7	28452	immunoglobulin heavy variable 3-7	IGHV37|VH	14	BCRSignalingPathway
IGHV3-72	28410	immunoglobulin heavy variable 3-72	IGHV372|VH	14	BCRSignalingPathway
IGHV3-73	28409	immunoglobulin heavy variable 3-73	IGHV373|VH	14	BCRSignalingPathway
IGHV3-74	28408	immunoglobulin heavy variable 3-74	IGHV374|VH	14	BCRSignalingPathway
IGHV3-9	28451	immunoglobulin heavy variable 3-9	IGHV39|VH	14	BCRSignalingPathway
IGHV3-38-3	28404	immunoglobulin heavy variable 3-38-3 (non-functional)	IGHV3-D|IGHV3D	14	BCRSignalingPathway
IGHV3	2840	immunoglobulin heavy variable 3-69-1	IGH|IGHM|IGHV|IGHV3-11|IGHV3-H|IGHV3	14	BCRSignalingPathway

-69-1	2	(pseudogene)	H|IgVH		
IGHV4-28	28400	immunoglobulin heavy variable 4-28	IGHV428|VH	14	BCRSignalingPathway
IGHV4-30-1	28399	immunoglobulin heavy variable 4-30-1	IGHV4-3	14	BCRSignalingPathway
IGHV4-30-2	28398	immunoglobulin heavy variable 4-30-2	IGHV4-3|IGHV4302	14	BCRSignalingPathway
IGHV4-30-4	28397	immunoglobulin heavy variable 4-30-4	IGHV4-3|IGHV4304	14	BCRSignalingPathway
IGHV4-31	28396	immunoglobulin heavy variable 4-31	IGHV431	14	BCRSignalingPathway
IGHV4-34	28395	immunoglobulin heavy variable 4-34	IGHV434|VH	14	BCRSignalingPathway
IGHV4-39	28394	immunoglobulin heavy variable 4-39	IGHV439|VH	14	BCRSignalingPathway
IGHV4-4	28401	immunoglobulin heavy variable 4-4	IGHV44|VH	14	BCRSignalingPathway
IGHV4-59	28392	immunoglobulin heavy variable 4-59	IGHV459|VH	14	BCRSignalingPathway
IGHV4-61	28391	immunoglobulin heavy variable 4-61	IGHV461|VH	14	BCRSignalingPathway
IGHV4-38-2	28389	immunoglobulin heavy variable 4-38-2	IGHV4-B|IGHV4B	14	BCRSignalingPathway
IGHV5-51	28388	immunoglobulin heavy variable 5-51	IGHV551|VH	14	BCRSignalingPathway
IGHV5	2838	immunoglobulin heavy variable 5-10-1	IGHV5-A|IGHV5A	14	BCRSignalingPathway

-10-1	6				
IGHV6-1	28385	immunoglobulin heavy variable 6-1	IGHV61|VH	14	BCRSignalingPathway
IGHV7-4-1	57289	immunoglobulin heavy variable 7-4-1	IGHV7-41|IGHV741	14	BCRSignalingPathway
IGHV7-81	28378	immunoglobulin heavy variable 7-81 (non-functional)	IGHV781	14	BCRSignalingPathway
IGK	50802	immunoglobulin kappa locus	IGK@	2	BCRSignalingPathway
IGKC	3514	immunoglobulin kappa constant	HCAK1|IGKCD|Km	2	BCRSignalingPathway
IGKDEL	3515	immunoglobulin kappa deleting element or like	IGKDE	2	BCRSignalingPathway
IGKJ	7842	-	IGKJ@	2	BCRSignalingPathway
IGKJ1	28950	immunoglobulin kappa joining 1	J1	2	BCRSignalingPathway
IGKJ2	28949	immunoglobulin kappa joining 2	J2	2	BCRSignalingPathway
IGKJ3	28948	immunoglobulin kappa joining 3	J3	2	BCRSignalingPathway
IGKJ4	28947	immunoglobulin kappa joining 4	J4	2	BCRSignalingPathway
IGKJ5	28946	immunoglobulin kappa joining 5	J5	2	BCRSignalingPathway
IGKV@	3519	-	IGKV|IGKV1|IGKV1@|IGKV2|IGKV2@|IGKV3|IGKV3@	2	BCRSignalingPathway
IGKV1	2894	immunoglobulin kappa variable 1-12	IGKV112|L19	2	BCRSignalingPathway

-12	0				
IGKV1-13	28939	immunoglobulin kappa variable 1-13	IGKV113|L18	2	BCRSignalingPathway
IGKV1-16	28938	immunoglobulin kappa variable 1-16	IGKV116|L1	2	BCRSignalingPathway
IGKV1-17	28937	immunoglobulin kappa variable 1-17	A30|IGKV117	2	BCRSignalingPathway
IGKV1-27	28935	immunoglobulin kappa variable 1-27	A20|IGKV127	2	BCRSignalingPathway
IGKV1-33	28933	immunoglobulin kappa variable 1-33	IGKV133|O18	2	BCRSignalingPathway
IGKV1-37	28931	immunoglobulin kappa variable 1-37 (non-functional)	IGKV137|O14	2	BCRSignalingPathway
IGKV1-39	28930	immunoglobulin kappa variable 1-39	IGKV139|O12|O12a	2	BCRSignalingPathway
IGKV1-5	28299	immunoglobulin kappa variable 1-5	IGKV|IGKV15|L12|L12a|V1	2	BCRSignalingPathway
IGKV1-6	28943	immunoglobulin kappa variable 1-6	IGKV16|L11	2	BCRSignalingPathway
IGKV1-8	28942	immunoglobulin kappa variable 1-8	IGKV18|L9	2	BCRSignalingPathway
IGKV1-9	28941	immunoglobulin kappa variable 1-9	IGKV19|L8	2	BCRSignalingPathway
IGKV1D-12	28903	immunoglobulin kappa variable 1D-12	IGKV1D12|L19	2	BCRSignalingPathway
IGKV1	2890	immunoglobulin kappa variable 1D-13	IGKV1D13|L18	2	BCRSignalingPathway

D-13	2				
IGKV1 D-16	2890 1	immunoglobulin kappa variable 1D-16	IGKV1D16|L15|L15a	2	BCRSignalingPathway
IGKV1 D-17	2890 0	immunoglobulin kappa variable 1D-17	IGKV1D17|L14	2	BCRSignalingPathway
IGKV1 D-33	2889 6	immunoglobulin kappa variable 1D-33	IGKV1D33|O8	2	BCRSignalingPathway
IGKV1 D-37	2889 4	immunoglobulin kappa variable 1D-37 (non-functional)	IGKV1D37|O4	2	BCRSignalingPathway
IGKV1 D-39	2889 3	immunoglobulin kappa variable 1D-39	IGKV1D39|O2	2	BCRSignalingPathway
IGKV1 D-42	2889 2	immunoglobulin kappa variable 1D-42 (non-functional)	IGKV1D42|L22	2	BCRSignalingPathway
IGKV1 D-43	2889 1	immunoglobulin kappa variable 1D-43	IGKV1D43|L23|L23a	2	BCRSignalingPathway
IGKV1 D-8	2890 4	immunoglobulin kappa variable 1D-8	IGKV1D8|L24|L24a	2	BCRSignalingPathway
IGKV2 -24	2892 3	immunoglobulin kappa variable 2-24	A23|IGKV224	2	BCRSignalingPathway
IGKV2 -28	2892 1	immunoglobulin kappa variable 2-28	A19|IGKV228	2	BCRSignalingPathway
IGKV2 -30	2891 9	immunoglobulin kappa variable 2-30	A17|IGKV230	2	BCRSignalingPathway
IGKV2 -40	2891 6	immunoglobulin kappa variable 2-40	IGKV240|O11|O11a	2	BCRSignalingPathway
IGKV2	2888	immunoglobulin kappa variable 2D-24	A7|IGKV2D24	2	BCRSignalingPathway

D-24	5	(non-functional)			
IGKV2<br>D-28	2888<br>3	immunoglobulin kappa variable 2D-28	A3|IGKV2D28	2	BCRSignalingPathway
IGKV2<br>D-29	2888<br>2	immunoglobulin kappa variable 2D-29	A2a|A2c|IGKV2D29	2	BCRSignalingPathway
IGKV2<br>D-30	2888<br>1	immunoglobulin kappa variable 2D-30	A1|IGKV2D30	2	BCRSignalingPathway
IGKV2<br>D-40	2887<br>8	immunoglobulin kappa variable 2D-40	IGKV2D40|O1	2	BCRSignalingPathway
IGKV3<br>-11	2891<br>4	immunoglobulin kappa variable 3-11	IGKV311|L6	2	BCRSignalingPathway
IGKV3<br>-15	2891<br>3	immunoglobulin kappa variable 3-15	IGKV315|L2	2	BCRSignalingPathway
IGKV3<br>-20	2891<br>2	immunoglobulin kappa variable 3-20	13K18|A27|IGKV320	2	BCRSignalingPathway
IGKV3<br>-7	2891<br>5	immunoglobulin kappa variable 3-7<br>(non-functional)	IGKV37|L10|L10a|Vh	2	BCRSignalingPathway
IGKV3<br>D-11	2887<br>6	immunoglobulin kappa variable 3D-11	IGKV3D11|L20	2	BCRSignalingPathway
IGKV3<br>D-15	2887<br>5	immunoglobulin kappa variable 3D-15	IGKV3D15|L16|L16a|L16b|L16c	2	BCRSignalingPathway
IGKV3<br>D-20	2887<br>4	immunoglobulin kappa variable 3D-20	A11|A11a|IGKV3D20	2	BCRSignalingPathway
IGKV3<br>D-7	2887<br>7	immunoglobulin kappa variable 3D-7	IGKV3D7|L25	2	BCRSignalingPathway
IGKV4	2890	immunoglobulin kappa variable 4-1	B3|IGKV41	2	BCRSignalingPathway

-1	8			
IGKV5-2	28907	immunoglobulin kappa variable 5-2	B2|IGKV52	2	BCRSignalingPathway
IGKV6-21	28906	immunoglobulin kappa variable 6-21 (non-functional)	A26|IGKV621	2	BCRSignalingPathway
IGKV6D-21	28870	immunoglobulin kappa variable 6D-21 (non-functional)	A10|IGKV6D21	2	BCRSignalingPathway
IGKV6D-41	28869	immunoglobulin kappa variable 6D-41 (non-functional)	A14	2	BCRSignalingPathway
IGL	3535	immunoglobulin lambda locus	IGL@|IGLC6	22	BCRSignalingPathway
IGLC1	3537	immunoglobulin lambda constant 1	IGLC	22	BCRSignalingPathway
IGLC2	3538	immunoglobulin lambda constant 2	IGLC	22	BCRSignalingPathway
IGLC3	3539	immunoglobulin lambda constant 3 (Kern-Oz+ marker)	IGLC	22	BCRSignalingPathway
IGLC6	3542	immunoglobulin lambda constant 6	IGLC	22	BCRSignalingPathway
IGLC7	28834	immunoglobulin lambda constant 7	C7	22	BCRSignalingPathway
IGLJ	8217	-	IGLJ@	22	BCRSignalingPathway
IGLJ1	28833	immunoglobulin lambda joining 1	J1	22	BCRSignalingPathway
IGLJ2	28832	immunoglobulin lambda joining 2	J2	22	BCRSignalingPathway
IGLJ3	28831	immunoglobulin lambda joining 3	J3	22	BCRSignalingPathway
IGLJ4	28830	immunoglobulin lambda joining 4 (non-functional)	-	22	BCRSignalingPathway

IGLJ5	28829	immunoglobulin lambda joining 5 (non-functional)	-	22	BCRSignalingPathway
IGLJ6	28828	immunoglobulin lambda joining 6	-	22	BCRSignalingPathway
IGLJ7	28827	immunoglobulin lambda joining 7	J7	22	BCRSignalingPathway
IGLV@	3546	-	IGLV	22	BCRSignalingPathway
IGLV1-36	28826	immunoglobulin lambda variable 1-36	IGLV136|V1-11	22	BCRSignalingPathway
IGLV1-40	28825	immunoglobulin lambda variable 1-40	IGLV140|V1-13	22	BCRSignalingPathway
IGLV1-44	28823	immunoglobulin lambda variable 1-44	IGLV144|V1-16	22	BCRSignalingPathway
IGLV1-47	28822	immunoglobulin lambda variable 1-47	IGLV147|V1-17	22	BCRSignalingPathway
IGLV1-50	28821	immunoglobulin lambda variable 1-50 (non-functional)	IGLV150|V1-18	22	BCRSignalingPathway
IGLV1-51	28820	immunoglobulin lambda variable 1-51	IGLV151|V1-19	22	BCRSignalingPathway
IGLV10-54	28772	immunoglobulin lambda variable 10-54	IGLV1054|V1-20	22	BCRSignalingPathway
IGLV11-55	28770	immunoglobulin lambda variable 11-55 (non-functional)	IGLV1155|V4-6	22	BCRSignalingPathway
IGLV2-11	28816	immunoglobulin lambda variable 2-11	IGLV211|V1-3	22	BCRSignalingPathway
IGLV2	2881	immunoglobulin lambda variable 2-14	IGLV214|V1-4	22	BCRSignalingPathway

-14	5				
IGLV2-18	28814	immunoglobulin lambda variable 2-18	IGLV218|V1-5	22	BCRSignalingPathway
IGLV2-23	28813	immunoglobulin lambda variable 2-23	IGLV223|V1-7	22	BCRSignalingPathway
IGLV2-33	28811	immunoglobulin lambda variable 2-33 (non-functional)	IGLV233|V1-9	22	BCRSignalingPathway
IGLV2-8	28817	immunoglobulin lambda variable 2-8	IGLV28|V1-2	22	BCRSignalingPathway
IGLV3-1	28809	immunoglobulin lambda variable 3-1	IGLV31|V2-1	22	BCRSignalingPathway
IGLV3-10	28803	immunoglobulin lambda variable 3-10	IGLV310|V2-7	22	BCRSignalingPathway
IGLV3-12	28802	immunoglobulin lambda variable 3-12	IGLV312|V2-8	22	BCRSignalingPathway
IGLV3-16	28799	immunoglobulin lambda variable 3-16	IGLV316|V2-11	22	BCRSignalingPathway
IGLV3-19	28797	immunoglobulin lambda variable 3-19	IGLV319|V2-13|VL3L	22	BCRSignalingPathway
IGLV3-21	28796	immunoglobulin lambda variable 3-21	IGLV321|V2-14	22	BCRSignalingPathway
IGLV3-22	28795	immunoglobulin lambda variable 3-22	IGLV322|V2-15	22	BCRSignalingPathway
IGLV3-25	28793	immunoglobulin lambda variable 3-25	IGLV325|V2-17	22	BCRSignalingPathway
IGLV3	2879	immunoglobulin lambda variable 3-27	IGLV327|V2-19	22	BCRSignalingPathway

-27	1				
IGLV3-32	28787	immunoglobulin lambda variable 3-32 (non-functional)	IGLV332|V2-23P	22	BCRSignalingPathway
IGLV3-9	28804	immunoglobulin lambda variable 3-9	IGLV39|V2-6	22	BCRSignalingPathway
IGLV4-3	28786	immunoglobulin lambda variable 4-3	IGLV43|V5-1	22	BCRSignalingPathway
IGLV4-60	28785	immunoglobulin lambda variable 4-60	IGLV460|V5-4	22	BCRSignalingPathway
IGLV4-69	28784	immunoglobulin lambda variable 4-69	IGLV469|V5-6	22	BCRSignalingPathway
IGLV5-37	28783	immunoglobulin lambda variable 5-37	IGLV537|V4-1	22	BCRSignalingPathway
IGLV5-39	28782	immunoglobulin lambda variable 5-39	IGLV539	22	BCRSignalingPathway
IGLV5-45	28781	immunoglobulin lambda variable 5-45	IGLV545|V4-2	22	BCRSignalingPathway
IGLV5-48	28780	immunoglobulin lambda variable 5-48 (non-functional)	IGLV548|V4-3	22	BCRSignalingPathway
IGLV5-52	28779	immunoglobulin lambda variable 5-52	IGLV552|V4-4	22	BCRSignalingPathway
IGLV6-57	28778	immunoglobulin lambda variable 6-57	IGLV657|V1-22	22	BCRSignalingPathway
IGLV7-43	28776	immunoglobulin lambda variable 7-43	IGLV743|V3-2	22	BCRSignalingPathway
IGLV7	2877	immunoglobulin lambda variable 7-46	IGLV746|V3-3	22	BCRSignalingPathway

-46	5				
IGLV8-61	28774	immunoglobulin lambda variable 8-61	IGLV861|V3-4	22	BCRSignalingPathway
IGLV9-49	28773	immunoglobulin lambda variable 9-49	IGLV949|V5-2	22	BCRSignalingPathway
C3	718	complement C3	AHUS5|ARMD9|ASP|C3a|C3b|CPAMD1|HEL-S-62p	19	Chemokines
C5	727	complement C5	C5D|C5a|C5b|CPAMD4|ECLZB	9	Chemokines
CAMP	820	cathelicidin antimicrobial peptide	CAP-18|CAP18|CRAMP|FALL-39|FALL39|HSD26|LL37	3	Chemokines
CCL1	6346	C-C motif chemokine ligand 1	I-309|P500|SCYA1|SISe|TCA3	17	Chemokines
CCL11	6356	C-C motif chemokine ligand 11	SCYA11	17	Chemokines
CCL13	6357	C-C motif chemokine ligand 13	CKb10|MCP-4|NCC-1|NCC1|SCYA13|SCYL1	17	Chemokines
CCL14	6358	C-C motif chemokine ligand 14	CC-1|CC-3|CKB1|HCC-1|HCC-1(1-74)|HCC-1/HCC-3|HCC-3|MCIF|NCC-2|NCC2|SCYA14|SCYL2|SY14	17	Chemokines
CCL15-CCL14	348249	CCL15-CCL14 readthrough (NMD candidate)	CCL15|HCC-2|LKN-1|MIP-5|MIP5|Mrp-2b|NCC-3|NCC3|SCYA15	17	Chemokines
CCL15	6359	C-C motif chemokine ligand 15	HCC-2|HMRP-2B|LKN-1|LKN1|MIP-1 delta|MIP-1D|MIP-5|MRP-2B|NCC-3|NCC3|SCYA15|SCYL3|SY15	17	Chemokines
CCL16	6360	C-C motif chemokine ligand 16	CKb12|HCC-4|ILINCK|LCC-1|LEC|LMC|Mtn-1|NCC-4|NCC4|SCYA16|SCYL4	17	Chemokines
CCL17	6361	C-C motif chemokine ligand 17	A-152E5.3|ABCD-2|SCYA17|TARC	16	Chemokines
CCL18	6362	C-C motif chemokine ligand 18	AMAC-1|AMAC1|CKb7|DC-CK1|DCCK1|MIP-4	17	Chemokines

			|PARC|SCYA18		
CCL19	6363	C-C motif chemokine ligand 19	CKb11|ELC|MIP-3b|MIP3B|SCYA19	9	Chemokines
CCL2	6347	C-C motif chemokine ligand 2	GDCF-2|HC11|HSMCR30|MCAF|MCP-1|MCP1|SCYA2|SMC-CF	17	Chemokines
CCL20	6364	C-C motif chemokine ligand 20	CKb4|Exodus|LARC|MIP-3-alpha|MIP-3a|MIP3A|SCYA20|ST38	2	Chemokines
CCL21	6366	C-C motif chemokine ligand 21	6Ckine|CKb9|ECL|SCYA21|SLC|TCA4	9	Chemokines
CCL22	6367	C-C motif chemokine ligand 22	A-152E5.1|ABCD-1|DC/B-CK|MDC|SCYA22|STCP-1	16	Chemokines
CCL23	6368	C-C motif chemokine ligand 23	CK-BETA-8|CKb8|Ckb-8|Ckb-8-1|MIP-3|MIP3|MPIF-1|SCYA23|hmrp-2a	17	Chemokines
CCL24	6369	C-C motif chemokine ligand 24	Ckb-6|MPIF-2|MPIF2|SCYA24	7	Chemokines
CCL25	6370	C-C motif chemokine ligand 25	Ckb15|SCYA25|TECK	19	Chemokines
CCL26	10344	C-C motif chemokine ligand 26	IMAC|MIP-4a|MIP-4alpha|SCYA26|TSC-1	7	Chemokines
CCL27	10850	C-C motif chemokine ligand 27	ALP|CTACK|CTAK|ESKINE|ILC|PESKY|SCYA27	9	Chemokines
CCL28	56477	C-C motif chemokine ligand 28	CCK1|MEC|SCYA28	5	Chemokines
CCL3	6348	C-C motif chemokine ligand 3	G0S19-1|LD78ALPHA|MIP-1-alpha|MIP1A|SCYA3	17	Chemokines
CCL3L1	6349	C-C motif chemokine ligand 3 like 1	464.2|D17S1718|G0S19-2|LD78|LD78-beta(1-70)|LD78BETA|MIP1AP|SCYA3L|SCYA3L1	17	Chemokines
CCL3P1	390788	C-C motif chemokine ligand 3 pseudogene 1	CCL3L2|G0S19-3|LD78gamma|SCYA3L2	17	Chemokines

CCL3L3	414062	C-C motif chemokine ligand 3 like 3	464.2|D17S1718|G0S19-2|LD78|LD78BETA|SCYA3L|SCYA3L1	17	Chemokines
CCL4	6351	C-C motif chemokine ligand 4	ACT2|AT744.1|G-26|HC21|LAG-1|LAG1|MIP-1-beta|MIP1B|MIP1B1|SCYA2|SCYA4	17	Chemokines
CCL4L2	9560	C-C motif chemokine ligand 4 like 2	AT744.2|CCL4L|SCYA4L|SCYQ4L2	17	Chemokines
CCL4L1	388372	C-C motif chemokine ligand 4 like 1	AT744.2|CCL4L|LAG-1|LAG1|MIP-1-beta|SCYA4L|SCYA4L1|SCYA4L2	17	Chemokines
CCL5	6352	C-C motif chemokine ligand 5	D17S136E|RANTES|SCYA5|SIS-delta|SISd|TCP228|eoCP	17	Chemokines
CCL7	6354	C-C motif chemokine ligand 7	FIC|MARC|MCP-3|MCP3|NC28|SCYA6|SCYA7	17	Chemokines
CCL8	6355	C-C motif chemokine ligand 8	HC14|MCP-2|MCP2|SCYA10|SCYA8	17	Chemokines
CKLF	51192	chemokine like factor	C32|CKLF1|CKLF2|CKLF3|CKLF4|HSPC224|UCK-1	16	Chemokines
CMA1	1215	chymase 1	CYH|MCT1|chymase	14	Chemokines
CTSG	1511	cathepsin G	CATG|CG	14	Chemokines
CX3CL1	6376	C-X3-C motif chemokine ligand 1	ABCD-3|C3Xkine|CXC3|CXC3C|NTN|NTT|SCYD1|fractalkine|neurotactin	16	Chemokines
CXCL1	2919	C-X-C motif chemokine ligand 1	FSP|GRO1|GROa|MGSA|MGSA-a|NAP-3|SCYB1	4	Chemokines
CXCL10	3627	C-X-C motif chemokine ligand 10	C7|IFI10|INP10|IP-10|SCYB10|crg-2|gIP-10|mob-1	4	Chemokines
CXCL11	6373	C-X-C motif chemokine ligand 11	H174|I-TAC|IP-9|IP9|SCYB11|SCYB9B|b-R1	4	Chemokines
CXCL12	6387	C-X-C motif chemokine ligand 12	IRH|PBSF|SCYB12|SDF1|TLSF|TPAR1	10	Chemokines

CXCL13	10563	C-X-C motif chemokine ligand 13	ANGIE|ANGIE2|BCA-1|BCA1|BLC|BLR1L|SCYB13	4	Chemokines
CXCL14	9547	C-X-C motif chemokine ligand 14	BMAC|BRAK|KEC|KS1|MIP-2g|MIP2G|NJAC|SCYB14	5	Chemokines
CXCL16	58191	C-X-C motif chemokine ligand 16	CXCLG16|SR-PSOX|SRPSOX	17	Chemokines
CXCL17	284340	C-X-C motif chemokine ligand 17	DMC|Dcip1|UNQ473|VCC-1|VCC1	19	Chemokines
CXCL2	2920	C-X-C motif chemokine ligand 2	CINC-2a|GRO2|GROb|MGSA-b|MIP-2a|MIP2|MIP2A|SCYB2	4	Chemokines
CXCL3	2921	C-X-C motif chemokine ligand 3	CINC-2b|GRO3|GROg|MIP-2b|MIP2B|SCYB3	4	Chemokines
CXCL5	6374	C-X-C motif chemokine ligand 5	ENA-78|SCYB5	4	Chemokines
CXCL6	6372	C-X-C motif chemokine ligand 6	CKA-3|GCP-2|GCP2|SCYB6	4	Chemokines
CXCL9	4283	C-X-C motif chemokine ligand 9	CMK|Humig|MIG|SCYB9|crg-10	4	Chemokines
CCN1	3491	cellular communication network factor 1	CYR61|GIG1|IGFBP10	1	Chemokines
DEFA1	1667	defensin alpha 1	DEF1|DEFA2|HNP-1|HP-1|HP1|MRS	8	Chemokines
DEFA3	1668	defensin alpha 3	DEF3|HNP-3|HNP3|HP-3|HP3	8	Chemokines
DEFA5	1670	defensin alpha 5	DEF5|HD-5	8	Chemokines
DEFB1	1672	defensin beta 1	BD1|DEFB-1|DEFB101|HBD1	8	Chemokines
DEFB103B	55894	defensin beta 103B	BD-3|DEFB-3|DEFB103|DEFB3|HBD-3|HBD3|HBP-3|HBP3	8	Chemokines
DEFB104A	140596	defensin beta 104A	BD-4|DEFB-4|DEFB104|DEFB4|hBD-4	8	Chemokines
DEFB4A	1673	defensin beta 4A	BD-2|DEFB-2|DEFB102|DEFB2|DEFB4|HBD-2|SAP1	8	Chemokines
EDN1	1906	endothelin 1	ARCND3|ET1|HDLCQ7|PPET1|QME	6	Chemokines

EDN2	1907	endothelin 2	ET-2|ET2|PPET2	1	Chemokines
EDN3	1908	endothelin 3	ET-3|ET3|HSCR4|PPET3|WS4B	20	Chemokines
FGF10	2255	fibroblast growth factor 10	-	5	Chemokines
FGF2	2247	fibroblast growth factor 2	BFGF|FGF-2|FGFB|HBGF-2	4	Chemokines
HTN3	3347	histatin 3	HIS2|HTN2|HTN5|PB	4	Chemokines
CXCL8	3576	C-X-C motif chemokine ligand 8	GCP-1|GCP1|IL8|LECT|LUCT|LYNAP|MDNCF|MONAP|NAF|NAP-1|NAP1|SCYB8	4	Chemokines
LECT2	3950	leukocyte cell derived chemotaxin 2	chm-II|chm2	5	Chemokines
PF4	5196	platelet factor 4	CXCL4|PF-4|SCYB4	4	Chemokines
PF4V1	5197	platelet factor 4 variant 1	CXCL4L1|CXCL4V1|PF4-ALT|PF4A|SCYB4V1	4	Chemokines
PLAU	5328	plasminogen activator, urokinase	ATF|BDPLT5|QPD|UPA|URK|u-PA	10	Chemokines
PPBP	5473	pro-platelet basic protein	B-TG1|Beta-TG|CTAP-III|CTAP3|CTAPIII|CXCL7|LA-PF4|LDGF|MDGF|NAP-2|PBP|SCYB7|TC1|TC2|TGB|TGB1|THBGB|THBGB1	4	Chemokines
PPBPP1	728045	pro-platelet basic protein pseudogene 1	PPBPL1|TGB2	4	Chemokines
PROK2	60675	prokineticin 2	BV8|HH4|KAL4|MIT1|PK2	3	Chemokines
RNASE2	6036	ribonuclease A family member 2	EDN|RAF3|RNS2	14	Chemokines
SAA1	6288	serum amyloid A1	PIG4|SAA|SAA2|TP53I4	11	Chemokines
SAA2	6289	serum amyloid A2	SAA|SAA1	11	Chemokines
SBDS	51119	SBDS ribosome maturation factor	CGI-97|SDS|SWDS	7	Chemokines
SEMA3A	10371	semaphorin 3A	COLL1|HH16|Hsema-I|Hsema-III|SEMA1|SEMAD|SEMAIII|SEMAL|SemD|coll-1	7	Chemokines

SEMA3B	7869	semaphorin 3B	LUCA-1|SEMA5|SEMAA|SemA|semaV	3	Chemokines
SEMA3C	10512	semaphorin 3C	SEMAE|SemE	7	Chemokines
SEMA3D	223117	semaphorin 3D	Sema-Z2|coll-2	7	Chemokines
SEMA3E	9723	semaphorin 3E	M-SEMAH|M-SemaK|SEMAH|coll-5	7	Chemokines
SEMA3F	6405	semaphorin 3F	SEMA-IV|SEMA4|SEMAK	3	Chemokines
SEMA3G	56920	semaphorin 3G	sem2	3	Chemokines
SEMA4A	64218	semaphorin 4A	CORD10|RP35|SEMAB|SEMB	1	Chemokines
SEMA4B	10509	semaphorin 4B	SEMAC|SemC	15	Chemokines
SEMA4C	54910	semaphorin 4C	M-SEMA-F|SEMACL1|SEMAF|SEMAI	2	Chemokines
SEMA4D	10507	semaphorin 4D	A8|BB18|C9orf164|CD100|COLL4|GR3|M-sema-G|SEMAJ|coll-4	9	Chemokines
SEMA4F	10505	ssemaphorin 4F	M-SEMA|PRO2353|S4F|SEMAM|SEMAW|m-Sema-M	2	Chemokines
SEMA4G	57715	semaphorin 4G	-	10	Chemokines
SEMA5A	9037	semaphorin 5A	SEMAF|semF	5	Chemokines

SEMA5B	54437	semaphorin 5B	SEMAG|SemG	3	Chemokines
SEMA6A	57556	semaphorin 6A	HT018|SEMA|SEMA6A1|SEMAQ|VIA	5	Chemokines
SEMA6B	10501	semaphorin 6B	EPM11|SEM-SEMA-Y|SEMA-VIB|SEMAN|semaZ	19	Chemokines
SEMA6C	10500	semaphorin 6C	SEMAY|m-SemaY|m-SemaY2	1	Chemokines
SEMA6D	80031	semaphorin 6D	-	15	Chemokines
SEMA7A	8482	semaphorin 7A (John Milton Hagen blood group)	CD108|CDw108|H-SEMA-K1|H-Sema-L|JMH|SEMAK1|SEMAL	15	Chemokines
SLIT1	6585	slit guidance ligand 1	MEGF4|SLIL1|SLIT-1|SLIT3	10	Chemokines
SLIT2	9353	slit guidance ligand 2	SLIL3|Slit-2	4	Chemokines
TNC	3371	tenascin C	150-225|DFNA56|GMEM|GP|HXB|JI|TN|TN-C	9	Chemokines
TYMP	1890	thymidine phosphorylase	ECGF|ECGF1|MEDPS1|MNGIE|MTDPS1|PDECGF|TP|hPD-ECGF	22	Chemokines
XCL1	6375	X-C motif chemokine ligand 1	ATAC|LPTN|LTN|SCM-1|SCM-1a|SCM1|SCM1A|SCYC1	1	Chemokines
XCL2	6846	X-C motif chemokine ligand 2	SCM-1b|SCM1B|SCYC2	1	Chemokines
C5AR1	728	complement C5a receptor 1	C5A|C5AR|C5R1|CD88	19	Chemokine_Receptors
ACKR2	1238	atypical chemokine receptor 2	CCBP2|CCR10|CCR9|CMKBR9|D6|hD6	3	Chemokine_Receptors
CCR1	1230	C-C motif chemokine receptor 1	CD191|CKR-1|CKR1|CMKBR1|HM145|MIP1aR|SCYAR1	3	Chemokine_Receptors
CCR10	2826	C-C motif chemokine receptor 10	GPR2	17	Chemokine_Receptors

CCR3	1232	C-C motif chemokine receptor 3	C C CKR3|CC-CKR-3|CD193|CKR 3|CKR3|CMKBR3		3	Chemokine_Receptors
CCR4	1233	C-C motif chemokine receptor 4	CC-CKR-4|CD194|CKR4|CMKBR4|ChemR13|HGCN:14099|K5-5		3	Chemokine_Receptors
CCR5	1234	C-C motif chemokine receptor 5	CC-CKR-5|CCCKR5|CCR-5|CD195|CKR-5|CKR5|CMKBR5|IDDM22		3	Chemokine_Receptors
CCR6	1235	C-C motif chemokine receptor 6	BN-1|C-C CKR-6|CC-CKR-6|CCR-6|CD196|CKR-L3|CKRL3|CMKBR6|DCR2|DRY6|GPR29|GPRCY4|STRL22		6	Chemokine_Receptors
CCR7	1236	C-C motif chemokine receptor 7	BLR2|CC-CKR-7|CCR-7|CD197|CDw197|CMKBR7|EBI1		17	Chemokine_Receptors
CCR8	1237	C-C motif chemokine receptor 8	CC-CKR-8|CCR-8|CDw198|CKRL1|CMKBR8|CMKBRL2|CY6|GPRCY6|TER1		3	Chemokine_Receptors
CCR9	10803	C-C motif chemokine receptor 9	CC-CKR-9|CDw199|GPR-9-6|GPR28		3	Chemokine_Receptors
ACKR4	51554	atypical chemokine receptor 4	CC-CKR-11|CCBP2|CCR-11|CCR10|CCR11|CCRL1|CCX CKR|CCX-CKR|CKR-11|PPR1|VSHK1		3	Chemokine_Receptors
CCRL2	9034	C-C motif chemokine receptor like 2	ACKR5|CKRX|CRAM|CRAM-A|CRAM-B|HCR		3	Chemokine_Receptors
CMKLR1	1240	chemerin chemokine-like receptor 1	CHEMERINR|ChemR23|DEZ|RVER1		12	Chemokine_Receptors
CX3CR1	1524	C-X3-C motif chemokine receptor 1	CCRL1|CMKBRL1|CMKDR1|GPR13|GPRV28|V28		3	Chemokine_Receptors
CXCR3	2833	C-X-C motif chemokine receptor 3	CD182|CD183|CKR-L2|CMKAR3|GPR9|IP10-R|Mig-R|MigR	X		Chemokine_Receptors

CXCR4	7852	C-X-C motif chemokine receptor 4	CD184|D2S201E|FB22|HM89|HSY3RR|LAP-3|LAP3|LCR1|LESTR|NPY3R|NPYR|NPYRL|NPYY3R|WHIM|WHIMS		2	Chemokine_Receptors
CXCR5	643	C-X-C motif chemokine receptor 5	BLR1|CD185|MDR15		11	Chemokine_Receptors
CXCR6	10663	C-X-C motif chemokine receptor 6	BONZO|CD186|STRL33|TYMSTR		3	Chemokine_Receptors
ACKR3	57007	atypical chemokine receptor 3	CMKOR1|CXC-R7|CXCR-7|CXCR7|GPR159|RDC-1|RDC1		2	Chemokine_Receptors
CYSLTR1	10800	cysteinyl leukotriene receptor 1	CYSLT1|CYSLT1R|CYSLTR|HMTMF81	X		Chemokine_Receptors
CYSLTR2	57105	cysteinyl leukotriene receptor 2	CYSLT2|CYSLT2R|GPCR21|HG57|HPN321|KPG_011|PSEC0146|hGPCR21		13	Chemokine_Receptors
ACKR1	2532	atypical chemokine receptor 1 (Duffy blood group)	CCBP1|CD234|DARC|DARC/ACKR1|Dfy|FY|GPD|GpFy|WBCQ1		1	Chemokine_Receptors
EDNRA	1909	endothelin receptor type A	ET-A|ETA|ETA-R|ETAR|ETRA|MFDA|hET-AR		4	Chemokine_Receptors
EDNRB	1910	endothelin receptor type B	ABCDS|ET-B|ET-BR|ETB|ETB1|ETBR|ETRB|HSCR|HSCR2|WS4A		13	Chemokine_Receptors
FPR1	2357	formyl peptide receptor 1	FMLP|FPR		19	Chemokine_Receptors
FPR2	2358	formyl peptide receptor 2	ALXR|FMLP-R-II|FMLPX|FPR2A|FPRH1|FPRH2|FPRL1|HM63|LXA4R		19	Chemokine_Receptors
FPR2	2358	formyl peptide receptor 2	ALXR|FMLP-R-II|FMLPX|FPR2A|FPRH1|FPRH2|FPRL1|HM63|LXA4R		19	Chemokine_Receptors
GPR17	2840	G protein-coupled receptor 17	-		2	Chemokine_Receptors
GPR32	2854	G protein-coupled receptor 32	RVDR1		19	Chemokine_Receptors
GPR33	2856	G protein-coupled receptor 33	-		14	Chemokine_Receptors
PTGDR	1125	prostaglandin D2 receptor 2	CD294|CRTH2|DL1R|DP2|GPR44		11	Chemokine_Receptors

2	1					
C5AR2	27202	complement component 5a receptor 2	C5L2|GPF77|GPR77		19	Chemokine_Receptors
CXCR1	3577	C-X-C motif chemokine receptor 1	C-C|C-C-CKR-1|CD128|CD181|CDw128a|CKR-1|CMKAR1|IL8R1|IL8RA|IL8RBA		2	Chemokine_Receptors
CXCR2	3579	C-X-C motif chemokine receptor 2	CD182|CDw128b|CMKAR2|IL8R2|IL8RA|IL8RB		2	Chemokine_Receptors
LTB4R	1241	leukotriene B4 receptor	BLT1|BLTR|CMKRL1|GPR16|LTB4R1|LTBR1|P2RY7|P2Y7		14	Chemokine_Receptors
LTB4R2	56413	leukotriene B4 receptor 2	BLT2|BLTR2|JULF2|KPG_004|LTB4-R2|LTB4-R2|NOP9		14	Chemokine_Receptors
PLAUR	5329	plasminogen activator, urokinase receptor	CD87|U-PAR|UPAR|URKR		19	Chemokine_Receptors
PLXNA1	5361	plexin A1	NOV|NOVP|PLEXIN-A1|PLXN1		3	Chemokine_Receptors
PLXNA2	5362	plexin A2	OCT|PLXN2		1	Chemokine_Receptors
PLXNA3	55558	plexin A3	6.3|HSSEXGENE|PLXN3|PLXN4|XAP-6	X		Chemokine_Receptors
PLXNA4	91584	plexin A4	FAYV2820|PLEXA4|PLXNA4A|PLXNA4B|PRO34003		7	Chemokine_Receptors
PLXNB1	5364	plexin B1	PLEXIN-B1|PLXN5|SEP		3	Chemokine_Receptors
PLXNB2	23654	plexin B2	MM1|Nbla00445|PLEXB2|dJ402G11.3		22	Chemokine_Receptors
PLXNB3	5365	plexin B3	PLEXB3|PLEXR|PLXN6	X		Chemokine_Receptors

PLXNC1	10154	plexin C1	CD232|PLXN-C1|VESPR	12	Chemokine_Receptors
PLXND1	23129	plexin D1	PLEXD1	3	Chemokine_Receptors
PTAFR	5724	platelet activating factor receptor	PAFR	1	Chemokine_Receptors
ROBO1	6091	roundabout guidance receptor 1	DUTT1|SAX3	3	Chemokine_Receptors
ROBO2	6092	roundabout guidance receptor 2	SAX3	3	Chemokine_Receptors
ROBO3	64221	roundabout guidance receptor 3	HGPPS|HGPPS1|HGPS|RBIG1|RIG1	11	Chemokine_Receptors
RXFP3	51289	relaxin family peptide receptor 3	GPCR135|RLN3R1|RXFPR3|SALPR	5	Chemokine_Receptors
XCR1	2829	X-C motif chemokine receptor 1	CCXCR1|GPR5	3	Chemokine_Receptors
ADIPOQ	9370	adiponectin, C1Q and collagen domain containing	ACDC|ACRP30|ADIPQTL1|ADPN|APM-1|APM1|GBP28	3	Cytokines
ADM	133	adrenomedullin	AM|PAMP	11	Cytokines
ADM2	79924	adrenomedullin 2	AM2|dJ579N16.4	22	Cytokines
AGRP	181	agouti related neuropeptide	AGRT|ART|ASIP2	16	Cytokines
AGT	183	angiotensinogen	ANHU|SERPINA8|hFLT1	1	Cytokines
AMBN	258	ameloblastin	AI1F	4	Cytokines
AMELX	265	amelogenin X-linked	AI1E|AIH1|ALGN|AMG|AMGL|AMGX	X	Cytokines
AMH	268	anti-Mullerian hormone	MIF|MIS	19	Cytokines
ANGPTL5	253935	angiopoietin like 5	-	11	Cytokines
ANGPTL7	10218	angiopoietin like 7	AngX|CDT6|dJ647M16.1	1	Cytokines

APLN	8862	apelin	APEL|XNPEP2	X		Cytokines
AREG	374	amphiregulin	AR|AREGB|CRDGF|SDGF		4	Cytokines
MANF	7873	mesencephalic astrocyte derived neurotrophic factor	ARMET|ARP		3	Cytokines
CDNF	441549	cerebral dopamine neurotrophic factor	ARMETL1		10	Cytokines
ARTN	9048	artemin	ART|ENOVIN|EVN|NBN		1	Cytokines
AVP	551	arginine vasopressin	ADH|ARVP|AVP-NPII|AVRP|VP		20	Cytokines
AZU1	566	azurocidin 1	AZAMP|AZU|CAP37|HBP|HUMAZUR|NAZC|hHBP		19	Cytokines
BDNF	627	brain derived neurotrophic factor	ANON2|BULN2		11	Cytokines
BMP1	649	bone morphogenetic protein 1	OI13|PCOLC|PCP|PCP2|TLD		8	Cytokines
BMP10	27302	bone morphogenetic protein 10	-		2	Cytokines
BMP15	9210	bone morphogenetic protein 15	GDF9B|ODG2|POF4	X		Cytokines
BMP2	650	bone morphogenetic protein 2	BDA2|BMP2A|SSFSC		20	Cytokines
BMP3	651	bone morphogenetic protein 3	BMP-3A		4	Cytokines
BMP4	652	bone morphogenetic protein 4	BMP2B|BMP2B1|MCOPS6|OFC11|ZYME		14	Cytokines
BMP5	653	bone morphogenetic protein 5	-		6	Cytokines
BMP6	654	bone morphogenetic protein 6	VGR|VGR1		6	Cytokines
BMP7	655	bone morphogenetic protein 7	OP-1		20	Cytokines
BMP8A	353500	bone morphogenetic protein 8a	OP-2		1	Cytokines
BMP8B	656	bone morphogenetic protein 8b	BMP8|OP2		1	Cytokines
BTC	685	betacellulin	-		4	Cytokines
MYDGF	5600	myeloid derived growth factor	C19orf10|EUROIMAGE1875335|IL25|IL27|		19	Cytokines

	5		IL27w|R33729_1|SF20		
C3	718	complement C3	AHUS5|ARMD9|ASP|C3a|C3b|CPAMD1|HEL-S-62p	19	Cytokines
C5	727	complement C5	C5D|C5a|C5b|CPAMD4|ECLZB	9	Cytokines
CALCA	796	calcitonin related polypeptide alpha	CALC1|CGRP|CGRP-I|CGRP-alpha|CGRP1|CT|KC|PCT	11	Cytokines
CALCB	797	calcitonin related polypeptide beta	CALC2|CGRP-II|CGRP2	11	Cytokines
CAMP	820	cathelicidin antimicrobial peptide	CAP-18|CAP18|CRAMP|FALL-39|FALL39|HSD26|LL37	3	Cytokines
CAT	847	catalase	-	11	Cytokines
CCK	885	cholecystokinin	-	3	Cytokines
CCL1	6346	C-C motif chemokine ligand 1	I-309|P500|SCYA1|SISe|TCA3	17	Cytokines
CCL11	6356	C-C motif chemokine ligand 11	SCYA11	17	Cytokines
CCL13	6357	C-C motif chemokine ligand 13	CKb10|MCP-4|NCC-1|NCC1|SCYA13|SCYL1	17	Cytokines
CCL14	6358	C-C motif chemokine ligand 14	CC-1|CC-3|CKB1|HCC-1|HCC-1(1-74)|HCC-1/HCC-3|HCC-3|MCIF|NCC-2|NCC2|SCYA14|SCYL2|SY14	17	Cytokines
CCL15-CCL14	348249	CCL15-CCL14 readthrough (NMD candidate)	CCL15|HCC-2|LKN-1|MIP-5|MIP5|Mrp-2b|NCC-3|NCC3|SCYA15	17	Cytokines
CCL15	6359	C-C motif chemokine ligand 15	HCC-2|HMRP-2B|LKN-1|LKN1|MIP-1 delta|MIP-1D|MIP-5|MRP-2B|NCC-3|NCC3|SCYA15|SCYL3|SY15	17	Cytokines
CCL16	6360	C-C motif chemokine ligand 16	CKb12|HCC-4|ILINCK|LCC-1|LEC|LMC|Mtn-1|NCC-4|NCC4|SCYA16|SCYL4	17	Cytokines
CCL17	6361	C-C motif chemokine ligand 17	A-152E5.3|ABCD-2|SCYA17|TARC	16	Cytokines

CCL18	6362	C-C motif chemokine ligand 18	AMAC-1|AMAC1|CKb7|DC-CK1|DCCK1|MIP-4|PARC|SCYA18	17	Cytokines
CCL19	6363	C-C motif chemokine ligand 19	CKb11|ELC|MIP-3b|MIP3B|SCYA19	9	Cytokines
CCL2	6347	C-C motif chemokine ligand 2	GDCF-2|HC11|HSMCR30|MCAF|MCP-1|MCP1|SCYA2|SMC-CF	17	Cytokines
CCL20	6364	C-C motif chemokine ligand 20	CKb4|Exodus|LARC|MIP-3-alpha|MIP-3a|MIP3A|SCYA20|ST38	2	Cytokines
CCL21	6366	C-C motif chemokine ligand 21	6Ckine|CKb9|ECL|SCYA21|SLC|TCA4	9	Cytokines
CCL22	6367	C-C motif chemokine ligand 22	A-152E5.1|ABCD-1|DC/B-CK|MDC|SCYA22|STCP-1	16	Cytokines
CCL23	6368	C-C motif chemokine ligand 23	CK-BETA-8|CKb8|Ckb-8|Ckb-8-1|MIP-3|MIP3|MPIF-1|SCYA23|hmrp-2a	17	Cytokines
CCL24	6369	C-C motif chemokine ligand 24	Ckb-6|MPIF-2|MPIF2|SCYA24	7	Cytokines
CCL25	6370	C-C motif chemokine ligand 25	Ckb15|SCYA25|TECK	19	Cytokines
CCL26	10344	C-C motif chemokine ligand 26	IMAC|MIP-4a|MIP-4alpha|SCYA26|TSC-1	7	Cytokines
CCL27	10850	C-C motif chemokine ligand 27	ALP|CTACK|CTAK|ESKINE|ILC|PESKY|SCYA27	9	Cytokines
CCL28	56477	C-C motif chemokine ligand 28	CCK1|MEC|SCYA28	5	Cytokines
CCL3	6348	C-C motif chemokine ligand 3	G0S19-1|LD78ALPHA|MIP-1-alpha|MIP1A|SCYA3	17	Cytokines
CCL3L1	6349	C-C motif chemokine ligand 3 like 1	464.2|D17S1718|G0S19-2|LD78|LD78-beta(1-70)|LD78BETA|MIP1AP|SCYA3L|SCYA3L1	17	Cytokines
CCL3P	3907	C-C motif chemokine ligand 3 pseudogene 1	CCL3L2|G0S19-3|LD78gamma|SCYA3L2	17	Cytokines

1	88					
CCL3L3	414062	C-C motif chemokine ligand 3 like 3	464.2|D17S1718|G0S19-2|LD78|LD78BETA|SCYA3L|SCYA3L1		17	Cytokines
CCL4	6351	C-C motif chemokine ligand 4	ACT2|AT744.1|G-26|HC21|LAG-1|LAG1|MIP-1-beta|MIP1B|MIP1B1|SCYA2|SCYA4		17	Cytokines
CCL4L2	9560	C-C motif chemokine ligand 4 like 2	AT744.2|CCL4L|SCYA4L|SCYQ4L2		17	Cytokines
CCL4L1	388372	C-C motif chemokine ligand 4 like 1	AT744.2|CCL4L|LAG-1|LAG1|MIP-1-beta|SCYA4L|SCYA4L1|SCYA4L2		17	Cytokines
CCL5	6352	C-C motif chemokine ligand 5	D17S136E|RANTES|SCYA5|SIS-delta|SISd|TCP228|eoCP		17	Cytokines
CCL7	6354	C-C motif chemokine ligand 7	FIC|MARC|MCP-3|MCP3|NC28|SCYA6|SCYA7		17	Cytokines
CCL8	6355	C-C motif chemokine ligand 8	HC14|MCP-2|MCP2|SCYA10|SCYA8		17	Cytokines
CD320	51293	CD320 molecule	8D6|8D6A|TCBLR|TCN2R		19	Cytokines
CD40LG	959	CD40 ligand	CD154|CD40L|HIGM1|IGM|IMD3|T-BAM|TNFSF5|TRAP|gp39|hCD40L	X		Cytokines
CD70	970	CD70 molecule	CD27-L|CD27L|CD27LG|LPFS3|TNFSF7|TNLG8A		19	Cytokines
ADA2	51816	adenosine deaminase 2	ADGF|CECR1|IDGFL|PAN|SNEDS|VAIHS		22	Cytokines
CER1	9350	cerberus 1, DAN family BMP antagonist	DAND4		9	Cytokines
CGA	1081	glycoprotein hormones, alpha polypeptide	CG-ALPHA|FSHA|GPA1|GPHA1|GPHa|HCG|LHA|TSHA		6	Cytokines
CGB3	1082	chorionic gonadotropin subunit beta 3	CGB|CGB5|CGB7|CGB8|hCGB		19	Cytokines
CGB1	1143	chorionic gonadotropin subunit beta 1	-		19	Cytokines

	35				
CGB2	114336	chorionic gonadotropin subunit beta 2	-	19	Cytokines
CGB5	93659	chorionic gonadotropin subunit beta 5	CGB|HCG|hCGB	19	Cytokines
CGB7	94027	chorionic gonadotropin subunit beta 7	CG-beta-a|CGB6	19	Cytokines
CGB8	94115	chorionic gonadotropin subunit beta 8	-	19	Cytokines
CHGA	1113	chromogranin A	CGA	14	Cytokines
CHGB	1114	chromogranin B	SCG1	20	Cytokines
CKLF	51192	chemokine like factor	C32|CKLF1|CKLF2|CKLF3|CKLF4|HSPC224|UCK-1	16	Cytokines
CLCF1	23529	cardiotrophin like cytokine factor 1	BSF-3|BSF3|CISS2|CLC|NNT-1|NNT1|NR6	11	Cytokines
CLEC11A	6320	C-type lectin domain containing 11A	CLECSF3|LSLCL|P47|SCGF	19	Cytokines
CMA1	1215	chymase 1	CYH|MCT1|chymase	14	Cytokines
CMTM1	113540	CKLF like MARVEL transmembrane domain containing 1	CKLFH|CKLFH1|CKLFSF1	16	Cytokines
CMTM2	146225	CKLF like MARVEL transmembrane domain containing 2	CKLFSF2	16	Cytokines
CMTM3	123920	CKLF like MARVEL transmembrane domain containing 3	BNAS2|CKLFSF3	16	Cytokines
CMTM4	146223	CKLF like MARVEL transmembrane domain containing 4	CKLFSF4	16	Cytokines

CMTM5	116173	CKLF like MARVEL transmembrane domain containing 5	CKLFSF5	14	Cytokines
CMTM6	54918	CKLF like MARVEL transmembrane domain containing 6	CKLFSF6|PRO2219	3	Cytokines
CMTM7	112616	CKLF like MARVEL transmembrane domain containing 7	CKLFSF7	3	Cytokines
CMTM8	152189	CKLF like MARVEL transmembrane domain containing 8	CKLFSF8|CKLFSF8-V2	3	Cytokines
CNTF	1270	ciliary neurotrophic factor	HCNTF	11	Cytokines
CORT	1325	cortistatin	CST-14|CST-17|CST-29	1	Cytokines
CRH	1392	corticotropin releasing hormone	CRF|CRH1	8	Cytokines
CSF1	1435	colony stimulating factor 1	CSF-1|MCSF	1	Cytokines
CSF2	1437	colony stimulating factor 2	CSF|GMCSF	5	Cytokines
CSF3	1440	colony stimulating factor 3	C17orf33|CSF3OS|GCSF	17	Cytokines
CSH1	1442	chorionic somatomammotropin hormone 1	CS-1|CSA|CSMT|GHB3|PL|hCS-1|hCS-A	17	Cytokines
CSH2	1443	chorionic somatomammotropin hormone 2	CS-2|CSB|GHB1|PL|hCS-B	17	Cytokines
CSHL1	1444	chorionic somatomammotropin hormone like 1	CS-5|CSHP1|CSL|GHB4|hCS-L	17	Cytokines
CSPG5	10675	chondroitin sulfate proteoglycan 5	NGC	3	Cytokines
CTF1	1489	cardiotrophin 1	CT-1|CT1	16	Cytokines
CCN2	1490	cellular communication network factor 2	CTGF|HCS24|IGFBP8|NOV2	6	Cytokines
CTSG	1511	cathepsin G	CATG|CG	14	Cytokines
CX3CL1	6376	C-X3-C motif chemokine ligand 1	ABCD-3|C3Xkine|CXC3|CXC3C|NTN|NTT|SCYD1|fractalkine|neurotactin	16	Cytokines
CXCL1	2919	C-X-C motif chemokine ligand 1	FSP|GRO1|GROa|MGSA|MGSA-a|NAP-3|SCYB1	4	Cytokines

CXCL10	3627	C-X-C motif chemokine ligand 10	C7|IFI10|INP10|IP-10|SCYB10|crg-2|gIP-10|mob-1	4	Cytokines
CXCL11	6373	C-X-C motif chemokine ligand 11	H174|I-TAC|IP-9|IP9|SCYB11|SCYB9B|b-R1	4	Cytokines
CXCL12	6387	C-X-C motif chemokine ligand 12	IRH|PBSF|SCYB12|SDF1|TLSF|TPAR1	10	Cytokines
CXCL13	10563	C-X-C motif chemokine ligand 13	ANGIE|ANGIE2|BCA-1|BCA1|BLC|BLR1L|SCYB13	4	Cytokines
CXCL14	9547	C-X-C motif chemokine ligand 14	BMAC|BRAK|KEC|KS1|MIP-2g|MIP2G|NJAC|SCYB14	5	Cytokines
CXCL16	58191	C-X-C motif chemokine ligand 16	CXCLG16|SR-PSOX|SRPSOX	17	Cytokines
CXCL17	284340	C-X-C motif chemokine ligand 17	DMC|Dcip1|UNQ473|VCC-1|VCC1	19	Cytokines
CXCL2	2920	C-X-C motif chemokine ligand 2	CINC-2a|GRO2|GROb|MGSA-b|MIP-2a|MIP2|MIP2A|SCYB2	4	Cytokines
CXCL3	2921	C-X-C motif chemokine ligand 3	CINC-2b|GRO3|GROg|MIP-2b|MIP2B|SCYB3	4	Cytokines
CXCL5	6374	C-X-C motif chemokine ligand 5	ENA-78|SCYB5	4	Cytokines
CXCL6	6372	C-X-C motif chemokine ligand 6	CKA-3|GCP-2|GCP2|SCYB6	4	Cytokines
CXCL9	4283	C-X-C motif chemokine ligand 9	CMK|Humig|MIG|SCYB9|crg-10	4	Cytokines
CCN1	3491	cellular communication network factor 1	CYR61|GIG1|IGFBP10	1	Cytokines
DEFA1	1667	defensin alpha 1	DEF1|DEFA2|HNP-1|HP-1|HP1|MRS	8	Cytokines
DEFA3	1668	defensin alpha 3	DEF3|HNP-3|HNP3|HP-3|HP3	8	Cytokines
DEFA5	1670	defensin alpha 5	DEF5|HD-5	8	Cytokines
DEFB1	1672	defensin beta 1	BD1|DEFB-1|DEFB101|HBD1	8	Cytokines
DEFB1	5589	defensin beta 103B	BD-3|DEFB-3|DEFB103|DEFB3|HBD-3|HBD3	8	Cytokines

03B	4		|HBP-3|HBP3		
DEFB1<br>04A	1405<br>96	defensin beta 104A	BD-4|DEFB-4|DEFB104|DEFB4|hBD-4	8	Cytokines
DEFB4<br>A	1673	defensin beta 4A	BD-2|DEFB-2|DEFB102|DEFB2|DEFB4|HBD-<br>2|SAP1	8	Cytokines
DKK1	2294<br>3	dickkopf WNT signaling pathway inhibitor 1	DKK-1|SK	10	Cytokines
EBI3	1014<br>8	Epstein-Barr virus induced 3	IL-27B|IL27B|IL35B	19	Cytokines
EDN1	1906	endothelin 1	ARCND3|ET1|HDLCQ7|PPET1|QME	6	Cytokines
EDN2	1907	endothelin 2	ET-2|ET2|PPET2	1	Cytokines
EDN3	1908	endothelin 3	ET-3|ET3|HSCR4|PPET3|WS4B	20	Cytokines
EGF	1950	epidermal growth factor	HOMG4|URG	4	Cytokines
EPGN	2553<br>24	epithelial mitogen	ALGV3072|EPG|PRO9904	4	Cytokines
EPO	2056	erythropoietin	DBAL|ECYT5|EP|MVCD2	7	Cytokines
EREG	2069	epiregulin	EPR|ER|Ep	4	Cytokines
ESM1	1108<br>2	endothelial cell specific molecule 1	endocan	5	Cytokines
FAM3B	5409<br>7	FAM3 metabolism regulating signaling molecule<br>B	2-21|C21orf11|C21orf76|ORF9|PANDER|P<br>RED44	21	Cytokines
FAM3C	1044<br>7	FAM3 metabolism regulating signaling molecule<br>C	GS3786|ILEI	7	Cytokines
FAM3D	1311<br>77	FAM3 metabolism regulating signaling molecule<br>D	EF7|OIT1	3	Cytokines
FASLG	356	Fas ligand	ALPS1B|APT1LG1|APTL|CD178|CD95-L|CD9	1	Cytokines

			5L|FASL|TNFSF6|TNLG1A			
FGF1	2246	fibroblast growth factor 1	AFGF|ECGF|ECGF-beta|ECGFA|ECGFB|FGF-1|FGF-alpha|FGFA|GLIO703|HBGF-1|HBGF1		5	Cytokines
FGF10	2255	fibroblast growth factor 10	-		5	Cytokines
FGF11	2256	fibroblast growth factor 11	FGF-11|FHF-3|FHF3		17	Cytokines
FGF12	2257	fibroblast growth factor 12	EIEE47|FGF12B|FHF1		3	Cytokines
FGF13	2258	fibroblast growth factor 13	FGF-13|FGF2|FHF-2|FHF2|LINC00889	X		Cytokines
FGF14	2259	fibroblast growth factor 14	FGF-14|FHF-4|FHF4|SCA27		13	Cytokines
FGF16	8823	fibroblast growth factor 16	FGF-16|MF4	X		Cytokines
FGF17	8822	fibroblast growth factor 17	FGF-13|FGF-17|HH20		8	Cytokines
FGF18	8817	fibroblast growth factor 18	FGF-18|ZFGF5		5	Cytokines
FGF19	9965	fibroblast growth factor 19	-		11	Cytokines
FGF2	2247	fibroblast growth factor 2	BFGF|FGF-2|FGFB|HBGF-2		4	Cytokines
FGF20	26281	fibroblast growth factor 20	FGF-20|RHDA2		8	Cytokines
FGF21	26291	fibroblast growth factor 21	-		19	Cytokines
FGF22	27006	fibroblast growth factor 22	-		19	Cytokines
FGF23	8074	fibroblast growth factor 23	ADHR|FGFN|HFTC2|HPDR2|HYPF|PHPTC		12	Cytokines
FGF3	2248	fibroblast growth factor 3	HBGF-3|INT2		11	Cytokines
FGF4	2249	fibroblast growth factor 4	FGF-4|HBGF-4|HST|HST-1|HSTF-1|HSTF1|K-FGF|KFGF		11	Cytokines
FGF5	2250	fibroblast growth factor 5	HBGF-5|Smag-82|TCMGLY		4	Cytokines
FGF6	2251	fibroblast growth factor 6	HBGF-6|HST2		12	Cytokines

FGF7	2252	fibroblast growth factor 7	HBGF-7|KGF		15	Cytokines
FGF8	2253	fibroblast growth factor 8	AIGF|FGF-8|HBGF-8|HH6|KAL6		10	Cytokines
FGF9	2254	fibroblast growth factor 9	FGF-9|GAF|HBFG-9|HBGF-9|SYNS3		13	Cytokines
VEGFD	2277	vascular endothelial growth factor D	FIGF|VEGF-D	X		Cytokines
FIGNL2	401720	fidgetin like 2	-		12	Cytokines
FLT3LG	2323	fms related receptor tyrosine kinase 3 ligand	FL|FLG3L|FLT3L		19	Cytokines
FSHB	2488	follicle stimulating hormone subunit beta	HH24		11	Cytokines
GAL	51083	galanin and GMAP prepropeptide	ETL8|GAL-GMAP|GALN|GLNN|GMAP		11	Cytokines
GALP	85569	galanin like peptide	-		19	Cytokines
GAST	2520	gastrin	GAS		17	Cytokines
GCG	2641	glucagon	GLP-1|GLP1|GLP2|GRPP		2	Cytokines
GDF1	2657	growth differentiation factor 1	CERS1|CHTD6|DORV|DTGA3|LAG1|LASS1|RAI|UOG1		19	Cytokines
GDF10	2662	growth differentiation factor 10	BIP|BMP-3b|BMP3B		10	Cytokines
GDF11	10220	growth differentiation factor 11	BMP-11|BMP11		12	Cytokines
GDF15	9518	growth differentiation factor 15	GDF-15|MIC-1|MIC1|NAG-1|PDF|PLAB|PTGFB		19	Cytokines
GDF2	2658	growth differentiation factor 2	BMP-9|BMP9|HHT5		10	Cytokines
GDF3	9573	growth differentiation factor 3	KFS3|MCOP7|MCOPCB6		12	Cytokines
GDF5	8200	growth differentiation factor 5	BDA1C|BMP-14|BMP14|CDMP1|DUPANS|LAP-4|LAP4|OS5|SYM1B|SYNS2		20	Cytokines

GDF6	392255	growth differentiation factor 6	BMP-13|BMP13|CDMP2|KFM|KFS|KFS1|KFSL|SGM1|SYNS4	8	Cytokines
GDF7	151449	growth differentiation factor 7	BMP12	2	Cytokines
GDF9	2661	growth differentiation factor 9	POF14	5	Cytokines
GDNF	2668	glial cell derived neurotrophic factor	ATF|ATF1|ATF2|HFB1-GDNF|HSCR3	5	Cytokines
GH1	2688	growth hormone 1	GH|GH-N|GHB5|GHN|IGHD1A|IGHD1B|IGHD2|hGH-N	17	Cytokines
GH2	2689	growth hormone 2	GH-V|GHB2|GHL|GHV|hGH-V	17	Cytokines
GHRH	2691	growth hormone releasing hormone	GHRF|GRF|INN	20	Cytokines
GHRL	51738	ghrelin and obestatin prepropeptide	MTLRP	3	Cytokines
GIP	2695	gastric inhibitory polypeptide	-	17	Cytokines
GKN1	56287	gastrokine 1	AMP18|BRICD1|CA11|FOV|foveolin	2	Cytokines
GMFB	2764	glia maturation factor beta	GMF	14	Cytokines
GMFG	9535	glia maturation factor gamma	GMF-GAMMA	19	Cytokines
GNRH1	2796	gonadotropin releasing hormone 1	GNRH|GRH|LHRH|LNRH	8	Cytokines
GNRH2	2797	gonadotropin releasing hormone 2	GnRH-II|LH-RHII	20	Cytokines
GPHA2	170589	glycoprotein hormone subunit alpha 2	A2|GPA2|ZSIG51	11	Cytokines
GPHB5	122876	glycoprotein hormone subunit beta 5	B5|GPB5|ZLUT1	14	Cytokines
GPI	2821	glucose-6-phosphate isomerase	AMF|GNPI|NLK|PGI|PHI|SA-36|SA36	19	Cytokines
GREM1	26585	gremlin 1, DAN family BMP antagonist	C15DUPq|CKTSF1B1|CRAC1|CRCS4|DAND2|DRM|DUP15q|GREMLIN|HMPS|HMPS1|IHG-2|M	15	Cytokines

			PSH|PIG2		
GREM2	64388	gremlin 2, DAN family BMP antagonist	CKTSF1B2|DAND3|PRDC|STHAG9	1	Cytokines
GRN	2896	granulin precursor	CLN11|GEP|GP88|PCDGF|PEPI|PGRN	17	Cytokines
GRP	2922	gastrin releasing peptide	BN|GRP-10|preproGRP|proGRP	18	Cytokines
GUCA2A	2980	guanylate cyclase activator 2A	GCAP-I|GUCA2|STARA	1	Cytokines
HAMP	57817	hepcidin antimicrobial peptide	HEPC|HFE2B|LEAP1|PLTR	19	Cytokines
HBEGF	1839	heparin binding EGF like growth factor	DTR|DTS|DTSF|HEGFL	5	Cytokines
HDGF	3068	heparin binding growth factor	HMG1L2	1	Cytokines
HDGFL3	50810	HDGF like 3	CGI-142|HDGF-2|HDGF2|HDGFRP3|HRP-3	15	Cytokines
HGF	3082	hepatocyte growth factor	DFNB39|F-TCF|HGFB|HPTA|SF	7	Cytokines
HTN3	3347	histatin 3	HIS2|HTN2|HTN5|PB	4	Cytokines
IAPP	3375	islet amyloid polypeptide	DAP|IAP	12	Cytokines
IFNA1	3439	interferon alpha 1	IFL|IFN|IFN-ALPHA|IFN-alphaD|IFNA13|IFNA@|leIF D	9	Cytokines
IFNA10	3446	interferon alpha 10	IFN-alphaC	9	Cytokines
IFNA13	3447	interferon alpha 13	-	9	Cytokines
IFNA14	3448	interferon alpha 14	IFN-alphaH|LEIF2H	9	Cytokines
IFNA16	3449	interferon alpha 16	IFN-alpha-16|IFN-alphaO	9	Cytokines

IFNA17	3451	interferon alpha 17	IFN-alphaI|IFNA|INFA|LEIF2C1	9	Cytokines
IFNA2	3440	interferon alpha 2	IFN-alpha-2|IFN-alphaA|IFNA|IFNA2B|leIF A	9	Cytokines
IFNA21	3452	interferon alpha 21	IFN-alphaI|LeIF F|leIF-F	9	Cytokines
IFNA4	3441	interferon alpha 4	IFN-alpha4a|INFA4	9	Cytokines
IFNA5	3442	interferon alpha 5	IFN-alpha-5|IFN-alphaG|INA5|INFA5|leIF G	9	Cytokines
IFNA6	3443	interferon alpha 6	IFN-alphaK	9	Cytokines
IFNA7	3444	interferon alpha 7	IFN-alphaJ|IFNA-J	9	Cytokines
IFNA8	3445	interferon alpha 8	IFN-alphaB	9	Cytokines
IFNB1	3456	interferon beta 1	IFB|IFF|IFN-beta|IFNB	9	Cytokines
IFNE	338376	interferon epsilon	IFN-E|IFNE1|IFNT1|INFE1|PRO655	9	Cytokines
IFNG	3458	interferon gamma	IFG|IFI	12	Cytokines
IFNK	56832	interferon kappa	IFNT1|INFE1	9	Cytokines
IFNW1	3467	interferon omega 1	-	9	Cytokines
IGF1	3479	insulin like growth factor 1	IGF|IGF-I|IGFI|MGF	12	Cytokines
IGF2	3481	insulin like growth factor 2	C11orf43|GRDF|IGF-II|PP9974	11	Cytokines
IL10	3586	interleukin 10	CSIF|GVHDS|IL-10|IL10A|TGIF	1	Cytokines
IL11	3589	interleukin 11	AGIF|IL-11	19	Cytokines
IL12A	3592	interleukin 12A	CLMF|IL-12A|NFSK|NKSF1|P35	3	Cytokines
IL12B	3593	interleukin 12B	CLMF|CLMF2|IL-12B|IMD28|IMD29|NKSF|NKSF2	5	Cytokines

IL13	3596	interleukin 13	IL-13|P600	5	Cytokines
IL15	3600	interleukin 15	IL-15	4	Cytokines
IL16	3603	interleukin 16	LCF|NIL16|PRIL16|prIL-16	15	Cytokines
IL17A	3605	interleukin 17A	CTLA-8|CTLA8|IL-17|IL-17A|IL17	6	Cytokines
IL17B	27190	interleukin 17B	IL-17B|IL-20|NIRF|ZCYTO7	5	Cytokines
IL17C	27189	interleukin 17C	CX2|IL-17C	16	Cytokines
IL17D	53342	interleukin 17D	IL-17D	13	Cytokines
IL17F	112744	interleukin 17F	CANDF6|IL-17F|ML-1|ML1	6	Cytokines
IL18	3606	interleukin 18	IGIF|IL-18|IL-1g|IL1F4	11	Cytokines
IL19	29949	interleukin 19	IL-10C|MDA1|NG.1|ZMDA1	1	Cytokines
IL1A	3552	interleukin 1 alpha	IL-1 alpha|IL-1A|IL1|IL1-ALPHA|IL1F1	2	Cytokines
IL1B	3553	interleukin 1 beta	IL-1|IL1-BETA|IL1F2|IL1beta	2	Cytokines
IL1F10	84639	interleukin 1 family member 10	FIL1-theta|FKSG75|IL-1HY2|IL-38|IL1-theta|IL1HY2	2	Cytokines
IL36RN	26525	interleukin 36 receptor antagonist	FIL1|FIL1(DELTA)|FIL1D|IL-36Ra|IL1F5|IL1HY1|IL1L1|IL1RP3|IL36RA|PSORP|PSORS14	2	Cytokines
IL36A	27179	interleukin 36 alpha	FIL1|FIL1(EPSILON)|FIL1E|IL-1F6|IL1(EPSILON)|IL1F6	2	Cytokines
IL37	27178	interleukin 37	FIL1|FIL1(ZETA)|FIL1Z|IL-1F7|IL-1H|IL-1H4|IL-1RP1|IL-37|IL1F7|IL1H4|IL1R	2	Cytokines

			P1		
IL36B	27177	interleukin 36 beta	FIL1|FIL1-(ETA)|FIL1H|FILI-(ETA)|IL-1F8|IL-1H2|IL1-ETA|IL1F8|IL1H2	2	Cytokines
IL36G	56300	interleukin 36 gamma	IL-1F9|IL-1H1|IL-1RP2|IL1E|IL1F9|IL1H1|IL1RP2	2	Cytokines
IL1RN	3557	interleukin 1 receptor antagonist	DIRA|ICIL-1RA|IL-1RN|IL-1ra|IL-1ra3|IL1F3|IL1RA|IRAP|MVCD4	2	Cytokines
IL2	3558	interleukin 2	IL-2|TCGF|lymphokine	4	Cytokines
IL20	50604	interleukin 20	IL-20|IL10D|ZCYTO10	1	Cytokines
IL21	59067	interleukin 21	CVID11|IL-21|Za11	4	Cytokines
IL22	50616	interleukin 22	IL-21|IL-22|IL-D110|IL-TIF|ILTIF|TIFIL-23|TIFa|zcyto18	12	Cytokines
IL23A	51561	interleukin 23 subunit alpha	IL-23|IL-23A|IL23P19|P19|SGRF	12	Cytokines
IL24	11009	interleukin 24	C49A|FISP|IL10B|MDA7|MOB5|ST16	1	Cytokines
IL25	64806	interleukin 25	IL17E	14	Cytokines
IL26	55801	interleukin 26	AK155|IL-26	12	Cytokines
IL27	246778	interleukin 27	IL-27|IL-27A|IL27A|IL27p28|IL30|p28	16	Cytokines
IFNL2	282616	interferon lambda 2	IL-28A|IL28A	19	Cytokines

IFNL3	282617	interferon lambda 3	IFN-lambda-3|IFN-lambda-4|IL-28B|IL-28C|IL28B|IL28C	19	Cytokines
IFNL1	282618	interferon lambda 1	IL-29|IL29	19	Cytokines
IL3	3562	interleukin 3	IL-3|MCGF|MULTI-CSF	5	Cytokines
IL31	386653	interleukin 31	IL-31	12	Cytokines
IL32	9235	interleukin 32	IL-32alpha|IL-32beta|IL-32delta|IL-32gamma|NK4|TAIF|TAIFa|TAIFb|TAIFc|TAIFd	16	Cytokines
IL33	90865	interleukin 33	C9orf26|DVS27|IL1F11|NF-HEV|NFEHEV	9	Cytokines
IL34	146433	interleukin 34	C16orf77|IL-34	16	Cytokines
IL4	3565	interleukin 4	BCGF-1|BCGF1|BSF-1|BSF1|IL-4	5	Cytokines
IL5	3567	interleukin 5	EDF|IL-5|TRF	5	Cytokines
IL6	3569	interleukin 6	BSF-2|BSF2|CDF|HGF|HSF|IFN-beta-2|IFNB2|IL-6	7	Cytokines
IL6ST	3572	interleukin 6 signal transducer	CD130|CDW130|GP130|HIES4|IL-6RB|sGP130	5	Cytokines
IL7	3574	interleukin 7	IL-7	8	Cytokines
CXCL8	3576	C-X-C motif chemokine ligand 8	GCP-1|GCP1|IL8|LECT|LUCT|LYNAP|MDNCF|MONAP|NAF|NAP-1|NAP1|SCYB8	4	Cytokines
IL9	3578	interleukin 9	HP40|IL-9|P40	5	Cytokines
INHA	3623	inhibin subunit alpha	-	2	Cytokines
INHBA	3624	inhibin subunit beta A	EDF|FRP	7	Cytokines

INHBB	3625	inhibin subunit beta B	-	2	Cytokines
INHBC	3626	inhibin subunit beta C	IHBC	12	Cytokines
INHBE	83729	inhibin subunit beta E	-	12	Cytokines
INS	3630	insulin	IDDM|IDDM1|IDDM2|ILPR|IRDN|MODY10|PNDM4	11	Cytokines
INS-IGF2	723961	INS-IGF2 readthrough	INSIGF	11	Cytokines
INSL3	3640	insulin like 3	RLF|RLNL|ley-I-L	19	Cytokines
INSL4	3641	insulin like 4	EPIL|PLACENTIN	9	Cytokines
INSL5	10022	insulin like 5	PRO182|UNQ156	1	Cytokines
INSL6	11172	insulin like 6	RIF1	9	Cytokines
JAG1	182	jagged canonical Notch ligand 1	AGS|AGS1|AHD|AWS|CD339|DCHE|HJ1|JAGL1	20	Cytokines
JAG2	3714	jagged canonical Notch ligand 2	HJ2|SER2	14	Cytokines
FGF7P6	387628	fibroblast growth factor 7 pseudogene 6	KGFLP1	9	Cytokines
FGF7P3	654466	fibroblast growth factor 7 pseudogene 3	KGFLP2	9	Cytokines
KITLG	4254	KIT ligand	DCUA|DFNA69|FPH2|FPHH|KL-1|Kitl|MGF|SCF|SF|SHEP7|SLF	12	Cytokines
KL	9365	klotho	HFTC3	13	Cytokines
LACRT	90070	lacritin	-	12	Cytokines

LECT2	3950	leukocyte cell derived chemotaxin 2	chm-II|chm2	5	Cytokines
LEFTY 1	1063 7	left-right determination factor 1	LEFTB|LEFTYB	1	Cytokines
LEFTY 2	7044	left-right determination factor 2	EBAF|LEFTA|LEFTYA|TGFB4	1	Cytokines
LEP	3952	leptin	LEPD|OB|OBS	7	Cytokines
LHB	3972	luteinizing hormone subunit beta	CGB4|HH23|LSH-B|LSH-beta	19	Cytokines
LIF	3976	LIF interleukin 6 family cytokine	CDF|DIA|HILDA|MLPLI	22	Cytokines
LRSAM 1	9067 8	leucine rich repeat and sterile alpha motif containing 1	CMT2P|RIFLE|TAL	9	Cytokines
LTA	4049	lymphotoxin alpha	LT|TNFB|TNFSF1|TNLG1E	6	Cytokines
LTB	4050	lymphotoxin beta	TNFC|TNFSF3|TNLG1C|p33	6	Cytokines
LTBP1	4052	latent transforming growth factor beta binding protein 1	-	2	Cytokines
LTBP2	4053	latent transforming growth factor beta binding protein 2	C14orf141|GLC3D|LTBP3|MSPKA|MSTP031|WMS3	14	Cytokines
LTBP3	4054	latent transforming growth factor beta binding protein 3	DASS|GPHYSD3|LTBP-3|LTBP2|STHAG6|pp6425	11	Cytokines
LTBP4	8425	latent transforming growth factor beta binding protein 4	ARCL1C|LTBP-4|LTBP4L|LTBP4S	19	Cytokines
MDK	4192	midkine	ARAP|MK|NEGF2	11	Cytokines
MIA	8190	MIA SH3 domain containing	CD-RAP	19	Cytokines
MIF	4282	macrophage migration inhibitory factor	GIF|GLIF|MMIF	22	Cytokines
MLN	4295	motilin	-	6	Cytokines
MSTN	2660	myostatin	GDF8|MSLHP	2	Cytokines
NAMPT	1013	nicotinamide phosphoribosyltransferase	1110035014Rik|PBEF|PBEF1|VF|VISFATIN	7	Cytokines

	5				
NDP	4693	norrin cystine knot growth factor NDP	EVR2|FEVR|ND	X	Cytokines
NENF	29937	neudesin neurotrophic factor	CIR2|SCIRP10|SPUF	1	Cytokines
NGF	4803	nerve growth factor	Beta-NGF|HSAN5|NGFB	1	Cytokines
NMB	4828	neuromedin B	-	15	Cytokines
NODAL	4838	nodal growth differentiation factor	HTX5	10	Cytokines
CCN3	4856	cellular communication network factor 3	IBP-9|IGFBP-9|IGFBP9|NOV|NOVh	8	Cytokines
NPFF	8620	neuropeptide FF-amide peptide precursor	FMRFAL	12	Cytokines
NPPA	4878	natriuretic peptide A	ANF|ANP|ATFB6|ATRST2|CDD|CDD-ANF|CDP|PND	1	Cytokines
NPPB	4879	natriuretic peptide B	BNP	1	Cytokines
NPPC	4880	natriuretic peptide C	CNP|CNP2	2	Cytokines
NPY	4852	neuropeptide Y	PYY4	7	Cytokines
NRG1	3084	neuregulin 1	ARIA|GGF|GGF2|HGL|HRG|HRG1|HRGA|MST131|MSTP131|NDF|NRG1-IT2|SMDF	8	Cytokines
NRG2	9542	neuregulin 2	DON1|HRG2|NTAK	5	Cytokines
NRG3	10718	neuregulin 3	HRG3|pro-NRG3	10	Cytokines
NRG4	145957	neuregulin 4	HRG4	15	Cytokines
NRTN	4902	neurturin	NTN	19	Cytokines
NTF3	4908	neurotrophin 3	HDNF|NGF-2|NGF2|NT-3|NT3	12	Cytokines
NTF4	4909	neurotrophin 4	GLC10|GLC1O|NT-4|NT-4/5|NT-5|NT4|NT5|NTF5	19	Cytokines
NTS	4922	neurotensin	NMN-125|NN|NT|NT/N|NTS1	12	Cytokines

NUDT6	11162	nudix hydrolase 6	ASFGF2|FGF-AS|FGF2AS|GFG-1|GFG1	4	Cytokines
OGN	4969	osteoglycin	OG|OIF|SLRR3A	9	Cytokines
OSGIN1	29948	oxidative stress induced growth inhibitor 1	BDGI|OKL38	16	Cytokines
OSM	5008	oncostatin M	-	22	Cytokines
OSTN	344901	osteocrin	MUSCLIN	3	Cytokines
OXT	5020	oxytocin/neurophysin I prepropeptide	OT|OT-NPI|OXT-NPI	20	Cytokines
ENDOU	8909	endonuclease, poly(U) specific	P11|PP11|PRSS26	12	Cytokines
PDGFA	5154	platelet derived growth factor subunit A	PDGF-A|PDGF1	7	Cytokines
PDGFB	5155	platelet derived growth factor subunit B	IBGC5|PDGF-2|PDGF2|SIS|SSV|c-sis	22	Cytokines
PDGFC	56034	platelet derived growth factor C	FALLOTEIN|SCDGF	4	Cytokines
PDGFD	80310	platelet derived growth factor D	IEGF|MSTP036|SCDGF-B|SCDGFB	11	Cytokines
PDGFRA	5156	platelet derived growth factor receptor alpha	CD140A|PDGFR-2|PDGFR2	4	Cytokines
PDGFRB	5159	platelet derived growth factor receptor beta	CD140B|IBGC4|IMF1|JTK12|KOGS|PDGFR|PDGFR-1|PDGFR1|PENTT	5	Cytokines
PDGFRL	5157	platelet derived growth factor receptor like	PDGRL|PRLTS	8	Cytokines
PDYN	5173	prodynorphin	ADCA|PENKB|SCA23	20	Cytokines
PENK	5179	proenkephalin	PE|PENK-A	8	Cytokines
PF4	5196	platelet factor 4	CXCL4|PF-4|SCYB4	4	Cytokines
PF4V1	5197	platelet factor 4 variant 1	CXCL4L1|CXCL4V1|PF4-ALT|PF4A|SCYB4V1	4	Cytokines

PGF	5228	placental growth factor	D12S1900|PGFL|PIGF|PLGF|PlGF-2|SHGC-10760	14	Cytokines
PLAU	5328	plasminogen activator, urokinase	ATF|BDPLT5|QPD|UPA|URK|u-PA	10	Cytokines
PMCH	5367	pro-melanin concentrating hormone	MCH|ppMCH	12	Cytokines
PNOC	5368	prepronociceptin	N/OFQ|NOP|OFQ|PPNOC|ppN/OFQ	8	Cytokines
POMC	5443	proopiomelanocortin	ACTH|CLIP|LPH|MSH|NPP|OBAIRH|POC	2	Cytokines
PPBP	5473	pro-platelet basic protein	B-TG1|Beta-TG|CTAP-III|CTAP3|CTAPIII|CXCL7|LA-PF4|LDGF|MDGF|NAP-2|PBP|SCYB7|TC1|TC2|TGB|TGB1|THBGB|THBGB1	4	Cytokines
PPBPP1	728045	pro-platelet basic protein pseudogene 1	PPBPL1|TGB2	4	Cytokines
PPBPP2	10895	pro-platelet basic protein pseudogene 2	PPBPL2|SPBPBP	4	Cytokines
PPY	5539	pancreatic polypeptide	PNP|PP	17	Cytokines
PRL	5617	prolactin	GHA1	6	Cytokines
PRLH	51052	prolactin releasing hormone	PRH|PRRP	2	Cytokines
PROK1	84432	prokineticin 1	EGVEGF|PK1|PRK1	1	Cytokines
PROK2	60675	prokineticin 2	BV8|HH4|KAL4|MIT1|PK2	3	Cytokines
PSPN	5623	persephin	PSP	19	Cytokines
PTH	5741	parathyroid hormone	FIH1|PTH1	11	Cytokines
PTH2	113091	parathyroid hormone 2	TIP39	19	Cytokines
PTHLH	5744	parathyroid hormone like hormone	BDE2|HHM|PLP|PTHR|PTHRP	12	Cytokines

PTN	5764	pleiotrophin	HARP|HB-GAM|HBBM|HBGF-8|HBGF8|HBNF|HBNF-1|NEGF1|OSF-1	7	Cytokines
PYY	5697	peptide YY	PYY-I|PYY1	17	Cytokines
QRFP	347148	pyroglutamylated RFamide peptide	26RFa|P518	9	Cytokines
RABEP1	9135	rabaptin, RAB GTPase binding effector protein 1	RAB5EP|RABPT5	17	Cytokines
RABEP2	79874	rabaptin, RAB GTPase binding effector protein 2	FRA	16	Cytokines
REG1A	5967	regenerating family member 1 alpha	ICRF|P19|PSP|PSPS|PSPS1|PTP|REG	2	Cytokines
RETN	56729	resistin	ADSF|FIZZ3|RETN1|RSTN|XCP1	19	Cytokines
RETNLB	84666	resistin like beta	FIZZ1|FIZZ2|HXCP2|RELM-beta|RELMb|RELMbeta|XCP2	3	Cytokines
RLN1	6013	relaxin 1	H1|H1RLX|RLXH1|bA12D24.3.1|bA12D24.3.2	9	Cytokines
RLN2	6019	relaxin 2	H2|H2-RLX|RLXH2|bA12D24.1.1|bA12D24.1.2	9	Cytokines
RLN3	117579	relaxin 3	H3|RXN3|ZINS4|insl7	19	Cytokines
RNASE2	6036	ribonuclease A family member 2	EDN|RAF3|RNS2	14	Cytokines
S100A6	6277	S100 calcium binding protein A6	2A9|5B10|CABP|CACY|PRA|S10A6	1	Cytokines
SAA1	6288	serum amyloid A1	PIG4|SAA|SAA2|TP53I4	11	Cytokines
SAA2	6289	serum amyloid A2	SAA|SAA1	11	Cytokines

SBDS	51119	SBDS ribosome maturation factor	CGI-97|SDS|SWDS	7	Cytokines
SCG2	7857	secretogranin II	CHGC|EM66|SN|SgII	2	Cytokines
SCGB3A1	92304	secretoglobin family 3A member 1	HIN-1|HIN1|LU105|PnSP-2|UGRP2	5	Cytokines
SCT	6343	secretin	-	11	Cytokines
AIMP1	9255	aminoacyl tRNA synthetase complex interacting multifunctional protein 1	EMAP2|EMAPII|HLD3|SCYE1|p43	4	Cytokines
SECTM1	6398	secreted and transmembrane 1	K12|SECTM	17	Cytokines
SEMA3A	10371	semaphorin 3A	COLL1|HH16|Hsema-I|Hsema-III|SEMA1|SEMAD|SEMAIII|SEMAL|SemD|coll-1	7	Cytokines
SEMA3B	7869	semaphorin 3B	LUCA-1|SEMA5|SEMAA|SemA|semaV	3	Cytokines
SEMA3C	10512	semaphorin 3C	SEMAE|SemE	7	Cytokines
SEMA3D	223117	semaphorin 3D	Sema-Z2|coll-2	7	Cytokines
SEMA3E	9723	semaphorin 3E	M-SEMAH|M-SemaK|SEMAH|coll-5	7	Cytokines
SEMA3F	6405	semaphorin 3F	SEMA-IV|SEMA4|SEMAK	3	Cytokines
SEMA3G	56920	semaphorin 3G	sem2	3	Cytokines
SEMA4A	64218	semaphorin 4A	CORD10|RP35|SEMAB|SEMB	1	Cytokines

SEMA4B	10509	semaphorin 4B	SEMAC|SemC	15	Cytokines
SEMA4C	54910	semaphorin 4C	M-SEMA-F|SEMACL1|SEMAF|SEMAI	2	Cytokines
SEMA4D	10507	semaphorin 4D	A8|BB18|C9orf164|CD100|COLL4|GR3|M-sema-G|SEMAJ|coll-4	9	Cytokines
SEMA4F	10505	ssemaphorin 4F	M-SEMA|PRO2353|S4F|SEMAM|SEMAW|m-Sema-M	2	Cytokines
SEMA4G	57715	semaphorin 4G	-	10	Cytokines
SEMA5A	9037	semaphorin 5A	SEMAF|semF	5	Cytokines
SEMA5B	54437	semaphorin 5B	SEMAG|SemG	3	Cytokines
SEMA6A	57556	semaphorin 6A	HT018|SEMA|SEMA6A1|SEMAQ|VIA	5	Cytokines
SEMA6B	10501	semaphorin 6B	EPM11|SEM-SEMA-Y|SEMA-VIB|SEMAN|semaZ	19	Cytokines
SEMA6C	10500	semaphorin 6C	SEMAY|m-SemaY|m-SemaY2	1	Cytokines
SEMA6D	80031	semaphorin 6D	-	15	Cytokines
SEMA7A	8482	semaphorin 7A (John Milton Hagen blood group)	CD108|CDw108|H-SEMA-K1|H-Sema-L|JMH|SEMAK1|SEMAL	15	Cytokines
SLIT1	6585	slit guidance ligand 1	MEGF4|SLIL1|SLIT-1|SLIT3	10	Cytokines
SLIT2	9353	slit guidance ligand 2	SLIL3|Slit-2	4	Cytokines

SLURP1	57152	secreted LY6/PLAUR domain containing 1	ANUP|ARS|ArsB|LY6-MT|LY6LS|MDM		8	Cytokines
SPP1	6696	secreted phosphoprotein 1	BNSP|BSPI|ETA-1|OPN		4	Cytokines
SST	6750	somatostatin	SMST		3	Cytokines
STC1	6781	stanniocalcin 1	STC		8	Cytokines
STC2	8614	stanniocalcin 2	STC-2|STCRP		5	Cytokines
TAC1	6863	tachykinin precursor 1	Hs.2563|NK2|NKNA|NPK|TAC2		7	Cytokines
TDGF1	6997	teratocarcinoma-derived growth factor 1	CR|CR-1|CRGF|CRIPTO		3	Cytokines
TDGF1P3	6998	teratocarcinoma-derived growth factor 1 pseudogene 3	CR-3|CRIPTO|CRIPTO-3|CRIPTO3|TDGF1|TDGF2|TDGF3	X		Cytokines
TG	7038	thyroglobulin	AITD3|TGN		8	Cytokines
TGFA	7039	transforming growth factor alpha	TFGA		2	Cytokines
TGFB1	7040	transforming growth factor beta 1	CED|DPD1|IBDIMDE|LAP|TGF-beta1|TGFB|TGFbeta		19	Cytokines
TGFB2	7042	transforming growth factor beta 2	G-TSF|LDS4|TGF-beta2		1	Cytokines
TGFB3	7043	transforming growth factor beta 3	ARVD|ARVD1|LDS5|RNHF|TGF-beta3		14	Cytokines
THPO	7066	thrombopoietin	MGDF|MKCSF|ML|MPLLG|THCYT1|TPO		3	Cytokines
TNC	3371	tenascin C	150-225|DFNA56|GMEM|GP|HXB|JI|TN|TN-C		9	Cytokines
TNF	7124	tumor necrosis factor	DIF|TNF-alpha|TNFA|TNFSF2|TNLG1F		6	Cytokines
TNFRSF11B	4982	TNF receptor superfamily member 11b	OCIF|OPG|PDB5|TR1		8	Cytokines
TNFSF10	8743	TNF superfamily member 10	APO2L|Apo-2L|CD253|TL2|TNLG6A|TRAIL		3	Cytokines
TNFSF11	8600	TNF superfamily member 11	CD254|ODF|OPGL|OPTB2|RANKL|TNLG6B|TRANCE|hRANKL2|sOdf		13	Cytokines

TNFSF12	8742	TNF superfamily member 12	APO3L|DR3LG|TNLG4A|TWEAK	17	Cytokines
TNFSF13	8741	TNF superfamily member 13	APRIL|CD256|TALL-2|TALL2|TNLG7B|TRDL-1|UNQ383/PRO715|ZTNF2	17	Cytokines
TNFSF13B	10673	TNF superfamily member 13b	BAFF|BLYS|CD257|DTL|TALL-1|TALL1|THANK|TNFSF20|TNLG7A|ZTNF4	13	Cytokines
TNFSF14	8740	TNF superfamily member 14	CD258|HVEML|LIGHT|LTg	19	Cytokines
TNFSF15	9966	TNF superfamily member 15	TL1|TL1A|TNLG1B|VEGI|VEGI192A	9	Cytokines
TNFSF18	8995	TNF superfamily member 18	AITRL|GITRL|TL6|TNLG2A|hGITRL	1	Cytokines
TNFSF4	7292	TNF superfamily member 4	CD134L|CD252|GP34|OX-40L|OX4OL|TNLG2B|TXGP1	1	Cytokines
TNFSF8	944	TNF superfamily member 8	CD153|CD30L|CD30LG|TNLG3A	9	Cytokines
TNFSF9	8744	TNF superfamily member 9	4-1BB-L|CD137L|TNLG5A	19	Cytokines
TOR2A	27433	torsin family 2 member A	TORP1	9	Cytokines
TRH	7200	thyrotropin releasing hormone	Pro-TRH|TRF	3	Cytokines
TSHB	7252	thyroid stimulating hormone subunit beta	TSH-B|TSH-BETA	1	Cytokines
TSLP	85480	thymic stromal lymphopoietin	-	5	Cytokines
TXLNA	200081	taxilin alpha	IL14|TXLN	1	Cytokines

TYMP	1890	thymidine phosphorylase	ECGF|ECGF1|MEDPS1|MNGIE|MTDPS1|PDECGF|TP|hPD-ECGF	22	Cytokines
UCN	7349	urocortin	UI|UROC	2	Cytokines
UCN2	90226	urocortin 2	SRP|UCN-II|UCNI|UR|URP	3	Cytokines
UCN3	114131	urocortin 3	SCP|SPC|UCNIII	10	Cytokines
UTS2	10911	urotensin 2	PRO1068|U-II|UCN2|UII	1	Cytokines
UTS2B	257313	urotensin 2B	U2B|URP|UTS2D	3	Cytokines
VEGFA	7422	vascular endothelial growth factor A	MVCD1|VEGF|VPF	6	Cytokines
VEGFB	7423	vascular endothelial growth factor B	VEGFL|VRF	11	Cytokines
VEGFC	7424	vascular endothelial growth factor C	Flt4-L|LMPH1D|LMPHM4|VRP	4	Cytokines
VGF	7425	VGF nerve growth factor inducible	SCG7|SgVII	7	Cytokines
VIP	7432	vasoactive intestinal peptide	PHM27	6	Cytokines
XCL1	6375	X-C motif chemokine ligand 1	ATAC|LPTN|LTN|SCM-1|SCM-1a|SCM1|SCM1A|SCYC1	1	Cytokines
XCL2	6846	X-C motif chemokine ligand 2	SCM-1b|SCM1B|SCYC2	1	Cytokines
ACVR1B	91	activin A receptor type 1B	ACTRIB|ACVRLK4|ALK4|SKR2	12	Cytokine_Receptors
ACVR1C	130399	activin A receptor type 1C	ACVRLK7|ALK7	2	Cytokine_Receptors
ACVR2A	92	activin A receptor type 2A	ACTRII|ACVR2	2	Cytokine_Receptors
ACVR2	93	activin A receptor type 2B	ACTRIIB|ActR-IIB|HTX4	3	Cytokine_Receptors

B					
ACVRL1	94	activin A receptor like type 1	ACVRLK1|ALK-1|ALK1|HHT|HHT2|ORW2|SKR3|TSR-I	12	Cytokine_Receptors
ADCYAP1R1	117	ADCYAP receptor type I	PAC1|PAC1R|PACAPR|PACAPRI	7	Cytokine_Receptors
ADIPOR1	51094	adiponectin receptor 1	ACDCR1|CGI-45|CGI45|PAQR1|TESBP1A	1	Cytokine_Receptors
ADIPOR2	79602	adiponectin receptor 2	ACDCR2|PAQR2	12	Cytokine_Receptors
ADRB1	153	adrenoceptor beta 1	ADRB1R|B1AR|BETA1AR|FNSS2|RHR	10	Cytokine_Receptors
ADRB2	154	adrenoceptor beta 2	ADRB2R|ADRBR|B2AR|BAR|BETA2AR	5	Cytokine_Receptors
AGTR1	185	angiotensin II receptor type 1	AG2S|AGTR1B|AT1|AT1AR|AT1B|AT1BR|AT1R|AT2R1|HAT1R	3	Cytokine_Receptors
AGTR2	186	angiotensin II receptor type 2	AT2|ATGR2|MRX88	X	Cytokine_Receptors
AMHR2	269	anti-Mullerian hormone receptor type 2	AMHR|MISR2|MISRII|MRII	12	Cytokine_Receptors
ANGPT1	284	angiopoietin 1	AGP1|AGPT|ANG1	8	Cytokine_Receptors
ANGPT4	51378	angiopoietin 4	ANG3|ANG4	20	Cytokine_Receptors
ANGPTL1	9068	angiopoietin like 1	ANG3|ANGPT3|ARP1|AngY|UNQ162|dJ595C2.2	1	Cytokine_Receptors
ANGPTL2	23452	angiopoietin like 2	ARP2|HARP	9	Cytokine_Receptors
ANGPTL3	27329	angiopoietin like 3	ANG-5|ANGPT5|ANL3|FHBL2	1	Cytokine_Receptors
ANGPT	5112	angiopoietin like 4	ARP4|FIAF|HARP|HFARP|NL2|PGAR|TGQTL|	19	Cytokine_Receptors

L4	9		UNQ171|pp1158		
ANGPTL6	83854	angiopoietin like 6	AGF|ARP5	19	Cytokine_Receptors
APLNR	187	apelin receptor	AGTRL1|APJ|APJR|HG11	11	Cytokine_Receptors
AR	367	androgen receptor	AIS|AR8|DHTR|HUMARA|HYSP1|KD|NR3C4|SBMA|SMAX1|TFM	X	Cytokine_Receptors
AVPR1A	552	arginine vasopressin receptor 1A	AVPR V1a|AVPR1|V1aR	12	Cytokine_Receptors
AVPR1B	553	arginine vasopressin receptor 1B	AVPR3|V1bR	1	Cytokine_Receptors
AVPR2	554	arginine vasopressin receptor 2	ADHR|DI1|DIR|DIR3|NDI|V2R	X	Cytokine_Receptors
BMPR1A	657	bone morphogenetic protein receptor type 1A	10q23del|ACVRLK3|ALK3|CD292|SKR5	10	Cytokine_Receptors
BMPR1B	658	bone morphogenetic protein receptor type 1B	ALK-6|ALK6|AMDD|BDA1D|BDA2|CDw293	4	Cytokine_Receptors
BMPR2	659	bone morphogenetic protein receptor type 2	BMPR-II|BMPR3|BMR2|BRK-3|POVD1|PPH1|T-ALK	2	Cytokine_Receptors
BRD8	10902	bromodomain containing 8	SMAP|SMAP2|p120	5	Cytokine_Receptors
C3AR1	719	complement C3a receptor 1	AZ3B|C3AR|HNFAG09	12	Cytokine_Receptors
C5AR1	728	complement C5a receptor 1	C5A|C5AR|C5R1|CD88	19	Cytokine_Receptors
CALCR	799	calcitonin receptor	CRT|CT-R|CTR|CTR1	7	Cytokine_Receptors
CALCRL	10203	calcitonin receptor like receptor	CGRPR|CRLR|LMPHM8	2	Cytokine_Receptors
ACKR2	1238	atypical chemokine receptor 2	CCBP2|CCR10|CCR9|CMKBR9|D6|hD6	3	Cytokine_Receptors
CCR1	1230	C-C motif chemokine receptor 1	CD191|CKR-1|CKR1|CMKBR1|HM145|MIP1aR	3	Cytokine_Receptors

			|SCYAR1		
CCR10	2826	C-C motif chemokine receptor 10	GPR2	17	Cytokine_Receptors
CCR3	1232	C-C motif chemokine receptor 3	C C CKR3|CC-CKR-3|CD193|CKR 3|CKR3|CMKBR3	3	Cytokine_Receptors
CCR4	1233	C-C motif chemokine receptor 4	CC-CKR-4|CD194|CKR4|CMKBR4|ChemR13|HGCN:14099|K5-5	3	Cytokine_Receptors
CCR5	1234	C-C motif chemokine receptor 5	CC-CKR-5|CCCKR5|CCR-5|CD195|CKR-5|CKR5|CMKBR5|IDDM22	3	Cytokine_Receptors
CCR6	1235	C-C motif chemokine receptor 6	BN-1|C-C CKR-6|CC-CKR-6|CCR-6|CD196|CKR-L3|CKRL3|CMKBR6|DCR2|DRY6|GPR29|GPRCY4|STRL22	6	Cytokine_Receptors
CCR7	1236	C-C motif chemokine receptor 7	BLR2|CC-CKR-7|CCR-7|CD197|CDw197|CMKBR7|EBI1	17	Cytokine_Receptors
CCR8	1237	C-C motif chemokine receptor 8	CC-CKR-8|CCR-8|CDw198|CKRL1|CMKBR8|CMKBRL2|CY6|GPRCY6|TER1	3	Cytokine_Receptors
CCR9	10803	C-C motif chemokine receptor 9	CC-CKR-9|CDw199|GPR-9-6|GPR28	3	Cytokine_Receptors
ACKR4	51554	atypical chemokine receptor 4	CC-CKR-11|CCBP2|CCR-11|CCR10|CCR11|CCRL1|CCX CKR|CCX-CKR|CKR-11|PPR1|VSHK1	3	Cytokine_Receptors
CCRL2	9034	C-C motif chemokine receptor like 2	ACKR5|CKRX|CRAM|CRAM-A|CRAM-B|HCR	3	Cytokine_Receptors
CD40	958	CD40 molecule	Bp50|CDW40|TNFRSF5|p50	20	Cytokine_Receptors
CMKLR1	1240	chemerin chemokine-like receptor 1	CHEMERINR|ChemR23|DEZ|RVER1	12	Cytokine_Receptors
CNTFR	1271	ciliary neurotrophic factor receptor	-	9	Cytokine_Receptors

CRHR1	1394	corticotropin releasing hormone receptor 1	CRF-R|CRF-R-1|CRF-R1|CRF1|CRFR-1|CRFR1|CRH-R-1|CRH-R1|CRHR|CRHR1L		17	Cytokine_Receptors
CRHR2	1395	corticotropin releasing hormone receptor 2	CRF-RB|CRF2|CRFR2|HM-CRF		7	Cytokine_Receptors
CRIM1	51232	cysteine rich transmembrane BMP regulator 1	CRIM-1|S52		2	Cytokine_Receptors
CRLF1	9244	cytokine receptor like factor 1	CISS|CISS1|CLF|CLF-1|NR6|zcytor5		19	Cytokine_Receptors
CRLF2	64109	cytokine receptor like factor 2	CRL2|CRLF2Y|TSLPR	X|Y		Cytokine_Receptors
CRLF3	51379	cytokine receptor like factor 3	CREME-9|CREME9|CRLM9|CYTOR4|FRWS|p48.2		17	Cytokine_Receptors
CSF1R	1436	colony stimulating factor 1 receptor	BANDDOS|C-FMS|CD115|CSF-1R|CSFR|FIM2|FMS|HDLS|M-CSF-R		5	Cytokine_Receptors
CSF2RA	1438	colony stimulating factor 2 receptor subunit alpha	CD116|CDw116|CSF2R|CSF2RAX|CSF2RAY|CSF2RX|CSF2RY|GM-CSF-R-alpha|GMCSFR|GMCSFR-alpha|GMR|GMR-alpha|SMDP4|alphaGMR	X|Y		Cytokine_Receptors
CSF2RB	1439	colony stimulating factor 2 receptor subunit beta	CD131|CDw131|IL3RB|IL5RB|SMDP5|betaGMR		22	Cytokine_Receptors
CSF3R	1441	colony stimulating factor 3 receptor	CD114|GCSFR|SCN7		1	Cytokine_Receptors
CX3CR1	1524	C-X3-C motif chemokine receptor 1	CCRL1|CMKBRL1|CMKDR1|GPR13|GPRV28|V28		3	Cytokine_Receptors
CXCR3	2833	C-X-C motif chemokine receptor 3	CD182|CD183|CKR-L2|CMKAR3|GPR9|IP10-R|Mig-R|MigR	X		Cytokine_Receptors
CXCR4	7852	C-X-C motif chemokine receptor 4	CD184|D2S201E|FB22|HM89|HSY3RR|LAP-3|LAP3|LCR1|LESTR|NPY3R|NPYR|NPYRL|NPYY3R|WHIM|WHIMS		2	Cytokine_Receptors

CXCR5	643	C-X-C motif chemokine receptor 5	BLR1|CD185|MDR15		11	Cytokine_Receptors
CXCR6	10663	C-X-C motif chemokine receptor 6	BONZO|CD186|STRL33|TYMSTR		3	Cytokine_Receptors
ACKR3	57007	atypical chemokine receptor 3	CMKOR1|CXC-R7|CXCR-7|CXCR7|GPR159|RDC-1|RDC1		2	Cytokine_Receptors
CYSLTR1	10800	cysteinyl leukotriene receptor 1	CYSLT1|CYSLT1R|CYSLTR|HMTMF81	X		Cytokine_Receptors
CYSLTR2	57105	cysteinyl leukotriene receptor 2	CYSLT2|CYSLT2R|GPCR21|HG57|HPN321|KPG_011|PSEC0146|hGPCR21		13	Cytokine_Receptors
ACKR1	2532	atypical chemokine receptor 1 (Duffy blood group)	CCBP1|CD234|DARC|DARC/ACKR1|Dfy|FY|GPD|GpFy|WBCQ1		1	Cytokine_Receptors
EDNRA	1909	endothelin receptor type A	ET-A|ETA|ETA-R|ETAR|ETRA|MFDA|hET-AR		4	Cytokine_Receptors
EDNRB	1910	endothelin receptor type B	ABCDS|ET-B|ET-BR|ETB|ETB1|ETBR|ETRB|HSCR|HSCR2|WS4A		13	Cytokine_Receptors
EGFR	1956	epidermal growth factor receptor	ERBB|ERBB1|HER1|NISBD2|PIG61|mENA		7	Cytokine_Receptors
ENG	2022	endoglin	END|HHT1|ORW1		9	Cytokine_Receptors
EPOR	2057	erythropoietin receptor	EPO-R		19	Cytokine_Receptors
ESR1	2099	estrogen receptor 1	ER|ESR|ESRA|ESTRR|Era|NR3A1		6	Cytokine_Receptors
ESR2	2100	estrogen receptor 2	ER-BETA|ESR-BETA|ESRB|ESTRB|Erb|NR3A2|ODG8		14	Cytokine_Receptors
ESRRA	2101	estrogen related receptor alpha	ERR1|ERRa|ERRalpha|ESRL1|NR3B1		11	Cytokine_Receptors
ESRRB	2103	estrogen related receptor beta	DFNB35|ERR beta-2|ERR2|ERRb|ERRbeta2|ESRL2|NR3B2		14	Cytokine_Receptors
ESRRG	2104	estrogen related receptor gamma	ERR-gamma|ERR3|ERRg|ERRgamma|NR3B3		1	Cytokine_Receptors
FGFR1	2260	fibroblast growth factor receptor 1	BFGFR|CD331|CEK|ECCL|FGFBR|FGFR-1|FL		8	Cytokine_Receptors

			G|FLT-2|FLT2|HBGFR|HH2|HRTFDS|KAL2|N-SAM|OGD|bFGF-R-1		
FGFR2	2263	fibroblast growth factor receptor 2	BBDS|BEK|BFR-1|CD332|CEK3|CFD1|ECT1|JWS|K-SAM|KGFR|TK14|TK25	10	Cytokine_Receptors
FGFR3	2261	fibroblast growth factor receptor 3	ACH|CD333|CEK2|HSFGFR3EX|JTK4	4	Cytokine_Receptors
FGFR4	2264	fibroblast growth factor receptor 4	CD334|JTK2|TKF	5	Cytokine_Receptors
FGFRL1	53834	fibroblast growth factor receptor like 1	FGFR-5|FGFR5|FHFR	4	Cytokine_Receptors
FLT1	2321	fms related receptor tyrosine kinase 1	FLT|FLT-1|VEGFR-1|VEGFR1	13	Cytokine_Receptors
FLT3	2322	fms related receptor tyrosine kinase 3	CD135|FLK-2|FLK2|STK1	13	Cytokine_Receptors
FLT4	2324	fms related receptor tyrosine kinase 4	CHTD7|FLT-4|FLT41|LMPH1A|LMPHM1|PCL|VEGFR-3|VEGFR3	5	Cytokine_Receptors
FPR1	2357	formyl peptide receptor 1	FMLP|FPR	19	Cytokine_Receptors
FPR2	2358	formyl peptide receptor 2	ALXR|FMLP-R-II|FMLPX|FPR2A|FPRH1|FPRH2|FPRL1|HM63|LXA4R	19	Cytokine_Receptors
FPR2	2358	formyl peptide receptor 2	ALXR|FMLP-R-II|FMLPX|FPR2A|FPRH1|FPRH2|FPRL1|HM63|LXA4R	19	Cytokine_Receptors
FSHR	2492	follicle stimulating hormone receptor	FSHR1|FSHRO|LGR1|ODG1	2	Cytokine_Receptors
GALR2	8811	galanin receptor 2	GAL2-R|GALNR2|GALR-2	17	Cytokine_Receptors
GALR3	8484	galanin receptor 3	-	22	Cytokine_Receptors
GCGR	2642	glucagon receptor	GGR|GL-R	17	Cytokine_Receptors
GHR	2690	growth hormone receptor	GHBP|GHIP	5	Cytokine_Receptors
GHRHR	2692	growth hormone releasing hormone receptor	GHRFR|GRFR|IGHD1B|IGHD4	7	Cytokine_Receptors
GHSR	2693	growth hormone secretagogue receptor	GHDP	3	Cytokine_Receptors
GIPR	2696	gastric inhibitory polypeptide receptor	PGQTL2	19	Cytokine_Receptors
GLP1R	2740	glucagon like peptide 1 receptor	GLP-1|GLP-1-R|GLP-1R	6	Cytokine_Receptors

GLP2R	9340	glucagon like peptide 2 receptor	-	17	Cytokine_Receptors
GNRHR	2798	gonadotropin releasing hormone receptor	GNRHR1|GRHR|HH7|LHRHR|LRHR	4	Cytokine_Receptors
GPER1	2852	G protein-coupled estrogen receptor 1	CEPR|CMKRL2|DRY12|FEG-1|GPCR-Br|GPER|GPR30|LERGU|LERGU2|LyGPR|mER	7	Cytokine_Receptors
GPR17	2840	G protein-coupled receptor 17	-	2	Cytokine_Receptors
GPR32	2854	G protein-coupled receptor 32	RVDR1	19	Cytokine_Receptors
GPR33	2856	G protein-coupled receptor 33	-	14	Cytokine_Receptors
PTGDR2	11251	prostaglandin D2 receptor 2	CD294|CRTH2|DL1R|DP2|GPR44	11	Cytokine_Receptors
C5AR2	27202	complement component 5a receptor 2	C5L2|GPF77|GPR77	19	Cytokine_Receptors
HNF4A	3172	hepatocyte nuclear factor 4 alpha	FRTS4|HNF4|HNF4a7|HNF4a8|HNF4a9|HNF4alpha|MODY|MODY1|NR2A1|NR2A21|TCF|TCF14	20	Cytokine_Receptors
HNF4G	3174	hepatocyte nuclear factor 4 gamma	NR2A2|NR2A3	8	Cytokine_Receptors
HTR3A	3359	5-hydroxytryptamine receptor 3A	5-HT-3|5-HT3A|5-HT3R|5HT3R|HTR3	11	Cytokine_Receptors
HTR3B	9177	5-hydroxytryptamine receptor 3B	5-HT3B	11	Cytokine_Receptors
HTR3C	170572	5-hydroxytryptamine receptor 3C	-	3	Cytokine_Receptors
HTR3D	200909	5-hydroxytryptamine receptor 3D	5HT3D	3	Cytokine_Receptors
HTR3E	285242	5-hydroxytryptamine receptor 3E	5-HT3-E|5-HT3E|5-HT3c1	3	Cytokine_Receptors
IFNAR1	3454	interferon alpha and beta receptor subunit 1	AVP|IFN-alpha-REC|IFNAR|IFNBR|IFRC	21	Cytokine_Receptors
IFNAR	3455	interferon alpha and beta receptor subunit 2	IFN-R|IFN-alpha-REC|IFNABR|IFNARB|IM	21	Cytokine_Receptors

2			D45			
IFNGR1	3459	interferon gamma receptor 1	CD119|IFNGR|IMD27A|IMD27B		6	Cytokine_Receptors
IFNGR2	3460	interferon gamma receptor 2	AF-1|IFGR2|IFNGT1|IMD28		21	Cytokine_Receptors
IGF1R	3480	insulin like growth factor 1 receptor	CD221|IGFIR|IGFR|JTK13		15	Cytokine_Receptors
IGF2R	3482	insulin like growth factor 2 receptor	CD222|CI-M6PR|CIMPR|M6P-R|M6P/IGF2R|MPR 300|MPR1|MPR300|MPRI		6	Cytokine_Receptors
IL10RA	3587	interleukin 10 receptor subunit alpha	CD210|CD210a|CDW210A|HIL-10R|IL-10R1|IL10R		11	Cytokine_Receptors
IL10RB	3588	interleukin 10 receptor subunit beta	CDW210B|CRF2-4|CRFB4|D21S58|D21S66|IL-10R2		21	Cytokine_Receptors
IL11RA	3590	interleukin 11 receptor subunit alpha	CRSDA		9	Cytokine_Receptors
IL12RB1	3594	interleukin 12 receptor subunit beta 1	CD212|IL-12R-BETA1|IL12RB|IMD30		19	Cytokine_Receptors
IL12RB2	3595	interleukin 12 receptor subunit beta 2	-		1	Cytokine_Receptors
IL13RA1	3597	interleukin 13 receptor subunit alpha 1	CD213A1|CT19|IL-13Ra|NR4	X		Cytokine_Receptors
IL13RA2	3598	interleukin 13 receptor subunit alpha 2	CD213A2|CT19|IL-13R|IL13BP	X		Cytokine_Receptors
IL15RA	3601	interleukin 15 receptor subunit alpha	CD215		10	Cytokine_Receptors
IL2RB	3560	interleukin 2 receptor subunit beta	CD122|IL15RB|IMD63|P70-75		22	Cytokine_Receptors
IL17R	2376	interleukin 17 receptor A	CANDF5|CD217|CDw217|IL-17RA|IL17R|IM		22	Cytokine_Receptors

A	5		D51|hIL-17R		
IL17RB	55540	interleukin 17 receptor B	CRL4|EVI27|IL17BR|IL17RH1	3	Cytokine_Receptors
IL17RC	84818	interleukin 17 receptor C	CANDF9|IL17-RL|IL17RL	3	Cytokine_Receptors
IL17RD	54756	interleukin 17 receptor D	HH18|IL-17RD|IL17RLM|SEF	3	Cytokine_Receptors
IL17RE	132014	interleukin 17 receptor E	-	3	Cytokine_Receptors
IL18R1	8809	interleukin 18 receptor 1	CD218a|CDw218a|IL-18R-alpha|IL-18Ralpha|IL-1Rrp|IL18RA|IL18Ralpha2|IL1RRP	2	Cytokine_Receptors
IL18RAP	8807	interleukin 18 receptor accessory protein	ACPL|CD218b|CDw218b|IL-18R-beta|IL-18RAcP|IL-18Rbeta|IL-1R-7|IL-1R7|IL-1RAcPL|IL18RB	2	Cytokine_Receptors
IL1R1	3554	interleukin 1 receptor type 1	CD121A|D2S1473|IL-1R-alpha|IL1R|IL1RA|P80	2	Cytokine_Receptors
IL1R2	7850	interleukin 1 receptor type 2	CD121b|CDw121b|IL-1R-2|IL-1RT-2|IL-1RT2|IL1R2c|IL1RB	2	Cytokine_Receptors
IL1RAP	3556	interleukin 1 receptor accessory protein	C3orf13|IL-1RAcP|IL1R3	3	Cytokine_Receptors
IL1RL1	9173	interleukin 1 receptor like 1	DER4|FIT-1|IL33R|ST2|ST2L|ST2V|T1	2	Cytokine_Receptors
IL1RL2	8808	interleukin 1 receptor like 2	IL-1Rrp2|IL-36R|IL1R-rp2|IL1RRP2	2	Cytokine_Receptors
IL20R	5383	interleukin 20 receptor subunit alpha	CRF2-8|IL-20R-alpha|IL-20R1|IL-20RA	6	Cytokine_Receptors

A	2				
IL20RB	53833	interleukin 20 receptor subunit beta	DIRS1|FNDC6|IL-20R2	3	Cytokine_Receptors
IL21R	50615	interleukin 21 receptor	CD360|IMD56|NILR	16	Cytokine_Receptors
IL22RA1	58985	interleukin 22 receptor subunit alpha 1	CRF2-9|IL22R|IL22R1	1	Cytokine_Receptors
IL22RA2	116379	interleukin 22 receptor subunit alpha 2	CRF2-10|CRF2-S1|CRF2X|IL-22BP|IL-22R-alpha-2|IL-22RA2|ZCYTOR16	6	Cytokine_Receptors
IL23R	149233	interleukin 23 receptor	-	1	Cytokine_Receptors
IL27RA	9466	interleukin 27 receptor subunit alpha	CRL1|IL-27RA|IL27R|TCCR|WSX1|zcytor1	19	Cytokine_Receptors
IFNLR1	163702	interferon lambda receptor 1	CRF2/12|IFNLR|IL-28R1|IL28RA|LICR2	1	Cytokine_Receptors
IL2RA	3559	interleukin 2 receptor subunit alpha	CD25|IDDM10|IL2R|IMD41|TCGFR|p55	10	Cytokine_Receptors
IL2RB	3560	interleukin 2 receptor subunit beta	CD122|IL15RB|IMD63|P70-75	22	Cytokine_Receptors
IL2RG	3561	interleukin 2 receptor subunit gamma	CD132|CIDX|IL-2RG|IMD4|P64|SCIDX|SCIDX1	X	Cytokine_Receptors
IL31RA	133396	interleukin 31 receptor A	CRL|CRL3|GLM-R|GLMR|GPL|IL-31RA|PLCA2|PRO21384|hGLM-R	5	Cytokine_Receptors
IL3RA	3563	interleukin 3 receptor subunit alpha	CD123|IL3R|IL3RAY|IL3RX|IL3RY|hIL-3Ra	X|Y	Cytokine_Receptors
IL4R	3566	interleukin 4 receptor	CD124|IL-4RA|IL4RA	16	Cytokine_Receptors
IL5RA	3568	interleukin 5 receptor subunit alpha	CD125|CDw125|HSIL5R3|IL5R	3	Cytokine_Receptors
IL6R	3570	interleukin 6 receptor	CD126|IL-6R-1|IL-6RA|IL6Q|IL6RA|IL6R	1	Cytokine_Receptors

			Q|gp80			
IL7R	3575	interleukin 7 receptor	CD127|CDW127|IL-7R-alpha|IL7RA|ILRA		5	Cytokine_Receptors
CXCR1	3577	C-X-C motif chemokine receptor 1	C-C|C-C-CKR-1|CD128|CD181|CDw128a|CKR-1|CMKAR1|IL8R1|IL8RA|IL8RBA		2	Cytokine_Receptors
CXCR2	3579	C-X-C motif chemokine receptor 2	CD182|CDw128b|CMKAR2|IL8R2|IL8RA|IL8RB		2	Cytokine_Receptors
IL9R	3581	interleukin 9 receptor	CD129|IL-9R	X|Y		Cytokine_Receptors
INSR	3643	insulin receptor	CD220|HHF5		19	Cytokine_Receptors
KDR	3791	kinase insert domain receptor	CD309|FLK1|VEGFR|VEGFR2		4	Cytokine_Receptors
LEPR	3953	leptin receptor	CD295|LEP-R|LEPRD|OB-R|OBR		1	Cytokine_Receptors
LGR4	55366	leucine rich repeat containing G protein-coupled receptor 4	BNMD17|GPR48		11	Cytokine_Receptors
LGR5	8549	leucine rich repeat containing G protein-coupled receptor 5	FEX|GPR49|GPR67|GRP49|HG38		12	Cytokine_Receptors
LGR6	59352	leucine rich repeat containing G protein-coupled receptor 6	GPCR|VTS20631		1	Cytokine_Receptors
LHCGR	3973	luteinizing hormone/choriogonadotropin receptor	HHG|LCGR|LGR2|LH/CG-R|LH/CGR|LHR|LHRHR|LSH-R|ULG5		2	Cytokine_Receptors
LIFR	3977	LIF receptor subunit alpha	CD118|LIF-R|SJS2|STWS|SWS		5	Cytokine_Receptors
LTB4R	1241	leukotriene B4 receptor	BLT1|BLTR|CMKRL1|GPR16|LTB4R1|LTBR1|P2RY7|P2Y7		14	Cytokine_Receptors
LTB4R2	56413	leukotriene B4 receptor 2	BLT2|BLTR2|JULF2|KPG_004|LTB4-R 2|LTB4-R2|NOP9		14	Cytokine_Receptors
LTBR	4055	lymphotoxin beta receptor	D12S370|LT-BETA-R|TNF-R-III|TNFCR|TNFR-RP|TNFR2-RP|TNFR3|TNFRSF3		12	Cytokine_Receptors
MC1R	4157	melanocortin 1 receptor	CMM5|MSH-R|SHEP2		16	Cytokine_Receptors

MC2R	4158	melanocortin 2 receptor	ACTHR		18	Cytokine_Receptors
MC3R	4159	melanocortin 3 receptor	BMIQ9|MC3|MC3-R|OB20|OQTL		20	Cytokine_Receptors
MC4R	4160	melanocortin 4 receptor	BMIQ20		18	Cytokine_Receptors
MCHR1	2847	melanin concentrating hormone receptor 1	GPR24|MCH-1R|MCH1R|SLC-1|SLC1		22	Cytokine_Receptors
MCHR2	84539	melanin concentrating hormone receptor 2	GPR145|GPRv17|MCH-2R|MCH-R2|MCH2|MCH2R|MCHR-2|SLT		6	Cytokine_Receptors
MET	4233	MET proto-oncogene, receptor tyrosine kinase	AUTS9|DFNB97|HGFR|RCCP2|c-Met		7	Cytokine_Receptors
MLNR	2862	motilin receptor	GPR38|MTLR1		13	Cytokine_Receptors
MPL	4352	MPL proto-oncogene, thrombopoietin receptor	C-MPL|CD110|MPLV|THCYT2|THPOR|TPOR		1	Cytokine_Receptors
MTNR1A	4543	melatonin receptor 1A	MEL-1A-R|MT1		4	Cytokine_Receptors
MTNR1B	4544	melatonin receptor 1B	FGQTL2|MEL-1B-R|MT2		11	Cytokine_Receptors
NGFR	4804	nerve growth factor receptor	CD271|Gp80-LNGFR|TNFRSF16|p75(NTR)|p75NTR		17	Cytokine_Receptors
NMBR	4829	neuromedin B receptor	BB1|BB1R|NMB-R		6	Cytokine_Receptors
NPR1	4881	natriuretic peptide receptor 1	ANPRA|ANPa|GUC2A|GUCY2A|NPRA		1	Cytokine_Receptors
NPR3	4883	natriuretic peptide receptor 3	ANP-C|ANPR-C|ANPRC|C5orf23|GUCY2B|NPR-C|NPRC		5	Cytokine_Receptors
NR0B1	190	nuclear receptor subfamily 0 group B member 1	AHC|AHCH|AHX|DAX-1|DAX1|DSS|GTD|HHG|NROB1|SRXY2	X		Cytokine_Receptors
NR0B2	8431	nuclear receptor subfamily 0 group B member 2	SHP|SHP1		1	Cytokine_Receptors
NR1D1	9572	nuclear receptor subfamily 1 group D member 1	EAR1|REVERBA|REVERBalpha|THRA1|THRAL|ear-1|hRev		17	Cytokine_Receptors
NR1D2	9975	nuclear receptor subfamily 1 group D member 2	BD73|EAR-1R|REVERBB|REVERBbeta|RVR		3	Cytokine_Receptors
NR1H2	7376	nuclear receptor subfamily 1 group H member 2	LXR-b|LXRB|NER|NER-I|RIP15|UNR		19	Cytokine_Receptors

NR1H3	1006<br>2	nuclear receptor subfamily 1 group H member 3	LXR-a|LXRA|RLD-1	11	Cytokine_Receptors
NR1H4	9971	nuclear receptor subfamily 1 group H member 4	BAR|FXR|HRR-1|HRR1|PFIC5|RIP14	12	Cytokine_Receptors
NR1I2	8856	nuclear receptor subfamily 1 group I member 2	BXR|ONR1|PAR|PAR1|PAR2|PARq|PRR|PXR| SAR|SXR	3	Cytokine_Receptors
NR1I3	9970	nuclear receptor subfamily 1 group I member 3	CAR|CAR1|MB67	1	Cytokine_Receptors
NR2C1	7181	nuclear receptor subfamily 2 group C member 1	TR2	12	Cytokine_Receptors
NR2C2	7182	nuclear receptor subfamily 2 group C member 2	TAK1|TR4	3	Cytokine_Receptors
NR2E1	7101	nuclear receptor subfamily 2 group E member 1	TLL|TLX|XTLL	6	Cytokine_Receptors
NR2E3	1000<br>2	nuclear receptor subfamily 2 group E member 3	ESCS|PNR|RNR|RP37|rd7	15	Cytokine_Receptors
NR2F1	7025	nuclear receptor subfamily 2 group F member 1	BBOAS|BBSOAS|COUP-TFI|COUPTF1|EAR-3| EAR3|ERBAL3|SVP44|TCFCOUP1|TFCOUP1	5	Cytokine_Receptors
NR2F2	7026	nuclear receptor subfamily 2 group F member 2	ARP-1|ARP1|CHTD4|COUPTF2|COUPTFB|COU<br>PTFII|NF-E3|SVP40|TFCOUP2	15	Cytokine_Receptors
NR2F6	2063	nuclear receptor subfamily 2 group F member 6	EAR-2|EAR2|ERBAL2	19	Cytokine_Receptors
NR3C1	2908	nuclear receptor subfamily 3 group C member 1	GCCR|GCR|GCRST|GR|GRL	5	Cytokine_Receptors
NR3C2	4306	nuclear receptor subfamily 3 group C member 2	MCR|MLR|MR|NR3C2VIT	4	Cytokine_Receptors
NR4A1	3164	nuclear receptor subfamily 4 group A member 1	GFRP1|HMR|N10|NAK-1|NGFIB|NP10|NUR77<br>|TR3	12	Cytokine_Receptors
NR4A2	4929	nuclear receptor subfamily 4 group A member 2	HZF-3|NOT|NURR1|RNR1|TINUR	2	Cytokine_Receptors
NR4A3	8013	nuclear receptor subfamily 4 group A member 3	CHN|CSMF|MINOR|NOR1|TEC	9	Cytokine_Receptors
NR5A1	2516	nuclear receptor subfamily 5 group A member 1	AD4BP|ELP|FTZ1|FTZF1|POF7|SF-1|SF1|S<br>PGF8|SRXX4|SRXY3|hSF-1	9	Cytokine_Receptors
NR5A2	2494	nuclear receptor subfamily 5 group A member 2	B1F|B1F2|CPF|FTF|FTZ-F1|FTZ-F1beta|L<br>RH-1|LRH1|hB1F-2	1	Cytokine_Receptors

NR6A1	2649	nuclear receptor subfamily 6 group A member 1	CT150|GCNF|GCNF1|NR61|RTR|hGCNF|hRTR		9	Cytokine_Receptors
NRP1	8829	neuropilin 1	BDCA4|CD304|NP1|NRP|VEGF165R		10	Cytokine_Receptors
NRP2	8828	neuropilin 2	NP2|NPN2|PRO2714|VEGF165R2		2	Cytokine_Receptors
OGFR	11054	opioid growth factor receptor	-		20	Cytokine_Receptors
OPRD1	4985	opioid receptor delta 1	DOP|DOR|DOR1|OPRD		1	Cytokine_Receptors
OPRK1	4986	opioid receptor kappa 1	K-OR-1|KOP|KOR|KOR-1|KOR1|OPRK		8	Cytokine_Receptors
OPRL1	4987	opioid related nociceptin receptor 1	KOR-3|KOR3|NOCIR|NOP|NOPr|OOR|OPRL|ORL1		20	Cytokine_Receptors
OPRM1	4988	opioid receptor mu 1	LMOR|M-OR-1|MOP|MOR|MOR1|OPRM		6	Cytokine_Receptors
OSMR	9180	oncostatin M receptor	IL-31R-beta|IL-31RB|OSMRB|OSMRbeta|PLCA1		5	Cytokine_Receptors
OXTR	5021	oxytocin receptor	OT-R		3	Cytokine_Receptors
PGR	5241	progesterone receptor	NR3C3|PR		11	Cytokine_Receptors
PGRMC2	10424	progesterone receptor membrane component 2	DG6|PMBP		4	Cytokine_Receptors
PLAUR	5329	plasminogen activator, urokinase receptor	CD87|U-PAR|UPAR|URKR		19	Cytokine_Receptors
PLXNA1	5361	plexin A1	NOV|NOVP|PLEXIN-A1|PLXN1		3	Cytokine_Receptors
PLXNA2	5362	plexin A2	OCT|PLXN2		1	Cytokine_Receptors
PLXNA3	55558	plexin A3	6.3|HSSEXGENE|PLXN3|PLXN4|XAP-6	X		Cytokine_Receptors
PLXNA4	91584	plexin A4	FAYV2820|PLEXA4|PLXNA4A|PLXNA4B|PRO34003		7	Cytokine_Receptors
PLXNB	5364	plexin B1	PLEXIN-B1|PLXN5|SEP		3	Cytokine_Receptors

1						
PLXNB2	23654	plexin B2	MM1|Nbla00445|PLEXB2|dJ402G11.3		22	Cytokine_Receptors
PLXNB3	5365	plexin B3	PLEXB3|PLEXR|PLXN6	X		Cytokine_Receptors
PLXNC1	10154	plexin C1	CD232|PLXN-C1|VESPR		12	Cytokine_Receptors
PLXND1	23129	plexin D1	PLEXD1		3	Cytokine_Receptors
PPARA	5465	peroxisome proliferator activated receptor alpha	NR1C1|PPAR|PPARalpha|hPPAR		22	Cytokine_Receptors
PPARD	5467	peroxisome proliferator activated receptor delta	FAAR|NR1C2|NUC1|NUCI|NUCII|PPARB		6	Cytokine_Receptors
PPARG	5468	peroxisome proliferator activated receptor gamma	CIMT1|GLM1|NR1C3|PPARG1|PPARG2|PPARG5|PPARgamma		3	Cytokine_Receptors
PRLHR	2834	prolactin releasing hormone receptor	GPR10|GR3|PrRPR		10	Cytokine_Receptors
PRLR	5618	prolactin receptor	HPRL|MFAB|RI-PRLR|hPRLrI		5	Cytokine_Receptors
PTAFR	5724	platelet activating factor receptor	PAFR		1	Cytokine_Receptors
PTGDR	5729	prostaglandin D2 receptor	AS1|ASRT1|DP|DP1|PTGDR1		14	Cytokine_Receptors
PTGDS	5730	prostaglandin D2 synthase	L-PGDS|LPGDS|PDS|PGD2|PGDS|PGDS2		9	Cytokine_Receptors
PTGER1	5731	prostaglandin E receptor 1	EP1		19	Cytokine_Receptors
PTGER2	5732	prostaglandin E receptor 2	EP2		14	Cytokine_Receptors
PTGER3	5733	prostaglandin E receptor 3	EP3|EP3-I|EP3-II|EP3-III|EP3-IV|EP3-VI|EP3e|PGE2-R|lnc003875		1	Cytokine_Receptors

PTGER4	5734	prostaglandin E receptor 4	EP4|EP4R	5	Cytokine_Receptors
PTGFR	5737	prostaglandin F receptor	FP	1	Cytokine_Receptors
PTH1R	5745	parathyroid hormone 1 receptor	EKNS|PFE|PTHR|PTHR1	3	Cytokine_Receptors
PTH2R	5746	parathyroid hormone 2 receptor	PTHR2	2	Cytokine_Receptors
RARA	5914	retinoic acid receptor alpha	NR1B1|RAR	17	Cytokine_Receptors
RARB	5915	retinoic acid receptor beta	HAP|MCOPS12|NR1B2|RARbeta1|RRB2	3	Cytokine_Receptors
RARG	5916	retinoic acid receptor gamma	NR1B3|RARC	12	Cytokine_Receptors
ROBO1	6091	roundabout guidance receptor 1	DUTT1|SAX3	3	Cytokine_Receptors
ROBO2	6092	roundabout guidance receptor 2	SAX3	3	Cytokine_Receptors
ROBO3	64221	roundabout guidance receptor 3	HGPPS|HGPPS1|HGPS|RBIG1|RIG1	11	Cytokine_Receptors
RORA	6095	RAR related orphan receptor A	IDDECA|NR1F1|ROR1|ROR2|ROR3|RZR-ALPHA|RZRA	15	Cytokine_Receptors
RORB	6096	RAR related orphan receptor B	EIG15|NR1F2|ROR-BETA|RZR-BETA|RZRB|bA133M9.1	9	Cytokine_Receptors
RORC	6097	RAR related orphan receptor C	IMD42|NR1F3|RORG|RZR-GAMMA|RZRG|TOR	1	Cytokine_Receptors
RXFP1	59350	relaxin family peptide receptor 1	LGR7|RXFPR1	4	Cytokine_Receptors
RXFP2	122042	relaxin family peptide receptor 2	GPR106|GREAT|INSL3R|LGR8|LGR8.1|RXFPR2	13	Cytokine_Receptors
RXFP3	51289	relaxin family peptide receptor 3	GPCR135|RLN3R1|RXFPR3|SALPR	5	Cytokine_Receptors
RXRA	6256	retinoid X receptor alpha	NR2B1	9	Cytokine_Receptors
RXRB	6257	retinoid X receptor beta	DAUDI6|H-2RIIBP|NR2B2|RCoR-1	6	Cytokine_Receptors
RXRG	6258	retinoid X receptor gamma	NR2B3|RXRC	1	Cytokine_Receptors

S1PR1	1901	sphingosine-1-phosphate receptor 1	CD363|CHEDG1|D1S3362|ECGF1|EDG-1|EDG1|S1P1	1	Cytokine_Receptors
S1PR2	9294	sphingosine-1-phosphate receptor 2	AGR16|DFNB68|EDG-5|EDG5|Gpcr13|H218|LPB2|S1P2	19	Cytokine_Receptors
SCTR	6344	secretin receptor	SR	2	Cytokine_Receptors
SDC1	6382	syndecan 1	CD138|SDC|SYND1|syndecan	2	Cytokine_Receptors
SDC2	6383	syndecan 2	CD362|HSPG|HSPG1|SYND2	8	Cytokine_Receptors
SDC3	9672	syndecan 3	SDCN|SYND3	1	Cytokine_Receptors
SDC4	6385	syndecan 4	SYND4	20	Cytokine_Receptors
SORT1	6272	sortilin 1	Gp95|LDLCQ6|NT3|NTR3	1	Cytokine_Receptors
SSTR1	6751	somatostatin receptor 1	SRIF-2|SS-1-R|SS1-R|SS1R	14	Cytokine_Receptors
SSTR2	6752	somatostatin receptor 2	-	17	Cytokine_Receptors
SSTR5	6755	somatostatin receptor 5	SS-5-R	16	Cytokine_Receptors
ST2	6761	-	-	11	Cytokine_Receptors
TACR1	6869	tachykinin receptor 1	NK1R|NKIR|SPR|TAC1R	2	Cytokine_Receptors
TEK	7010	TEK receptor tyrosine kinase	CD202B|GLC3E|TIE-2|TIE2|VMCM|VMCM1	9	Cytokine_Receptors
TGFBR1	7046	transforming growth factor beta receptor 1	AAT5|ACVRLK4|ALK-5|ALK5|ESS1|LDS1|LDS1A|LDS2A|MSSE|SKR4|TBR-i|TBRI|TGFR-1|tbetaR-I	9	Cytokine_Receptors
TGFBR2	7048	transforming growth factor beta receptor 2	AAT3|FAA3|LDS1B|LDS2|LDS2B|MFS2|RIIC|TAAD2|TBR-ii|TBRII|TGFR-2|TGFbeta-RII	3	Cytokine_Receptors
TGFBR3	7049	transforming growth factor beta receptor 3	BGCAN|betaglycan	1	Cytokine_Receptors
THRA	7067	thyroid hormone receptor alpha	AR7|CHNG6|EAR7|ERB-T-1|ERBA|ERBA1|NR1A1|THRA1|THRA2|c-ERBA-1	17	Cytokine_Receptors

THRB	7068	thyroid hormone receptor beta	C-ERBA-2|C-ERBA-BETA|ERBA2|GRTH|NR1A2|PRTH|THR1|THRB1|THRB2	3	Cytokine_Receptors
TIE1	7075	tyrosine kinase with immunoglobulin like and EGF like domains 1	JTK14|TIE	1	Cytokine_Receptors
TNFRSF10A	8797	TNF receptor superfamily member 10a	APO2|CD261|DR4|TRAILR-1|TRAILR1	8	Cytokine_Receptors
TNFRSF10B	8795	TNF receptor superfamily member 10b	CD262|DR5|KILLER|KILLER/DR5|TRAIL-R2|TRAILR2|TRICK2|TRICK2A|TRICK2B|TRICKB|ZTNFR9	8	Cytokine_Receptors
TNFRSF10C	8794	TNF receptor superfamily member 10c	CD263|DCR1|DCR1-TNFR|LIT|TRAIL-R3|TRAILR3|TRID	8	Cytokine_Receptors
TNFRSF10D	8793	TNF receptor superfamily member 10d	CD264|DCR2|TRAIL-R4|TRAILR4|TRUNDD	8	Cytokine_Receptors
TNFRSF11A	8792	TNF receptor superfamily member 11a	CD265|FEO|LOH18CR1|ODFR|OFE|OPTB7|OSTS|PDB2|RANK|TRANCER	18	Cytokine_Receptors
TNFRSF12A	51330	TNF receptor superfamily member 12A	CD266|FN14|TWEAKR	16	Cytokine_Receptors
TNFRSF13B	23495	TNF receptor superfamily member 13B	CD267|CVID|CVID2|IGAD2|RYZN|TACI|TNFRSF14B	17	Cytokine_Receptors
TNFRSF13C	115650	TNF receptor superfamily member 13C	BAFF-R|BAFFR|BROMIX|CD268|CVID4|prolixin	22	Cytokine_Receptors
TNFRSF14	8764	TNF receptor superfamily member 14	ATAR|CD270|HVEA|HVEM|LIGHTR|TR2	1	Cytokine_Receptors
TNFRSF17	608	TNF receptor superfamily member 17	BCM|BCMA|CD269|TNFRSF13A	16	Cytokine_Receptors
TNFRS	8784	TNF receptor superfamily member 18	AITR|CD357|ENERGEN|GITR|GITR-D	1	Cytokine_Receptors

F18					
TNFRSF19	55504	TNF receptor superfamily member 19	TAJ|TAJ-alpha|TRADE|TROY	13	Cytokine_Receptors
TNFRSF1A	7132	TNF receptor superfamily member 1A	CD120a|FPF|TBP1|TNF-R|TNF-R-I|TNF-R55|TNFAR|TNFR1|TNFR55|TNFR60|p55|p55-R|p60	12	Cytokine_Receptors
TNFRSF1B	7133	TNF receptor superfamily member 1B	CD120b|TBPII|TNF-R-II|TNF-R75|TNFBR|TNFR1B|TNFR2|TNFR80|p75|p75TNFR	1	Cytokine_Receptors
TNFRSF21	27242	TNF receptor superfamily member 21	BM-018|CD358|DR6	6	Cytokine_Receptors
TNFRSF25	8718	TNF receptor superfamily member 25	APO-3|DDR3|DR3|GEF720|LARD|PLEKHG5|TNFRSF12|TR3|TRAMP|WSL-1|WSL-LR	1	Cytokine_Receptors
TNFRSF4	7293	TNF receptor superfamily member 4	ACT35|CD134|IMD16|OX40|TXGP1L	1	Cytokine_Receptors
TNFRSF6B	8771	TNF receptor superfamily member 6b	DCR3|DJ583P15.1.1|M68|M68E|TR6	20	Cytokine_Receptors
TNFRSF8	943	TNF receptor superfamily member 8	CD30|D1S166E|Ki-1	1	Cytokine_Receptors
TNFRSF9	3604	TNF receptor superfamily member 9	4-1BB|CD137|CDw137|ILA	1	Cytokine_Receptors
TRHR	7201	thyrotropin releasing hormone receptor	CHNG7|TRH-R	8	Cytokine_Receptors
TSHR	7253	thyroid stimulating hormone receptor	CHNG1|LGR3|hTSHR-I	14	Cytokine_Receptors
TUBB3	10381	tubulin beta 3 class III	CDCBM|CDCBM1|CFEOM3|CFEOM3A|FEOM3|TUBB4|beta-4	16	Cytokine_Receptors
VDR	7421	vitamin D receptor	NR1I1|PPP1R163	12	Cytokine_Receptors
VIPR1	7433	vasoactive intestinal peptide receptor 1	HVR1|II|PACAP-R-2|PACAP-R2|RDC1|V1RG	3	Cytokine_Receptors

			|VAPC1|VIP-R-1|VIPR|VIRG|VPAC1|VPAC1R|VPCAP1R		
VIPR2	7434	vasoactive intestinal peptide receptor 2	C16DUPq36.3|DUP7q36.3|PACAP-R-3|PACAP-R3|VIP-R-2|VPAC2|VPAC2R|VPCAP2R	7	Cytokine_Receptors
XCR1	2829	X-C motif chemokine receptor 1	CCXCR1|GPR5	3	Cytokine_Receptors
IFNA10	3446	interferon alpha 10	IFN-alphaC	9	Interferons
IFNA13	3447	interferon alpha 13	-	9	Interferons
IFNA14	3448	interferon alpha 14	IFN-alphaH|LEIF2H	9	Interferons
IFNA16	3449	interferon alpha 16	IFN-alpha-16|IFN-alphaO	9	Interferons
IFNA17	3451	interferon alpha 17	IFN-alphaI|IFNA|INFA|LEIF2C1	9	Interferons
IFNA2	3440	interferon alpha 2	IFN-alpha-2|IFN-alphaA|IFNA|IFNA2B|leIF A	9	Interferons
IFNA21	3452	interferon alpha 21	IFN-alphaI|LeIF F|leIF-F	9	Interferons
IFNA4	3441	interferon alpha 4	IFN-alpha4a|INFA4	9	Interferons
IFNA5	3442	interferon alpha 5	IFN-alpha-5|IFN-alphaG|INA5|INFA5|leIF G	9	Interferons
IFNA6	3443	interferon alpha 6	IFN-alphaK	9	Interferons
IFNA7	3444	interferon alpha 7	IFN-alphaJ|IFNA-J	9	Interferons
IFNA8	3445	interferon alpha 8	IFN-alphaB	9	Interferons
IFNB1	3456	interferon beta 1	IFB|IFF|IFN-beta|IFNB	9	Interferons

IFNE	338376	interferon epsilon	IFN-E|IFNE1|IFNT1|INFE1|PRO655	9	Interferons
IFNG	3458	interferon gamma	IFG|IFI	12	Interferons
IFNK	56832	interferon kappa	IFNT1|INFE1	9	Interferons
IFNW1	3467	interferon omega 1	-	9	Interferons
IFNAR2	3455	interferon alpha and beta receptor subunit 2	IFN-R|IFN-alpha-REC|IFNABR|IFNARB|IMD45	21	Interferon_Receptor
IFNGR1	3459	interferon gamma receptor 1	CD119|IFNGR|IMD27A|IMD27B	6	Interferon_Receptor
IFNGR2	3460	interferon gamma receptor 2	AF-1|IFGR2|IFNGT1|IMD28	21	Interferon_Receptor
IL11	3589	interleukin 11	AGIF|IL-11	19	Interleukins
IL12A	3592	interleukin 12A	CLMF|IL-12A|NFSK|NKSF1|P35	3	Interleukins
IL12B	3593	interleukin 12B	CLMF|CLMF2|IL-12B|IMD28|IMD29|NKSF|NKSF2	5	Interleukins
IL13	3596	interleukin 13	IL-13|P600	5	Interleukins
IL15	3600	interleukin 15	IL-15	4	Interleukins
IL16	3603	interleukin 16	LCF|NIL16|PRIL16|prIL-16	15	Interleukins
IL17A	3605	interleukin 17A	CTLA-8|CTLA8|IL-17|IL-17A|IL17	6	Interleukins
IL17B	27190	interleukin 17B	IL-17B|IL-20|NIRF|ZCYTO7	5	Interleukins
IL17C	27189	interleukin 17C	CX2|IL-17C	16	Interleukins
IL17D	53342	interleukin 17D	IL-17D	13	Interleukins

IL17F	112744	interleukin 17F	CANDF6|IL-17F|ML-1|ML1	6	Interleukins
IL18	3606	interleukin 18	IGIF|IL-18|IL-1g|IL1F4	11	Interleukins
IL19	29949	interleukin 19	IL-10C|MDA1|NG.1|ZMDA1	1	Interleukins
IL1A	3552	interleukin 1 alpha	IL-1 alpha|IL-1A|IL1|IL1-ALPHA|IL1F1	2	Interleukins
IL1B	3553	interleukin 1 beta	IL-1|IL1-BETA|IL1F2|IL1beta	2	Interleukins
IL1F10	84639	interleukin 1 family member 10	FIL1-theta|FKSG75|IL-1HY2|IL-38|IL1-theta|IL1HY2	2	Interleukins
IL36RN	26525	interleukin 36 receptor antagonist	FIL1|FIL1(DELTA)|FIL1D|IL-36Ra|IL1F5|IL1HY1|IL1L1|IL1RP3|IL36RA|PSORP|PSORS14	2	Interleukins
IL36A	27179	interleukin 36 alpha	FIL1|FIL1(EPSILON)|FIL1E|IL-1F6|IL1(EPSILON)|IL1F6	2	Interleukins
IL37	27178	interleukin 37	FIL1|FIL1(ZETA)|FIL1Z|IL-1F7|IL-1H|IL-1H4|IL-1RP1|IL-37|IL1F7|IL1H4|IL1RP1	2	Interleukins
IL36B	27177	interleukin 36 beta	FIL1|FIL1-(ETA)|FIL1H|FILI-(ETA)|IL-1F8|IL-1H2|IL1-ETA|IL1F8|IL1H2	2	Interleukins
IL36G	56300	interleukin 36 gamma	IL-1F9|IL-1H1|IL-1RP2|IL1E|IL1F9|IL1H1|IL1RP2	2	Interleukins
IL1RN	3557	interleukin 1 receptor antagonist	DIRA|ICIL-1RA|IL-1RN|IL-1ra|IL-1ra3|IL1F3|IL1RA|IRAP|MVCD4	2	Interleukins
IL2	3558	interleukin 2	IL-2|TCGF|lymphokine	4	Interleukins
IL20	50604	interleukin 20	IL-20|IL10D|ZCYTO10	1	Interleukins

IL21	59067	interleukin 21	CVID11|IL-21|Za11	4	Interleukins
IL22	50616	interleukin 22	IL-21|IL-22|IL-D110|IL-TIF|ILTIF|TIFIL-23|TIFa|zcyto18	12	Interleukins
IL23A	51561	interleukin 23 subunit alpha	IL-23|IL-23A|IL23P19|P19|SGRF	12	Interleukins
IL24	11009	interleukin 24	C49A|FISP|IL10B|MDA7|MOB5|ST16	1	Interleukins
IL25	64806	interleukin 25	IL17E	14	Interleukins
IL26	55801	interleukin 26	AK155|IL-26	12	Interleukins
IL27	246778	interleukin 27	IL-27|IL-27A|IL27A|IL27p28|IL30|p28	16	Interleukins
IFNL2	282616	interferon lambda 2	IL-28A|IL28A	19	Interleukins
IFNL3	282617	interferon lambda 3	IFN-lambda-3|IFN-lambda-4|IL-28B|IL-28C|IL28B|IL28C	19	Interleukins
IFNL1	282618	interferon lambda 1	IL-29|IL29	19	Interleukins
IL3	3562	interleukin 3	IL-3|MCGF|MULTI-CSF	5	Interleukins
IL31	386653	interleukin 31	IL-31	12	Interleukins
IL32	9235	interleukin 32	IL-32alpha|IL-32beta|IL-32delta|IL-32gamma|NK4|TAIF|TAIFa|TAIFb|TAIFc|TAIFd	16	Interleukins

IL33	90865	interleukin 33	C9orf26|DVS27|IL1F11|NF-HEV|NFEHEV	9	Interleukins
IL34	146433	interleukin 34	C16orf77|IL-34	16	Interleukins
IL4	3565	interleukin 4	BCGF-1|BCGF1|BSF-1|BSF1|IL-4	5	Interleukins
IL5	3567	interleukin 5	EDF|IL-5|TRF	5	Interleukins
IL6	3569	interleukin 6	BSF-2|BSF2|CDF|HGF|HSF|IFN-beta-2|IFNB2|IL-6	7	Interleukins
IL6ST	3572	interleukin 6 signal transducer	CD130|CDW130|GP130|HIES4|IL-6RB|sGP130	5	Interleukins
IL7	3574	interleukin 7	IL-7	8	Interleukins
CXCL8	3576	C-X-C motif chemokine ligand 8	GCP-1|GCP1|IL8|LECT|LUCT|LYNAP|MDNCF|MONAP|NAF|NAP-1|NAP1|SCYB8	4	Interleukins
IL9	3578	interleukin 9	HP40|IL-9|P40	5	Interleukins
TXLNA	200081	taxilin alpha	IL14|TXLN	1	Interleukins
IL10RA	3587	interleukin 10 receptor subunit alpha	CD210|CD210a|CDW210A|HIL-10R|IL-10R1|IL10R	11	Interleukins_Receptor
IL10RB	3588	interleukin 10 receptor subunit beta	CDW210B|CRF2-4|CRFB4|D21S58|D21S66|IL-10R2	21	Interleukins_Receptor
IL11RA	3590	interleukin 11 receptor subunit alpha	CRSDA	9	Interleukins_Receptor
IL12RB1	3594	interleukin 12 receptor subunit beta 1	CD212|IL-12R-BETA1|IL12RB|IMD30	19	Interleukins_Receptor
IL12RB2	3595	interleukin 12 receptor subunit beta 2	-	1	Interleukins_Receptor

IL13RA1	3597	interleukin 13 receptor subunit alpha 1	CD213A1|CT19|IL-13Ra|NR4	X	Interleukins_Receptor
IL13RA2	3598	interleukin 13 receptor subunit alpha 2	CD213A2|CT19|IL-13R|IL13BP	X	Interleukins_Receptor
IL15RA	3601	interleukin 15 receptor subunit alpha	CD215	10	Interleukins_Receptor
IL2RB	3560	interleukin 2 receptor subunit beta	CD122|IL15RB|IMD63|P70-75	22	Interleukins_Receptor
IL17RA	23765	interleukin 17 receptor A	CANDF5|CD217|CDw217|IL-17RA|IL17R|IMD51|hIL-17R	22	Interleukins_Receptor
IL17RB	55540	interleukin 17 receptor B	CRL4|EVI27|IL17BR|IL17RH1	3	Interleukins_Receptor
IL17RC	84818	interleukin 17 receptor C	CANDF9|IL17-RL|IL17RL	3	Interleukins_Receptor
IL17RD	54756	interleukin 17 receptor D	HH18|IL-17RD|IL17RLM|SEF	3	Interleukins_Receptor
IL17RE	132014	interleukin 17 receptor E	-	3	Interleukins_Receptor
IL18R1	8809	interleukin 18 receptor 1	CD218a|CDw218a|IL-18R-alpha|IL-18Ralpha|IL-1Rrp|IL18RA|IL18Ralpha2|IL1RRP	2	Interleukins_Receptor
IL18RAP	8807	interleukin 18 receptor accessory protein	ACPL|CD218b|CDw218b|IL-18R-beta|IL-18RAcP|IL-18Rbeta|IL-1R-7|IL-1R7|IL-1RAcPL|IL18RB	2	Interleukins_Receptor
IL1R1	3554	interleukin 1 receptor type 1	CD121A|D2S1473|IL-1R-alpha|IL1R|IL1RA|P80	2	Interleukins_Receptor
IL1R2	7850	interleukin 1 receptor type 2	CD121b|CDw121b|IL-1R-2|IL-1RT-2|IL-1	2	Interleukins_Receptor

			RT2|IL1R2c|IL1RB		
IL1RAP	3556	interleukin 1 receptor accessory protein	C3orf13|IL-1RAcP|IL1R3	3	Interleukins_Receptor
IL1RL1	9173	interleukin 1 receptor like 1	DER4|FIT-1|IL33R|ST2|ST2L|ST2V|T1	2	Interleukins_Receptor
IL1RL2	8808	interleukin 1 receptor like 2	IL-1Rrp2|IL-36R|IL1R-rp2|IL1RRP2	2	Interleukins_Receptor
IL20RA	53832	interleukin 20 receptor subunit alpha	CRF2-8|IL-20R-alpha|IL-20R1|IL-20RA	6	Interleukins_Receptor
IL20RB	53833	interleukin 20 receptor subunit beta	DIRS1|FNDC6|IL-20R2	3	Interleukins_Receptor
IL21R	50615	interleukin 21 receptor	CD360|IMD56|NILR	16	Interleukins_Receptor
IL22RA1	58985	interleukin 22 receptor subunit alpha 1	CRF2-9|IL22R|IL22R1	1	Interleukins_Receptor
IL22RA2	116379	interleukin 22 receptor subunit alpha 2	CRF2-10|CRF2-S1|CRF2X|IL-22BP|IL-22R-alpha-2|IL-22RA2|ZCYTOR16	6	Interleukins_Receptor
IL23R	149233	interleukin 23 receptor	-	1	Interleukins_Receptor
IL27RA	9466	interleukin 27 receptor subunit alpha	CRL1|IL-27RA|IL27R|TCCR|WSX1|zcytor1	19	Interleukins_Receptor
IFNLR1	163702	interferon lambda receptor 1	CRF2/12|IFNLR|IL-28R1|IL28RA|LICR2	1	Interleukins_Receptor
IL2RA	3559	interleukin 2 receptor subunit alpha	CD25|IDDM10|IL2R|IMD41|TCGFR|p55	10	Interleukins_Receptor
IL2RB	3560	interleukin 2 receptor subunit beta	CD122|IL15RB|IMD63|P70-75	22	Interleukins_Receptor
IL2RG	3561	interleukin 2 receptor subunit gamma	CD132|CIDX|IL-2RG|IMD4|P64|SCIDX|SCI	X	Interleukins_Receptor

			DX1			
IL31RA	133396	interleukin 31 receptor A	CRL|CRL3|GLM-R|GLMR|GPL|IL-31RA|PLCA2|PRO21384|hGLM-R		5	Interleukins_Receptor
IL3RA	3563	interleukin 3 receptor subunit alpha	CD123|IL3R|IL3RAY|IL3RX|IL3RY|hIL-3Ra	X|Y		Interleukins_Receptor
IL4R	3566	interleukin 4 receptor	CD124|IL-4RA|IL4RA		16	Interleukins_Receptor
IL5RA	3568	interleukin 5 receptor subunit alpha	CD125|CDw125|HSIL5R3|IL5R		3	Interleukins_Receptor
IL6R	3570	interleukin 6 receptor	CD126|IL-6R-1|IL-6RA|IL6Q|IL6RA|IL6RQ|gp80		1	Interleukins_Receptor
IL7R	3575	interleukin 7 receptor	CD127|CDW127|IL-7R-alpha|IL7RA|ILRA		5	Interleukins_Receptor
CXCR1	3577	C-X-C motif chemokine receptor 1	C-C|C-C-CKR-1|CD128|CD181|CDw128a|CKR-1|CMKAR1|IL8R1|IL8RA|IL8RBA		2	Interleukins_Receptor
CXCR2	3579	C-X-C motif chemokine receptor 2	CD182|CDw128b|CMKAR2|IL8R2|IL8RA|IL8RB		2	Interleukins_Receptor
IL9R	3581	interleukin 9 receptor	CD129|IL-9R	X|Y		Interleukins_Receptor
ST2	6761	-	-		11	Interleukins_Receptor
HLA-A	3105	major histocompatibility complex, class I, A	HLAA		6	NaturalKiller_Cell_Cytotoxicity
HLA-B	3106	major histocompatibility complex, class I, B	AS|B-4901|HLAB		6	NaturalKiller_Cell_Cytotoxicity
HLA-C	3107	major histocompatibility complex, class I, C	D6S204|HLA-JY3|HLAC|HLC-C|MHC|PSORS1		6	NaturalKiller_Cell_Cytotoxicity
HLA-E	3133	major histocompatibility complex, class I, E	HLA-6.2|QA1		6	NaturalKiller_Cell_Cytotoxicity
HLA-G	3135	major histocompatibility complex, class I, G	MHC-G		6	NaturalKiller_Cell_Cytotoxicity
KIR3DL1	3811	killer cell immunoglobulin like receptor, three Ig domains and long cytoplasmic tail 1	CD158E1|KIR|KIR3DL1/S1|NKAT-3|NKAT3|NKB1|NKB1B		19	NaturalKiller_Cell_Cytotoxicity
KIR3DL2	3812	killer cell immunoglobulin like receptor, three Ig domains and long cytoplasmic tail 2	3DL2|CD158K|KIR-3DL2|NKAT-4|NKAT4|NKAT4B|p140		19	NaturalKiller_Cell_Cytotoxicity
KIR2D	3802	killer cell immunoglobulin like receptor, two	CD158A|KIR-K64|KIR221|KIR2DL3|NKAT|N		19	NaturalKiller_Cell_Cytotoxicity

<table>
<tr><td>L1</td><td></td><td>Ig domains and long cytoplasmic tail 1</td><td>KAT-1|NKAT1|p58.1</td><td></td><td></td></tr>
<tr><td>KIR2DL2</td><td>3803</td><td>killer cell immunoglobulin like receptor, two Ig domains and long cytoplasmic tail 2</td><td>CD158B1|CD158b|NKAT-6|NKAT6|p58.2</td><td>19</td><td>NaturalKiller_Cell_Cytotoxicity</td></tr>
<tr><td>KIR2DL3</td><td>3804</td><td>killer cell immunoglobulin like receptor, two Ig domains and long cytoplasmic tail 3</td><td>CD158B2|CD158b|GL183|KIR-023GB|KIR-K7b|KIR-K7c|KIR2DL|KIR2DS5|KIRCL23|NKAT|NKAT2|NKAT2A|NKAT2B|p58</td><td>19</td><td>NaturalKiller_Cell_Cytotoxicity</td></tr>
<tr><td>KIR2DL4</td><td>3805</td><td>killer cell immunoglobulin like receptor, two Ig domains and long cytoplasmic tail 4</td><td>CD158D|G9P|KIR-103AS|KIR-2DL4|KIR103|KIR103AS</td><td>19</td><td>NaturalKiller_Cell_Cytotoxicity</td></tr>
<tr><td>KIR2DL5A</td><td>57292</td><td>killer cell immunoglobulin like receptor, two Ig domains and long cytoplasmic tail 5A</td><td>CD158F|KIR2DL5|KIR2DL5.1|KIR2DL5.3</td><td>19</td><td>NaturalKiller_Cell_Cytotoxicity</td></tr>
<tr><td>KLRC1</td><td>3821</td><td>killer cell lectin like receptor C1</td><td>CD159A|NKG2|NKG2A</td><td>12</td><td>NaturalKiller_Cell_Cytotoxicity</td></tr>
<tr><td>KLRC2</td><td>3822</td><td>killer cell lectin like receptor C2</td><td>CD159c|NKG2-C|NKG2C</td><td>12</td><td>NaturalKiller_Cell_Cytotoxicity</td></tr>
<tr><td>KLRC3</td><td>3823</td><td>killer cell lectin like receptor C3</td><td>NKG2-E|NKG2E</td><td>12</td><td>NaturalKiller_Cell_Cytotoxicity</td></tr>
<tr><td>KLRD1</td><td>3824</td><td>killer cell lectin like receptor D1</td><td>CD94</td><td>12</td><td>NaturalKiller_Cell_Cytotoxicity</td></tr>
<tr><td>PTPN6</td><td>5777</td><td>protein tyrosine phosphatase non-receptor type 6</td><td>HCP|HCPH|HPTP1C|PTP-1C|SH-PTP1|SHP-1|SHP-1L|SHP1</td><td>12</td><td>NaturalKiller_Cell_Cytotoxicity</td></tr>
<tr><td>PTPN11</td><td>5781</td><td>protein tyrosine phosphatase non-receptor type 11</td><td>BPTP3|CFC|JMML|METCDS|NS1|PTP-1D|PTP2C|SH-PTP2|SH-PTP3|SHP2</td><td>12</td><td>NaturalKiller_Cell_Cytotoxicity</td></tr>
<tr><td>ICAM1</td><td>3383</td><td>intercellular adhesion molecule 1</td><td>BB2|CD54|P3.58</td><td>19</td><td>NaturalKiller_Cell_Cytotoxicity</td></tr>
<tr><td>ICAM2</td><td>3384</td><td>intercellular adhesion molecule 2</td><td>CD102</td><td>17</td><td>NaturalKiller_Cell_Cytotoxicity</td></tr>
<tr><td>ITGAL</td><td>3683</td><td>integrin subunit alpha L</td><td>CD11A|LFA-1|LFA1A</td><td>16</td><td>NaturalKiller_Cell_Cytotoxicity</td></tr>
<tr><td>ITGB2</td><td>3689</td><td>integrin subunit beta 2</td><td>CD18|LAD|LCAMB|LFA-1|MAC-1|MF17|MFI7</td><td>21</td><td>NaturalKiller_Cell_Cytotoxicity</td></tr>
<tr><td>PTK2B</td><td>2185</td><td>protein tyrosine kinase 2 beta</td><td>CADTK|CAKB|FADK2|FAK2|PKB|PTK|PYK2|RAFTK</td><td>8</td><td>NaturalKiller_Cell_Cytotoxicity</td></tr>
<tr><td>VAV3</td><td>10451</td><td>vav guanine nucleotide exchange factor 3</td><td>-</td><td>1</td><td>NaturalKiller_Cell_Cytotoxicity</td></tr>
</table>

VAV1	7409	vav guanine nucleotide exchange factor 1	VAV	19	NaturalKiller_Cell_Cytotoxicity
VAV2	7410	vav guanine nucleotide exchange factor 2	VAV-2	9	NaturalKiller_Cell_Cytotoxicity
RAC1	5879	Rac family small GTPase 1	MIG5|MRD48|Rac-1|TC-25|p21-Rac1	7	NaturalKiller_Cell_Cytotoxicity
RAC2	5880	Rac family small GTPase 2	EN-7|Gx|HSPC022|p21-Rac2	22	NaturalKiller_Cell_Cytotoxicity
RAC3	5881	Rac family small GTPase 3	-	17	NaturalKiller_Cell_Cytotoxicity
PAK1	5058	p21 (RAC1) activated kinase 1	IDDMSSD|PAKalpha|alpha-PAK|p65-PAK	11	NaturalKiller_Cell_Cytotoxicity
MAP2K1	5604	mitogen-activated protein kinase kinase 1	CFC3|MAPKK1|MEK1|MKK1|PRKMK1	15	NaturalKiller_Cell_Cytotoxicity
MAP2K2	5605	mitogen-activated protein kinase kinase 2	CFC4|MAPKK2|MEK2|MKK2|PRKMK2	19	NaturalKiller_Cell_Cytotoxicity
MAPK1	5594	mitogen-activated protein kinase 1	ERK|ERK-2|ERK2|ERT1|MAPK2|P42MAPK|PRKM1|PRKM2|p38|p40|p41|p41mapk|p42-MAPK	22	NaturalKiller_Cell_Cytotoxicity
MAPK3	5595	mitogen-activated protein kinase 3	ERK-1|ERK1|ERT2|HS44KDAP|HUMKER1A|P44ERK1|P44MAPK|PRKM3|p44-ERK1|p44-MAPK	16	NaturalKiller_Cell_Cytotoxicity
TNF	7124	tumor necrosis factor	DIF|TNF-alpha|TNFA|TNFSF2|TNLG1F	6	NaturalKiller_Cell_Cytotoxicity
CSF2	1437	colony stimulating factor 2	CSF|GMCSF	5	NaturalKiller_Cell_Cytotoxicity
IFNG	3458	interferon gamma	IFG|IFI	12	NaturalKiller_Cell_Cytotoxicity
KIR2DS1	3806	killer cell immunoglobulin like receptor, two Ig domains and short cytoplasmic tail 1	CD158H|CD158a|p50.1	19	NaturalKiller_Cell_Cytotoxicity
KIR2DS3	3808	killer cell immunoglobulin like receptor, two Ig domains and short cytoplasmic tail 3	NKAT7	19	NaturalKiller_Cell_Cytotoxicity
KIR2DS4	3809	killer cell immunoglobulin like receptor, two Ig domains and short cytoplasmic tail 4	CD158I|KIR-2DS4|KIR1D|KIR412|KKA3|NKAT-8|NKAT8	19	NaturalKiller_Cell_Cytotoxicity
KIR2D	3810	killer cell immunoglobulin like receptor, two	CD158G|NKAT9	19	NaturalKiller_Cell_Cytotoxicity

S5		Ig domains and short cytoplasmic tail 5			
NCR2	9436	natural cytotoxicity triggering receptor 2	CD336|LY95|NK-p44|NKP44|dJ149M18.1	6	NaturalKiller_Cell_Cytotoxicity
TYROBP	7305	transmembrane immune signaling adaptor TYROBP	DAP12|KARAP|PLOSL|PLOSL1	19	NaturalKiller_Cell_Cytotoxicity
LCK	3932	LCK proto-oncogene, Src family tyrosine kinase	IMD22|LSK|YT16|p56lck|pp58lck	1	NaturalKiller_Cell_Cytotoxicity
FCGR3A	2214	Fc fragment of IgG receptor IIIa	CD16|CD16A|FCG3|FCGR3|FCGRIII|FCR-10|FCRIII|FCRIIIA|IGFR3|IMD20	1	NaturalKiller_Cell_Cytotoxicity
FCGR3B	2215	Fc fragment of IgG receptor IIIb	CD16|CD16A|CD16b|FCG3|FCGR3|FCGR3A|FCR-10|FCRIII|FCRIIIb	1	NaturalKiller_Cell_Cytotoxicity
NCR1	9437	natural cytotoxicity triggering receptor 1	CD335|LY94|NK-p46|NKP46	19	NaturalKiller_Cell_Cytotoxicity
NCR3	259197	natural cytotoxicity triggering receptor 3	1C7|CD337|LY117|MALS|NKp30	6	NaturalKiller_Cell_Cytotoxicity
FCER1G	2207	Fc fragment of IgE receptor Ig	FCRG	1	NaturalKiller_Cell_Cytotoxicity
CD247	919	CD247 molecule	CD3-ZETA|CD3H|CD3Q|CD3Z|IMD25|T3Z|TCRZ	1	NaturalKiller_Cell_Cytotoxicity
ZAP70	7535	zeta chain of T cell receptor associated protein kinase 70	ADMIO2|IMD48|SRK|STCD|STD|TZK|ZAP-70	2	NaturalKiller_Cell_Cytotoxicity
SYK	6850	spleen associated tyrosine kinase	p72-Syk	9	NaturalKiller_Cell_Cytotoxicity
LCP2	3937	lymphocyte cytosolic protein 2	SLP-76|SLP76	5	NaturalKiller_Cell_Cytotoxicity
LAT	27040	linker for activation of T cells	IMD52|LAT1|pp36	16	NaturalKiller_Cell_Cytotoxicity
PLCG1	5335	phospholipase C gamma 1	NCKAP3|PLC-II|PLC1|PLC148|PLCgamma1	20	NaturalKiller_Cell_Cytotoxicity
PLCG2	5336	phospholipase C gamma 2	APLAID|FCAS3|PLC-IV|PLC-gamma-2	16	NaturalKiller_Cell_Cytotoxicity
SH3BP2	6452	SH3 domain binding protein 2	3BP-2|3BP2|CRBM|CRPM|RES4-23	4	NaturalKiller_Cell_Cytotoxicity

PIK3CA	5290	phosphatidylinositol-4,5-bisphosphate 3-kinase catalytic subunit alpha	CLAPO|CLOVE|CWS5|MCAP|MCM|MCMTC|PI3K|PI3K-alpha|p110-alpha	3	NaturalKiller_Cell_Cytotoxicity
PIK3CB	5291	phosphatidylinositol-4,5-bisphosphate 3-kinase catalytic subunit beta	P110BETA|PI3K|PI3KBETA|PIK3C1	3	NaturalKiller_Cell_Cytotoxicity
PIK3CD	5293	phosphatidylinositol-4,5-bisphosphate 3-kinase catalytic subunit delta	APDS|IMD14|P110DELTA|PI3K|p110D	1	NaturalKiller_Cell_Cytotoxicity
PIK3CG	5294	phosphatidylinositol-4,5-bisphosphate 3-kinase catalytic subunit gamma	PI3CG|PI3K|PI3Kgamma|PIK3|p110gamma|p120-PI3K	7	NaturalKiller_Cell_Cytotoxicity
PIK3R5	23533	phosphoinositide-3-kinase regulatory subunit 5	F730038I15Rik|FOAP-2|P101-PI3K|p101	17	NaturalKiller_Cell_Cytotoxicity
PIK3R1	5295	phosphoinositide-3-kinase regulatory subunit 1	AGM7|GRB1|IMD36|p85|p85-ALPHA	5	NaturalKiller_Cell_Cytotoxicity
PIK3R2	5296	phosphoinositide-3-kinase regulatory subunit 2	MPPH|MPPH1|P85B|p85|p85-BETA	19	NaturalKiller_Cell_Cytotoxicity
PIK3R3	8503	phosphoinositide-3-kinase regulatory subunit 3	p55|p55-GAMMA|p55PIK	1	NaturalKiller_Cell_Cytotoxicity
FYN	2534	FYN proto-oncogene, Src family tyrosine kinase	SLK|SYN|p59-FYN	6	NaturalKiller_Cell_Cytotoxicity
SHC2	25759	SHC adaptor protein 2	SCK|SHCB|SLI	19	NaturalKiller_Cell_Cytotoxicity
SHC4	399694	SHC adaptor protein 4	RaLP|SHCD	15	NaturalKiller_Cell_Cytotoxicity
SHC3	53358	SHC adaptor protein 3	N-Shc|NSHC|RAI|SHCC	9	NaturalKiller_Cell_Cytotoxicity
SHC1	6464	SHC adaptor protein 1	SHC|SHCA	1	NaturalKiller_Cell_Cytotoxicity
GRB2	2885	growth factor receptor bound protein 2	ASH|EGFRBP-GRB2|Grb3-3|MST084|MSTP084|NCKAP2	17	NaturalKiller_Cell_Cytotoxicity

SOS1	6654	SOS Ras/Rac guanine nucleotide exchange factor 1	GF1|GGF1|GINGF|HGF|NS4|SOS-1		2	NaturalKiller_Cell_Cytotoxicity
SOS2	6655	SOS Ras/Rho guanine nucleotide exchange factor 2	NS9|SOS-2		14	NaturalKiller_Cell_Cytotoxicity
HRAS	3265	HRas proto-oncogene, GTPase	C-BAS/HAS|C-H-RAS|C-HA-RAS1|CTLO|H-RASIDX|HAMSV|HRAS1|RASH1|p21ras		11	NaturalKiller_Cell_Cytotoxicity
KRAS	3845	KRAS proto-oncogene, GTPase	'C-K-RAS|C-K-RAS|CFC2|K-RAS2A|K-RAS2B|K-RAS4A|K-RAS4B|K-Ras|K-Ras 2|KI-RAS|KRAS1|KRAS2|NS|NS3|OES|RALD|RASK2|c-Ki-ras|c-Ki-ras2		12	NaturalKiller_Cell_Cytotoxicity
NRAS	4893	NRAS proto-oncogene, GTPase	ALPS4|CMNS|N-ras|NCMS|NRAS1|NS6		1	NaturalKiller_Cell_Cytotoxicity
ARAF	369	A-Raf proto-oncogene, serine/threonine kinase	A-RAF|ARAF1|PKS2|RAFA1	X		NaturalKiller_Cell_Cytotoxicity
BRAF	673	B-Raf proto-oncogene, serine/threonine kinase	B-RAF1|B-raf|BRAF1|NS7|RAFB1		7	NaturalKiller_Cell_Cytotoxicity
RAF1	5894	Raf-1 proto-oncogene, serine/threonine kinase	CMD1NN|CRAF|NS5|Raf-1|c-Raf		3	NaturalKiller_Cell_Cytotoxicity
MICA	100507436	MHC class I polypeptide-related sequence A	MIC-A|PERB11.1		6	NaturalKiller_Cell_Cytotoxicity
MICB	4277	MHC class I polypeptide-related sequence B	PERB11.2		6	NaturalKiller_Cell_Cytotoxicity
ULBP3	79465	UL16 binding protein 3	N2DL-3|NKG2DL3|RAET1N		6	NaturalKiller_Cell_Cytotoxicity
ULBP2	80328	UL16 binding protein 2	ALCAN-alpha|N2DL2|NKG2DL2|RAET1H|RAET1L		6	NaturalKiller_Cell_Cytotoxicity
ULBP1	80329	UL16 binding protein 1	N2DL-1|NKG2DL1|RAET1I		6	NaturalKiller_Cell_Cytotoxicity
KLRK1	22914	killer cell lectin like receptor K1	CD314|D12S2489E|KLR|NKG2-D|NKG2D		12	NaturalKiller_Cell_Cytotoxicity

HCST	10870	hematopoietic cell signal transducer	DAP10|KAP10|PIK3AP	19	NaturalKiller_Cell_Cytotoxicity
CD48	962	CD48 molecule	BCM1|BLAST|BLAST1|MEM-102|SLAMF2|hCD48|mCD48	1	NaturalKiller_Cell_Cytotoxicity
CD244	51744	CD244 molecule	2B4|NAIL|NKR2B4|Nmrk|SLAMF4	1	NaturalKiller_Cell_Cytotoxicity
PPP3CA	5530	protein phosphatase 3 catalytic subunit alpha	ACCIID|CALN|CALNA|CALNA1|CCN1|CNA1|IECEE|IECEE1|PPP2B	4	NaturalKiller_Cell_Cytotoxicity
PPP3CB	5532	protein phosphatase 3 catalytic subunit beta	CALNA2|CALNB|CNA2|PP2Bbeta	10	NaturalKiller_Cell_Cytotoxicity
PPP3CC	5533	protein phosphatase 3 catalytic subunit gamma	CALNA3|CNA3|PP2Bgamma	8	NaturalKiller_Cell_Cytotoxicity
CHP1	11261	calcineurin like EF-hand protein 1	CHP|SLC9A1BP|SPAX9|Sid470p|p22|p24	15	NaturalKiller_Cell_Cytotoxicity
PPP3R1	5534	protein phosphatase 3 regulatory subunit B, alpha	CALNB1|CNB|CNB1	2	NaturalKiller_Cell_Cytotoxicity
PPP3R2	5535	protein phosphatase 3 regulatory subunit B, beta	PPP3RL	9	NaturalKiller_Cell_Cytotoxicity
CHP2	63928	calcineurin like EF-hand protein 2	-	16	NaturalKiller_Cell_Cytotoxicity
NFAT5	10725	nuclear factor of activated T cells 5	NF-AT5|NFATL1|NFATZ|OREBP|TONEBP	16	NaturalKiller_Cell_Cytotoxicity
NFATC1	4772	nuclear factor of activated T cells 1	NF-ATC|NF-ATc1.2|NFAT2|NFATc	18	NaturalKiller_Cell_Cytotoxicity
NFATC2	4773	nuclear factor of activated T cells 2	NFAT1|NFATP	20	NaturalKiller_Cell_Cytotoxicity

NFATC3	4775	nuclear factor of activated T cells 3	NF-AT4c|NFAT4|NFATX	16	NaturalKiller_Cell_Cytotoxicity
NFATC4	4776	nuclear factor of activated T cells 4	NF-AT3|NF-ATC4|NFAT3	14	NaturalKiller_Cell_Cytotoxicity
PRKCA	5578	protein kinase C alpha	AAG6|PKC-alpha|PKCA|PKCI+/-|PKCalpha|PRKACA	17	NaturalKiller_Cell_Cytotoxicity
PRKCB	5579	protein kinase C beta	PKC-beta|PKCB|PKCI(2)|PKCbeta|PRKCB1|PRKCB2	16	NaturalKiller_Cell_Cytotoxicity
PRKCG	5582	protein kinase C gamma	PKC-gamma|PKCC|PKCG|PKCI(3)|PKCgamma|SCA14	19	NaturalKiller_Cell_Cytotoxicity
SH2D1B	117157	SH2 domain containing 1B	EAT2	1	NaturalKiller_Cell_Cytotoxicity
SH2D1A	4068	SH2 domain containing 1A	DSHP|EBVS|IMD5|LYP|MTCP1|SAP|SAP/SH2D1A|XLP|XLPD|XLPD1	X	NaturalKiller_Cell_Cytotoxicity
IFNGR1	3459	interferon gamma receptor 1	CD119|IFNGR|IMD27A|IMD27B	6	NaturalKiller_Cell_Cytotoxicity
IFNGR2	3460	interferon gamma receptor 2	AF-1|IFGR2|IFNGT1|IMD28	21	NaturalKiller_Cell_Cytotoxicity
IFNA1	3439	interferon alpha 1	IFL|IFN|IFN-ALPHA|IFN-alphaD|IFNA13|IFNA@|leIF D	9	NaturalKiller_Cell_Cytotoxicity
IFNA2	3440	interferon alpha 2	IFN-alpha-2|IFN-alphaA|IFNA|IFNA2B|leIF A	9	NaturalKiller_Cell_Cytotoxicity
IFNA4	3441	interferon alpha 4	IFN-alpha4a|INFA4	9	NaturalKiller_Cell_Cytotoxicity
IFNA5	3442	interferon alpha 5	IFN-alpha-5|IFN-alphaG|INA5|INFA5|leIF G	9	NaturalKiller_Cell_Cytotoxicity
IFNA6	3443	interferon alpha 6	IFN-alphaK	9	NaturalKiller_Cell_Cytotoxicity

IFNA7	3444	interferon alpha 7	IFN-alphaJ|IFNA-J	9	NaturalKiller_Cell_Cytotoxicity
IFNA8	3445	interferon alpha 8	IFN-alphaB	9	NaturalKiller_Cell_Cytotoxicity
IFNA10	3446	interferon alpha 10	IFN-alphaC	9	NaturalKiller_Cell_Cytotoxicity
IFNA13	3447	interferon alpha 13	-	9	NaturalKiller_Cell_Cytotoxicity
IFNA14	3448	interferon alpha 14	IFN-alphaH|LEIF2H	9	NaturalKiller_Cell_Cytotoxicity
IFNA16	3449	interferon alpha 16	IFN-alpha-16|IFN-alphaO	9	NaturalKiller_Cell_Cytotoxicity
IFNA17	3451	interferon alpha 17	IFN-alphaI|IFNA|INFA|LEIF2C1	9	NaturalKiller_Cell_Cytotoxicity
IFNA21	3452	interferon alpha 21	IFN-alphaI|LeIF F|leIF-F	9	NaturalKiller_Cell_Cytotoxicity
IFNB1	3456	interferon beta 1	IFB|IFF|IFN-beta|IFNB	9	NaturalKiller_Cell_Cytotoxicity
IFNAR1	3454	interferon alpha and beta receptor subunit 1	AVP|IFN-alpha-REC|IFNAR|IFNBR|IFRC	21	NaturalKiller_Cell_Cytotoxicity
IFNAR2	3455	interferon alpha and beta receptor subunit 2	IFN-R|IFN-alpha-REC|IFNABR|IFNARB|IMD45	21	NaturalKiller_Cell_Cytotoxicity
TNFSF10	8743	TNF superfamily member 10	APO2L|Apo-2L|CD253|TL2|TNLG6A|TRAIL	3	NaturalKiller_Cell_Cytotoxicity
TNFRSF10D	8793	TNF receptor superfamily member 10d	CD264|DCR2|TRAIL-R4|TRAILR4|TRUNDD	8	NaturalKiller_Cell_Cytotoxicity
TNFRSF10C	8794	TNF receptor superfamily member 10c	CD263|DCR1|DCR1-TNFR|LIT|TRAIL-R3|TRAILR3|TRID	8	NaturalKiller_Cell_Cytotoxicity
TNFRS	8795	TNF receptor superfamily member 10b	CD262|DR5|KILLER|KILLER/DR5|TRAIL-R2	8	NaturalKiller_Cell_Cytotoxicity

F10B			|TRAILR2|TRICK2|TRICK2A|TRICK2B|TRICKB|ZTNFR9		
TNFRSF10A	8797	TNF receptor superfamily member 10a	APO2|CD261|DR4|TRAILR-1|TRAILR1	8	NaturalKiller_Cell_Cytotoxicity
FASLG	356	Fas ligand	ALPS1B|APT1LG1|APTL|CD178|CD95-L|CD95L|FASL|TNFSF6|TNLG1A	1	NaturalKiller_Cell_Cytotoxicity
FAS	355	Fas cell surface death receptor	ALPS1A|APO-1|APT1|CD95|FAS1|FASTM|TNFRSF6	10	NaturalKiller_Cell_Cytotoxicity
GZMB	3002	granzyme B	C11|CCPI|CGL-1|CGL1|CSP-B|CSPB|CTLA1|CTSGL1|HLP|SECT	14	NaturalKiller_Cell_Cytotoxicity
PRF1	5551	perforin 1	HPLH2|P1|PFP	10	NaturalKiller_Cell_Cytotoxicity
CASP3	836	caspase 3	CPP32|CPP32B|SCA-1	4	NaturalKiller_Cell_Cytotoxicity
BID	637	BH3 interacting domain death agonist	FP497	22	NaturalKiller_Cell_Cytotoxicity
CD3D	915	CD3d molecule	CD3-DELTA|IMD19|T3D	11	TCRsignalingPathway
CD3E	916	CD3e molecule	IMD18|T3E|TCRE	11	TCRsignalingPathway
CD3G	917	CD3g molecule	CD3-GAMMA|IMD17|T3G	11	TCRsignalingPathway
CD247	919	CD247 molecule	CD3-ZETA|CD3H|CD3Q|CD3Z|IMD25|T3Z|TCRZ	1	TCRsignalingPathway
CD4	920	CD4 molecule	CD4mut	12	TCRsignalingPathway
CD8A	925	CD8a molecule	CD8|Leu2|p32	2	TCRsignalingPathway
CD8B	926	CD8b molecule	CD8B1|LEU2|LY3|LYT3|P37	2	TCRsignalingPathway
PTPRC	5788	protein tyrosine phosphatase receptor type C	B220|CD45|CD45R|GP180|L-CA|LCA|LY5|T200	1	TCRsignalingPathway
LCK	3932	LCK proto-oncogene, Src family tyrosine kinase	IMD22|LSK|YT16|p56lck|pp58lck	1	TCRsignalingPathway
FYN	2534	FYN proto-oncogene, Src family tyrosine kinase	SLK|SYN|p59-FYN	6	TCRsignalingPathway
ZAP70	7535	zeta chain of T cell receptor associated	ADMIO2|IMD48|SRK|STCD|STD|TZK|ZAP-70	2	TCRsignalingPathway

		protein kinase 70			
LCP2	3937	lymphocyte cytosolic protein 2	SLP-76|SLP76	5	TCRsignalingPathway
LAT	27040	linker for activation of T cells	IMD52|LAT1|pp36	16	TCRsignalingPathway
ITK	3702	IL2 inducible T cell kinase	EMT|LPFS1|LYK|PSCTK2	5	TCRsignalingPathway
TEC	7006	tec protein tyrosine kinase	PSCTK4	4	TCRsignalingPathway
NCK1	4690	NCK adaptor protein 1	NCK|NCKalpha|nck-1	3	TCRsignalingPathway
NCK2	8440	NCK adaptor protein 2	GRB4|NCKbeta	2	TCRsignalingPathway
VAV3	10451	vav guanine nucleotide exchange factor 3	-	1	TCRsignalingPathway
VAV1	7409	vav guanine nucleotide exchange factor 1	VAV	19	TCRsignalingPathway
VAV2	7410	vav guanine nucleotide exchange factor 2	VAV-2	9	TCRsignalingPathway
GRAP2	9402	GRB2 related adaptor protein 2	GADS|GRAP-2|GRB2L|GRBLG|GRID|GRPL|GrbX|Grf40|Mona|P38	22	TCRsignalingPathway
GRB2	2885	growth factor receptor bound protein 2	ASH|EGFRBP-GRB2|Grb3-3|MST084|MSTP084|NCKAP2	17	TCRsignalingPathway
PAK1	5058	p21 (RAC1) activated kinase 1	IDDMSSD|PAKalpha|alpha-PAK|p65-PAK	11	TCRsignalingPathway
PAK2	5062	p21 (RAC1) activated kinase 2	PAK65|PAKgamma	3	TCRsignalingPathway
PAK3	5063	p21 (RAC1) activated kinase 3	ARA|MRX30|MRX47|OPHN3|PAK-3|PAK3beta|bPAK|beta-PAK	X	TCRsignalingPathway
PAK4	10298	p21 (RAC1) activated kinase 4	-	19	TCRsignalingPathway
PAK6	56924	p21 (RAC1) activated kinase 6	PAK5	15	TCRsignalingPathway
PAK5	57144	p21 (RAC1) activated kinase 5	PAK7	20	TCRsignalingPathway

RHOA	387	ras homolog family member A	ARH12|ARHA|EDFAOB|RHO12|RHOH12	3	TCRsignalingPathway
CDC42	998	cell division cycle 42	CDC42Hs|G25K|TKS	1	TCRsignalingPathway
PPP3CA	5530	protein phosphatase 3 catalytic subunit alpha	ACCIID|CALN|CALNA|CALNA1|CCN1|CNA1|IECEE|IECEE1|PPP2B	4	TCRsignalingPathway
PPP3CB	5532	protein phosphatase 3 catalytic subunit beta	CALNA2|CALNB|CNA2|PP2Bbeta	10	TCRsignalingPathway
PPP3CC	5533	protein phosphatase 3 catalytic subunit gamma	CALNA3|CNA3|PP2Bgamma	8	TCRsignalingPathway
CHP1	11261	calcineurin like EF-hand protein 1	CHP|SLC9A1BP|SPAX9|Sid470p|p22|p24	15	TCRsignalingPathway
PPP3R1	5534	protein phosphatase 3 regulatory subunit B, alpha	CALNB1|CNB|CNB1	2	TCRsignalingPathway
PPP3R2	5535	protein phosphatase 3 regulatory subunit B, beta	PPP3RL	9	TCRsignalingPathway
CHP2	63928	calcineurin like EF-hand protein 2	-	16	TCRsignalingPathway
NFAT5	10725	nuclear factor of activated T cells 5	NF-AT5|NFATL1|NFATZ|OREBP|TONEBP	16	TCRsignalingPathway
NFATC1	4772	nuclear factor of activated T cells 1	NF-ATC|NF-ATc1.2|NFAT2|NFATc	18	TCRsignalingPathway
NFATC2	4773	nuclear factor of activated T cells 2	NFAT1|NFATP	20	TCRsignalingPathway
NFATC3	4775	nuclear factor of activated T cells 3	NF-AT4c|NFAT4|NFATX	16	TCRsignalingPathway
NFATC4	4776	nuclear factor of activated T cells 4	NF-AT3|NF-ATC4|NFAT3	14	TCRsignalingPathway

SOS1	6654	SOS Ras/Rac guanine nucleotide exchange factor 1	GF1|GGF1|GINGF|HGF|NS4|SOS-1		2	TCRsignalingPathway
SOS2	6655	SOS Ras/Rho guanine nucleotide exchange factor 2	NS9|SOS-2		14	TCRsignalingPathway
HRAS	3265	HRas proto-oncogene, GTPase	C-BAS/HAS|C-H-RAS|C-HA-RAS1|CTLO|H-RASIDX|HAMSV|HRAS1|RASH1|p21ras		11	TCRsignalingPathway
KRAS	3845	KRAS proto-oncogene, GTPase	'C-K-RAS|C-K-RAS|CFC2|K-RAS2A|K-RAS2B|K-RAS4A|K-RAS4B|K-Ras|K-Ras 2|KI-RAS|KRAS1|KRAS2|NS|NS3|OES|RALD|RASK2|c-Ki-ras|c-Ki-ras2		12	TCRsignalingPathway
NRAS	4893	NRAS proto-oncogene, GTPase	ALPS4|CMNS|N-ras|NCMS|NRAS1|NS6		1	TCRsignalingPathway
FOS	2353	Fos proto-oncogene, AP-1 transcription factor subunit	AP-1|C-FOS|p55		14	TCRsignalingPathway
JUN	3725	Jun proto-oncogene, AP-1 transcription factor subunit	AP-1|AP1|c-Jun|cJUN|p39		1	TCRsignalingPathway
CARD11	84433	caspase recruitment domain family member 11	BENTA|BIMP3|CARMA1|IMD11|IMD11A|PPBL		7	TCRsignalingPathway
BCL10	8915	BCL10 immune signaling adaptor	CARMEN|CIPER|CLAP|IMD37|c-E10|mE10		1	TCRsignalingPathway
MALT1	10892	MALT1 paracaspase	IMD12|MLT|MLT1|PCASP1		18	TCRsignalingPathway
CHUK	1147	component of inhibitor of nuclear factor kappa B kinase complex	IKBKA|IKK-alpha|IKK1|IKKA|NFKBIKA|TCF16		10	TCRsignalingPathway
IKBKB	3551	inhibitor of nuclear factor kappa B kinase subunit beta	IKK-beta|IKK2|IKKB|IMD15|IMD15A|IMD15B|NFKBIKB		8	TCRsignalingPathway
IKBKG	8517	inhibitor of nuclear factor kappa B kinase regulatory subunit gamma	AMCBX1|EDAID1|FIP-3|FIP3|Fip3p|IKK-gamma|IKKAP1|IKKG|IMD33|IP|IP1|IP2|IP	X		TCRsignalingPathway

			D2|NEMO|ZC2HC9		
NFKB1	4790	nuclear factor kappa B subunit 1	CVID12|EBP-1|KBF1|NF-kB|NF-kB1|NF-kappa-B1|NF-kappaB|NF-kappabeta|NFKB-p105|NFKB-p50|NFkappaB	4	TCRsignalingPathway
RELA	5970	RELA proto-oncogene, NF-kB subunit	CMCU|NFKB3|p65	11	TCRsignalingPathway
NFKBIA	4792	NFKB inhibitor alpha	EDAID2|IKBA|MAD-3|NFKBI	14	TCRsignalingPathway
NFKBIB	4793	NFKB inhibitor beta	IKBB|TRIP9	19	TCRsignalingPathway
NFKBIE	4794	NFKB inhibitor epsilon	IKBE	6	TCRsignalingPathway
CD28	940	CD28 molecule	Tp44	2	TCRsignalingPathway
ICOS	29851	inducible T cell costimulator	AILIM|CD278|CVID1	2	TCRsignalingPathway
CD40LG	959	CD40 ligand	CD154|CD40L|HIGM1|IGM|IMD3|T-BAM|TNFSF5|TRAP|gp39|hCD40L	X	TCRsignalingPathway
PIK3R5	23533	phosphoinositide-3-kinase regulatory subunit 5	F730038I15Rik|FOAP-2|P101-PI3K|p101	17	TCRsignalingPathway
PIK3R1	5295	phosphoinositide-3-kinase regulatory subunit 1	AGM7|GRB1|IMD36|p85|p85-ALPHA	5	TCRsignalingPathway
PIK3R2	5296	phosphoinositide-3-kinase regulatory subunit 2	MPPH|MPPH1|P85B|p85|p85-BETA	19	TCRsignalingPathway
PIK3R3	8503	phosphoinositide-3-kinase regulatory subunit 3	p55|p55-GAMMA|p55PIK	1	TCRsignalingPathway
PIK3CA	5290	phosphatidylinositol-4,5-bisphosphate 3-kinase catalytic subunit alpha	CLAPO|CLOVE|CWS5|MCAP|MCM|MCMTC|PI3K|PI3K-alpha|p110-alpha	3	TCRsignalingPathway

PIK3CB	5291	phosphatidylinositol-4,5-bisphosphate 3-kinase catalytic subunit beta	P110BETA|PI3K|PI3KBETA|PIK3C1	3	TCRsignalingPathway
PIK3CD	5293	phosphatidylinositol-4,5-bisphosphate 3-kinase catalytic subunit delta	APDS|IMD14|P110DELTA|PI3K|p110D	1	TCRsignalingPathway
PIK3CG	5294	phosphatidylinositol-4,5-bisphosphate 3-kinase catalytic subunit gamma	PI3CG|PI3K|PI3Kgamma|PIK3|p110gamma|p120-PI3K	7	TCRsignalingPathway
AKT3	10000	AKT serine/threonine kinase 3	MPPH|MPPH2|PKB-GAMMA|PKBG|PRKBG|RAC-PK-gamma|RAC-gamma|STK-2	1	TCRsignalingPathway
AKT1	207	AKT serine/threonine kinase 1	AKT|CWS6|PKB|PKB-ALPHA|PRKBA|RAC|RAC-ALPHA	14	TCRsignalingPathway
AKT2	208	AKT serine/threonine kinase 2	HIHGHH|PKBB|PKBBETA|PRKBB|RAC-BETA	19	TCRsignalingPathway
MAP3K8	1326	mitogen-activated protein kinase kinase kinase 8	AURA2|COT|EST|ESTF|MEKK8|TPL2|Tpl-2|c-COT	10	TCRsignalingPathway
MAP3K14	9020	mitogen-activated protein kinase kinase kinase 14	FTDCR1B|HS|HSNIK|NIK	17	TCRsignalingPathway
PDCD1	5133	programmed cell death 1	CD279|PD-1|PD1|SLEB2|hPD-1|hPD-1|hSLE1	2	TCRsignalingPathway
CTLA4	1493	cytotoxic T-lymphocyte associated protein 4	ALPS5|CD|CD152|CELIAC3|CTLA-4|GRD4|GSE|IDDM12	2	TCRsignalingPathway
PTPN6	5777	protein tyrosine phosphatase non-receptor type 6	HCP|HCPH|HPTP1C|PTP-1C|SH-PTP1|SHP-1|SHP-1L|SHP1	12	TCRsignalingPathway
CBLC	23624	Cbl proto-oncogene C	CBL-3|CBL-SL|RNF57	19	TCRsignalingPathway
CBL	867	Cbl proto-oncogene	C-CBL|CBL2|FRA11B|NSLL|RNF55	11	TCRsignalingPathway
CBLB	868	Cbl proto-oncogene B	Cbl-b|Nbla00127|RNF56	3	TCRsignalingPathway
IL2	3558	interleukin 2	IL-2|TCGF|lymphokine	4	TCRsignalingPathway

IL4	3565	interleukin 4	BCGF-1|BCGF1|BSF-1|BSF1|IL-4	5	TCRsignalingPathway
IL5	3567	interleukin 5	EDF|IL-5|TRF	5	TCRsignalingPathway
IL10	3586	interleukin 10	CSIF|GVHDS|IL-10|IL10A|TGIF	1	TCRsignalingPathway
IFNG	3458	interferon gamma	IFG|IFI	12	TCRsignalingPathway
CSF2	1437	colony stimulating factor 2	CSF|GMCSF	5	TCRsignalingPathway
TNF	7124	tumor necrosis factor	DIF|TNF-alpha|TNFA|TNFSF2|TNLG1F	6	TCRsignalingPathway
CDK4	1019	cyclin dependent kinase 4	CMM3|PSK-J3	12	TCRsignalingPathway
RASGRP1	10125	RAS guanyl releasing protein 1	CALDAG-GEFI|CALDAG-GEFII|IMD64|RASGRP	15	TCRsignalingPathway
PDK1	5163	pyruvate dehydrogenase kinase 1	-	2	TCRsignalingPathway
PLCG1	5335	phospholipase C gamma 1	NCKAP3|PLC-II|PLC1|PLC148|PLCgamma1	20	TCRsignalingPathway
PRKCQ	5588	protein kinase C theta	PRKCT|nPKC-theta	10	TCRsignalingPathway
TRAC	28755	T cell receptor alpha constant	IMD7|TCRA|TRA|TRCA	14	TCRsignalingPathway
TRAJ1	28754	T cell receptor alpha joining 1 (non-functional)	-	14	TCRsignalingPathway
TRAJ2	28753	T cell receptor alpha joining 2 (non-functional)	-	14	TCRsignalingPathway
TRAJ3	28752	T cell receptor alpha joining 3	-	14	TCRsignalingPathway
TRAJ4	28751	T cell receptor alpha joining 4	-	14	TCRsignalingPathway
TRAJ5	28750	T cell receptor alpha joining 5	-	14	TCRsignalingPathway
TRAJ6	28749	T cell receptor alpha joining 6	-	14	TCRsignalingPathway

TRAJ7	28748	T cell receptor alpha joining 7	-	14	TCRsignalingPathway
TRAJ8	28747	T cell receptor alpha joining 8	-	14	TCRsignalingPathway
TRAJ9	28746	T cell receptor alpha joining 9	-	14	TCRsignalingPathway
TRAJ10	28745	T cell receptor alpha joining 10	-	14	TCRsignalingPathway
TRAJ11	28744	T cell receptor alpha joining 11	-	14	TCRsignalingPathway
TRAJ12	28743	T cell receptor alpha joining 12	-	14	TCRsignalingPathway
TRAJ13	28742	T cell receptor alpha joining 13	-	14	TCRsignalingPathway
TRAJ14	28741	T cell receptor alpha joining 14	-	14	TCRsignalingPathway
TRAJ15	28740	T cell receptor alpha joining 15	-	14	TCRsignalingPathway
TRAJ16	28739	T cell receptor alpha joining 16	-	14	TCRsignalingPathway
TRAJ17	28738	T cell receptor alpha joining 17	-	14	TCRsignalingPathway
TRAJ18	28737	T cell receptor alpha joining 18	-	14	TCRsignalingPathway
TRAJ19	28736	T cell receptor alpha joining 19 (non-functional)	-	14	TCRsignalingPathway

TRAJ20	28735	T cell receptor alpha joining 20	-	14	TCRsignalingPathway
TRAJ21	28734	T cell receptor alpha joining 21	-	14	TCRsignalingPathway
TRAJ22	28733	T cell receptor alpha joining 22	-	14	TCRsignalingPathway
TRAJ23	28732	T cell receptor alpha joining 23	-	14	TCRsignalingPathway
TRAJ24	28731	T cell receptor alpha joining 24	-	14	TCRsignalingPathway
TRAJ25	28730	T cell receptor alpha joining 25 (non-functional)	-	14	TCRsignalingPathway
TRAJ26	28729	T cell receptor alpha joining 26	-	14	TCRsignalingPathway
TRAJ27	28728	T cell receptor alpha joining 27	-	14	TCRsignalingPathway
TRAJ28	28727	T cell receptor alpha joining 28	-	14	TCRsignalingPathway
TRAJ29	28726	T cell receptor alpha joining 29	-	14	TCRsignalingPathway
TRAJ30	28725	T cell receptor alpha joining 30	-	14	TCRsignalingPathway
TRAJ31	28724	T cell receptor alpha joining 31	-	14	TCRsignalingPathway
TRAJ32	28723	T cell receptor alpha joining 32	-	14	TCRsignalingPathway

TRAJ33	28722	T cell receptor alpha joining 33	-	14	TCRsignalingPathway
TRAJ34	28721	T cell receptor alpha joining 34	-	14	TCRsignalingPathway
TRAJ35	28720	T cell receptor alpha joining 35 (non-functional)	-	14	TCRsignalingPathway
TRAJ36	28719	T cell receptor alpha joining 36	-	14	TCRsignalingPathway
TRAJ37	28718	T cell receptor alpha joining 37	-	14	TCRsignalingPathway
TRAJ38	28717	T cell receptor alpha joining 38	-	14	TCRsignalingPathway
TRAJ39	28716	T cell receptor alpha joining 39	-	14	TCRsignalingPathway
TRAJ40	28715	T cell receptor alpha joining 40	-	14	TCRsignalingPathway
TRAJ41	28714	T cell receptor alpha joining 41	-	14	TCRsignalingPathway
TRAJ42	28713	T cell receptor alpha joining 42	-	14	TCRsignalingPathway
TRAJ43	28712	T cell receptor alpha joining 43	-	14	TCRsignalingPathway
TRAJ44	28711	T cell receptor alpha joining 44	-	14	TCRsignalingPathway
TRAJ45	28710	T cell receptor alpha joining 45	-	14	TCRsignalingPathway

TRAJ46	28709	T cell receptor alpha joining 46	-	14	TCRsignalingPathway
TRAJ47	28708	T cell receptor alpha joining 47	-	14	TCRsignalingPathway
TRAJ48	28707	T cell receptor alpha joining 48	-	14	TCRsignalingPathway
TRAJ49	28706	T cell receptor alpha joining 49	-	14	TCRsignalingPathway
TRAJ50	28705	T cell receptor alpha joining 50	-	14	TCRsignalingPathway
TRAJ52	28703	T cell receptor alpha joining 52	-	14	TCRsignalingPathway
TRAJ53	28702	T cell receptor alpha joining 53	-	14	TCRsignalingPathway
TRAJ54	28701	T cell receptor alpha joining 54	-	14	TCRsignalingPathway
TRAJ56	28699	T cell receptor alpha joining 56	-	14	TCRsignalingPathway
TRAJ57	28698	T cell receptor alpha joining 57	-	14	TCRsignalingPathway
TRAJ58	28697	T cell receptor alpha joining 58 (non-functional)	-	14	TCRsignalingPathway
TRAJ59	28696	T cell receptor alpha joining 59 (non-functional)	-	14	TCRsignalingPathway
TRAJ61	28694	T cell receptor alpha joining 61 (non-functional)	-	14	TCRsignalingPathway

TRAV1-1	28693	T cell receptor alpha variable 1-1	TCRAV1S1|TCRAV7S1|TRAV11	14	TCRsignalingPathway
TRAV1-2	28692	T cell receptor alpha variable 1-2	TCRAV1S2|TCRAV7S2|TRAV12	14	TCRsignalingPathway
TRAV2	28691	T cell receptor alpha variable 2	TCRAV11S1|TCRAV2S1	14	TCRsignalingPathway
TRAV3	28690	T cell receptor alpha variable 3	TCRAV16S1|TCRAV3S1	14	TCRsignalingPathway
TRAV4	28689	T cell receptor alpha variable 4	TCRAV20S1|TCRAV4S1	14	TCRsignalingPathway
TRAV5	28688	T cell receptor alpha variable 5	TCRAV15S1|TCRAV5S1	14	TCRsignalingPathway
TRAV7	28686	T cell receptor alpha variable 7	TCRAV7S1	14	TCRsignalingPathway
TRAV8-1	28685	T cell receptor alpha variable 8-1	TCRAV1S1|TCRAV8S1|TRAV81	14	TCRsignalingPathway
TRAV8-2	28684	T cell receptor alpha variable 8-2	TCRAV1S5|TCRAV8S2|TRAV82	14	TCRsignalingPathway
TRAV8-3	28683	T cell receptor alpha variable 8-3	TCRAV1S4|TCRAV8S3|TRAV83	14	TCRsignalingPathway
TRAV8-4	28682	T cell receptor alpha variable 8-4	TCRAV1S2|TCRAV8S4|TRAV84	14	TCRsignalingPathway
TRAV8-6	28680	T cell receptor alpha variable 8-6	TCRAV1S3|TCRAV8S6|TRAV86	14	TCRsignalingPathway
TRAV8-7	28679	T cell receptor alpha variable 8-7 (pseudogene)	TCRAV8S7|TRAV87	14	TCRsignalingPathway

TRAV9-1	28678	T cell receptor alpha variable 9-1	TCRAV9S1|TRAV91	14	TCRsignalingPathway
TRAV9-2	28677	T cell receptor alpha variable 9-2	TCRAV22S1|TCRAV9S2|TRAV92	14	TCRsignalingPathway
TRAV10	28676	T cell receptor alpha variable 10	TCRAV10S1|TCRAV24S1	14	TCRsignalingPathway
TRAV12-1	28674	T cell receptor alpha variable 12-1	TCRAV12S1|TCRAV2S3|TRAV121	14	TCRsignalingPathway
TRAV12-2	28673	T cell receptor alpha variable 12-2	TCRAV12S2|TCRAV2S1|TRAV122	14	TCRsignalingPathway
TRAV12-3	28672	T cell receptor alpha variable 12-3	TCRAV12S3|TCRAV2S2|TRAV123	14	TCRsignalingPathway
TRAV13-1	28671	T cell receptor alpha variable 13-1	TCRAV13S1|TCRAV8S1|TRAV131	14	TCRsignalingPathway
TRAV13-2	28670	T cell receptor alpha variable 13-2	TCRAV13S2|TCRAV8S2|TRAV132	14	TCRsignalingPathway
TRAV14DV4	28669	T cell receptor alpha variable 14/delta variable 4	TCRAV6S1-hDV104S1|TRAV14/DV4|hADV14S1	14	TCRsignalingPathway
TRAV16	28667	T cell receptor alpha variable 16	TCRAV16S1|TCRAV9S1	14	TCRsignalingPathway
TRAV17	28666	T cell receptor alpha variable 17	TCRAV17S1|TCRAV3S1	14	TCRsignalingPathway
TRAV18	28665	T cell receptor alpha variable 18	TCRAV18S1	14	TCRsignalingPathway
TRAV19	28664	T cell receptor alpha variable 19	TCRAV12S1|TCRAV19S1	14	TCRsignalingPathway

TRAV20	28663	T cell receptor alpha variable 20	TCRAV20S1|TCRAV30S1	14	TCRsignalingPathway
TRAV21	28662	T cell receptor alpha variable 21	TCRAV21S1|TCRAV23S1	14	TCRsignalingPathway
TRAV22	28661	T cell receptor alpha variable 22	TCRAV13S1|TCRAV22S1	14	TCRsignalingPathway
TRAV23DV6	28660	T cell receptor alpha variable 23/delta variable 6	TCRAV17S1|TRAV23/DV6|hADV23S1	14	TCRsignalingPathway
TRAV24	28659	T cell receptor alpha variable 24	TCRAV18S1|TCRAV24S1	14	TCRsignalingPathway
TRAV25	28658	T cell receptor alpha variable 25	TCRAV25S1|TCRAV32S1	14	TCRsignalingPathway
TRAV26-1	28657	T cell receptor alpha variable 26-1	TCRAV26S1|TCRAV4S2|TRAV261	14	TCRsignalingPathway
TRAV26-2	28656	T cell receptor alpha variable 26-2	TCRAV26S2|TCRAV4S1|TRAV262	14	TCRsignalingPathway
TRAV27	28655	T cell receptor alpha variable 27	TCRAV10S1|TCRAV27S1	14	TCRsignalingPathway
TRAV29DV5	28653	T cell receptor alpha variable 29/delta variable 5	TCRA|TCRAV21S1|TRAV29/DV5|hADV29S1	14	TCRsignalingPathway
TRAV30	28652	T cell receptor alpha variable 30	TCRAV29S1|TCRAV30S1	14	TCRsignalingPathway
TRAV34	28648	T cell receptor alpha variable 34	TCRAV26S1|TCRAV34S1	14	TCRsignalingPathway
TRAV35	28647	T cell receptor alpha variable 35	TCRAV25S1|TCRAV35S1	14	TCRsignalingPathway

TRAV36DV7	28646	T cell receptor alpha variable 36/delta variable 7	TCRAV28S1|TRAV36/DV7|hADV36S1	14	TCRsignalingPathway
TRAV38-1	28644	T cell receptor alpha variable 38-1	TCRAV14S2|TCRAV38S1|TRAV381	14	TCRsignalingPathway
TRAV38-2DV8	28643	T cell receptor alpha variable 38-2/delta variable 8	TCRAV14S1|TRAV382DV8|hADV38S2	14	TCRsignalingPathway
TRAV39	28642	T cell receptor alpha variable 39	TCRAV27S1|TCRAV39S1	14	TCRsignalingPathway
TRAV40	28641	T cell receptor alpha variable 40	TCRAV31S1|TCRAV40S1	14	TCRsignalingPathway
TRAV41	28640	T cell receptor alpha variable 41	TCRAV19S1|TCRAV41S1	14	TCRsignalingPathway
TRBC1	28639	T cell receptor beta constant 1	BV05S1J2.2|TCRB|TCRBC1	7	TCRsignalingPathway
TRBC2	28638	T cell receptor beta constant 2	TCRBC2	7	TCRsignalingPathway
TRBD1	28637	T cell receptor beta diversity 1	TCRBD1	7	TCRsignalingPathway
TRBD2	28636	T cell receptor beta diversity 2	TCRBD2	7	TCRsignalingPathway
TRBJ1-1	28635	T cell receptor beta joining 1-1	TCRBJ1S1|TRBJ11	7	TCRsignalingPathway
TRBJ1-2	28634	T cell receptor beta joining 1-2	TCRBJ1S2|TRBJ12	7	TCRsignalingPathway
TRBJ1	2863	T cell receptor beta joining 1-3	TCRBJ1S3|TRBJ13	7	TCRsignalingPathway

-3	3				
TRBJ1-4	28632	T cell receptor beta joining 1-4	TCRBJ1S4|TRBJ14	7	TCRsignalingPathway
TRBJ1-5	28631	T cell receptor beta joining 1-5	TCRBJ1S5|TRBJ15	7	TCRsignalingPathway
TRBJ1-6	28630	T cell receptor beta joining 1-6	TCRBJ1S6|TRBJ16	7	TCRsignalingPathway
TRBJ2-1	28629	T cell receptor beta joining 2-1	TCRBJ2S1|TRBJ21	7	TCRsignalingPathway
TRBJ2-2	28628	T cell receptor beta joining 2-2	TCRBJ2S2|TRBJ22	7	TCRsignalingPathway
TRBJ2-3	28626	T cell receptor beta joining 2-3	TCRBJ2S3|TRBJ23	7	TCRsignalingPathway
TRBJ2-4	28625	T cell receptor beta joining 2-4	TCRBJ2S4|TRBJ24	7	TCRsignalingPathway
TRBJ2-5	28624	T cell receptor beta joining 2-5	TCRBJ2S5|TRBJ25	7	TCRsignalingPathway
TRBJ2-6	28623	T cell receptor beta joining 2-6	TCRBJ2S6|TRBJ26	7	TCRsignalingPathway
TRBJ2-7	28622	T cell receptor beta joining 2-7	TCRBJ2S7|TRBJ27	7	TCRsignalingPathway
TRBV2	28620	T cell receptor beta variable 2	TCRBV22S1A2N1T|TCRBV2S1	7	TCRsignalingPathway
TRBV3-1	28619	T cell receptor beta variable 3-1	TCRBV3S1|TCRBV9S1A1T|TRBV31	7	TCRsignalingPathway
TRBV4	2861	T cell receptor beta variable 4-1	BV07S1J2.7|TCRBV4S1|TCRBV7S1A1N2T|TR	7	TCRsignalingPathway

-1	7		BV41		
TRBV4-2	28616	T cell receptor beta variable 4-2	TCRBV4S2|TCRBV7S3A2|TCRBV7S3A2T|TRBV42	7	TCRsignalingPathway
TRBV4-3	28615	T cell receptor beta variable 4-3	TCRBV4S3|TCRBV7S2A1N4T|TRBV43	7	TCRsignalingPathway
TRBV5-1	28614	T cell receptor beta variable 5-1	TCRBV5S1|TCRBV5S1A1T|TRBV51	7	TCRsignalingPathway
TRBV5-4	28611	T cell receptor beta variable 5-4	TCRBV5S4|TCRBV5S6A3N2T|TRBV54	7	TCRsignalingPathway
TRBV5-5	28610	T cell receptor beta variable 5-5	TCRBV5S3A2T|TCRBV5S5|TRBV55	7	TCRsignalingPathway
TRBV5-6	28609	T cell receptor beta variable 5-6	TCRBV5S2|TCRBV5S6|TRBV56	7	TCRsignalingPathway
TRBV5-7	28608	T cell receptor beta variable 5-7 (non-functional)	TCRBV5S7|TCRBV5S7P|TRBV57	7	TCRsignalingPathway
TRBV5-8	28607	T cell receptor beta variable 5-8	TCRBV5S4A2T|TCRBV5S8|TRBV58	7	TCRsignalingPathway
TRBV6-1	28606	T cell receptor beta variable 6-1	TCRBV13S3|TCRBV6S1|TRBV61	7	TCRsignalingPathway
TRBV6-2	28605	T cell receptor beta variable 6-2	TCRBV13S2|TCRBV13S2A1T|TCRBV6S2|TRBV62	7	TCRsignalingPathway
TRBV6-3	28604	T cell receptor beta variable 6-3	TCRBV13S9/13S2A1T|TCRBV6S3|TRBV63	7	TCRsignalingPathway
TRBV6-4	28603	T cell receptor beta variable 6-4	TCRBV13S5|TCRBV6S4|TRBV64	7	TCRsignalingPathway
TRBV6	2860	T cell receptor beta variable 6-5	TCRBV13S1|TCRBV6S5|TRBV65	7	TCRsignalingPathway

-5	2				
TRBV6 -6	2860 1	T cell receptor beta variable 6-6	TCRBV13S6A2T|TCRBV6S6|TRBV66	7	TCRsignalingPathway
TRBV6 -7	2860 0	T cell receptor beta variable 6-7 (non-functional)	TCRBV13S8P|TCRBV6S7|TRBV67	7	TCRsignalingPathway
TRBV6 -8	2859 9	T cell receptor beta variable 6-8	TCRBV13S7P|TCRBV6S8|TRBV68	7	TCRsignalingPathway
TRBV6 -9	2859 8	T cell receptor beta variable 6-9	TCRBV13S4|TCRBV6S9|TRBV69	7	TCRsignalingPathway
TRBV7 -2	2859 6	T cell receptor beta variable 7-2	TCRBV6S5A1N1|TCRBV6S5A2|TCRBV7S2|TRB V72	7	TCRsignalingPathway
TRBV7 -3	2859 5	T cell receptor beta variable 7-3	TCRBV6S1A1N1|TCRBV7S3|TRBV73	7	TCRsignalingPathway
TRBV7 -4	2859 4	T cell receptor beta variable 7-4	TCRBV6S8A2T|TCRBV7S4|TRBV74	7	TCRsignalingPathway
TRBV7 -6	2859 2	T cell receptor beta variable 7-6	TCRBV6S3A1N1T|TCRBV7S6|TRBV76	7	TCRsignalingPathway
TRBV7 -7	2859 1	T cell receptor beta variable 7-7	TCRBV6S6A2T|TCRBV7S7|TRBV77	7	TCRsignalingPathway
TRBV7 -8	2859 0	T cell receptor beta variable 7-8	TCRBV6S2A1N1T|TCRBV7S8|TRBV78	7	TCRsignalingPathway
TRBV7 -9	2858 9	T cell receptor beta variable 7-9	TCRB|TCRBV6S4A1|TCRBV7S9|TRBV79	7	TCRsignalingPathway
TRBV9	2858 6	T cell receptor beta variable 9	TCRBV1S1A1N1|TCRBV9S1	7	TCRsignalingPathway
TRBV1	2858	T cell receptor beta variable 10-1	TCRBV10S1|TCRBV12S2|TCRBV12S2A1T|TRB	7	TCRsignalingPathway

0-1	5		V101		
TRBV1<br>0-2	2858<br>4	T cell receptor beta variable 10-2	TCRBV10S2|TCRBV12S3|TRBV102	7	TCRsignalingPathway
TRBV1<br>0-3	2858<br>3	T cell receptor beta variable 10-3	TCRBV10S3|TCRBV12S1A1N2|TRBV103	7	TCRsignalingPathway
TRBV1<br>1-1	2858<br>2	T cell receptor beta variable 11-1	TCRBV11S1|TCRBV21S1|TRBV111	7	TCRsignalingPathway
TRBV1<br>1-2	2858<br>1	T cell receptor beta variable 11-2	TCRBV11S2|TCRBV21S3A2N2T|TRBV112	7	TCRsignalingPathway
TRBV1<br>1-3	2858<br>0	T cell receptor beta variable 11-3	TCRBV11S3|TCRBV21S2A2|TRBV113	7	TCRsignalingPathway
TRBV1<br>2-3	2857<br>7	T cell receptor beta variable 12-3	TCRBV12S3|TCRBV8S1|TRBV123	7	TCRsignalingPathway
TRBV1<br>2-4	2857<br>6	T cell receptor beta variable 12-4	TCRBV12S4|TCRBV8S2A1T|TRBV124	7	TCRsignalingPathway
TRBV1<br>2-5	2857<br>5	T cell receptor beta variable 12-5	TCRBV12S5|TCRBV8S3|TRBV125	7	TCRsignalingPathway
TRBV1<br>3	2857<br>4	T cell receptor beta variable 13	TCRBV13S1|TCRBV23S1A2T	7	TCRsignalingPathway
TRBV1<br>4	2857<br>3	T cell receptor beta variable 14	TCRBV14S1|TCRBV16S1A1N1	7	TCRsignalingPathway
TRBV1<br>5	2857<br>2	T cell receptor beta variable 15	TCRBV15S1|TCRBV24S1A3T	7	TCRsignalingPathway
TRBV1<br>6	2857<br>1	T cell receptor beta variable 16	BV25S1J1.2|TCRB|TCRBV16S1|TCRBV25S1| TCRBV25S1A2PT	7	TCRsignalingPathway
TRBV1	2857	T cell receptor beta variable 17	TCRBV17S1|TCRBV26S1P	7	TCRsignalingPathway

7	0	(non-functional)			
TRBV18	28569	T cell receptor beta variable 18	TCRBV18S1	7	TCRsignalingPathway
TRBV19	28568	T cell receptor beta variable 19	TCRBV17S1A1T|TCRBV19S1	7	TCRsignalingPathway
TRBV20-1	28567	T cell receptor beta variable 20-1	TCRBV20S1|TCRBV2S1|TRBV201	7	TCRsignalingPathway
TRBV24-1	28563	T cell receptor beta variable 24-1	TCRBV15S1|TCRBV24S1|TRBV241	7	TCRsignalingPathway
TRBV25-1	28562	T cell receptor beta variable 25-1	TCRBV11S1A1T|TCRBV25S1|TRBV251	7	TCRsignalingPathway
TRBV27	28560	T cell receptor beta variable 27	TCRBV14S1|TCRBV27S1	7	TCRsignalingPathway
TRBV28	28559	T cell receptor beta variable 28	TCRBV28S1|TCRBV3S1	7	TCRsignalingPathway
TRBV29-1	28558	T cell receptor beta variable 29-1	TCRBV29S1|TCRBV4S1A1T|TRBV291	7	TCRsignalingPathway
TRBV30	28557	T cell receptor beta variable 30	TCRBV20S1A1N2|TCRBV30S1	7	TCRsignalingPathway
TRDC	28526	T cell receptor delta constant	TCRD	14	TCRsignalingPathway
TRDD1	28525	T cell receptor delta diversity 1	-	14	TCRsignalingPathway
TRDD2	28524	T cell receptor delta diversity 2	-	14	TCRsignalingPathway
TRDD3	2852	T cell receptor delta diversity 3	TCRD	14	TCRsignalingPathway

	3				
TRDJ1	28522	T cell receptor delta joining 1	TCRD	14	TCRsignalingPathway
TRDJ2	28521	T cell receptor delta joining 2	-	14	TCRsignalingPathway
TRDJ3	28520	T cell receptor delta joining 3	-	14	TCRsignalingPathway
TRDJ4	28519	T cell receptor delta joining 4	-	14	TCRsignalingPathway
TRDV1	28518	T cell receptor delta variable 1	hDV101S1	14	TCRsignalingPathway
TRDV2	28517	T cell receptor delta variable 2	hDV102S1	14	TCRsignalingPathway
TRDV3	28516	T cell receptor delta variable 3	hDV103S1	14	TCRsignalingPathway
TRGV9	6983	T cell receptor gamma variable 9	TCRGV9|TRGC1|V2	7	TCRsignalingPathway
TRGV8	6982	T cell receptor gamma variable 8	TCRGV8|V1S8	7	TCRsignalingPathway
TRGV5	6978	T cell receptor gamma variable 5	TCRGV5|V1S5	7	TCRsignalingPathway
TRGV4	6977	T cell receptor gamma variable 4	TCRGV4|V1S4	7	TCRsignalingPathway
TRGV3	6976	T cell receptor gamma variable 3	TCRGV3|V1S3	7	TCRsignalingPathway
TRGV2	6974	T cell receptor gamma variable 2	TCRGV2|VIS2	7	TCRsignalingPathway
TRGJP2	6972	T cell receptor gamma joining P2	JP2|TCRGJP2	7	TCRsignalingPathway
TRGJP1	6971	T cell receptor gamma joining P1	JP1|TCRGJP1	7	TCRsignalingPathway
TRGJP	6970	T cell receptor gamma joining P	JP|TCRGJP	7	TCRsignalingPathway

TRGJ2	6969	T cell receptor gamma joining 2	J2|TCRGJ2		7	TCRsignalingPathway
TRGJ1	6968	T cell receptor gamma joining 1	J1|TCRGJ1		7	TCRsignalingPathway
TRGC2	6967	T cell receptor gamma constant 2	TCRGC2|TRGC2(2X)|TRGC2(3X)		7	TCRsignalingPathway
TRGC1	6966	T cell receptor gamma constant 1	C1|TCRG|TCRGC1		7	TCRsignalingPathway
TRAV6	6956	T cell receptor alpha variable 6	TCRAV5S1|TCRAV6S1		14	TCRsignalingPathway
BMP1	649	bone morphogenetic protein 1	OI13|PCOLC|PCP|PCP2|TLD		8	TGFb_Family_Member
BMP10	27302	bone morphogenetic protein 10	-		2	TGFb_Family_Member
BMP15	9210	bone morphogenetic protein 15	GDF9B|ODG2|POF4	X		TGFb_Family_Member
BMP2	650	bone morphogenetic protein 2	BDA2|BMP2A|SSFSC		20	TGFb_Family_Member
BMP3	651	bone morphogenetic protein 3	BMP-3A		4	TGFb_Family_Member
BMP4	652	bone morphogenetic protein 4	BMP2B|BMP2B1|MCOPS6|OFC11|ZYME		14	TGFb_Family_Member
BMP5	653	bone morphogenetic protein 5	-		6	TGFb_Family_Member
BMP6	654	bone morphogenetic protein 6	VGR|VGR1		6	TGFb_Family_Member
BMP7	655	bone morphogenetic protein 7	OP-1		20	TGFb_Family_Member
BMP8A	353500	bone morphogenetic protein 8a	OP-2		1	TGFb_Family_Member
BMP8B	656	bone morphogenetic protein 8b	BMP8|OP2		1	TGFb_Family_Member
GDF1	2657	growth differentiation factor 1	CERS1|CHTD6|DORV|DTGA3|LAG1|LASS1|RAI|UOG1		19	TGFb_Family_Member
GDF10	2662	growth differentiation factor 10	BIP|BMP-3b|BMP3B		10	TGFb_Family_Member
GDF11	10220	growth differentiation factor 11	BMP-11|BMP11		12	TGFb_Family_Member
GDF15	9518	growth differentiation factor 15	GDF-15|MIC-1|MIC1|NAG-1|PDF|PLAB|PTGFB		19	TGFb_Family_Member
GDF2	2658	growth differentiation factor 2	BMP-9|BMP9|HHT5		10	TGFb_Family_Member

GDF3	9573	growth differentiation factor 3	KFS3|MCOP7|MCOPCB6	12	TGFb_Family_Member
GDF5	8200	growth differentiation factor 5	BDA1C|BMP-14|BMP14|CDMP1|DUPANS|LAP-4|LAP4|OS5|SYM1B|SYNS2	20	TGFb_Family_Member
GDF6	392255	growth differentiation factor 6	BMP-13|BMP13|CDMP2|KFM|KFS|KFS1|KFSL|SGM1|SYNS4	8	TGFb_Family_Member
GDF7	151449	growth differentiation factor 7	BMP12	2	TGFb_Family_Member
GDF9	2661	growth differentiation factor 9	POF14	5	TGFb_Family_Member
GDNF	2668	glial cell derived neurotrophic factor	ATF|ATF1|ATF2|HFB1-GDNF|HSCR3	5	TGFb_Family_Member
INHA	3623	inhibin subunit alpha	-	2	TGFb_Family_Member
INHBA	3624	inhibin subunit beta A	EDF|FRP	7	TGFb_Family_Member
INHBB	3625	inhibin subunit beta B	-	2	TGFb_Family_Member
INHBC	3626	inhibin subunit beta C	IHBC	12	TGFb_Family_Member
INHBE	83729	inhibin subunit beta E	-	12	TGFb_Family_Member
LEFTY1	10637	left-right determination factor 1	LEFTB|LEFTYB	1	TGFb_Family_Member
LEFTY2	7044	left-right determination factor 2	EBAF|LEFTA|LEFTYA|TGFB4	1	TGFb_Family_Member
NODAL	4838	nodal growth differentiation factor	HTX5	10	TGFb_Family_Member
TGFB1	7040	transforming growth factor beta 1	CED|DPD1|IBDIMDE|LAP|TGF-beta1|TGFB|TGFbeta	19	TGFb_Family_Member
TGFB2	7042	transforming growth factor beta 2	G-TSF|LDS4|TGF-beta2	1	TGFb_Family_Member
TGFB3	7043	transforming growth factor beta 3	ARVD|ARVD1|LDS5|RNHF|TGF-beta3	14	TGFb_Family_Member
ACVR1B	91	activin A receptor type 1B	ACTRIB|ACVRLK4|ALK4|SKR2	12	TGFb_Family_Member_Receptor

ACVR1C	130399	activin A receptor type 1C	ACVRLK7|ALK7	2	TGFb_Family_Member_Receptor
ACVR2A	92	activin A receptor type 2A	ACTRII|ACVR2	2	TGFb_Family_Member_Receptor
ACVR2B	93	activin A receptor type 2B	ACTRIIB|ActR-IIB|HTX4	3	TGFb_Family_Member_Receptor
ACVRL1	94	activin A receptor like type 1	ACVRLK1|ALK-1|ALK1|HHT|HHT2|ORW2|SKR3|TSR-I	12	TGFb_Family_Member_Receptor
AMHR2	269	anti-Mullerian hormone receptor type 2	AMHR|MISR2|MISRII|MRII	12	TGFb_Family_Member_Receptor
BMPR1A	657	bone morphogenetic protein receptor type 1A	10q23del|ACVRLK3|ALK3|CD292|SKR5	10	TGFb_Family_Member_Receptor
BMPR1B	658	bone morphogenetic protein receptor type 1B	ALK-6|ALK6|AMDD|BDA1D|BDA2|CDw293	4	TGFb_Family_Member_Receptor
BMPR2	659	bone morphogenetic protein receptor type 2	BMPR-II|BMPR3|BMR2|BRK-3|POVD1|PPH1|T-ALK	2	TGFb_Family_Member_Receptor
TGFBR1	7046	transforming growth factor beta receptor 1	AAT5|ACVRLK4|ALK-5|ALK5|ESS1|LDS1|LDS1A|LDS2A|MSSE|SKR4|TBR-i|TBRI|TGFR-1|tbetaR-I	9	TGFb_Family_Member_Receptor
TGFBR2	7048	transforming growth factor beta receptor 2	AAT3|FAA3|LDS1B|LDS2|LDS2B|MFS2|RIIC|TAAD2|TBR-ii|TBRII|TGFR-2|TGFbeta-RII	3	TGFb_Family_Member_Receptor
TGFBR3	7049	transforming growth factor beta receptor 3	BGCAN|betaglycan	1	TGFb_Family_Member_Receptor
TNFRSF11B	4982	TNF receptor superfamily member 11b	OCIF|OPG|PDB5|TR1	8	TNF_Family_Members
TNFSF	8743	TNF superfamily member 10	APO2L|Apo-2L|CD253|TL2|TNLG6A|TRAIL	3	TNF_Family_Members

10					
TNFSF 11	8600	TNF superfamily member 11	CD254|ODF|OPGL|OPTB2|RANKL|TNLG6B|TRANCE|hRANKL2|sOdf	13	TNF_Family_Members
TNFSF 12	8742	TNF superfamily member 12	APO3L|DR3LG|TNLG4A|TWEAK	17	TNF_Family_Members
TNFSF 13	8741	TNF superfamily member 13	APRIL|CD256|TALL-2|TALL2|TNLG7B|TRDL-1|UNQ383/PRO715|ZTNF2	17	TNF_Family_Members
TNFSF 13B	10673	TNF superfamily member 13b	BAFF|BLYS|CD257|DTL|TALL-1|TALL1|THANK|TNFSF20|TNLG7A|ZTNF4	13	TNF_Family_Members
TNFSF 14	8740	TNF superfamily member 14	CD258|HVEML|LIGHT|LTg	19	TNF_Family_Members
TNFSF 15	9966	TNF superfamily member 15	TL1|TL1A|TNLG1B|VEGI|VEGI192A	9	TNF_Family_Members
TNFSF 18	8995	TNF superfamily member 18	AITRL|GITRL|TL6|TNLG2A|hGITRL	1	TNF_Family_Members
TNFSF 4	7292	TNF superfamily member 4	CD134L|CD252|GP34|OX-40L|OX40L|TNLG2B|TXGP1	1	TNF_Family_Members
TNFSF 8	944	TNF superfamily member 8	CD153|CD30L|CD30LG|TNLG3A	9	TNF_Family_Members
TNFSF 9	8744	TNF superfamily member 9	4-1BB-L|CD137L|TNLG5A	19	TNF_Family_Members
TNFRSF10B	8795	TNF receptor superfamily member 10b	CD262|DR5|KILLER|KILLER/DR5|TRAIL-R2|TRAILR2|TRICK2|TRICK2A|TRICK2B|TRICKB|ZTNFR9	8	TNF_Family_Members_Receptors
TNFRSF10C	8794	TNF receptor superfamily member 10c	CD263|DCR1|DCR1-TNFR|LIT|TRAIL-R3|TRAILR3|TRID	8	TNF_Family_Members_Receptors

TNFRSF10D	8793	TNF receptor superfamily member 10d	CD264|DCR2|TRAIL-R4|TRAILR4|TRUNDD	8	TNF_Family_Members_Receptors
TNFRSF11A	8792	TNF receptor superfamily member 11a	CD265|FEO|LOH18CR1|ODFR|OFE|OPTB7|OSTS|PDB2|RANK|TRANCER	18	TNF_Family_Members_Receptors
TNFRSF12A	51330	TNF receptor superfamily member 12A	CD266|FN14|TWEAKR	16	TNF_Family_Members_Receptors
TNFRSF13B	23495	TNF receptor superfamily member 13B	CD267|CVID|CVID2|IGAD2|RYZN|TACI|TNFRSF14B	17	TNF_Family_Members_Receptors
TNFRSF13C	115650	TNF receptor superfamily member 13C	BAFF-R|BAFFR|BROMIX|CD268|CVID4|prolixin	22	TNF_Family_Members_Receptors
TNFRSF14	8764	TNF receptor superfamily member 14	ATAR|CD270|HVEA|HVEM|LIGHTR|TR2	1	TNF_Family_Members_Receptors
TNFRSF17	608	TNF receptor superfamily member 17	BCM|BCMA|CD269|TNFRSF13A	16	TNF_Family_Members_Receptors
TNFRSF18	8784	TNF receptor superfamily member 18	AITR|CD357|ENERGEN|GITR|GITR-D	1	TNF_Family_Members_Receptors
TNFRSF19	55504	TNF receptor superfamily member 19	TAJ|TAJ-alpha|TRADE|TROY	13	TNF_Family_Members_Receptors
TNFRSF1A	7132	TNF receptor superfamily member 1A	CD120a|FPF|TBP1|TNF-R|TNF-R-I|TNF-R55|TNFAR|TNFR1|TNFR55|TNFR60|p55|p55-R|p60	12	TNF_Family_Members_Receptors
TNFRSF1B	7133	TNF receptor superfamily member 1B	CD120b|TBPII|TNF-R-II|TNF-R75|TNFBR|TNFR1B|TNFR2|TNFR80|p75|p75TNFR	1	TNF_Family_Members_Receptors
TNFRSF21	27242	TNF receptor superfamily member 21	BM-018|CD358|DR6	6	TNF_Family_Members_Receptors
TNFRS	8718	TNF receptor superfamily member 25	APO-3|DDR3|DR3|GEF720|LARD|PLEKHG5|T	1	TNF_Family_Members_Receptors

F25			NFRSF12|TR3|TRAMP|WSL-1|WSL-LR		
TNFRSF4	7293	TNF receptor superfamily member 4	ACT35|CD134|IMD16|OX40|TXGP1L	1	TNF_Family_Members_Receptors
TNFRSF6B	8771	TNF receptor superfamily member 6b	DCR3|DJ583P15.1.1|M68|M68E|TR6	20	TNF_Family_Members_Receptors
TNFRSF8	943	TNF receptor superfamily member 8	CD30|D1S166E|Ki-1	1	TNF_Family_Members_Receptors
TNFRSF9	3604	TNF receptor superfamily member 9	4-1BB|CD137|CDw137|ILA	1	TNF_Family_Members_Receptors
