# Supplementary material for: Identification and Validation of STC1 Act as a Biomarker for High-Altitude Diseases and Its Pan-Cancer Analysis
Source: Int J Mol Sci. 2024 Aug 21;25(16):9085. doi: 10.3390/ijms25169085 (PMC11354978; doi:10.3390/ijms25169085)
Supplement: Supplementary file 1 [file ijms-25-09085-s001.zip › Supplementary Table S6.pdf]

Supplementary Table S6.Differential expression of the STC1 gene in samples with different clinical stages

| Label | tumor group(Mean±std) | control group(Mean±std) | t-test | ANOVA |
|---|---|---|---|---|
| LUAD(T1=169,T2=276,T3=47,T4=18) | T1(2.10±1.48) | T2(2.55±1.60) | 0 | 0 |
| | T1(2.10±1.48) | T3(2.58±1.61) | 0.07 | |
| | T1(2.10±1.48) | T4(3.44±1.78) | 0.01 | |
| | T2(2.55±1.60) | T3(2.58±1.61) | 0.91 | |
| | T2(2.55±1.60) | T4(3.44±1.78) | 0.05 | |
| | T3(2.58±1.61) | T4(3.44±1.78) | 0.09 | |
| COADREAD(T1=10,T2=57,T3=259,T4=50) | T3(2.02±1.30) | T4(2.12±1.20) | 0.6 | 0.02 |
| | T3(2.02±1.30) | T1(1.26±1.34) | 0.11 | |
| | T3(2.02±1.30) | T2(1.57±1.23) | 0.01 | |
| | T4(2.12±1.20) | T1(1.26±1.34) | 0.08 | |
| | T4(2.12±1.20) | T2(1.57±1.23) | 0.02 | |
| | T1(1.26±1.34) | T2(1.57±1.23) | 0.5 | |
| STES(T1=52,T2=132,T3=277,T4=121) | T3(3.37±1.22) | T1(2.60±1.70) | 0 | 0 |
| | T3(3.37±1.22) | T4(3.45±1.03) | 0.5 | |
| | T3(3.37±1.22) | T2(3.34±1.36) | 0.85 | |
| | T1(2.60±1.70) | T4(3.45±1.03) | 0 | |
| | T1(2.60±1.70) | T2(3.34±1.36) | 0.01 | |
| | T4(3.45±1.03) | T2(3.34±1.36) | 0.49 | |
| KIRP(T1=191,T2=33,T3=60) | T1(0.81±1.79) | T3(1.66±1.98) | 0 | 0 |
| | T1(0.81±1.79) | T2(1.33±1.68) | 0.11 | |
| | T3(1.66±1.98) | T2(1.33±1.68) | 0.39 | |
| KIPAN(T1=484,T2=127,T3=257,T4=14) | T1(3.53±2.81) | T3(4.40±2.28) | 0 | 0 |
| | T1(3.53±2.81) | T2(3.77±2.45) | 0.33 | |

| | | | | |
|---|---|---|---|---|
| | T1(3.53±2.81) | T4(4.31±2.06) | 0.18 | |
| | T3(4.40±2.28) | T2(3.77±2.45) | 0.02 | |
| | T3(4.40±2.28) | T4(4.31±2.06) | 0.89 | |
| | T2(3.77±2.45) | T4(4.31±2.06) | 0.38 | |
| STAD(T1=21,T2=89,T3=180,T4=115) | T3(3.27±1.13) | T4(3.47±1.04) | 0.12 | 0 |
| | T3(3.27±1.13) | T2(3.16±1.21) | 0.48 | |
| | T3(3.27±1.13) | T1(1.96±1.74) | 0 | |
| | T4(3.47±1.04) | T2(3.16±1.21) | 0.06 | |
| | T4(3.47±1.04) | T1(1.96±1.74) | 0 | |
| | T2(3.16±1.21) | T1(1.96±1.74) | 0.01 | |
| HNSC(T1=51,T2=157,T3=120,T4=189) | T4(2.68±1.51) | T1(1.87±1.26) | 0 | 0 |
| | T4(2.68±1.51) | T3(2.67±1.46) | 0.97 | |
| | T4(2.68±1.51) | T2(2.15±1.57) | 0 | |
| | T1(1.87±1.26) | T3(2.67±1.46) | 0 | |
| | T1(1.87±1.26) | T2(2.15±1.57) | 0.19 | |
| | T3(2.67±1.46) | T2(2.15±1.57) | 0 | |
| LUSC(T1=112,T2=292,T3=71,T4=23) | T2(3.13±1.39) | T1(2.71±1.32) | 0.01 | 0.05 |
| | T2(3.13±1.39) | T4(2.91±1.51) | 0.5 | |
| | T2(3.13±1.39) | T3(3.12±1.36) | 0.95 | |
| | T1(2.71±1.32) | T4(2.91±1.51) | 0.57 | |
| | T1(2.71±1.32) | T3(3.12±1.36) | 0.05 | |
| | T4(2.91±1.51) | T3(3.12±1.36) | 0.56 | |
| BLCA(T1=3,T2=150,T3=195,T4=58) | T3(2.64±1.50) | T4(2.62±1.67) | 0.94 | 0 |
| | T3(2.64±1.50) | T2(2.01±1.50) | 0 | |
| | T3(2.64±1.50) | T1(2.82±0.31) | 0.45 | |
| | T4(2.62±1.67) | T2(2.01±1.50) | 0.02 | |

| | | | |
|---|---|---|---|
| | T4(2.62±1.67) | T1(2.82±0.31) | 0.51 |
| | T2(2.01±1.50) | T1(2.82±0.31) | 0.02 |
